# Synthesis of All‐Carbon Disubstituted Bicyclo[1.1.1]pentanes by Iron‐Catalyzed Kumada Cross‐Coupling†

**DOI:** 10.1002/anie.202004090

**Published:** 2020-05-14

**Authors:** Jeremy Nugent, Bethany R. Shire, Dimitri F. J. Caputo, Helena D. Pickford, Frank Nightingale, Ian T. T. Houlsby, James J. Mousseau, Edward A. Anderson

**Affiliations:** ^1^ Chemistry Research Laboratory University of Oxford 12 Mansfield Road Oxford OX1 3TA UK; ^2^ Syngenta Ltd. Jealott's Hill International Research Centre Bracknell RG42 6EY UK; ^3^ Pfizer Medicine Design Eastern Point Road Groton CT 06340 USA

**Keywords:** bicyclopentane, bioisosteres, cross-coupling, homogeneous catalysis, iron

## Abstract

1,3‐Disubstituted bicyclo[1.1.1]pentanes (BCPs) are important motifs in drug design as surrogates for *p*‐substituted arenes and alkynes. Access to all‐carbon disubstituted BCPs via cross‐coupling has to date been limited to use of the BCP as the organometallic component, which restricts scope due to the harsh conditions typically required for the synthesis of metallated BCPs. Here we report a general method to access 1,3‐*C*‐disubstituted BCPs from 1‐iodo‐bicyclo[1.1.1]pentanes (iodo‐BCPs) by direct iron‐catalyzed cross‐coupling with aryl and heteroaryl Grignard reagents. This chemistry represents the first general use of iodo‐BCPs as electrophiles in cross‐coupling, and the first Kumada coupling of tertiary iodides. Benefiting from short reaction times, mild conditions, and broad scope of the coupling partners, it enables the synthesis of a wide range of 1,3‐*C*‐disubstituted BCPs including various drug analogues.

## Graphical abstract

**1,3‐*C*‐disubstituted bicyclo[1.1.1]pentanes (BCPs)** are accessed from 1‐iodo‐BCPs by direct iron‐catalyzed cross‐coupling with aryl and heteroaryl Grignard reagents. This represents the first general use of iodo‐BCPs as cross‐coupling electrophiles, and the first Kumada coupling of tertiary iodides in general. Benefiting from short reaction times, mild conditions, and broad scope, it enables the synthesis of a wide range of BCPs including drug analogues.

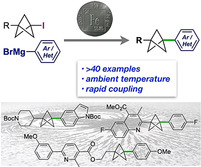

1,3‐Disubstituted bicyclo[1.1.1]pentanes (BCPs) are of high interest in drug discovery as bioisosteres for 1,4‐disubstituted arenes and alkynes (Figure 1 a).^1^ Incorporation of these sp^3^‐rich motifs into drug leads often results in pharmacological benefits such as improved solubility, membrane permeability and metabolic stability.^2^ However, access to promising BCP‐bearing compounds2b, 3 can be impeded by lengthy and unscalable reaction sequences, in particular where two carbon substituents are required. These challenges have inspired the development of a number of methods to synthesize 1,3‐*C*‐disubstituted BCPs in which installation of the carbon substituents relies on addition of an organometallic nucleophile to the strained C1–C3 σ‐bond of [1.1.1]propellane,1e, 4 followed by palladium‐catalyzed cross‐coupling of the resulting metallated BCP (Figure 1 b).1e, 4a, 5 While such methods can generate useful products, the harsh conditions required to achieve the initial nucleophilic addition limit the suitability of this chemistry for industrial applications.

**Figure 1:**
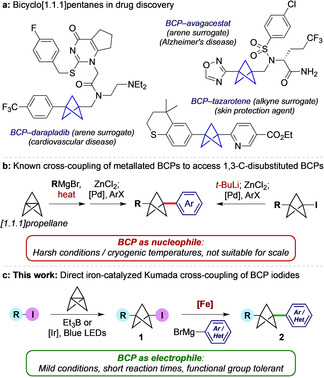
a) Examples of bicyclo[1.1.1]pentanes (BCPs) in medicinal chemistry; b) known cross‐coupling of metallated BCPs; c) this work: Direct iron‐catalyzed cross‐coupling of iodo‐BCPs with aryl/ heteroaryl Grignard reagents.

1‐Iodobicyclo[1.1.1]pentanes (iodo‐BCPs, **1**) are attractive substrates for the introduction of carbon substituents on the BCP skeleton. We recently described efficient and functional group‐tolerant conditions to access these compounds by atom transfer radical addition of C–I bonds to [1.1.1]propellane, under photoredox catalysis^6^ or using triethylborane as initiator.^7^ As direct palladium‐catalyzed cross‐coupling of iodo‐BCPs can suffer from competing ring fragmentation,^8^ approaches to all‐carbon disubstituted BCPs from iodo‐BCPs have to date necessitated lithiation of the iodide, followed by cross‐coupling as the nucleophilic component (Figure 1 b, or reaction with other carbon‐based electrophiles).1e, 4a However, the conditions needed for lithiation of the iodo‐BCP again limit scope and scalability.

We targeted an alternative, catalytic method to access all‐carbon disubstituted BCPs (**2**, Figure 1 c) directly from iodo‐BCPs **1**, under mild conditions and without recourse to organolithium reagents. Iron‐catalyzed Kumada cross‐couplings of aryl Grignard reagents with secondary alkyl iodides are an efficient means to achieve sp^3^–sp^2^ C−C bond formation,^9^ and we questioned whether the tertiary iodide resident in an iodo‐BCP could engage in this coupling manifold. While isolated examples of Fe‐catalyzed Kumada couplings of tertiary alkyl bromides and chlorides have been described,9d, 10 the equivalent reaction of tertiary iodides is, to our knowledge, unknown. Here we describe the development of iron‐catalyzed cross‐coupling reactions of iodo‐BCPs with both aryl and heteroaryl Grignard reagents, which represents the first general procedure for the direct cross‐coupling of iodo‐BCP electrophiles.^11^ The chemistry proceeds under mild conditions and short reaction times, displays wide functional group tolerance, and is applicable to the synthesis of drug‐like molecules.

Our studies began with the coupling of iodo‐BCP **1 a** with *p*‐methoxyphenylmagnesium bromide (1.6 equiv, 0.7 mL h^−1^), which afforded small amounts of coupled product **2 a** using Fe(acac)_3_ or FeCl_3_ (20 mol %) as catalyst (Table 1, Entries 1, 2); the main byproduct was the dehalogenated BCP **3**. Notably, no reaction was seen using Cu(acac)_2_ (Entry 3). A number of amine and phosphorus ligands were investigated (Entries 4–9),^12^ among which TMEDA9a, 9b, 13 (40 mol %) provided the highest yields of **2 a** using either a standard Grignard reagent (PMPBr/Mg, 86 %, Entry 8), or the turbo‐Grignard (PMPBr/Mg/LiCl, 79 %, Entry 9). THF was found to be a superior solvent compared to others such as MTBE, 2‐MeTHF, and toluene (Entries 10–12).9g, 14 In spite of the failure of Cu(acac)_2_ alone to catalyze the reaction, we were mindful of the potential presence of trace metals in the (97 % purity) Fe(acac)_3_.^15^ However, equivalent reaction efficiency was observed using ≥99.9 % Fe(acac)_3_ (Entry 13), and no reaction with Cu(acac)_2_/TMEDA (Entry 14), suggesting this to indeed be an iron‐catalyzed transformation. Reducing the loading of catalyst to 10 mol % led to lower yields (Entry 15).

**Table 1:** Reaction optimization.^[a]^ 
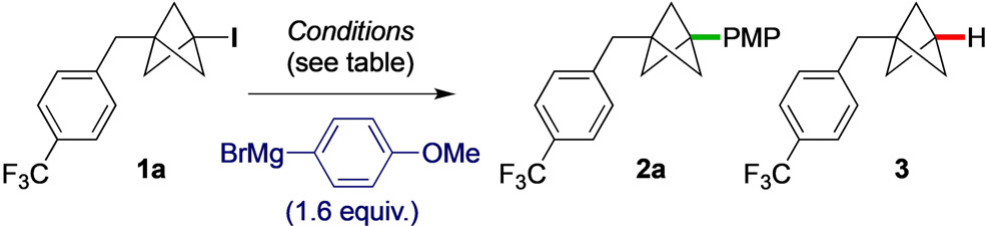

Entry	Catalyst (20 mol %)	Additive (40 mol %)	Solvent	Yield^[b]^ (**2 a**:**1 a**:**3**)
1	Fe(acac)_3_	–	THF	13:30:9
2	FeCl_3_	–	THF	4:23:9
3	Cu(acac)_2_	–	THF	0:100:0
4	Fe(acac)_3_	DMEDA	THF	5:22:9
5	Fe(acac)_3_	TMCD	THF	9:76:1
6	Fe(acac)_3_	1,2‐DPE	THF	46:33:8
7	Fe(acac)_3_	dcypt	THF	55:20:11
**8**	**Fe(acac)_3_**	**TMEDA**	**THF**	**90:0:2 (86)** **(86 %)**
**9^[c]^**	**Fe(acac)_3_**	**TMEDA**	**THF**	**86:0:4 (79)** **(86 %)**
10	Fe(acac)_3_	TMEDA	2‐MeTHF	71:15:2
11	Fe(acac)_3_	TMEDA	MTBE	70:16:2
12	Fe(acac)_3_	TMEDA	toluene	79:0:3
13^[d]^	Fe(acac)_3_	TMEDA	THF	92:0:2
14	Cu(acac)_2_	TMEDA	THF	0:100:0
15^[e]^	Fe(acac)_3_	TMEDA	THF	75:7:3

[a] **1 a** (0.2 mmol), catalyst (20 mol %), additive (40 mol %), solvent (0.2 mL), 20 °C; then add PMPMgBr (0.8 m in THF, 0.32 mmol) added at 0.7 mL h^−1^; then stir, 20 °C, 1 h. [b] Yields determined by ^1^H NMR spectroscopic analysis using 1,3,5‐trimethoxybenzene as an internal standard. Isolated yield in parentheses. [c] Using PMPMgBr⋅LiCl (1 m in THF). [d] Using ≥99.9 % Fe(acac)_3_. [e] Using 10 mol % Fe(acac)_3_, 20 mol % TMEDA. acac=acetoacetonate. DMEDA=*N*,*N′*‐dimethylethylenediamine. dcypt=3,4‐bis(dicyclohexylphosphino)thiophene. 1,2‐DPE=1,2‐dipiperidinoethane. MTBE=methyl *tert*‐butyl ether. PMP=4‐methoxyphenyl. TMCD=*N*,*N*,*N′*,*N′‐trans*‐tetramethylcyclohexanediamine. TMEDA=*N*,*N*,*N′*,*N′*‐tetramethylethylenediamine.

With optimized conditions in hand (Table 1, Entries 8/9), the scope of the aryl Grignard coupling partner was examined (Figure 2) using *p*‐trifluoromethylbenzyl (**1 a**) and 4‐*N*‐Boc‐piperidyl (**1 b**) iodo‐BCPs as representative substrates. Both electron‐rich and moderately electron‐poor Grignard reagents gave good to excellent yields of the coupled products for *para*‐substituted aryl organometallics (**2 b**–**2 m**, 53–83 %), with the reaction of 4‐trimethylsilylphenylmagnesium bromide also delivering an excellent yield of **2 m** on 2.7 mmol scale (90 %). Substitution at the *meta* and *ortho* positions was well tolerated (**2 n**–**2 r**, 55–86 %), although in the latter cases elevated temperatures (45 °C) were required. Bicyclic and tri‐substituted Grignard reagents gave similarly high yields of BCP products **2 s**–**2 u** (70–72 %).^16^

**Figure 2:**
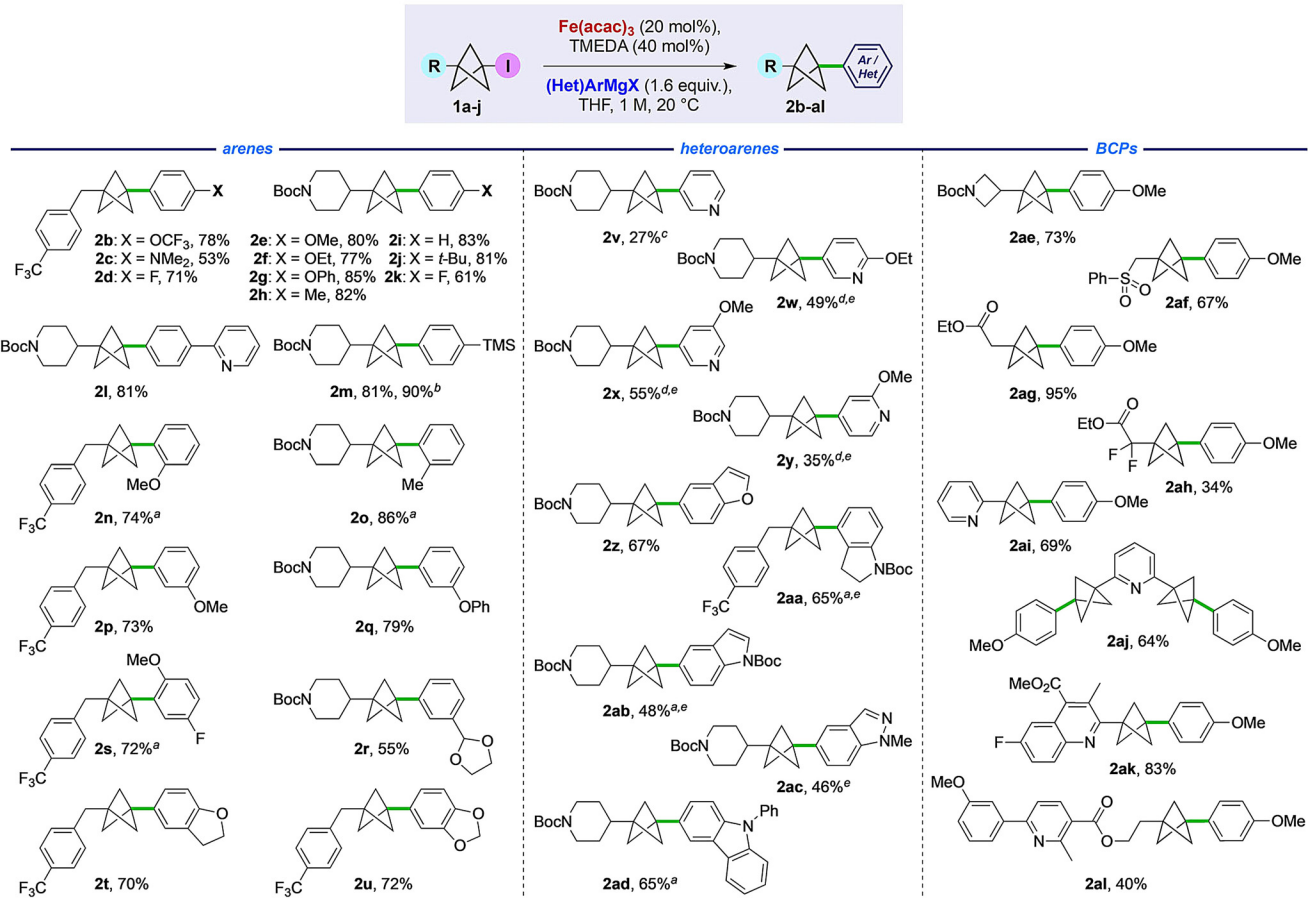
Cross‐coupling of electron rich, electron neutral, aryl and heteroaryl Grignard reagents with iodo‐BCPs. a) Reaction performed at 45 °C. b) 1.0 g scale. c) Reaction performed at 65 °C with rapid addition of the Grignard reagent. d) Reaction performed at 45 °C with 3 equiv of Grignard reagent. e) Using (Het)ArMgBr⋅LiCl.

The direct coupling of heteroaromatic Grignard reagents would be of high interest in a pharmaceutical context. To our delight, a variety of heteroaryl Grignards underwent successful reaction to give heteroaryl‐functionalized BCPs **2 v**–**2 ad**, albeit warming was required for electron‐deficient heterocycles (45 °C). The unsubstituted 3‐pyridyl Grignard reagent gave **2 v** in a modest 27 % yield, however significant improvement was observed with more electron‐rich pyridines (**2 w**–**2 y**, 35–55 %), although reactions of 2‐pyridyl Grignard reagents were unsuccessful. Cross‐couplings of benzofuran, *N*‐Boc‐indoline, *N*‐Boc‐indole, *N*‐methyl indazole, and *N*‐phenyl carbazole organometallics were successful, giving good yields of the heteroarylated products **2 z**–**2 ad** (46–67 %).

The scope of the iodo‐BCP coupling partner was next investigated in couplings with PMPMgBr. An *N*‐Boc azetidine‐substituted BCP gave product **2 ae** in excellent yield (73 %). Electron‐withdrawing groups were well‐tolerated, such as sulfone **2 af**, and the electrophilic ester **2 ag** (67 % and 95 % respectively); in the latter case, no addition of the Grignard to the ester was observed. However, a more electrophilic α,α‐difluoro ester proved less successful (**2 ah**), likely due to competing addition to the carbonyl. Heteroaryl‐substituted iodo‐BCPs also proved good substrates, affording the bis‐arylated BCP **2 ai** in 69 % yield, and double cross‐coupled product **2 aj** in 64 % yield. The chemistry was applied to more complex iodides, with quinoline **2 ak** and nicotinic acid derivative **2 al** being formed in 83 % and 40 % yields, respectively. These examples emphasize the mild conditions and functional group tolerance of this methodology.

The mechanistic pathways of iron‐catalyzed cross‐coupling reactions are dependent on a number of factors, including the nature of the Grignard reagent, the rate of its addition, the additive (ligand), and solvent.9i, 17 The formation of iron nanoparticles may also be observed, in particular under a “rapid addition” (of Grignard) regime.^18^ In our system, a distinctive colour change from orange/red to dark/black was observed during Grignard addition which may indicate nanoparticle formation,^19^ albeit this is not commonly observed under “slow” addition regimes.13a The presence of substoichiometric TMEDA is also clearly beneficial to reaction efficiency,9j, 20 although its role is unclear given evidence that it may not be ligated to the metal during the coupling process.^21^

In keeping with couplings carried out using less sterically‐hindered aryl Grignard reagents with amine additives, we therefore favour a reaction pathway involving single electron transfer from an Fe^I^ species such as **4** 17c, 22 to the iodo‐BCP (Scheme 1), generating a bicyclopentyl radical **5**.^23^ Reaction of this species with the LArFe(II) complex/ArMgBr liberates the cross‐coupled product and results in catalyst turnover; however, the precise mechanism by which C−C bond formation occurs (e.g. in cage/out of cage) is not apparent.17a, 24

**Scheme 1:**
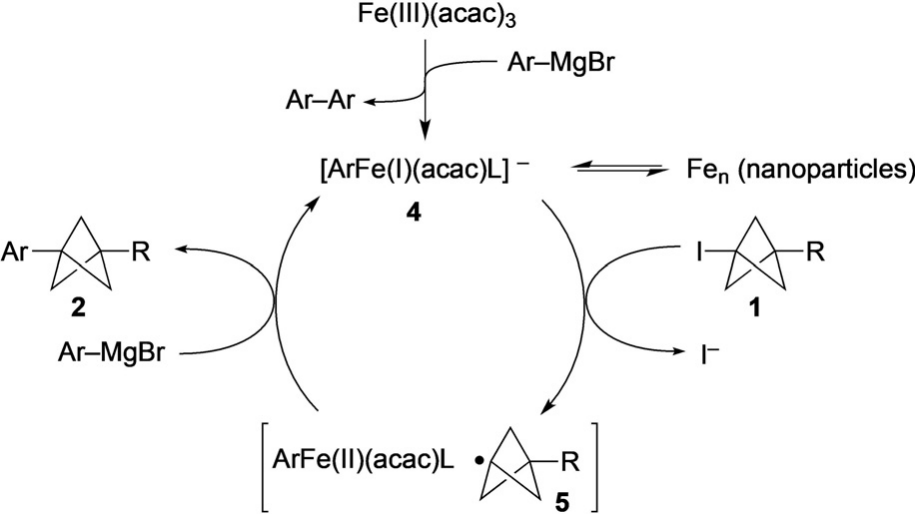
Putative reaction mechanism.

When combined with our previous methods for iodo‐BCP synthesis, the Kumada cross‐coupling offers a powerful method for the mild and rapid generation of valuable, pharmaceutically‐relevant 1,3‐*C*‐difunctionalized BCPs. To demonstrate potential utility, we targeted BCP analogues of the anti‐inflammatory drug flurbiprofen, and the anti‐neoplastic agent brequinar (Scheme 2 a). The requisite iodo‐BCPs **1 k** and **1 i** were synthesized in excellent yields from reaction of [1.1.1]propellane with commercially available ethyl iodopropanoate **6** (80 %), and iodoquinoline **7** (85 %),^8^ using Et_3_B initiation and photoredox catalysis (Ir(ppy)_3_/blue LEDs), respectively. Kumada cross‐coupling of iodo‐BCP ester **1 k** with PhMgBr, followed by hydrolysis, furnished BCP‐flurbiprofen **8** in 78 % yield; coupling of 2‐pyridyl iodo‐BCP **1 i** with 4‐fluorophenylmagnesium bromide afforded brequinar analogue **9** (66 %).^25^ Finally, access to aryl‐BCPs featuring substituents not tolerated under Kumada coupling could be achieved by *ipso*‐substitution^26^ of aryl silane **2 m** (Scheme 2 b), for example with halides suitable for further elaboration by cross‐coupling (**10**, **11**),^27^ or an electron‐withdrawing acetyl group (**12**).^28^

**Scheme 2:**
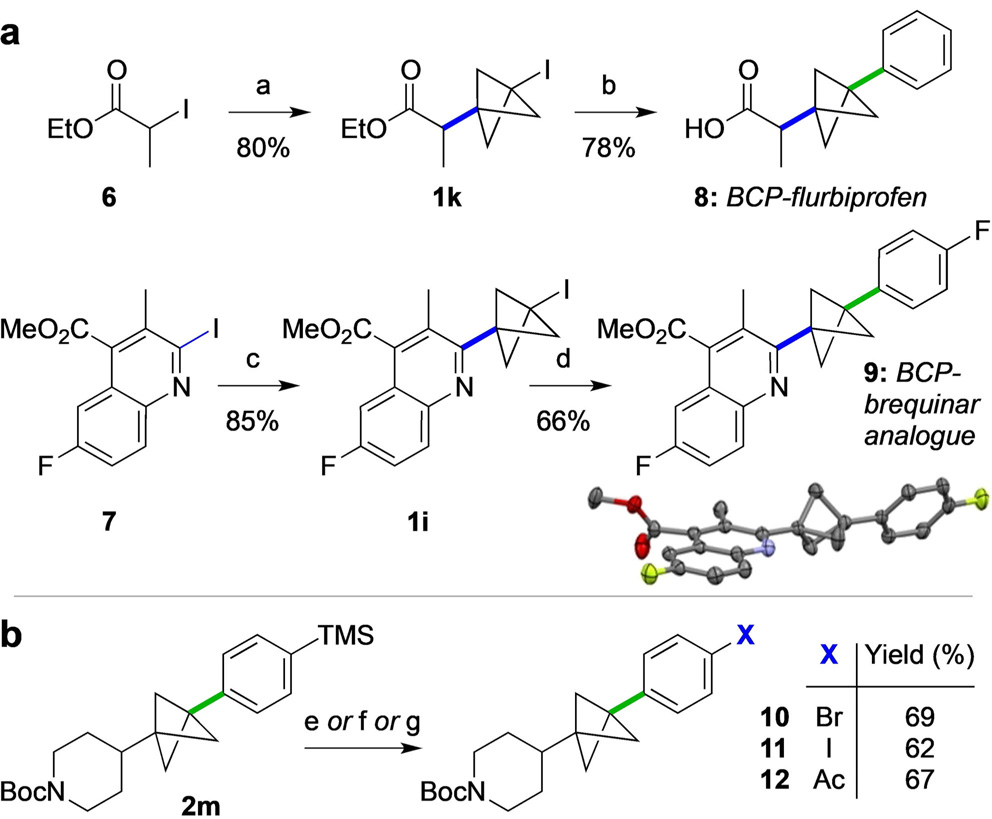
a) Synthesis of BCP drug analogues. b) Further functionalization. Reagents and Conditions: a) [1.1.1]propellane, Et_3_B (10 mol %), Et_2_O, 0 °C, 15 min; b) Fe(acac)_3_ (20 mol %), TMEDA (40 mol %), PhMgBr, THF, rt, 1 h, then NaOH/ MeOH; c) Ir(ppy)_3_ (2.5 mol %), [1.1.1]propellane, *t*‐BuCN, blue LEDs, 24 h, 30 °C; d) Fe(acac)_3_ (20 mol %), TMEDA (40 mol %), 4‐FPhMgBr, THF, rt, 1 h; e) KBr, NCS, AcOH/ MeOH, rt; f) ICl, CH_2_Cl_2_, 0 °C; g) AcCl, AlCl_3_, CH_2_Cl_2_, 0 °C→rt; then Boc_2_O, Et_3_N, CH_2_Cl_2_, rt. ppy=2‐phenylpyridine.

In conclusion, we have developed a mild, efficient iron‐catalyzed cross‐coupling of iodo‐BCPs and (hetero)aryl Grignard reagents, which represents the first such example of Kumada cross‐coupling of tertiary iodides. The reaction is rapid, exhibits good functional group tolerance, and performs well on gram scale.

Applications to the functionalization of pharmaceutical derivatives, including the synthesis of two BCP drug analogues, demonstrate the potential of this transformation to access highly functionalized 1,3‐C‐disubstituted BCPs of direct relevance in medicinal chemistry settings.

## Conflict of interest

The authors declare no conflict of interest.

## Supporting information

As a service to our authors and readers, this journal provides supporting information supplied by the authors. Such materials are peer reviewed and may be re‐organized for online delivery, but are not copy‐edited or typeset. Technical support issues arising from supporting information (other than missing files) should be addressed to the authors.

Supplementary
