# Supplementary material for: Synthesis of All‐Carbon Disubstituted Bicyclo[1.1.1]pentanes by Iron‐Catalyzed Kumada Cross‐Coupling
Source: Angew Chem Int Ed Engl. 2020 May 14;59(29):11866–70. doi: 10.1002/anie.202004090 (PMC7383991; doi:10.1002/anie.202004090)

Angewandte Chemie
Eine Zeitschrift der Gesellschaft Deutscher Chemiker

Supporting Information

Synthesis of All-Carbon Disubstituted Bicyclo[1.1.1]pentanes by Iron-Catalyzed Kumada Cross-Coupling**

*Jeremy Nugent[+], Bethany R. Shire[+], Dimitri F. J. Caputo, Helena D. Pickford, Frank Nightingale, Ian T. T. Houlsby, James J. Mousseau, and Edward A. Anderson**

anie_202004090_sm_miscellaneous_information.pdf

Author Contributions

J.N. Investigation: Equal; Methodology: Equal; Writing—Original Draft: Supporting; Writing—Review & Editing: Supporting
B.S. Investigation: Equal; Methodology: Equal; Writing—Original Draft: Supporting; Writing—Review & Editing: Supporting
D.C. Conceptualization: Supporting; Investigation: Supporting; Methodology: Supporting
H.P. Formal analysis: Supporting; Investigation: Supporting; Methodology: Supporting.

Table of Contents

1. General Experimental Considerations 2

2. General Procedures 3

3. Optimization of Kumada Coupling 4

4. Competition Experiments and Mechanistic Discussion 7

5. Experimental Procedures and Characterization Data 9

- **a)** Synthesis of Iodo-bicyclo[1.1.1]pentanes 9
- **b)** Palladium-catalyzed iodo-BCP fragmentation 15
- **c)** Kumada cross-coupling products 16
- **d)** TMS ipso-substitution reactions 44
- **f)** Unsuccessful couplings 47
- **g)** References 48

6. X-ray Crystallography 49

7. Copies of NMR Spectra 52

1. General Experimental Considerations

NMR Spectroscopy: ^{1}H, ^{13}C and ^{19}F NMR spectra were recorded on Bruker AV400 or Bruker AVII500 spectrometers using TOPSPIN software, with the deuterated solvent acting as the internal deuterium lock. ^{1}H NMR spectra were recorded at 400 or 500 MHz, ^{13}C NMR spectra were recorded at 101 or 126 MHz with ^{1}H decoupling, and ^{19}F NMR spectra were recorded at 376 or 470 MHz. Assignments were made either on the basis of unambiguous chemical shift / coupling patterns, or from 2D COSY, HMBC, HSQC and / or NOESY experiments. Peak multiplicities are defined as: s = singlet, d = doublet, t = triplet, q = quartet, quin = quintet, m = multiplet, br = broad, app = apparent; coupling constants (J) are reported to the nearest 0.1 Hz.

Infrared Spectroscopy: Infrared spectra were recorded on a Bruker Tensor 27 FT-IR spectrometer with the sample being prepared as a thin film on a diamond ATR module. Absorption maxima (ν_{max}) are quoted in wavenumbers (cm^{-1}).

Mass Spectrometry: Low resolution mass spectra were recorded on a Micromass LCT Premier Open Access using electrospray ionization (ESI). Accurate mass (HRMS) data was determined under conditions of ESI, EI and CI on a Bruker MicroTOF. High resolution values are calculated to 4 decimal places from the molecular formula, and all values are within a tolerance of 5 ppm.

Melting Points: Melting points were obtained using a Griffin melting point apparatus and are uncorrected.

Reagents, solvents and techniques: All reagents were used directly as supplied. Solvents were either used as commercially supplied or purified by standard techniques. Anhydrous Et_2O, CH_2Cl_2 and toluene were obtained from solvent dispenser units having been passed through an activated alumina column under argon. $Fe(acac)_3$ (97%) and $Fe(acac)_3$ (≥99.9%), TMEDA (≥99.5%, purified by redistillation) were supplied by Sigma Aldrich. Unless stated otherwise, non-aqueous reactions were performed using flame-dried glassware under a N_2 atmosphere.

Reactions were monitored by thin layer chromatography on pre-coated aluminium-backed plates (Merck Kieselgel 60 with fluorescent indicator UV254). Spots were visualized by quenching of UV fluorescence or by staining with potassium permanganate or vanillin, and retention factors are reported with the solvent system in parentheses. Flash column chromatography was performed on silica gel obtained from Merck (Silica gel Si 60, 0.04-0.063 mm) under a positive pressure of nitrogen, using the stated solvent system.

2. General Procedures

General Procedure 1: BEt_3-initiated bicyclopentylation of alkyl iodides

To a vial containing the specified halide (1.0 equiv.) was added [1.1.1]propellane (1.1-2.0 equiv., 0.5-0.7 M solution in Et_2O). BEt_3 (10 mol%, 1 M in hexane) was added to the solution via syringe (needle tip in the solution). After the indicated time the reaction was concentrated *in vacuo* and purified by column chromatography.

General Procedure 2: Formation of Grignard reagents

Mg turnings (88 mg, 3.6 mmol, 1.2 equiv.) were added to a flask which was then heated (heat gun) under vacuum for 2 minutes with stirring. To the cooled flask was added anhydrous THF (2.5 mL) and I_2 (1 crystal) before dropwise addition of the aryl halide (3 mmol, 1 equiv.). The mixture was then heated at reflux for 2 h (unless specified otherwise). The concentration of the resulting Grignard reagent was determined *via* iodometric titration.

General Procedure 3: Formation of Turbo Grignard reagents

Mg turnings (146 mg, 2.0 equiv., 6 mmol) and LiCl (140 mg, 1.1 equiv., 3.3 mmol) were added to a flask which was then heated (heat gun) under vacuum for 2 minutes with stirring. To the cooled flask was added anhydrous THF (3 mL) and DIBALH (0.15 mL, 1 M in hexanes or toluene, 0.05 equiv., 0.15 mmol). After 5 mins at room temperature, the halide (1 equiv., 3 mmol) was added at the specified temperature and stirred for the specified time. The concentration of the resulting Turbo Grignard reagent was determined *via* iodometric titration.

General Procedure 4: Kumada cross coupling of iodo-BCPs

To a flame-dried vial was added iodo-BCP (0.2 mmol, 1 equiv.) and $Fe(acac)_3$ (14 mg, 20 mol%, 0.04 mmol). The vial was then evacuated and refilled with $N_{2\,(g)}$ three times. To this was added THF (0.2 mL) and TMEDA (12 µL, 40 mol%, 0.08 mmol), and the resulting mixture was stirred for 5 min. The Grignard reagent (1.6 equiv., 0.32 mmol) was then added via syringe pump at a rate of 0.7 mL/h (over approximately 45 mins) at the specified temperature. The reaction was stirred for a further 1 h, then quenched by addition of aqueous HCl (5 mL, 1 M) or aqueous NH_4Cl (5 mL, saturated). The layers were separated, and the aqueous layer was extracted with Et_2O (3 × 10 mL). The combined organic layers were washed with brine, dried over $MgSO_4$ and concentrated *in vacuo*. The crude product was purified by column chromatography.

3. Optimization of Kumada Coupling

Studies began with the screening of a range of transition metal complexes in the Kumada cross-coupling reaction of iodo-BCP **1a** and PMPMgBr (3 equiv.) in THF at room temperature on a 0.1 mmol scale (Figure S1). Preliminary experiments employing rapid Grignard addition (addition over approximately 20 seconds) indicated that $Fe(acac)_3$ was the best catalyst, providing BCP **2a** in 42% yield. A range of other iron catalysts were screened, and again $Fe(acac)_3$ provided the highest yields of the desired product, out-performing complexes such as $FeCl_3$ and $FeBr_3$ (Figure S2). The addition of various nitrogen and phosphorus ligands was detrimental to reaction yield using the rapid addition protocol.

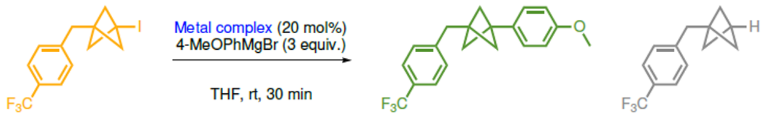

1a **2a** **3**

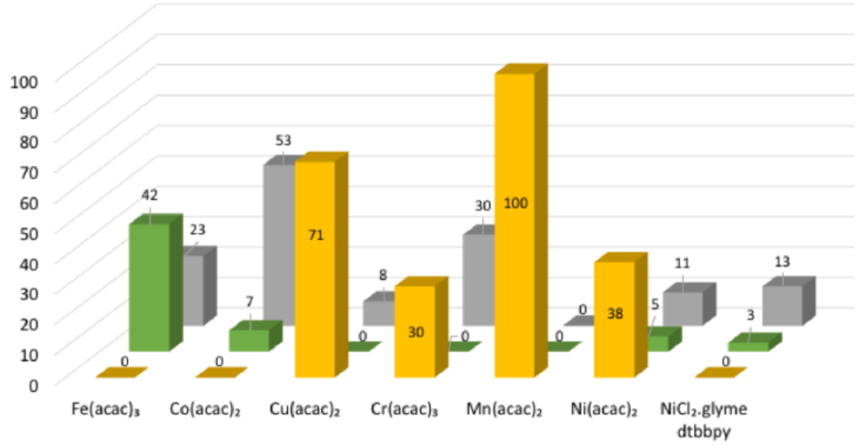

Figure S1: Preliminary screen of transition metal complexes

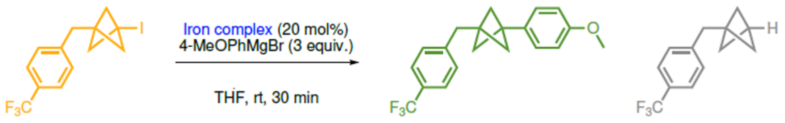

1a **2a** **3**

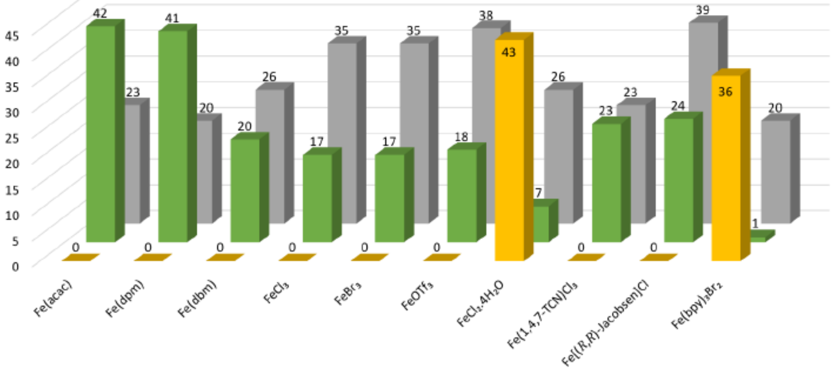

Figure S2: Screen of Fe(III) complexes

The effect of varying the rate of Grignard addition was studied. Addition of PMPMgBr over approximately 40 mins gave low yields of product in the absence of any additive / ligand, but addition of a number of amine or phosphorus-based ligands gave significantly improved yields. TMEDA providing a 66% yield of BCP **2a** (Figure S3).

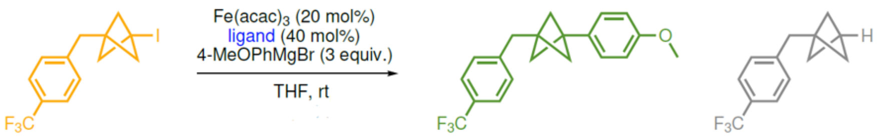

1a **2a** **3**

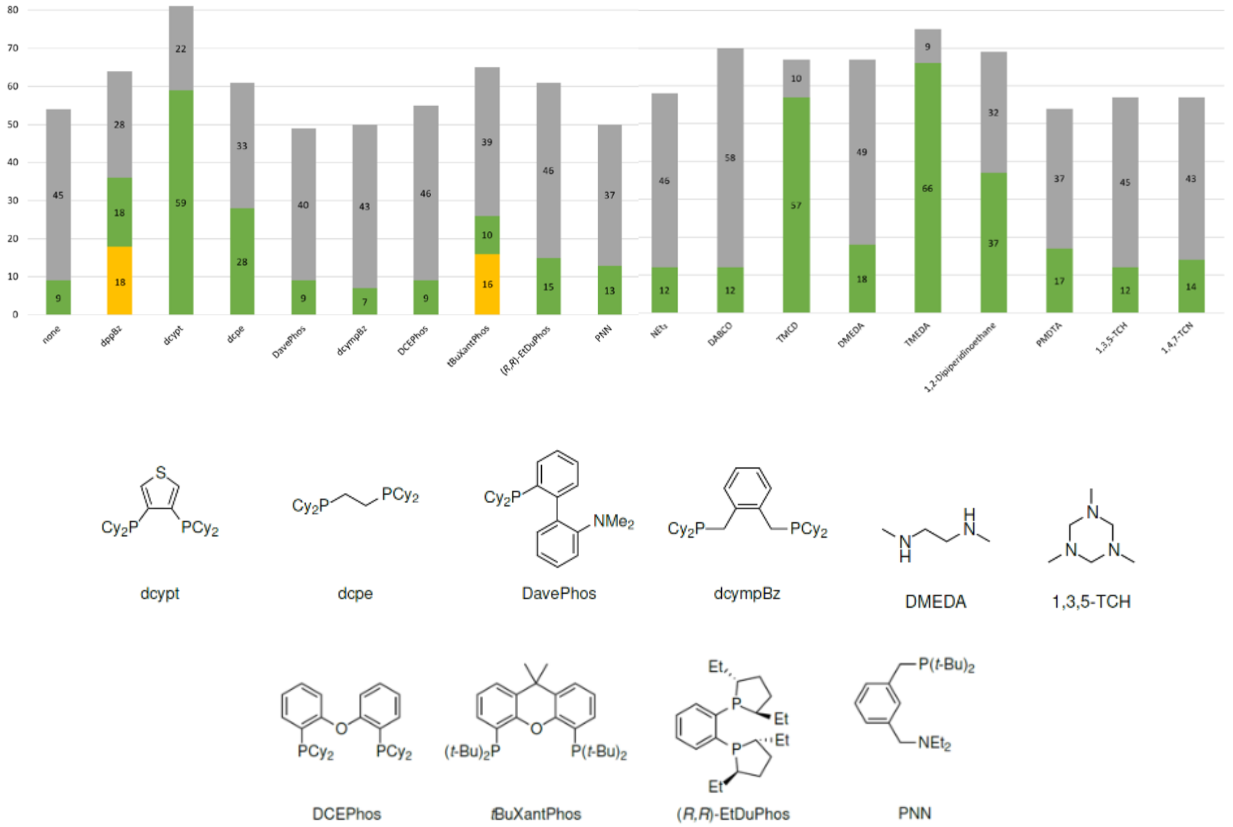

Figure S3: Screen of additives

A solvent screen identified several ethereal and aromatic solvents as suitable media (Figure S4). THF was selected for further experiments. A screen of reaction concentrations determined that higher concentration was beneficial to yields (Table S1). 1.6 equiv. of PMPMgBr proved optimal; fewer equivalents resulted in incomplete reaction, while more equivalents led to lower product yields. Further reaction optimisation is discussed in Table 1 of the manuscript.

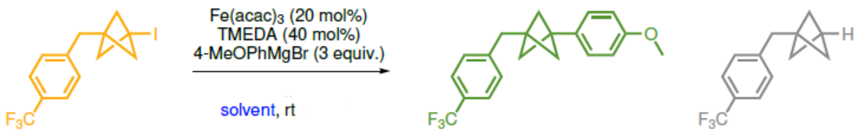

1a **2a** **3**

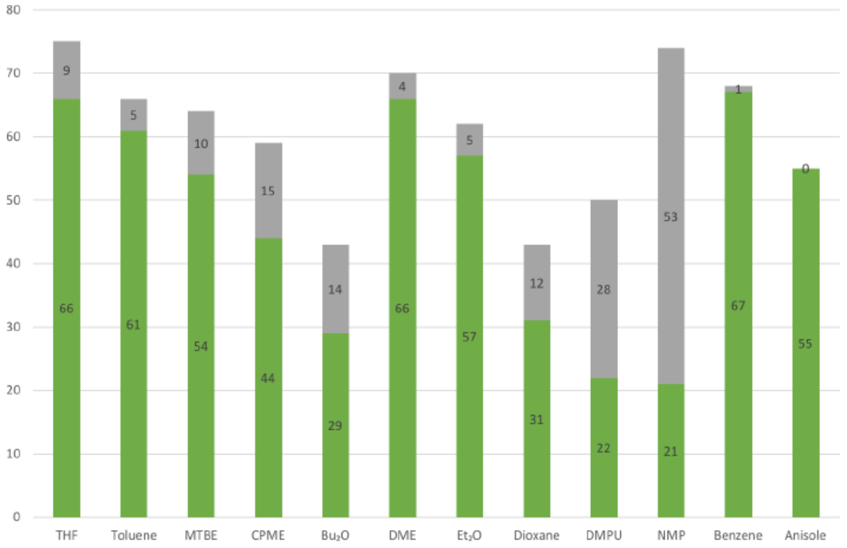

Figure S4: Screen of solvents

Entry	Concentration (M)	Yield (%)
1	0.1	60
2	0.2	64
3	0.4	66
4	0.8	73
5	1.0	73

Table S1: Screen of reaction concentration (slow addition)

4. Competition Experiments and Mechanistic Discussion

The range of arene substituents that can be accommodated in the coupling ranges from electron-rich (e.g. *p*-NMe_2) to moderately electron-poor (*p*-OCF_3). We questioned whether competition experiments might provide some insight into to the relative efficacy of cross-coupling of different aryl Grignards, and therefore into the reaction pathway. Accordingly, 4-*N*-Boc-piperidinyl iodo-BCP **1b** was subjected to coupling with an equimolar excess of PhMgBr and ArMgBr (3 equiv. each, Ar = *p/m*-substituted arene, Figure S5). These reactions were conducted under a rapid addition regime, to ensure an excess of both Grignard reagents was present throughout the reaction. Curiously, the highest rate of coupling (as reflected by product ratios) was observed with electron *neutral* substrates (H, *p*-TMS); increasing electron density on the aryl Grignard resulted in reduced reactivity. No correlation could be discerned for electron poor Grignard reagents, which likely reflects the complex interplay of factors involved in the mechanisms of Fe-catalyzed Kumada coupling reactions.

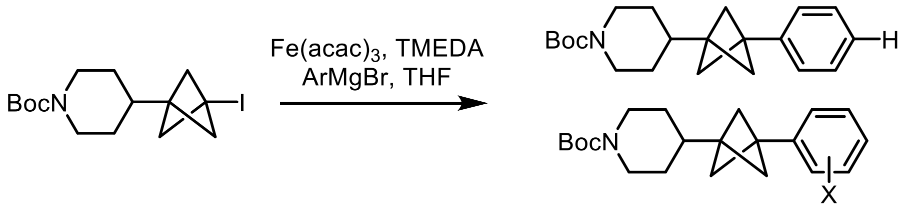

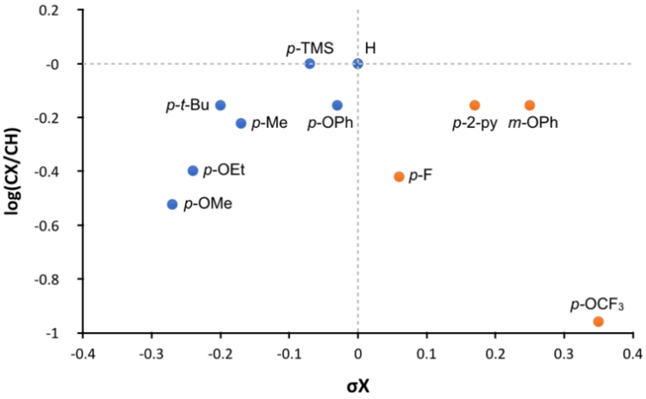

X	σX	Product Ratio (X/H)
p-OMe	-0.27	0.30
p-OEt	-0.24	0.43
p-tBu	-0.20	0.73
p-Me	-0.17	0.60
p-TMS	-0.07	1
p-OPh	-0.03	0.74
p-F	0.06	0.38
p-2-py	0.17	0.71
m-OPh	0.25	0.71
p-OCF_3	0.35	0.11

Figure S5. Substituent effects in the cross-coupling.

Procedure: To a flame dried vial under argon was added **1b** (76 mg, 0.20 mmol, 1.0 equiv.), $Fe(acac)_3$ (14 mg, 0.04 mmol, 20 mol%), TMEDA (12 µL, 0.08 mmol, 40 mol%) and anhydrous THF (0.2 mL), and the mixture was stirred for 5 minutes. To this mixture was rapidly added a 1:1 mixture of PhMgBr (0.6 mmol, 3 equiv.) and XArMgBr (0.6 mmol, 3 equiv.) as a solution in THF. The reaction mixture was stirred for 5 min then quenched by the dropwise addition of aqueous NH_4Cl (5 mL, saturated). The phases were separated, and the aqueous phase was extracted with Et_2O (3 × 10 mL). The combined organic phases were washed with brine (10 mL), dried over $MgSO_4$ and concentrated *in vacuo*. The crude mixture was

then filtered through a plug of silica, concentrated *in vacuo*, and the ratio of products determined by ^{1}H NMR spectroscopy.

Colour changes during the reaction:

In our system, the crimson solution of $Fe(acac)_3$ / TMEDA / substrate initially turns paler red / orange at the onset of ('slow') addition of aryl Grignard (Figure S6). However, after addition of ~0.6 equiv. the solution rapidly becomes dark / black, potentially due to the formation of iron nanoparticles,[4] albeit the role of these particles (if formed) is not clear.

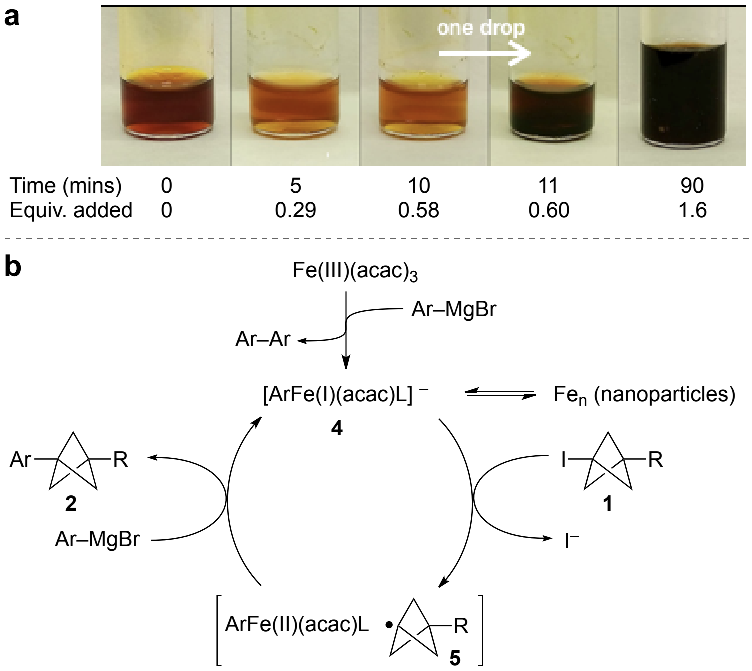

Figure S6. a. Observed colour change. **b.** Putative reaction mechanism.

5. Experimental Procedures and Characterization Data

[1.1.1]propellane (Tricyclo[1.1.1.0^{1,3}]pentane)

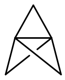

To a flame-dried round-bottom flask equipped with a stirrer bar was added 1,1-dibromo-2,2-bis(chloromethyl)cyclopropane (5.0 g, 16.9 mmol, 1.0 equiv.). The reaction vessel was evacuated and back-filled with argon three times, and then anhydrous Et_2O (10 mL) was added. The vessel was cooled to −45 °C (dry ice / isopropanol bath). Phenyllithium (17.8 mL, 1.9 M in Bu_2O, 33.7 mmol, 2.0 equiv.) was added dropwise over 15 min at –45 °C, and the resulting mixture was stirred for 15 min at −45 °C. The cooling bath was replaced with an ice bath, and the reaction mixture was warmed to 0 °C, and stirred at this temperature for 2 h. The mixture was then distilled at room temperature (50 mbar) using a rotary evaporator, the receiving flask of which was immersed in a dry ice / acetone bath. The [1.1.1]propellane-containing distillate (10 mL, [1.1.1]propellane concentration 0.91 M in Et2O, 54%) was transferred to a flame-dried septum-sealed bottle under an inert atmosphere, and stored at −20 °C. The yield was determined by ^{1}H NMR spectroscopy with 1,2-dichloroethane as an internal standard. The concentration of the [1.1.1]propellane solution ranged between 0.51 M and 1.10 M, with yields of 45-74%.

a) Synthesis of Iodo-bicyclo[1.1.1]pentanes

The following iodo-bicyclo[1.1.1]pentanes were used in this work. Iodo-BCPs **1a**, **1d**, **1e**, **1f**, **1g**, **1h** and **1j** have been reported previously by our group.[1-2] Iodo-BCPs **1b**, **1c**, **1i** and **1k** are novel; their syntheses are described below.

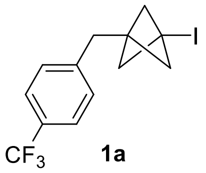

1a

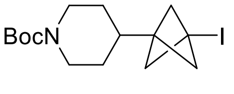

1b

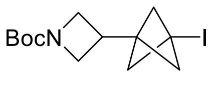

1c

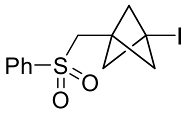

1d

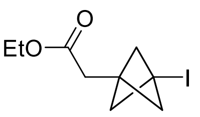

1e

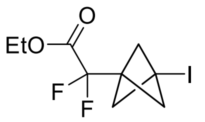

1f

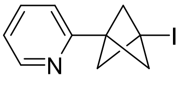

1g

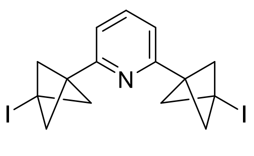

1h

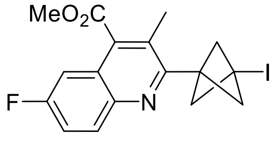

1i

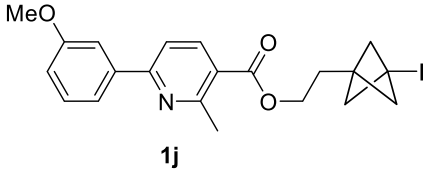

1j

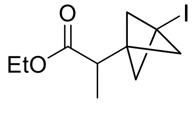

1k

***tert*-Butyl 4-(3-iodobicyclo[1.1.1]pentan-1-yl)piperidine-1-carboxylate, 1b**

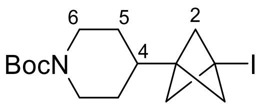

tert-Butyl 4-iodopiperidine-1-carboxylate (156 mg, 0.50 mmol, 1.0 equiv.), [1.1.1]propellane (1.7 mL, 0.54 M in Et_2O, 0.90 mmol, 1.8 equiv.) and BEt_3 (50 µL, 1.0 M in Et_2O, 0.05 mmol, 0.1 equiv.) were subjected to **General Procedure 1** at room temperature for 2 h. Purification by column chromatography (SiO_2, pentane / EtOAc, 100:0 to 95:5) afforded **1b** (155 mg, 0.31 mmol, 61%) as a white solid.

$\mathbf{R_f}$ 0.43 (pentane / EtOAc, 95:5)

m.p. 50-52 °C

^{1}H NMR (400 MHz, $CDCl_3$) δ 4.16-4.06 (2H, m, H6), 2.61 (2H, app t, J = 12.7 Hz, H6), 2.16 (6H, s, H2), 1.63-1.49 (3H, m, H4, H5), 1.44 (9H, s, *t*-Bu) 1.06 (2H, app qd, J = 12.7, 4.4 Hz, H5).

^{13}C NMR (101 MHz, $CDCl_3$) δ 154.9, 79.6, 58.6, 51.6, 43.6, 37.9, 28.8, 28.6, 7.9.

HRMS [ESI$^+$, EI$^+$, CI$^+$] Not found.

IR (film) ν_{max}/cm^{-1} 2974, 2929, 2852, 1691, 1421, 1171, 769.

***tert*-Butyl 3-(3-iodobicyclo[1.1.1]pentan-1-yl)azetidine-1-carboxylate, 1c**

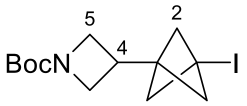

tert-Butyl 3-iodoazetidine-1-carboxylate (227 mg, 0.80 mmol, 1.0 equiv.), [1.1.1]propellane (2.2 mL, 0.54 M in Et_2O, 1.20 mmol, 1.5 equiv.) and BEt_3 (80 µL, 1.0 M in Et_2O, 0.08 mmol, 0.1 equiv.) were subjected to **General Procedure 1** at room temperature for 4 h. Purification by column chromatography (SiO_2, pentane / EtOAc, 100:0 to 95:5) afforded **1c** (185 mg, 0.53 mmol, 66%) as a white solid.

$\mathbf{R_f}$ 0.33 (pentane / Et_2O, 95:5)

m.p. 53-55 °C

^{1}H NMR (400 MHz, $CDCl_3$) δ 3.90 (2H, app t, J = 8.4, 1.8 Hz, H5), 3.61-3.52 (2H, m, H5), 2.65-2.58 (1H, m, H4), 2.24 (6H, s, H2), 1.42 (9H, s, *t*-Bu).

^{13}C NMR (101 MHz, $CDCl_3$) δ 156.5, 79.7, 58.4, 51.5, 49.1, 30.0, 28.5, 7.1.

HRMS (ESI$^+$) Found [M+H]$^+$ = 350.0611; $C_{13}H_{21}O_2NI$ requires 350.0611.

IR (film) ν_{max}/cm^{-1} 2973, 2880, 1698, 1393, 1182, 1124, 834.

Synthesis of 1i

1i was synthesized according to the following scheme.

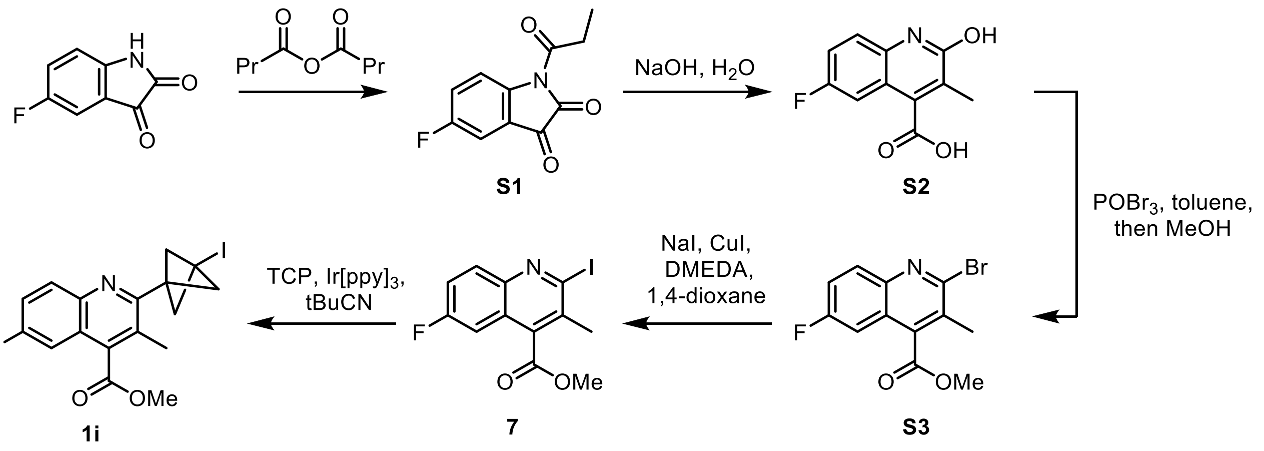

5-Fluoro-1-propionylindoline-2,3-dione, S1

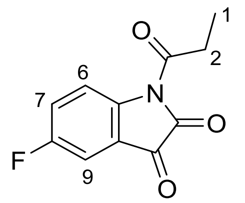

5-Fluoroisatin (26.0 g, 176 mmol, 1.0 equiv.) in propionic anhydride (113 mL, 884 mmol, 5.0 equiv.) was stirred at reflux for 3 h. The resulting solution was cooled to room temperature and filtered. The collected solid was then triturated with Et_2O (5 x 5 mL), filtered and dried to afford **S1** (24.4 g, 110 mmol, 67%) as a brown solid.

^{1}H NMR (400 MHz, DMSO-d_6) δ 8.35-8.28 (1H, m, ArH), 7.69-7.56 (2H, m, ArH), 3.01 (2H, q, J = 7.3 Hz, H2), 1.14 (3H, t, J = 7.3 Hz, H1).

^{13}C NMR (101 MHz, DMSO-d_6) δ 179.5 (d, J = 2.3 Hz), 173.5, 159.2 (d, J = 244.9 Hz), 157.9, 144.4 (d, J = 2.1 Hz), 123.8 (d, J = 23.4 Hz), 121.5 (d, J = 7.6 Hz), 119.2 (d, J = 7.4 Hz), 110.9 (d, J = 24.0 Hz), 30.9, 8.2.

^{19}F NMR (377 MHz, DMSO-d_6) δ −116.4 (m).

Spectroscopic data in agreement with that reported previously.[3]

6-Fluoro-2-hydroxy-3-methylquinoline-4-carboxylic acid, S2

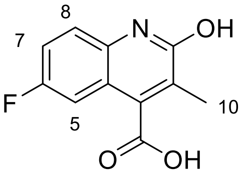

A solution of **S1** (24.1 g, 109 mmol, 1.0 equiv.) and NaOH (10.9 g, 272 mmol, 2.5 equiv.) in water (220 mL) was heated at reflux for 2 h, then cooled to room temperature. Activated charcoal (1 g) was added, and the mixture stirred for 30 minutes, then filtered through Celite. The filtrate was acidified to pH 5 with aqueous HCl (6 M), and the resulting yellow precipitate was collected by filtration, then dried to give **S2** (10.1 g, 45.7 mmol, 42%) as a pale yellow solid.

^{1}H NMR (400 MHz, DMSO-d_6) δ 11.59 (1H, s, OH), 7.31-7.21 (3H, m, ArH), 2.00 (3H, s, H10),

^{13}C NMR (101 MHz, DMSO-d_6) δ 169.4, 163.1, 156.8 (d, J = 235.9 Hz), 149.8 (d, J = 3.3 Hz), 134.6, 121.0, 118.5 (d, J = 8.8 Hz), 116.5 (d, J = 16.5 Hz), 116.3, 111.5 (d, J = 23.1 Hz), 14.0.

^{19}F NMR (377 MHz, DMSO-d_6) δ −122.3 (ddd, J = 10.6, 8.3, 5.6 Hz).

Spectroscopic data in agreement with that reported previously.[3]

Methyl 2-bromo-6-fluoro-3-methylquinoline-4-carboxylate, S3

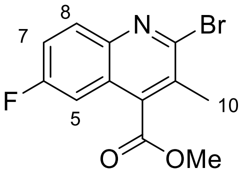

To a solution of **S2** (500 mg, 2.3 mmol, 1.0 equiv.) in anhydrous toluene (5 mL) was added $POBr_3$ (2.6 g, 9.2 mmol, 4.0 equiv.). The mixture was heated at reflux for 1.5 h (caution: HBr evolution) then cooled to room temperature. The resulting thick mixture was cooled in an ice bath, and anhydrous MeOH (5 mL) was added slowly, then maintained at this temperature for 2 h. Water (15 mL) and K_2CO_3 (5 mL, aq. sat.) were added then the aqueous layer was extracted with EtOAc (3 x 10 mL). The combined organic phases were washed with brine, dried ($MgSO_4$), filtered, and concentrated *in vacuo*. Purification by column chromatography (SiO_2, pentane / EtOAc, 9:1) afforded **S3** (470 mg, 1.6 mmol, 70%) as a white solid.

^{1}H NMR (400 MHz, DMSO-d_6) δ 8.09 (1H, dd, J = 9.3, 5.4 Hz, H8), 7.75 (1H, ddd, J = 9.3, 8.8, 2.8 Hz, H7), 7.56 (1H, dd, J = 9.7, 2.8 Hz, H5), 4.06 (3H, s, OMe), 2.45 (3H, s, H10).

^{13}C NMR (101 MHz, DMSO-d_6) δ 166.0, 160.7 (d, J = 248.2 Hz), 144.5 (d, J = 2.9 Hz), 143.2, 139.3 (d, J = 5.6 Hz), 131.3 (d, J = 9.8 Hz), 129.4, 123.8 (d, J = 10.6 Hz), 120.7 (d, J = 25.9 Hz), 108.5 (d, J = 24.0 Hz), 53.5, 19.8.

^{19}**F NMR** (377 MHz, DMSO-d_6) δ −109.8 (m).

Spectroscopic data in agreement with that reported previously.[3]

Methyl 6-fluoro-2-iodo-3-methylquinoline-4-carboxylate, 7

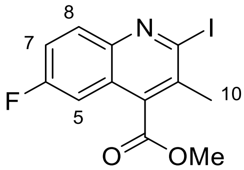

In a flame dried thick-walled tube, a solution of **S3** (310 mg, 1.0 mmol, 1.0 equiv.), NaI (320 mg, 2.0 mmol, 2.0 equiv.), CuI (9.6 mg, 0.05 mmol, 0.05 equiv.) and DMEDA (12 µl, 0.1 mmol, 0.1 equiv.) in 1,4-dioxane (3 mL) was heated at 100 °C for 24 h. After cooling to room temperature, 35% aq. NH_3 (10 mL) and water (20 mL) were added and the aqueous layer was extracted with CH_2Cl_2 (3 x 15 mL). The combined organic layers were washed with brine, dried ($MgSO_4$), and concentrated. Purification by column chromatography (SiO_2, pentane / EtOAc, 95:5) afforded **7** (264 mg, 0.76 mmol, 76%) as a pale yellow solid.

$\mathbf{R_f}$ 0.30 (pentane / EtOAc, 95:5)

m.p. 125-127 °C

^{1}**H NMR** ($CDCl_3$, 400 MHz) δ 8.03 (1H, dd, J = 9.3, 5.4 Hz, H8), 7.43 (1H, ddd, J = 9.3, 8.1, 2.7 Hz, H7), 7.28 (1H, dd, J = 9.3, 2.7 Hz, H5), 4.06 (3H, s, OMe), 2.51 (3H, s, H10).

^{13}**C NMR** ($CDCl_3$, 101 MHz) δ 166.8, 161.2 (d, J = 250.5 Hz), 144.7, 137.0 (d, J = 5.7 Hz), 132.3, 131.6 (d, J = 9.5 Hz), 126.4 (d, J = 3.2 Hz), 124.3 (d, J = 10.3 Hz), 120.1 (d, J = 25.8 Hz), 108.3 (d, J = 23.9 Hz), 53.0, 24.7.

^{19}**F NMR** (377 MHz, $CDCl_3$) δ −109.7 (m).

HRMS (ESI^+) $[M+H]^+$ = 345.9734; $C_{12}H_9O_2NFI$ requires 345.9735.

IR (film) ν_{max}/cm^{-1} 1725, 1551, 1311, 1267, 1221, 1201, 987, 831.

Methyl 6-fluoro-2-(3-iodobicyclo[1.1.1]pentan-1-yl)-3-methylquinoline-4-carboxylate, 1i

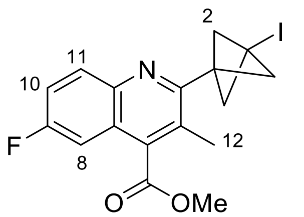

To a solution of **7** (480 mg, 1.4 mmol, 1.0 equiv.) and *fac*-Ir(ppy)$_3$ (23 mg, 35 µmol, 0.025 equiv.) in *t*-BuCN (15 ml) was added [1.1.1]propellane (3.4 mL, 0.63 M in Et_2O, 2.1 mmol, 1.5 equiv.). The solution was degassed using a modified freeze-pump-thaw method (3 ×, vacuum only applied while the solution was frozen to prevent loss of [1.1.1]propellane). The resultant mixture was then stirred under blue LED irradiation for 24 h. The reaction was then concentrated *in vacuo* and the residue purified via column chromatography (SiO_2, pentane / EtOAc, 95:5) to afford **1i** (490 mg, 1.2 mmol, 85%) as a white solid.

R$_f$ 0.24 (pentane / EtOAc, 95:5)

m.p. 144-145 °C

^{1}H NMR (400 MHz, $CDCl_3$) δ 8.02 (1H, dd, *J* = 9.2, 5.5 Hz, H11), 7.42 (1H, ddd, *J* = 9.2, 8.2, 2.8 Hz, H10), 7.26 (1H, dd, *J* = 9.5, 2.8 Hz, H8), 4.06 (3H, s, OMe), 2.85 (6H, s, H2), 2.47 (3H, s, H12).

^{13}C NMR ($CDCl_3$, 101 MHz) δ 168.0, 161.1 (d, *J* = 249.0 Hz), 155.0 (d, *J* = 2.9 Hz), 143.4, 138.6 (d, *J* = 5.6 Hz), 132.3 (d, *J* = 9.4 Hz), 127.1, 124.3 (d, *J* = 10.2 Hz), 119.5 (d, *J* = 25.7 Hz), 107.9 (d, *J* = 23.5 Hz), 62.1, 52.9, 52.8, 17.3, 8.3.

^{19}F NMR ($CDCl_3$, 377 MHz) δ −111.4 (ddd, *J* = 9.5, 8.2, 5.5 Hz).

HRMS (ESI$^+$) $[M+H]^+$ = 412.0205; $C_{17}H_{16}O_2NFI$ requires 412.0204.

IR (film) ν_{max}/cm^{-1} 2959, 1731, 1498, 1376, 1205, 1157, 1071, 847.

Ethyl 2-(3-iodobicyclo[1.1.1]pentan-1-yl)propanoate, 1k

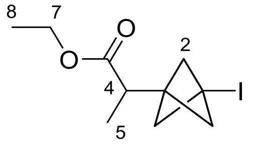

Ethyl 2-iodopropionate (0.34 mL, 2.5 mmol, 1.0 equiv.), [1.1.1]propellane (4.10 mL, 0.79 M in Et_2O, 3.3 mmol, 1.3 equiv.) and BEt_3 (0.25 mL, 1 M in hexane, 0.25 mmol, 10 mol%) were submitted to **General Procedure 1** at 0 °C for 15 min. Purification by column chromatography (SiO_2, pentane / Et_2O, 95:5) afforded **6** (593 mg, 2.0 mmol, 80%) as a colourless oil.

R$_f$ 0.12 (pentane / Et_2O, 96:4)

^{1}H NMR (400 MHz, $CDCl_3$) δ 4.20-4.01 (2H, m, H7), 2.62 (1H, q, *J* = 7.1 Hz, H4), 2.23 (6H, s, H2), 1.25 (3H, t, *J* = 7.1 Hz, H8), 1.08 (3H, d, *J* = 7.1 Hz, H5).

^{13}C NMR (101 MHz, $CDCl_3$) δ 173.2, 60.6, 59.4, 48.6, 41.6, 14.5, 13.9, 6.3.

HRMS (EI) Found $[M-OC_2H_5]^+$ = 248.9779; $C_8H_{10}IO$ requires 248.9780.

IR (film) ν_{max}/cm^{-1} 2978, 2916, 2879, 1731, 1455, 1375, 1334, 1256, 1177, 1150

.

b) Palladium-catalyzed iodo-BCP fragmentation

***tert*-Butyl 4-(3-methylenecyclobutylidene)piperidine-1-carboxylate, S5**

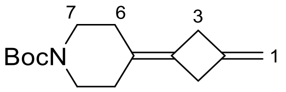

To a flame dried vial under $N_{2\ (g)}$ was added **1b** (76 mg, 1 equiv., 0.2 mmol), $Pd(PPh_3)_4$ (5.4 mg, 0.025 equiv., 0.05 mmol) and THF (2 mL), and the mixture was stirred for 18 h at 80 °C. The mixture was then concentrated *in vacuo* and purified by column chromatography (SiO_2, pentane / Et_2O, 90:10) to afford **S5** (15 mg, 30%) as a colourless oil.

R_f = 0.30 (9:1 pentane / Et_2O)

^{1}H NMR (400 MHz, $CDCl_3$) δ 4.91 (2H, quin., *J* = 2.6 Hz, H1), 3.37 (4H, t, *J* = 5.9 Hz, H7), 3.27 – 3.23 (4H, m, H3), 2.05 (4H, t, *J* = 5.9 Hz, H6), 1.46 (9H, s, *t*-Bu).

^{13}C NMR (101 MHz, $CDCl_3$) δ 155.0, 144.1, 127.0, 125.5, 106.9, 79.6, 44.7, 38.1, 29.0, 28.6.

HRMS (ESI$^+$) $[M+Na]^+$ = 272.1623; $C_{15}H_{23}O_2NNa$ requires 272.1621.

IR (film) ν_{max}/cm^{-1} 3661, 2981, 2890, 2360, 1697.

c) Kumada cross-coupling products

1-(4-Methoxyphenyl)-3-(4-(trifluoromethyl)benzyl)bicyclo[1.1.1]pentane, 2a

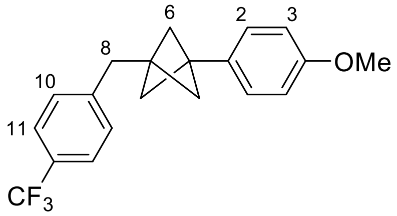

1a (70 mg, 0.20 mmol, 1.0 equiv.), Fe(acac)$_3$ (14 mg, 0.04 mmol, 20 mol%), TMEDA (12 µL, 0.08 mmol, 40 mol%) and 4-methoxyphenylmagnesium bromide (0.44 mL, 0.9 M in THF, 0.32 mmol, 1.6 equiv.) were submitted to **General Procedure 4** at room temperature. The reaction was quenched with aqueous HCl (5 mL, 1 M). Purification by column chromatography (SiO$_2$, pentane / Et$_2$O, 1:0 to 98:2) afforded **2a** (57 mg, 0.17 mmol, 86%) as a colourless oil.

R$_f$ 0.12 (pentane)

^{1}H NMR (500 MHz, CDCl$_3$) δ 7.56 (2H, d, *J* = 7.9 Hz, ArH), 7.27-7.22 (2H, d, *J* = 7.9, ArH), 7.11–7.06 (2H, m, ArH), 6.84-6.78 (2H, m, ArH), 3.78 (3H, s, OMe), 2.90 (2H, s, H8), 1.84 (6H, s, H6).

^{13}C NMR (126 MHz, CDCl$_3$) δ 158.4, 143.9, 133.5, 129.4, 128.4 (q, *J* = 32.3 Hz), 127.2, 125.3 (q, *J* = 3.8 Hz), 124.6 (q, *J* = 271.7 Hz), 113.7, 55.4, 52.3, 42.2, 39.1, 38.7.

^{19}F NMR (471 MHz, CDCl$_3$) δ –62.2.

HRMS (CI$^+$) Found [M+H]$^+$ = 333.1463; $C_{20}H_{20}OF_3$ requires 333.1461.

IR (film) ν_{max}/cm^{-1} 2965, 2867, 2871, 1615, 1517, 1317, 1244, 1161, 1115, 1062, 1040.

1-(4-(Trifluoromethoxy)phenyl)-3-(4-(trifluoromethyl)benzyl)bicyclo[1.1.1]pentane, 2b

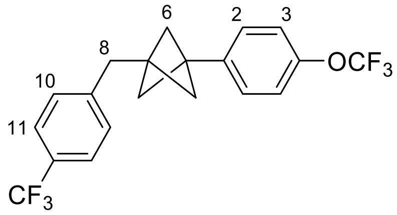

4-Trifluoromethoxyphenylmagnesium bromide: 1-Bromo-4-(trifluoromethoxy)benzene (0.45 mL, 3.0 mmol, 1.0 equiv.), and Mg turnings (88 mg, 3.6 mmol, 1.2 equiv.) were submitted to **General Procedure 2** to give 4-trifluoromethoxyphenylmagnesium bromide as a 1.0 M solution in THF.

1a (70 mg, 0.20 mmol, 1.0 equiv.), Fe(acac)$_3$ (14 mg, 0.04 mmol, 20 mol%), TMEDA (12 µL, 0.08 mmol, 40 mol%) and 4-trifluoromethoxyphenylmagnesium bromide (0.32 mL, 1.0 M in THF, 0.32 mmol, 1.6 equiv.) were submitted to **General Procedure 4** at room temperature. The reaction was quenched

with aqueous HCl (5 mL, 1 M). Purification by column chromatography (SiO_2, pentane / Et_2O, 98:2) afforded **2b** (60 mg, 0.16 mmol, 78%) as a white solid.

R$_f$ 0.32 (pentane)

m.p. 59-60 °C

^{1}H NMR (400 MHz, CD_3OD) δ 7.60-7.56 (2H, d, *J* = 8.1 Hz, H11), 7.35-7.30 (2H, d, *J* = 8.1 Hz, H10), 7.24-7.19 (2H, m, H2), 7.12 (2H, m, H3), 2.93 (2H, s, H8), 1.86 (6H, s, H6).

^{13}C NMR (101 MHz, CD_3OD) δ 149.1 (q, *J* = 1.6 Hz), 145.3, 141.6, 130.6, 129.4 (q, *J* = 32.1 Hz), 128.6, 126.2 (q, *J* = 3.6 Hz), 125.9 (q, *J* = 270.8 Hz), 121.9 (q, *J* = 253.2), 121.7, 53.0, 42.8, 39.8, 39.5.

^{19}F NMR (376 MHz, CD_3OD) δ –55.6, –59.8.

HRMS (CI^+) Found $[M+H]^+$ = 387.1175; $C_{20}H_{17}OF_6$ requires 387.1178.

IR (film) ν_{max}/cm^{-1} 2967, 2871, 1326, 1261, 1224, 1162, 1124, 1067, 1020.

***N,N*-Dimethyl-4-(3-(4-(trifluoromethyl)benzyl)bicyclo[1.1.1]pentan-1-yl)aniline, 2c**

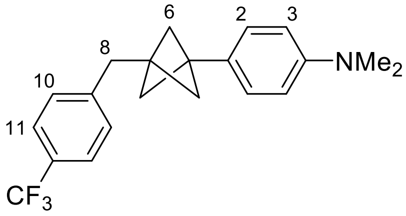

4-(*N,N*-Dimethyl)aniline magnesium bromide: 4-Bromo-*N,N*-dimethylaniline (600 mg, 3.0 mmol, 1.0 equiv.), and Mg turnings (88 mg, 3.6 mmol, 1.2 equiv.) were submitted to **General Procedure 2** to give 4-(*N,N*-dimethyl)aniline magnesium bromide as a 1.0 M solution in THF.

1a (70 mg, 0.20 mmol, 1.0 equiv.), $Fe(acac)_3$ (14 mg, 0.04 mmol, 20 mol%), TMEDA (12 µL, 0.08 mmol, 40 mol%) and 4-(*N,N*-dimethyl)aniline magnesium bromide (0.32 mL, 1.0 M in THF, 0.32 mmol, 1.6 equiv.) were submitted to **General Procedure 4** at room temperature. The reaction was quenched with aqueous HCl (5 mL, 1 M). Purification by column chromatography (SiO_2, pentane / Et_2O, 98:2) afforded **2c** (37 mg, 0.11 mmol, 53%) as a white solid.

R$_f$ 0.47 (pentane / Et_2O, 96:4)

m.p. 100-102 °C

^{1}H NMR (400 MHz, $CDCl_3$) δ 7.58 (2H, d, *J* = 8.0 Hz, H11), 7.29-7.23 (2H, d, *J* = 8.0 Hz, H10), 7.12-7.04 (2H, m, H2), 6.75-6.67 (2H, m, H3), 2.92 (6H, s, NMe_2), 2.91 (2H, s, H8), 1.85 (6H, s, H6).

^{13}C NMR (101 MHz, $CDCl_3$) δ 149.6, 144.0, 129.5, 129.4, 128.3 (q, *J* = 31.6 Hz), 126.8, 125.3 (q, *J* = 3.9 Hz), 124.6 (q, *J* = 271.6 Hz), 112.7, 52.2, 42.2, 41.0, 39.2, 38.7.

^{19}**F NMR** (376 MHz, $CDCl_3$), δ –62.2.

HRMS (ESI^+) Found $[M+H]^+$ = 346.1774; $C_{21}H_{23}NF_3$ requires 346.1777.

IR (film) ν_{max}/cm^{-1} 2969, 2906, 2869, 1614, 1525, 1323, 1157, 1114, 1066.

1-(4-Fluorophenyl)-3-(4-(trifluoromethyl)benzyl)bicyclo[1.1.1]pentane, 2d

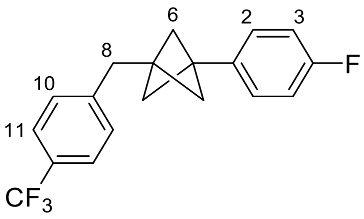

4-Fluorophenylmagnesium bromide: 4-Bromo-fluorobenzene (0.33 mL, 3.0 mmol, 1.0 equiv.) and Mg turnings (88 mg, 3.6 mmol, 1.2 equiv.) were submitted to **General Procedure 2** at room temperature for 1 h to give 4-fluorophenylmagnesium bromide as a 0.7 M solution in THF.

1a (70 mg, 0.20 mmol, 1.0 equiv.), $Fe(acac)_3$ (14 mg, 0.04 mmol, 20 mol%), TMEDA (12 µL, 0.08 mmol, 40 mol%) and 4-fluorophenylmagnesium bromide (0.45 mL, 0.7 M in THF, 0.32 mmol, 1.6 equiv.,) were submitted to **General Procedure 4** at room temperature. The reaction was quenched with aqueous HCl (5 mL, 1 M). Purification by column chromatography (SiO_2, pentane) afforded **2d** (45 mg, 0.14 mmol, 71%) as a white solid.

$\mathbf{R_f}$ 0.57 (pentane)

m.p. 58-60 °C

^{1}**H NMR** (400 MHz, $CDCl_3$) δ 7.58 (2H, d, *J* = 7.9 Hz, H11), 7.26 (2H, d, *J* = 7.9 Hz, H10), 7.16-7.09 (2H, m, H2), 7.00-6.92 (2H, m, H3), 2.92 (2H, s, H8), 1.88 (6H, s, H6).

^{13}**C NMR** (101 MHz, $CDCl_3$) δ 161.8 (d, *J* = 244.2 Hz), 143.7, 137.0 (d, *J* = 3.2 Hz), 129.4, 128.5 (q, *J* = 32.2 Hz), 127.7 (d, *J* = 8.0 Hz), 125.4 (q, *J* = 3.7 Hz), 124.6 (q, *J* = 271.3 Hz), 115.0 (d, *J* = 21.6 Hz), 52.3, 42.1, 39.0, 38.8.

^{19}**F NMR** (376 MHz, CDC13) δ –62.2, –116.6.

HRMS (CI^+) Found $[M+H]^+$ = 321.1265; $C_{19}H_{17}F_4$ requires 321.1261.

IR (film) ν_{max}/cm^{-1} 2969, 2908, 2869, 1519, 1503, 1325, 1162, 1124.

***tert*-Butyl 4-(3-(4-methoxyphenyl)bicyclo[1.1.1]pentan-1-yl)piperidine-1-carboxylate, 2e**

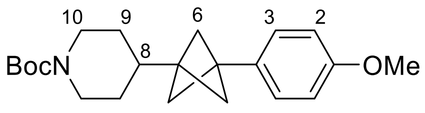

1b (83 mg, 0.22 mmol, 1.0 equiv.), Fe(acac)$_3$ (16 mg, 0.04 mmol, 20 mol%), TMEDA (13 µL, 0.09 mmol, 40 mol%) and 4-methoxyphenylmagnesium bromide (0.44 mL, 0.8 M in THF, 0.35 mmol, 1.6 equiv.) were submitted to **General Procedure 4** at room temperature. The reaction was quenched with aqueous NH_4Cl (5 mL, saturated). Purification by column chromatography (SiO_2, pentane / EtOAc, 95:5) afforded **2e** (63 mg, 0.18 mmol, 80%) as a white solid.

R$_f$ 0.43 (pentane / EtOAc, 95:5)

m.p. 103-105 °C

^{1}H NMR (400 MHz, CD_3OD) δ 7.12-7.08 (2H, m, H3), 6.83-6.79 (2H, m, H2), 4.11 (2H, m, H10), 3.75 (3H, s, OMe), 2.71 (2H, m, H10), 1.81 (6H, s, H6), 1.67-1.53 (3H, m, H8, H9), 1.46 (9H, s, *t*-Bu), 1.16-1.01 (2H, m, H9).

^{13}C NMR (101 MHz, CD_3OD) δ 159.8, 156.5, 134.9, 128.0, 114.5, 80.9, 55.6, 50.8, 44.9, 42.5, 41.5, 37.7, 29.6, 28.7.

HRMS (ESI$^+$) Found $[M+Na]^+$ = 380.2196 ; $C_{22}H_{31}O_3NNa$ requires 380.2196.

IR (film) ν_{max}/cm^{-1} 2957, 2864, 2342, 1692, 1422, 1246, 1161

***tert*-Butyl 4-(3-(4-ethoxyphenyl)bicyclo[1.1.1]pentan-1-yl)piperidine-1-carboxylate, 2f**

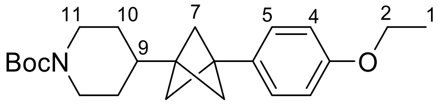

1b (76 mg, 0.20 mmol, 1.0 equiv.), Fe(acac)$_3$ (14 mg, 0.04 mmol, 20 mol%), TMEDA (12 µL, 0.08 mmol, 40 mol%) and 4-ethoxyphenylmagnesium bromide (0.32 mL, 1.0 M in THF, 0.32 mmol, 1.6 equiv.) were submitted to **General Procedure 4** at room temperature. The reaction was quenched with aqueous NH_4Cl (5 mL, saturated). Purification by column chromatography (SiO_2, pentane / EtOAc, 95:5) afforded **2f** (57 mg, 0.15 mmol, 77%) as a colourless foam.

R$_f$ 0.48 (pentane / EtOAc, 90:10)

^{1}H NMR (400 MHz, $CDCl_3$) δ 7.12 (2H, d, *J* = 8.7 Hz, ArH), 6.82 (2H, d, *J* = 8.7 Hz, ArH), 4.15 (2H, app d, *J* = 13.0 Hz, H11), 4.01 (2H, q, *J* = 7.0 Hz, H2), 2.66 (2H, td, *J* = 13.0, 2.6 Hz, H11), 1.81 (6H, s, H7), 1.66-1.48 (3H, m, H9, H10), 1.46 (9H, s, *t*Bu), 1.39 (3H, t, *J* = 7.0 Hz, H1) 1.19-1.06 (2H, m, H10).

^{13}C NMR (101 MHz, $CDCl_3$) δ 157.7, 155.0, 133.7, 127.2, 114.3, 79.4, 63.6, 50.1, 43.9, 41.7, 40.6, 36.6, 28.6, 28.6, 15.0.

HRMS (ESI^+) Found $[M+Na]^+$ = 394.2352; $C_{23}H_{33}O_3NNa$ requires 394.2352.

IR (film) ν_{max}/cm^{-1} 2960, 2857, 2332, 1691, 1425.

***tert*-Butyl 4-(3-(4-phenoxyphenyl)bicyclo[1.1.1]pentan-1-yl)piperidine-1-carboxylate, 2g**

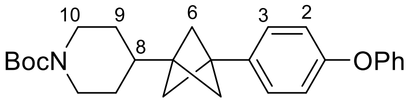

1b (76 mg, 0.20 mmol, 1.0 equiv.), $Fe(acac)_3$ (14 mg, 0.04 mmol, 20 mol%), TMEDA (12 µL, 0.08 mmol, 40 mol%) and 4-phenoxyphenylmagnesium bromide (0.36 mL, 0.9 M in THF, 0.32 mmol, 1.6 equiv.) were submitted to **General Procedure 4** at room temperature. The reaction was quenched with aqueous NH_4Cl (5 mL, saturated). Purification by column chromatography (SiO_2, pentane / EtOAc, 95:5) afforded **2g** (71 mg, 0.15 mmol, 85%) as a white solid.

R_f 0.52 (pentane / EtOAc, 90:10)

m.p. 68-70 °C

^{1}H NMR (400 MHz, $CDCl_3$) δ 7.36-7.27 (2H, m, ArH), 7.22-7.14 (2H, m, ArH), 7.12-7.03 (1H, m, ArH), 7.03-6.95 (2H, m, ArH), 6.97-6.91 (2H, m, ArH), , 4.16 (2H, app d, J = 13.1 Hz, H10), 2.67 (2H, td, J = 13.0, 2.6 Hz, H10), 1.85 (6H, s, H6), 1.70-1.51 (3H, m, H8, H9), 1.47 (9H, s, *t*Bu), 1.24-1.06 (2H, m, H9).

^{13}C NMR (101 MHz, $CDCl_3$) δ 157.7, 155.7, 155.0, 136.6, 129.8, 127.5, 123.1, 118.9, 118.7, 79.4, 50.1, 43.9, 41.8, 40.6, 36.6, 28.6, 28.6.

HRMS (ESI^+) Found $[M+Na]^+$ = 442.2353; $C_{27}H_{33}O_3NNa$ requires 442.2353.

IR (film) ν_{max}/cm^{-1} 2955, 2862, 2341, 1685, 1161.

***tert*-Butyl 4-(3-(*p*-tolyl)bicyclo[1.1.1]pentan-1-yl)piperidine-1-carboxylate, 2h**

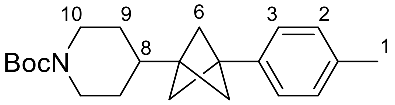

1b (76 mg, 0.20 mmol, 1.0 equiv.), $Fe(acac)_3$ (14 mg, 0.04 mmol, 20 mol%), TMEDA (12 µL, 0.08 mmol, 40 mol%) and phenylmagnesium bromide (0.54 mL, 0.6 M in THF, 0.32 mmol, 1.6 equiv.) were submitted to **General Procedure 4** at room temperature. The reaction was quenched with aqueous NH_4Cl (5 mL, saturated). Purification by column chromatography (SiO_2, pentane / Et_2O, 90:10) afforded **2h** (56 mg, 0.16 mmol, 82%) as a white solid.

R$_f$ 0.27 (pentane / Et_2O, 90:10)

m.p. 109-111 °C

^{1}H NMR (400 MHz, $CDCl_3$) δ 7.15-7.07 (4H, m, ArH), 4.15 (2H, m, H10), 2.66 (2H, app t, J = 12.7 Hz, H10), 2.32 (3H, s, H1), 1.83 (6H, s, H6), 1.64-1.49 (3H, m, H8, H9), 1.47 (9H, s, tBu), 1.13 (2H, app qd, J = 12.7, 4.7 Hz, H9).

^{13}C NMR (101 MHz, $CDCl_3$) δ 155.0, 138.6, 136.0, 128.9, 126.1, 79.4, 50.1, 43.9, 41.8, 40.8, 36.6, 28.6, 28.6, 21.2.

HRMS (ESI$^+$) Found $[M+Na]^+$ = 364.2247; $C_{22}H_{31}O_2NNa$ requires 364.2247.

IR (film) ν_{max}/cm^{-1} 2963, 2925, 2853, 1696, 1160.

***tert*-Butyl 4-(3-phenylbicyclo[1.1.1]pentan-1-yl)piperidine-1-carboxylate, 2i**

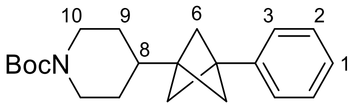

1b (76 mg, 0.20 mmol, 1.0 equiv.), $Fe(acac)_3$ (14 mg, 0.04 mmol, 20 mol%), TMEDA (12 µL, 0.08 mmol, 40 mol%) and phenylmagnesium bromide (0.32 mL, 1.0 M in THF, 0.32 mmol, 1.6 equiv.) were submitted to **General Procedure 4** at room temperature. The reaction was quenched with aqueous NH_4Cl (5 mL, saturated). Purification by column chromatography (SiO_2, pentane / Et_2O, 90:10) afforded **2i** (54 mg, 0.17 mmol, 83%) as a white solid.

R$_f$ 0.25 (pentane / Et_2O, 90:10)

m.p. 100-102 °C

^{1}H NMR (400 MHz, $CDCl_3$) δ 7.35-7.28 (2H, m, ArH), 7.26-7.20 (3H, m, ArH), 4.23-4.09 (2H, m, H10), 2.70 (2H, app t, J = 12.9 Hz, H10), 1.88 (6H, s, H6), 1.74-1.52 (3H, m, H8, H9), 1.50 (9H, s, *t*-Bu), 1.24-1.08 (2H, m, H9).

^{13}C NMR (101 MHz, $CDCl_3$) δ 155.0, 141.5, 128.2, 126.4, 126.1, 79.4, 50.0, 43.9, 41.9, 41.0, 36.6, 28.62, 28.57.

HRMS (ESI$^+$) Found $[M+H]^+$ = 328.2273; $C_{21}H_{30}O_2N$ requires 328.2271.

IR (film) ν_{max}/cm^{-1} 2964, 2865, 1691, 1423, 1235, 1161, 698.

tert-Butyl 4-(3-(4-(tert-butyl)phenyl)bicyclo[1.1.1]pentan-1-yl)piperidine-1-carboxylate, 2j

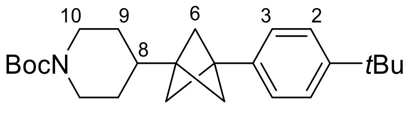

4-tert-Butylphenylmagnesium bromide: 1-Bromo-4-(*tert*-butyl)benzene (0.52 mL, 3.0 mmol, 1.0 equiv.) and Mg turnings (88 mg, 3.6 mmol, 1.2 equiv.) were submitted to **General Procedure 2** to give 4-*tert*-butylphenylmagnesium bromide as a 1.0 M solution in THF.

1b (76 mg, 0.20 mmol, 1.0 equiv.), Fe(acac)$_3$ (14 mg, 0.04 mmol, 20 mol%), TMEDA (12 µL, 0.08 mmol, 40 mol%) and 4-*tert*-butylphenylmagnesium bromide (0.32 mL, 1.0 M in THF, 0.32 mmol, 1.6 equiv.) were submitted to **General Procedure 4** at room temperature. The reaction was quenched with aqueous NH_4Cl (5 mL, saturated). Purification by column chromatography (SiO_2, pentane / Et_2O, 90:10) afforded **2j** (62 mg, 0.16 mmol, 81%) as a colourless oil.

R$_f$ 0.29 (pentane / Et_2O, 90:10)

^{1}H NMR (400 MHz, $CDCl_3$) δ 7.36-7.32 (2H, m, ArH), 7.20-7.15 (2H, m, ArH), 4.24-4.08 (2H, m, H10), 2.67 (2H, app t, J = 12.7 Hz, H10), 1.85 (6H, s, H6), 1.71-1.50 (3H, m, H8, H9), 1.47 (9H, s, *t*-Bu), 1.32 (9H, s, *t*-Bu), 1.21-1.07 (2H, m, H9).

^{13}C NMR (101 MHz, $CDCl_3$) δ 155.0, 149.3, 138.5, 125.9, 125.1, 79.3, 50.1, 43.9, 41.9, 40.7, 36.6, 34.6, 31.5, 28.62, 28.61.

HRMS (ESI$^+$) Found $[M+Na]^+$ = 406.2718; $C_{25}H_{37}O_2NNa$ requires 406.2717.

IR (film) ν_{max}/cm^{-1} 2960, 2864, 1361, 1693, 1421, 1234, 1161, 841

tert-Butyl 4-(3-(4-fluorophenyl)bicyclo[1.1.1]pentan-1-yl)piperidine-1-carboxylate, 2k

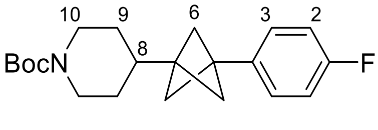

4-Fluorophenylmagnesium bromide: 4-Bromo-fluorobenzene (0.33 mL, 3.0 mmol, 1.0 equiv.) and Mg turnings (88 mg, 3.6 mmol, 1.2 equiv.) were submitted to **General Procedure 2** at room temperature for 1 h to give 4-fluorophenylmagnesium bromide as a 0.9 M solution in THF.

1b (76 mg, 0.20 mmol, 1.0 equiv.), Fe(acac)$_3$ (14 mg, 0.04 mmol, 20 mol%), TMEDA (12 µL, 0.08 mmol, 40 mol%) and 4-fluorophenylmagniesium bromide (0.38 mL, 0.9 M in THF, 0.32 mmol, 1.6 equiv.) were submitted to **General Procedure 4** at room temperature. The reaction was quenched with aqueous NH_4Cl (5 mL, saturated). Purification by column chromatography (SiO_2, pentane / Et_2O, 90:10) afforded **2k** (42 mg, 0.12 mmol, 61%) as a white solid.

R$_f$ 0.58 (pentane / Et_2O, 70:30)

m.p. 53-55 °C

^{1}H NMR (400 MHz, $CDCl_3$) δ ^{1}H NMR (400 MHz, $CDCl_3$) δ 7.19-7.11 (2H, m, H3), 7.00-6.92 (2H, m, H2), 4.23-4.08 (2H, m, H10), 2.66 (2H, app t, *J* = 12.9 Hz, H10), 1.83 (6H, s, H6), 1.68-1.49 (3H, m, H8, H9), 1.46 (9H, s, *t*-Bu), 1.18-1.05 (2H, m, H9).

^{13}C NMR (101 MHz, $CDCl_3$) δ 161.8 (d, *J* = 243.5 Hz) 155.0, 137.3 (d, *J* = 3.4 Hz), 127.7 (d, *J* = 8.1 Hz), 115.0 (d, *J* = 21.4 Hz), 79.4, 50.1, 43.9, 41.8, 40.5, 36.5, 28.6, 28.6.

^{19}F NMR (376 MHz, $CDCl_3$) δ -116.8.

HRMS : Found $[M+Na]^+$ = 368.1997; $C_{21}H_{28}O_2NF^{23}Na$ requires 368.1996

IR (film) ν_{max}/cm^{-1} 2961, 2929, 2866, 1689, 1446, 1234, 1154, 842

***tert*-Butyl 4-(3-(4-(pyridin-2-yl)phenyl)bicyclo[1.1.1]pentan-1-yl)piperidine-1-carboxylate, 2l**

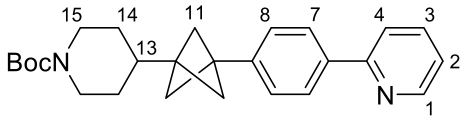

(4-(Pyridin-2-yl)phenyl)magnesium bromide: 2-(4-bromophenyl)pyridine (702 mg, 3.0 mmol, 1.0 equiv.), and Mg turnings (88 mg, 3.6 mmol, 1.2 equiv.) were submitted to **General Procedure 2** for 1 h at 45 °C to give (4-(pyridin-2-yl)phenyl)magnesium bromide as a 0.7 M solution in THF.

1b (76 mg, 0.20 mmol, 1.0 equiv.), $Fe(acac)_3$ (14 mg, 0.04 mmol, 20 mol%), TMEDA (12 μL, 0.08 mmol, 40 mol%) and (4-(pyridin-2-yl)phenyl)magnesium bromide (0.43 mL, 0.7 M in THF, 0.32 mmol, 1.6 equiv.) were submitted to **General Procedure 4** at room temperature. The reaction was quenched with aqueous NH_4Cl (5 mL, saturated). Purification by column chromatography (SiO_2, pentane / Et_2O, 70:30) afforded **2l** (66 mg, 0.16 mmol, 81%) as a white solid.

R$_f$ = 0.18 (pentane / Et_2O, 70:30)

m.p. = 152-154 °C

^{1}H NMR (400 MHz, $CDCl_3$) δ 8.71-8.63 (1H, m, H1), 7.96-7.90 (2H, m, H7), 7.75-7.67 (2H, m, ArH), 7.34-7.28 (2H, m, ArH), 7.19 (1H, ddd, *J* = 6.7, 4.8, 2.0 Hz, ArH), 4.27-4.05 (2H, m, H15), 2.74-2.60 (2H, m, H15), 1.89 (6H, s, H11), 1.58 (3H, m, H13, H14), 1.47 (9H, s, *t*-Bu), 1.21-1.07 (2H, m, H14).

^{13}C NMR (101 MHz, $CDCl_3$) δ 157.5, 155.0, 149.7, 142.3, 137.6, 136.8, 126.8, 126.6, 122.0, 120.4, 79.3, 50.1, 43.9, 42.0, 40.9, 36.6, 29.8, 28.6.

HRMS: Found $[M+H]^+$ = 405.2543; $C_{26}H_{33}O_2N_2$ requires 405.2537

IR (film) ν_{max}/cm^{-1} 2962, 2928, 2866, 1680, 1425, 1236, 1162, 777.

***tert*-butyl 4-(3-(4-(trimethylsilyl)phenyl)bicyclo[1.1.1]pentan-1-yl)piperidine-1-carboxylate, 2m**

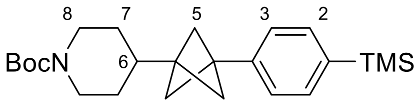

(4-(Trimethylsilyl)phenyl)magnesium bromide: (4-Bromophenyl)trimethylsilane (586 µL, 3.0 mmol, 1.0 equiv.) and Mg turnings (88 mg, 3.6 mmol, 1.2 equiv.) were submitted to **General Procedure 2** to give (4-(trimethylsilyl)phenyl)magnesium bromide as a 0.8 M solution in THF.

1b (76 mg, 0.20 mmol, 1.0 equiv.), Fe(acac)$_3$ (14 mg, 0.04 mmol, 20 mol%), TMEDA (12 µL, 0.08 mmol, 40 mol%) and (4-(trimethylsilyl)phenyl)magnesium bromide (0.4 mL, 0.8 M in THF, 0.32 mmol, 1.6 equiv.) were submitted to **General Procedure 4** at room temperature. The reaction was quenched with aqueous NH_4Cl (5 mL, saturated). Purification by column chromatography (SiO_2, pentane / Et_2O, 90:10) afforded **2m** (65 mg, 0.16 mmol, 81%) as a clear foam.

Gram scale:

To a vial was added **1b** (1.00 g, 2.70 mmol, 1.0 equiv.), and Fe(acac)$_3$ (186 mg, 0.53 mmol, 20 mol%). The vial was then evacuated and refilled with $N_{2\,(g)}$ three times. To this was added TMEDA (0.16 mL, 1.10 mmol, 40 mol%) and THF (2.7 mL), the resultant mixture was then stirred for 5 minutes. (4-(Trimethylsilyl)phenyl)magnesium bromide (4.2 mL, 1.0 M in THF, 4.2 mmol, 1.6 equiv.) was then added via syringe pump at a rate of 5.6 mL/h (over approximately 45 min) at room temperature. The reaction was stirred for a further 1 hour then quenched with aqueous NH_4Cl (20 mL, saturated). The layers were separated, and the aqueous layer was extracted with Et_2O (3 × 50 mL). The combined organic layers were washed with brine, dried over $MgSO_4$ and concentrated *in vacuo*. Purification by column chromatography (SiO_2, pentane / Et_2O, 90:10) afforded **2m** (0.96 g, 2.4 mmol, 90%) as a clear foam.

R_f 0.36 (pentane / Et_2O, 90:10)

^{1}H NMR (400 MHz, $CDCl_3$) δ 7.47 (2H, d, J = 7.0 Hz, ArH), 7.22 (2H, d, J = 7.0 Hz, ArH), 4.21-4.07 (2H, m, H8), 2.67 (2H, app t, J = 13.0 Hz, H8), 1.85 (6H, s, H5), 1.65-1.50 (3H, m, H6, H7), 1.47 (9H, s, *t*-Bu), 1.13 (2H, app qd, J = 12.5, 4.5 Hz, H7), 0.25 (9H, s, TMS).

^{13}C NMR (101 MHz, $CDCl_3$) δ 155.0, 142.0, 138.2, 133.3, 125.6, 79.4, 50.0, 43.9, 42.0, 41.0, 36.6, 28.6, 28.6, -1.0.

HRMS (ESI$^+$) Found $[M+Na]^+$ = 422.2487; $C_{24}H_{37}O_2NNaSi$ requires 422.2486.

IR (film) ν_{max}/cm^{-1} 2956, 2866, 1696, 1422, 841.

1-(2-Methoxyphenyl)-3-(4-(trifluoromethyl)benzyl)bicyclo[1.1.1]pentane, 2n

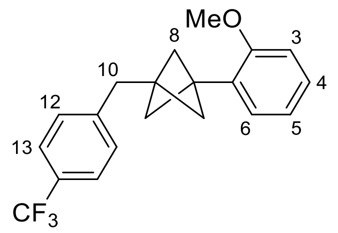

2-Methoxyphenylmagnesium bromide: 2-Bromoanisole (0.37 mL, 3 mmol, 1.0 equiv.), and Mg turnings (88 mg, 3.6 mmol, 1.2 equiv.) were submitted to **General Procedure 2** to give 2-methoxyphenylmagnesium bromide as a 0.7 M solution in THF.

1a (70 mg, 0.20 mmol, 1.0 equiv.), $Fe(acac)_3$ (14 mg, 0.04 mmol, 20 mol%), TMEDA (12 mL, 0.08 mmol, 40 mol%) and 2-methoxyphenylmagnesium bromide (0.45 mL, 0.7 M in THF, 0.32 mmol, 1.6 equiv.) were submitted to **General Procedure 4** at 45 °C. The reaction was quenched with aqueous HCl (5 mL, 1 M). Purification by column chromatography (SiO_2, pentane) afforded **2n** (49 mg, 0.15 mmol, 74%) as a white solid.

R_f = 0.21 (pentane / Et_2O, 98:2)

m.p. 81-83 °C

^{1}H NMR (400 MHz, $CDCl_3$) δ 7.62-7.57 (2H, m, H13), 7.31-7.26 (2H, m, H12), 7.21 (1H, ddd, *J* = 8.2, 7.4, 1.8 Hz, ArH), 7.06 (1H, dd, *J* = 7.4, 1.8 Hz, ArH), 6.89 (1H, app td, *J* = 7.4, 1.1 Hz, ArH), 6.83 (1H, dd, *J* = 8.3, 1.1 Hz, ArH), 3.81 (3H, s, OMe), 2.93 (2H, s, H10), 1.97 (6H, s, H8).

^{13}C NMR (101 MHz, $CDCl_3$) δ 158.9, 144.1, 129.4, 128.8, 128.4, 128.2 (q, *J* = 32.2 Hz), 128.0, 125.3 (q, *J* = 3.7 Hz), 123.3 (q, *J* = 271.9 Hz), 120.2, 110.5, 55.2, 52.2, 41.1, 40.3, 39.2.

^{19}F NMR (376 MHz, $CDCl_3$) δ –62.2.

HRMS (CI^+) Found $[M+H]^+$ = 333.1463; $C_{20}H_{20}OF_3$ requires 333.1461.

IR (film) ν_{max}/cm^{-1} 2970, 2870, 1492, 1324, 1162, 1116, 752.

***tert*-Butyl 4-(3-(o-tolyl)bicyclo[1.1.1]pentan-1-yl)piperidine-1-carboxylate, 2o**

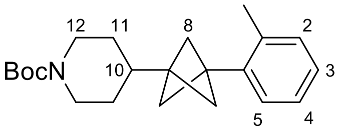

***o*-Tolylmagnesium bromide:** 2-Methylbromobenzene (360 μL, 3.0 mmol, 1.0 equiv.) and Mg turnings (88 mg, 3.6 mmol, 1.2 equiv.) were submitted to **General Procedure 2** to give *o*-tolylmagnesium bromide as a 0.8 M solution in THF.

1b (76 mg, 0.20 mmol, 1.0 equiv.), $Fe(acac)_3$ (14 mg, 0.04 mmol, 20 mol%), TMEDA (12 µL, 0.08 mmol, 40 mol%) and *o*-tolylmagnesium bromide (0.40 mL, 0.8 M in THF, 0.32 mmol, 1.6 equiv.) were submitted to **General Procedure 4** at 45 °C. The reaction was quenched with aqueous NH_4Cl (5 mL, saturated). Purification by column chromatography (SiO_2, pentane / Et_2O, 95:5) afforded **2o** (59 mg, 0.17 mmol, 86%) as a clear oil.

R$_f$ 0.28 (pentane / Et_2O, 90:10)

^{1}H NMR (400 MHz, $CDCl_3$) δ 7.15-7.05 (4H, m, ArH), 4.24-4.01 (2H, m, H12), 2.67 (2H, app t, *J* = 13.2 Hz, H12), 2.39 (3H, s, H13), 1.96 (6H, s, H8), 1.68-1.50 (3H, m, H10, H11), 1.47 (9H, s, *t*-Bu), 1.24-1.05 (2H, m, H11).

^{13}C NMR (101 MHz, $CDCl_3$) δ 155.0, 138.7, 137.0, 130.6, 127.9, 126.9, 125.8, 79.4, 50.1, 43.9, 43.1, 42.1, 36.6, 28.6, 28.6, 20.8.

HRMS (ESI$^+$) Found $[M+Na]^+$ = 364.2249; $C_{22}H_{31}O_2NNa$ requires 364.2247.

IR (film) ν_{max}/cm^{-1} 2981, 1692, 1612, 1392, 1155.

1-(3-Methoxyphenyl)-3-(4-(trifluoromethyl)benzyl)bicyclo[1.1.1]pentane, 2p

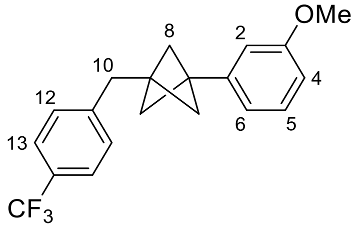

1a (70 mg, 0.20 mmol, 1.0 equiv.), $Fe(acac)_3$ (14 mg, 0.04 mmol, 20 mol%), TMEDA (12 µL, 0.08 mmol, 40 mol%) and 3-methoxyphenylmagnesium bromide (0.32 mL, 1 M in THF, 0.32 mmol, 1.6 equiv.) were submitted to **General Procedure 4** at room temperature. The reaction was quenched with aqueous HCl (5 mL, 1 M). Purification by column chromatography (SiO_2, pentane / Et_2O, 1:0 to 98:2) afforded **2p** (48 mg, 0.15 mmol, 73%) as a colourless oil.

R$_f$ 0.14 (pentane / Et_2O, 98:2).

^{1}H NMR (400 MHz, $CDCl_3$) δ 7.58 (2H, d, *J* = 8.5 Hz, H13), 7.27 (2H, d, *J* = 8.5 Hz, H12), 7.21 (1H, app t, *J* = 7.8 Hz, ArH), 6.80-6.73 (3H, m, ArH), 3.80 (3H, s, OMe), 2.93 (2H, s, H10), 1.89 (6H, s, H8).

^{13}C NMR (101 MHz, $CDCl_3$) δ 159.7, 143.8, 142.8, 129.4, 129.3, 128.4 (q, *J* = 32.9 Hz), 125.3 (q, *J* = 3.7 Hz), 122.6 (q, *J* = 272.3 Hz), 118.5, 111.9, 111.8, 55.3, 52.2, 42.6, 39.0, 38.8.

^{19}F NMR (376 MHz, $CDCl_3$) δ –62.2.

HRMS (CI$^+$) Found $[M+H]^+$ = 333.1462; $C_{20}H_{20}OF_3$ requires 333.1461.

IR (film) ν_{max}/cm^{-1} 2962, 1603, 1582, 1434, 1322, 1160, 1066, 696.

***tert*-Butyl 4-(3-(3-phenoxyphenyl)bicyclo[1.1.1]pentan-1-yl)piperidine-1-carboxylate, 2q**

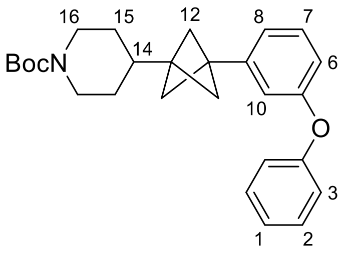

(3-Phenoxyphenyl)magnesium bromide: 1-bromo-3-phenoxybenzene (0.55 mL, 3.0 mmol, 1.0 equiv.), and Mg turnings (88 mg, 3.6 mmol, 1.2 equiv.) were submitted to **General Procedure 2** to give (3-phenoxyphenyl)magnesium bromide as a 1.1 M solution in THF.

1b (76 mg, 0.20 mmol, 1.0 equiv.), $Fe(acac)_3$ (14 mg, 0.04 mmol, 20 mol%), TMEDA (12 μL, 0.08 mmol, 40 mol%) and (3-phenoxyphenyl)magnesium bromide (0.31 mL, 1.1 M in THF, 0.32 mmol, 1.6 equiv.) were submitted to **General Procedure 4** at room temperature. The reaction was quenched with aqueous NH_4Cl (5 mL, saturated). Purification by column chromatography (SiO_2, pentane / Et_2O, 90:10) afforded **2q** (66 mg, 0.16 mmol, 78%) as a white solid.

$\mathbf{R_f}$ 0.24 (pentane / Et_2O, 90:10)

m.p. 60-62 °C

^{1}H NMR (400 MHz, $CDCl_3$) δ 7.36-7.31 (2H, m, ArH), 7.27-7.22 (1H, m, ArH), 7.13-7.07 (1H, m, ArH), 7.04-6.99 (2H, m, ArH), 6.96 (1H, ddd, *J* = 7.6, 1.6, 1.0 Hz, ArH), 6.90 (1H, dd, *J* = 2.5, 1.5 Hz, ArH), 6.82 (1H, ddd, *J* = 8.1, 2.5, 1.0 Hz, ArH), 4.16 (2H, app s, H16), 2.73-2.61 (2H, m, H16), 1.84 (6H, s, H12), 1.65-1.56 (2H, m, H15), 1.53 (1H, dt, *J* = 11.7, 3.6 Hz, H14), 1.47 (9H, s, *t*-Bu), 1.19-1.06 (2H, m, H15).

^{13}C NMR (101 MHz, $CDCl_3$) δ 157.4, 157.2, 155.0, 143.7, 129.8, 129.5, 123.2, 121.1, 118.8, 116.9, 116.8, 79.4, 50.1, 43.9, 41.9, 40.8, 36.5, 28.6, 28.5.

HRMS (ESI$^+$) Found $[M+Na]^+$ = 442.2350; $C_{27}H_{33}O_3NNa$ requires 442.2350.

IR (film) ν_{max}/cm^{-1} 2962, 2929, 2865, 1691, 1489, 1424, 1233, 1151.

tert-Butyl 4-(3-(3-(1,3-dioxolan-2-yl)phenyl)bicyclo[1.1.1]pentan-1-yl)piperidine-1-carboxylate, 2r

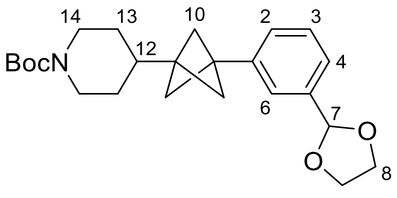

(3-(1,3-Dioxolan-2-yl)phenyl)magnesium bromide: 2-(3-Bromophenyl)-1,3-dioxolane (0.46 mL, 3.0 mmol, 1.0 equiv.), and Mg turnings (88 mg, 3.6 mmol, 1.2 equiv.) were submitted to **General Procedure 2** to give (3-(1,3-dioxolan-2-yl)phenyl)magnesium bromide as a 0.6 M solution in THF.

1b (76 mg, 0.20 mmol, 1.0 equiv.), Fe(acac)$_3$ (14 mg, 0.04 mmol, 20 mol%), TMEDA (12 µL, 0.08 mmol, 40 mol%) and (3-(1,3-dioxolan-2-yl)phenyl)magnesium bromide (0.53 mL, 0.6 M in THF, 0.32 mmol, 1.6 equiv.) were submitted to **General Procedure 4** at room temperature. The reaction was quenched with aqueous NH_4Cl (5 mL, saturated). Purification by column chromatography (SiO_2, pentane / Et_2O, 60:40) afforded **2r** (44 mg, 0.11 mmol, 55%) as a colourless oil.

R$_f$ 0.24 (pentane / Et_2O, 70:30)

^{1}H NMR (400 MHz, $CDCl_3$) δ 7.34-7.27 (3H, m, ArH), 7.24-7.19 (1H, m, ArH), 5.79 (1H, s, H7), 4.29-3.84 (6H, m, H8, H14), 2.66 (2H, app t, J = 12.8 Hz, H14), 1.85 (6H, s, H14), 1.56 (3H, m, H12, H13), 1.46 (9H, s, *t*-Bu), 1.12 (2H, app qd, J = 12.6, 4.4 Hz, H13).

^{13}C NMR (101 MHz, $CDCl_3$) δ 155.0, 141.7, 137.8, 128.3, 127.1, 124.5, 124.1, 103.9, 79.4, 65.4, 50.1, 44.0, 41.9, 41.0, 36.6, 28.6, 28.6.

HRMS (ESI$^+$) Found $[M+Na]^+$ = 422.2300; $C_{24}H_{33}O_4NNa$ requires 422.2302.

IR (film) ν_{max}/cm^{-1} 2963, 2929, 2865, 1688, 1445, 1235, 1156, 1097.

1-(5-Fluoro-2-methoxyphenyl)-3-(4-(trifluoromethyl)benzyl)bicyclo[1.1.1]pentane, 2s

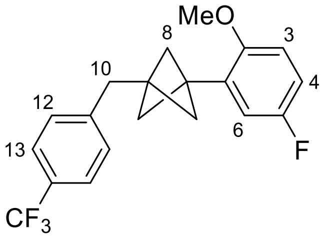

2-Methoxy-5-fluorophenylmagnesium bromide: 2-Bromo-4-fluoro-1-methoxybenzene (0.33 mL, 3.0 mmol, 1.0 equiv.) and Mg turnings (88 mg, 3.6 mmol, 1.2 equiv.) were submitted to **General Procedure 2** to give 2-methoxy-5-fluorophenylmagnesium bromide as a 1.0 M solution in THF.

1a (70 mg, 0.20 mmol, 1.0 equiv.), Fe(acac)$_3$ (14 mg, 0.04 mmol, 20 mol%), TMEDA (12 µL, 0.08 mmol, 40 mol%) and 2-methoxy-5-fluorophenylmagnesium bromide (0.32 mL, 1.0 M in THF, 0.32 mmol, 1.6

equiv.) were submitted to **General Procedure 4** at 45 °C. The reaction was quenched with aqueous HCl (5 mL, 1 M). Purification by column chromatography (SiO_2, pentane / Et_2O, 98:2) afforded **2s** (50 mg, 0.143 mmol, 72%) as a white solid.

R$_f$ 0.45 (pentane / Et_2O, 98:2)

m.p. 86-88 °C

^{1}H NMR (400 MHz, $CDCl_3$) δ 7.59-7.54 (2H, d, J = 7.9 Hz, H13), 7.27-7.23 (2H, d, J = 7.9 Hz, H12), 6.84 (1H, ddd, J = 8.8, 8.0, 3.2 Hz, ArH), 6.75-6.68 (2H, m, ArH), 3.75 (3H, s, OMe), 2.90 (2H, s, H10), 1.93 (6H, s, H8).

^{13}C NMR (101 MHz, $CDCl_3$) δ 157.0 (d, J = 238.6 Hz), 155.0 (d, J = 2.17), 143.9, 130.7 (d, J = 6.6 Hz), 129.4, 128.4 (q, J = 32.1 Hz), 125.3 (q, J = 3.8 Hz), 124.6 (q, J = 271.3 Hz), 115.3 (d, J = 22.2 Hz), 113.5 (d, J = 22.2 Hz), 111.4 (d, J = 6.7 Hz), 55.9, 52.2, 40.8, 40.3, 39.1.

^{19}F NMR (376 MHz, $CDCl_3$) δ −62.2, −124.8.

HRMS [ESI$^+$, EI$^+$, CI$^+$] Not found.

IR (film) ν_{max}/cm^{-1} 2966, 2906, 2866, 1615, 1330, 1110

5-(3-(4-(Trifluoromethyl)benzyl)bicyclo[1.1.1]pentan-1-yl)-2,3-dihydrobenzofuran, 2t

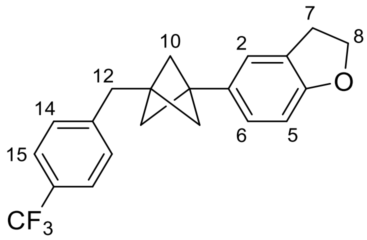

(2,3-Dihydrobenzofuran-5-yl)magnesium bromide: 5-Bromo-2,3-dihydrobenzofuran (597 mg, 3.0 mmol, 1.0 equiv.) and Mg turnings (88 mg, 3.6 mmol, 1.2 equiv.) were submitted to **General Procedure 2** to give (2,3-dihydrobenzofuran-5-yl)magnesium bromide as a 0.9 M solution in THF.

1a (70 mg, 0.20 mmol, 1.0 equiv.), $Fe(acac)_3$ (14 mg, 0.04 mmol, 20 mol%), TMEDA (12 µL, 0.08 mmol, 40 mol%) and (2,3-dihydrobenzofuran-5-yl)magnesium bromide (0.35 mL, 0.9 M in THF, 0.32 mmol, 1.6 equiv.) were submitted to **General Procedure 4** at room temperature. The reaction was quenched with aqueous HCl (5 mL, 1 M). Purification by column chromatography (SiO_2, pentane / Et_2O, 98:2) afforded **2t** (48 mg, 0.14 mmol, 70%) as a white solid.

R$_f$ 0.42 (pentane / Et_2O, 98:2)

m.p. 65-67 °C

^{1}H NMR (400 MHz, $CDCl_3$) δ 7.56 (2H, d, *J* = 8.0 Hz, H15), 7.25 (2H, d, *J* = 8.0 Hz, H14), 7.02 (1H, d, *J* = 1.4 Hz, H2), 6.92-6.89 (1H, m, H6), 6.69 (1H, d, *J* = 8.1 Hz, H5), 4.53 (2H, t, *J* = 8.7 Hz, H8), 3.16 (2H, t, *J* = 8.7 Hz, H7), 2.90 (2H, s, H12), 1.84 (6H, s, H10).

^{13}C NMR (101 MHz, $CDCl_3$) δ 158.9, 143.9, 133.5, 129.4, 128.4 (q, *J* = 32.6 Hz), 127.0, 125.7, 125.3 (q, *J* = 3.8 Hz), 124.6 (q, *J* = 270.3), 122.8, 108.9, 71.3, 52.3, 42.4, 39.1, 38.6, 29.8.

^{19}F NMR (376 MHz, $CDCl_3$) δ –62.2.

HRMS (ESI^+) Found $[M+H]^+$ = 345.1461; $C_{21}H_{20}OF_3$ requires 345.1461.

IR (film) ν_{max}/cm^{-1} 2962, 2905, 2866, 1492, 1323, 1162, 1117, 822.

5-(3-(4-(Trifluoromethyl)benzyl)bicyclo[1.1.1]pentan-1-yl)benzo[d][1,3]dioxole, 2u

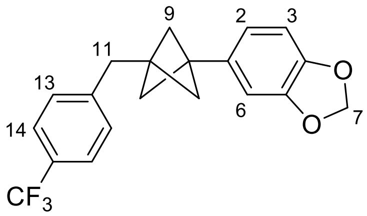

Benzo[*d*][1,3]dioxol-5-ylmagnesium bromide: 5-Bromo-1,3-benzodioxole (0.36 mL, 3.0 mmol, 1.0 equiv.) and Mg turnings (88 mg, 3.6 mmol, 1.2 equiv.) were submitted to **General Procedure 2** to give benzo[*d*][1,3]dioxol-5-ylmagnesium bromide as a 1.1 M solution in THF.

1a (70 mg, 0.20 mmol, 1.0 equiv.), $Fe(acac)_3$ (14 mg, 0.04 mmol, 20 mol%), TMEDA (12 µL, 0.08 mmol, 40 mol%) and benzo[*d*][1,3]dioxol-5-ylmagnesium bromide (0.30 mL, 1.1 M in THF, 0.32 mmol, 1.6 equiv.) were submitted to **General Procedure 4** at room temperature. The reaction was quenched with aqueous HCl (5 mL, 1 M). Purification by column chromatography (SiO_2, pentane / Et_2O, 98:2) afforded **2u** (50 mg, 0.14 mmol, 72%) as a white solid.

R_f 0.22 (pentane)

m.p. 64-66 °C

^{1}H NMR (400 MHz, $CDCl_3$) δ 7.57 (2H, d, *J* = 8.1 Hz, H14), 7.25 (2H, d, *J* = 8.1 Hz, H13), 6.72 (1H, d, *J* = 7.9 Hz, H3), 6.66 (1H, d, *J* = 1.6 Hz, H6), 6.61 (1H, dd, *J* = 7.9, 1.6 Hz, H2), 5.90 (2H, s, H7), 2.90 (2H, s, H11), 1.83 (6H, s, H9).

^{13}C NMR (101 MHz, $CDCl_3$) δ 147.6, 146.3, 143.8, 135.3, 129.3, 128.4 (q, *J* = 32.6 Hz), 125.3 (q, *J* = 3.8 Hz), 124.6 (q, *J* = 271.1 Hz), 119.1, 108.1, 106.8, 101.0, 52.3, 42.5, 39.0, 38.6.

^{19}F NMR (376 MHz, $CDCl_3$) δ –62.2.

HRMS (ESI) Found $[M+H]^+$ = 347.1255; $C_{20}H_{18}O_2F_3$ requires 347.1253.

IR (film) ν_{max}/cm^{-1} 2964, 2906, 1489, 1439, 1323, 1117

***tert*-Butyl 4-(3-(pyridin-3-yl)bicyclo[1.1.1]pentan-1-yl)piperidine-1-carboxylate, 2v**

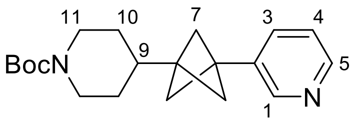

Pyridin-3-ylmagnesium bromide lithium chloride complex: 3-Bromopyridine (0.48 mL, 5.0 mmol, 1.0 equiv.), Mg turnings (365 mg, 15.0 mmol, 3.0 equiv.) and LiCl (318 mg, 7.5 mmol, 1.5 equiv.) were submitted to **General Procedure 3** at 0 °C for 2 h to give pyridin-3-ylmagnesium bromide lithium chloride complex as a 0.6 M solution in THF.

To a vial was added **1b** (76 mg, 0.2 mmol, 1.0 equiv.) and $Fe(acac)_3$ (14 mg, 20 mol%, 0.04 mmol). The vial was then evacuated and refilled with $N_{2\,(g)}$ three times. To this was added TMEDA (12 µL, 40 mol%, 0.08 mmol) and THF (0.2 mL), the resultant mixture was then stirred for 5 minutes at 65 °C. Pyridin-3-ylmagnesium bromide lithium chloride complex (1.10 mL, 0.6 M in THF, 0.60 mmol, 3.0 equiv.) was added and resulting mixture was stirred for 1 hour then quenched with aqueous NH_4Cl (5 mL, saturated). The layers were separated, and the aqueous layer was extracted with EtOAc (3 × 10 mL). The combined organic layers were washed with brine, dried over $MgSO_4$ and concentrated *in vacuo*. Purification by column chromatography (SiO_2, pentane / Et_2O, 75:25 to 50:50) afforded **2v** (17 mg, 0.05 mmol, 27%) as a yellow solid.

$\mathbf{R_f}$ 0.45 (pentane / EtOAc, 50:50)

m.p. 58-60 °C

^{1}H NMR (400 MHz, $CDCl_3$) δ 8.45 (2H, m, H1 + H5), 7.49 (1H, ddd, *J* = 7.7, 2.2, 1.7 Hz, H3), 7.19 (1H, ddd, *J* = 7.8, 4.8, 0.9 Hz, H4), 4.15 (2H, br app s, H11), 2.66 (2H, app t, *J* = 12.8 Hz, H11), 1.90 (6H, s, H7), 1.65-1.51 (3H, m, H10 + H9), 1.46 (9H, s, *t*-Bu), 1.23-1.05 (2H, m, H10).

^{13}C NMR (101 MHz, $CDCl_3$) δ 155.0, 148.1, 147.8, 136.5, 133.8, 123.1, 79.4, 50.1, 44.0, 42.6, 39.1, 36.5, 28.6, 28.5.

HRMS (ESI^+) Found $[M+H]^+$ = 329.2222; $C_{20}H_{29}O_2N_2$ requires 329.2224.

IR (film) ν_{max}/cm^{-1} 2962, 2925, 2865, 1692, 1421, 1236, 1164

***tert*-Butyl 4-(3-(6-ethoxypyridin-3-yl)bicyclo[1.1.1]pentan-1-yl)piperidine-1-carboxylate, 2w**

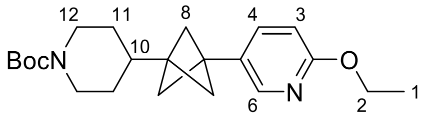

(6-ethoxypyridin-3-yl)magnesium bromide lithium chloride complex: 5-bromo-2-ethoxypyridine (606 mg, 3.0 mmol, 1.0 equiv.), Mg turnings (146 mg, 6.0 mmol, 2.0 equiv.) and LiCl (153 mg, 3.6 mmol, 1.2 equiv.) were submitted to **General Procedure 3** at 60 °C for 2 h to give (6-ethoxypyridin-3-yl)magnesium bromide lithium chloride complex as a 0.6 M solution in THF.

1b (76 mg, 0.20 mmol, 1.0 equiv.), Fe(acac)$_3$ (14 mg, 0.04 mmol, 20 mol%), TMEDA (12 μL, 0.08 mmol, 40 mol%) and (6-ethoxypyridin-3-yl)magnesium bromide lithium chloride complex (1.1 mL, 0.6 M in THF, 0.60 mmol, 3.0 equiv.) were submitted to **General Procedure 4** at 45 °C. The reaction was quenched with aqueous NH_4Cl (5 mL, saturated). Purification by column chromatography (SiO_2, pentane / Et_2O, 85:15) afforded **2w** (37 mg, 0.09 mmol, 49%) as a colourless oil.

$\mathbf{R_f}$ 0.24 (pentane / Et_2O, 75:25)

^{1}H NMR (400 MHz, $CDCl_3$) δ 7.97 (1H, d, *J* = 2.4 Hz, H6), 7.43 (1H, dd, *J* = 8.5, 2.4 Hz, H4), 6.66 (1H, d, *J* = 8.5 Hz, H3), 4.34 (2H, q, *J* = 7.1 Hz, H2), 4.15 (2H, app s, H12), 2.65 (2H, app t, *J* = 13.0 Hz, H12), 1.84 (6H, s, H8), 1.64 – 1.56 (2H, m, H11), 1.56 – 1.49 (1H, m, H10), 1.45 (9H, s, *t*-Bu), 1.38 (3H, t, *J* = 7.1 Hz, H1), 1.17 – 1.04 (2H, m, H11).

^{13}C NMR (126 MHz, $CDCl_3$) δ 162.6, 155.0, 144.1, 137.4, 129.5, 110.6, 79.4, 62.2, 50.1, 44.0, 42.5, 38.6, 36.5, 28.6, 28.5, 14.8.

HRMS (ESI$^+$) Found $[M+H]^+$ = 373.2497; $C_{22}H_{33}O_3N_2$ requires 373.2486.

IR (film) ν_{max}/cm^{-1} 3016, 2928, 2866, 2366, 1693, 1607, 1568, 1283, 1164.

***tert*-Butyl 4-(3-(5-methoxypyridin-3-yl)bicyclo[1.1.1]pentan-1-yl)piperidine-1-carboxylate, 2x**

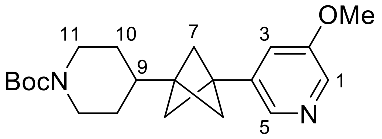

(5-Methoxypyridin-3-yl)magnesium bromide lithium chloride complex: (3-Bromo-5-methoxypyridine (564 mg, 3.0 mmol, 1.0 equiv.), Mg turnings (146 mg, 6.0 mmol, 2.0 equiv.) and LiCl (140 mg, 3.3 mmol, 1.1 equiv.) were submitted to **General Procedure 3** at 0 °C for 2 h to give (5-methoxypyridin-3-yl)magnesium bromide lithium chloride complex as a 0.7 M solution in THF.

1b (76 mg, 0.20 mmol, 1.0 equiv.), Fe(acac)$_3$ (14 mg, 0.04 mmol, 20 mol%), TMEDA (12 μL, 0.08 mmol, 40 mol%) and (5-methoxypyridin-3-yl)magnesium bromide lithium chloride complex (0.86 mL, 0.7 M in

THF, 0.60 mmol, 3.0 equiv.) were submitted to **General Procedure 4** at 45 °C. The reaction was quenched with aqueous NH_4Cl (5 mL, saturated). Purification by column chromatography (SiO_2, pentane / EtOAc, 70:30) afforded **2x** (39 mg, 0.12 mmol, 55%) as a colourless oil.

R$_f$ = 0.55 (pentane / EtOAc, 50:50)

^{1}H NMR (400 MHz, $CDCl_3$) δ 8.13 (1H, d, *J* = 2.9 Hz, H1), 8.06 (1H, d, *J* = 1.7 Hz, H5), 6.97 (1H, dd, *J* = 2.9, 1.7 Hz, H3), 4.26-4.03 (2H, m, H11), 3.83 (3H, s, OMe), 2.80-2.53 (2H, m, H11), 1.87 (6H, s, H7), 1.67-1.48 (3H, m, H9, H10), 1.44 (9H, s, *t*-Bu), 1.18-1.06 (2H, m, H10).

^{13}C NMR (101 MHz, $CDCl_3$) δ 155.5, 155.0, 140.4, 137.2, 135.5, 118.4, 79.4, 55.6, 50.1, 43.8, 42.6, 38.9, 36.5, 28.6, 28.5.

HRMS (ESI$^+$) Found $[M+H]^+$ = 359.2337; $C_{21}H_{31}O_3N_2$ requires 359.2340.

IR (film) ν_{max}/cm^{-1} 2965, 2930, 2866, 1687, 1419, 1234, 1152, 873

***tert*-butyl 4-(3-(2-methoxypyridin-4-yl)bicyclo[1.1.1]pentan-1-yl)piperidine-1-carboxylate, 2y**

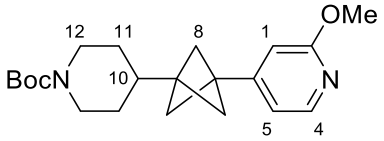

(2-Methoxypyridin-4-yl)magnesium bromide lithium chloride complex: 4-Bromo-2-methoxypyridine (564 mg, 2.0 mmol, 1.0 equiv.), Mg turnings (146 mg, 6.0 mmol, 2.0 equiv.) and LiCl (140 mg, 3.3 mmol, 1.1 equiv.) were submitted to **General Procedure 3** at room temperature for 2 h to give (2-methoxypyridin-4-yl)magnesium bromide lithium chloride complex as a 0.5 M solution in THF.

1b (76 mg, 0.20 mmol, 1.0 equiv.), $Fe(acac)_3$ (14 mg, 0.04 mmol, 20 mol%), TMEDA (12 µL, 0.08 mmol, 40 mol%) and (2-methoxypyridin-4-yl)magnesium bromide lithium chloride complex (1.2 mL, 0.5 M in THF, 0.32 mmol, 3.0 equiv.) were submitted to **General Procedure 4** at 45 °C. The reaction was quenched with aqueous NH_4Cl (5 mL, saturated). Purification by column chromatography (SiO_2, pentane / Et_2O, 80:20) afforded **2y** (25 mg, 0.07 mmol, 35%) as a clear oil.

R$_f$ 0.42 (pentane / Et_2O, 4:1)

m.p. 79-80 °C

^{1}H NMR (400 MHz, $CDCl_3$) δ 8.05 (1H, dd, *J* = 5.2, 0.8 Hz, H4), 6.71 (1H, dd, *J* = 5.2, 1.4 Hz, H5), 6.53 (1H, dd, *J* = 1.4, 0.8 Hz, H1), 4.17-4.13 (2H, m, H12), 3.91 (3H, s, OMe), 2.65 (2H, app t, *J* = 13.0 Hz, H12), 1.83 (6H, s, H8), 1.64-1.48 (3H, m, H10, H11), 1.24-1.03 (2H, m, H11).

^{13}C NMR (101 MHz, $CDCl_3$) δ 164.6, 155.0, 152.7, 146.7, 115.0, 108.2, 79.4, 53.5, 49.9, 43.9, 42.4, 40.1, 36.5, 28.6, 28.5.

HRMS (ESI$^+$) Found $[M+H]^+$ = 359.2330; $C_{21}H_{31}O_3N_2$ requires 359.2329.

IR (film) ν_{max}/cm^{-1} 2980, 1692, 1612, 1392, 1155.

***tert*-Butyl 4-(3-(benzofuran-5-yl)bicyclo[1.1.1]pentan-1-yl)piperidine-1-carboxylate, 2z**

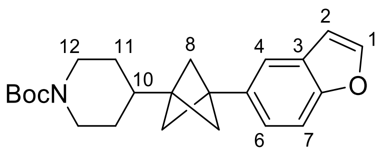

Benzofuran-5-ylmagnesium bromide lithium chloride complex: 5-bromobenzofuran (0.38 mL, 3.00 mmol, 1.0 equiv.), Mg turnings (146.0 mg, 6.00 mmol, 2.0 equiv.) and LiCl (153 mg, 3.60 mmol, 1.2 equiv.) were submitted to **General Procedure 3** at 60 °C for 2 h to give benzofuran-5-ylmagnesium bromide lithium chloride complex as a 1.0 M solution in THF.

1b (76 mg, 0.20 mmol, 1.0 equiv.), $Fe(acac)_3$ (14 mg, 0.04 mmol, 20 mol%), TMEDA (12 µL, 0.08 mmol, 40 mol%) and benzofuran-5-ylmagnesium bromide lithium chloride complex (0.32 mL, 1.0 M in THF, 0.320 mmol, 1.6 equiv.) were submitted to **General Procedure 4** at room temperature. The reaction was quenched with aqueous NH_4Cl (5 mL, saturated). Purification by column chromatography (SiO_2, pentane / Et_2O, 90:10) afforded **2z** (49 mg, 0.13 mmol, 67%) as a white solid.

$\mathbf{R_f}$ 0.17 (pentane / Et_2O, 90:10)

m.p. 103-105 °C

^{1}**H NMR** (400 MHz, $CDCl_3$) δ 7.59 (1H, d, *J* = 2.2 Hz, ArH), 7.44-7.40 (2H, m, ArH), 7.16 (1H, dd, *J* = 8.5, 1.7 Hz, ArH), 6.72 (1H, dd, *J* = 2.2, 0.9 Hz, ArH), 4.16 (2H, br app s, H12), 2.68 (2H, app t, *J* = 13.0 Hz, H12), 1.88 (6H, s, H8), 1.68-1.52 (3H, m, H11 + H10), 1.47 (9H, s, *t*-Bu), 1.21-1.08 (2H, m, H11).

^{13}**C NMR** (101 MHz, $CDCl_3$) δ 155.0, 154.0, 145.4, 136.2, 127.4, 122.6, 118.5, 111.0, 106.6, 79.4, 50.3, 44.0, 41.7, 41.1, 36.6, 28.6, 28.6.

HRMS (ESI$^+$) Found $[M+Na]^+$ = 390.2042; $C_{23}H_{29}O_3NNa$ requires 390.2040.

IR (film) ν_{max}/cm^{-1} 3656, 2980, 2865, 2361, 2341, 1691, 1236, 1173, 1150.

***tert*-butyl 4-(3-(4-(trifluoromethyl)benzyl)bicyclo[1.1.1]pentan-1-yl)indoline-1-carboxylate, 2aa**

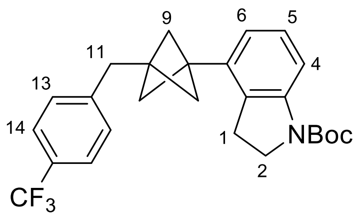

(1-(*tert*-Butoxycarbonyl)indolin-4-yl)magnesium bromide lithium chloride complex: *tert*-Butyl 4-bromoindoline-1-carboxylate (596 mg, 2.0 mmol, 1.0 equiv.), Mg turnings (97 mg, 4.0 mmol, 1.5 equiv.) and LiCl (93 mg, 2.2 mmol, 1.1 equiv.) were submitted to **General Procedure 3** at room temperature for 3 h to give (1-(*tert*-butoxycarbonyl)indolin-4-yl)magnesium bromide lithium chloride complex as a 0.7 M solution in THF.

1a (70 mg, 0.20 mmol, 1.0 equiv.), $Fe(acac)_3$ (14 mg, 0.04 mmol, 20 mol%), TMEDA (12 μL, 0.08 mmol, 40 mol%) and (1-(*tert*-butoxycarbonyl)indolin-4-yl)magnesium bromide lithium chloride complex (0.5 mL, 0.7 M in THF, 0.32 mmol, 1.6 equiv.) were submitted to **General Procedure 4** at 45 °C. The reaction was quenched with aqueous NH_4Cl (5 mL, saturated). Purification by column chromatography (SiO_2, pentane / Et_2O, 90:10) afforded **2aa** (57 mg, 0.13 mmol, 65%) as a clear oil.

$\mathbf{R_f}$ 0.43 (pentane / Et_2O, 70:30)

m.p. 122-123 °C

^{1}**H NMR** (400 MHz, $CDCl_3$) δ 7.75 (1H, m, H4), 7.57 (2H, d, *J* = 8.0 Hz, H14), 7.28-7.21 (2H, d, *J* = 8.0 Hz, H13), 7.09 (1H, app t, *J* = 7.7 Hz, H5), 6.70 (1H, dd, *J* = 7.7, 1.1 Hz, H6), 3.95 (2H, t, *J* = 8.7 Hz, H2), 3.06 (2H, t, *J* = 8.7 Hz, H1), 2.91 (2H, s, H11), 1.93 (6H, s, H9), 1.56 (9H, s, *t*Bu).

^{13}**C NMR {^{19}F}** (125 MHz, $CDCl_3$) δ 152.7, 143.6, 142.9, 136.4, 129.3, 128.4, 127.5, 125.3, 124.5, 120.9, 113.3, 81.0, 52.0, 47.7, 42.3, 40.1, 39.0, 28.6, 26.6. *(one aromatic signal overlapping).*

HRMS (ESI^+) Found $[M+Na]^+$ = 466.1967; $C_{26}H_{28}O_2NF_3Na$ requires 466.1964.

IR (film) ν_{max}/cm^{-1} 2981, 2883, 1763, 1247, 1157.

***tert*-Butyl 5-(3-(1-(tert-butoxycarbonyl)piperidin-4-yl)bicyclo[1.1.1]pentan-1-yl)-1*H*-indole-1-carboxylate, 2ab**

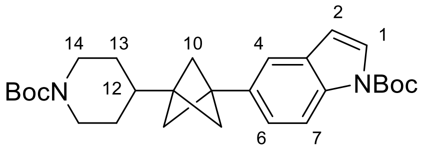

(1-(*tert*-Butoxycarbonyl)-1*H*-indol-5-yl)magnesium bromide lithium chloride complex: (*tert*-Butyl 5-bromo-1*H*-indole-1-carboxylate (564 mg, 3.0 mmol, 1.0 equiv.), Mg turnings (146 mg, 3.2 mmol, 2.0

equiv.) and LiCl (140 mg, 3.30 mmol, 1.1 equiv.) were submitted to **General Procedure 3** at room temperature for 3 h to give (1-(*tert*-butoxycarbonyl)-1*H*-indol-5-yl)magnesium bromide lithium chloride complex as a 0.7 M solution in THF.

1b (76 mg, 0.20 mmol, 1.0 equiv.), $Fe(acac)_3$ (14 mg, 0.04 mmol, 20 mol%), TMEDA (12 μL, 0.08 mmol, 40 mol%) and (1-(*tert*-butoxycarbonyl)-1*H*-indol-5-yl)magnesium bromide lithium chloride complex (0.46 mL, 0.7 M in THF, 0.60 mmol, 1.6 equiv.) were submitted to **General Procedure 4** at 45 °C. The reaction was quenched with aqueous NH_4Cl (5 mL, saturated). Purification by column chromatography (SiO_2, pentane / Et_2O, 90:10) afforded **2ab** (45 mg, 0.10 mmol, 48%) as a clear oil.

R_f 0.43 (pentane / Et_2O, 80:20)

^{1}H NMR (400 MHz, $CDCl_3$) δ 8.05 (1H, app d, *J* = 8.5 Hz, H7), 7.57 (1H, d, *J* = 3.8 Hz, H1), 7.38 (1H, dd, *J* = 1.7, 0.8 Hz, H4), 7.18 (1H, dd, *J* = 8.5, 1.7 Hz, H6), 6.52 (1H, dd, *J* = 3.8, 0.8 Hz, H2), 4.18-4.14 (2H, m, H14), 2.68 (2H, app t, *J* = 12.8 Hz, H14), 1.88 (6H, s, H10), 1.66 (9H, s, *t*-Bu), 1.64-1.50 (3H, m, H12, H13), 1.47 (9H, s, *t*-Bu), 1.15 (2H, app qd, *J* = 12.8, 4.4 Hz, H13).

^{13}C NMR (101 MHz, $CDCl_3$) δ 155.0, 149.9, 136.0, 134.0, 130.7, 126.3, 122.6, 118.3, 114.9, 107.3, 83.7, 79.4, 50.2, 44.0, 41.8, 41.2, 36.6, 28.6, 28.6, 28.3.

HRMS (ESI$^+$) Found $[M+Na]^+$ = 489.2723; $C_{28}H_{38}O_4N_2Na$ requires 489.2724.

IR (film) ν_{max}/cm^{-1} 2980, 2889, 1734, 1693, 1380, 1151.

***tert*-Butyl 4-(3-(1-methyl-1H-indazol-5-yl)bicyclo[1.1.1]pentan-1-yl)piperidine-1-carboxylate, 2ac**

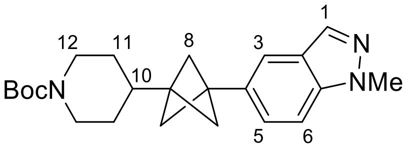

(3-Methyl-3*H*-indazol-6-yl)magnesium bromide lithium chloride complex: 6-Bromo-3-methyl-3*H*-indazole (422 mg, 2.0 mmol, 1.0 equiv.), Mg turnings (71 mg, 3.0 mmol, 1.5 equiv.) and LiCl (93 mg, 2.2 mmol, 1.1 equiv.) were submitted to **General Procedure 3** at room temperature for 2 h to give (3-methyl-3*H*-indazol-6-yl)magnesium bromide lithium chloride complex as a 0.7 M solution in THF.

1b (76 mg, 0.20 mmol, 1.0 equiv.), $Fe(acac)_3$ (14 mg, 0.04 mmol, 20 mol%), TMEDA (12 μL, 0.08 mmol, 40 mol%) and (3-methyl-3*H*-indazol-6-yl)magnesium bromide lithium chloride complex (0.5 mL, 0.7 M in THF, 0.32 mmol, 1.6 equiv.) were submitted to **General Procedure 4** at room temperature. The reaction was quenched with aqueous NH_4Cl (5 mL, saturated). Purification by column chromatography (SiO_2, pentane / Et_2O, 70:30) afforded **2ac** (35 mg, 0.09 mmol, 46%) as a white solid.

R_f 0.11 (pentane / Et_2O, 70:30)

m.p. 146-148 °C

^{1}H NMR (400 MHz, $CDCl_3$) δ 7.91 (1H, d, *J* = 0.8 Hz, H1), 7.51 (1H, app t, *J* = 1.2 Hz, H3), 7.36-7.27 (2H, m, H5, H6), 4.23-4.10 (2H, m, H12), 4.05 (3H, s, Me), 2.68 (2H, app t, *J* = 12.7 Hz, H12), 1.89 (6H, s, H8), 1.69-1.51 (3H, m, H10, H11), 1.47 (9H, s, *t*-Bu), 1.22-1.08 (2H, m, H11).

^{13}C NMR (101 MHz, $CDCl_3$) δ 155.1, 139.1, 134.0, 132.6, 125.2, 124.2, 117.8, 108.7, 79.4, 50.2, 43.7, 41.9, 41.2, 36.6, 35.7, 28.6, 28.6.

HRMS (ESI$^+$) Found $[M+H]^+$ = 382.2489; $C_{23}H_{32}O_2N_3$ requires 382.2489.

IR (film) ν_{max}/cm^{-1} 2961, 2864, 1689, 1423, 1236, 1174, 1150

***tert*-Butyl 4-(3-(9-phenyl-9*H*-carbazol-3-yl)bicyclo[1.1.1]pentan-1-yl)piperidine-1-carboxylate**, **2ad**

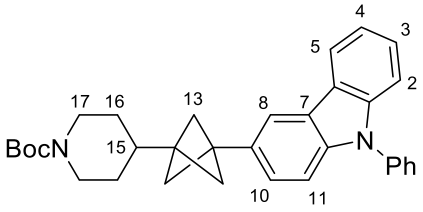

(9-Phenyl-9*H*-carbazol-3-yl)magnesium bromide: 3-bromo-9-phenyl-9*H*-carbazole (967 mg, 3.0 mmol, 1.0 equiv.), and Mg turnings (88 mg, 3.6 mmol, 1.2 equiv.) were submitted to **General Procedure 2** for 3 h at reflux to give (9-phenyl-9*H*-carbazol-3-yl)magnesium bromide as a 1.0 M solution in THF.

1b (76 mg, 0.20 mmol, 1.0 equiv.), $Fe(acac)_3$ (14 mg, 0.04 mmol, 20 mol%), TMEDA (12 μL, 0.08 mmol, 40 mol%) and (9-phenyl-9*H*-carbazol-3-yl)magnesium bromide (0.46 mL, 1.0 M in THF, 0.32 mmol, 1.6 equiv.) were submitted to **General Procedure 4** at 45 °C. The reaction was quenched with aqueous NH_4Cl (5 mL, saturated). Purification by column chromatography (SiO_2, pentane / Et_2O, 90:10) afforded **2ad** (64 mg, 0.13 mmol, 65%) as a clear oil.

R_f = 0.43 (pentane / Et_2O, 80:20)

^{1}H NMR (400 MHz, $CDCl_3$) δ 8.14 (1H, dt, *J* = 7.8, 1.0 Hz, ArH), 7.96 (1H, dd, *J* = 1.6, 0.7 Hz, ArH), 7.63-7.52 (4H, m, ArH), 7.49-7.43 (1H, m, ArH), 7.41-7.38 (2H, m, ArH), 7.35-7.26 (3H, m, ArH), 4.29-4.11 (2H, m, H17), 2.77-2.65 (2H, m, H17), 1.96 (6H, s, H13), 1.71-1.54 (3H, m, H15, H16), 1.48 (9H, s, *t*-Bu), 1.20 (2 H, app qd, *J* = 13.3, 12.2, 9.2 Hz, H16).

^{13}C NMR (101 MHz, $CDCl_3$) δ 155.1, 141.3, 139.9, 138.0, 133.4, 130.0, 127.5, 127.2, 126.0, 124.3, 123.42, 123.39, 120.4, 119.9, 117.7, 109.9, 109.5, 79.4, 50.4, 43.9, 41.8, 41.4, 36.7, 28.7, 28.6.

HRMS (ESI$^+$) Found $[M+H]^+$ = 493.2849; $C_{33}H_{37}O_2N_2$ requires 493.2850.

IR (film) ν_{max}/cm^{-1} 2980, 2865, 1688, 1234, 1154.

tert-Butyl 3-(3-(4-methoxyphenyl)bicyclo[1.1.1]pentan-1-yl)azetidine-1-carboxylate, 2ae

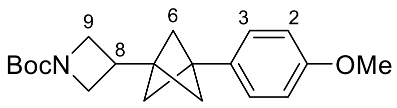

1c (70 mg, 0.20 mmol, 1.0 equiv.), Fe(acac)$_3$ (14 mg, 0.04 mmol, 20 mol%), TMEDA (12 μL, 0.08 mmol, 40 mol%) and 4-methoxyphenylmagnesium bromide (0.4 mL, 0.9 M in THF, 0.32 mmol, 1.6 equiv.) were submitted to **General Procedure 4** at room temperature. The reaction was quenched with aqueous NH_4Cl (5 mL, saturated). Purification by column chromatography (SiO_2, pentane / Et_2O, 95:5) afforded **2ae** (48 mg, 0.12 mmol, 73%) as a white solid.

R$_f$ 0.35 (pentane / Et_2O, 95:5)

^{1}H NMR (400 MHz, $CDCl_3$) δ 7.14 (2H, m, H3), 6.88-6.79 (2H, m, H2), 3.94 (2H, app t, *J* = 8.4 Hz, H9), 3.79 (3H, s, OMe), 3.67 (2H, dd, *J* = 8.4, 5.4 Hz, H9), 2.66 (1H, app tt, *J* = 8.4, 5.4 Hz, H8), 1.92 (6H, s, H6), 1.45 (9H, s, *t*-Bu).

^{13}C NMR (101 MHz, $CDCl_3$) δ 158.5, 156.7, 133.3, 127.2, 113.7, 79.4, 55.4, 51.2, 50.2, 41.6, 39.5, 29.4, 28.6.

HRMS (ESI$^+$) Found $[M+Na]^+$ = 352.1883; $C_{20}H_{27}O_3NNa$ requires 352.1883.

IR (film) ν_{max}/cm^{-1} 2960, 2867, 1699, 1401, 1365, 1245, 1135.

1-(4-Methoxyphenyl)-3-((phenylsulfonyl)methyl)bicyclo[1.1.1]pentane, 2af

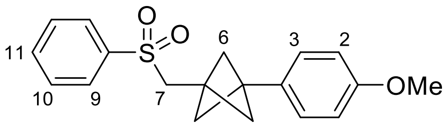

1-Iodo-3-((phenylsulfonyl)methyl)bicyclo[1.1.1]pentane **1d** (70 mg, 0.20 mmol, 1.0 equiv.), Fe(acac)$_3$ (14 mg, 0.04 mmol, 20 mol%), TMEDA (12 μL, 0.08 mmol, 40 mol%) and 4-methoxyphenylmagnesium bromide (0.4 mL, 0.83 M in THF, 0.32 mmol, 1.6 equiv.) were submitted to **General Procedure 4** at room temperature. The reaction was quenched with aqueous HCl (5 mL, 1 M). Purification by column chromatography (SiO_2, pentane / EtOAc, 80:20) afforded **2af** (44 mg, 0.13 mmol, 67%) as a white solid.

R$_f$ 0.35 (pentane / EtOAc, 80:20)

m.p. 107-109 °C

^{1}H NMR (400 MHz, $CDCl_3$) δ 7.97-7.90 (2H, m, H9), 7.66 (1H, m, H11), 7.61-7.54 (2H, m, H10), 7.09-7.04 (2H, m, H3), 6.84-6.78 (2H, m, H2), 3.77 (3H, s, OMe), 3.43 (2H, s, H7), 2.04 (6H, s, H6).

^{13}C NMR (101 MHz, $CDCl_3$) δ 158.6, 140.5, 133.8, 132.4, 129.4, 128.0, 127.2, 113.7, 57.6, 55.4, 53.9, 43.2, 32.0.

HRMS (ESI^+) Found $[M+Na]^+$ = 351.1026; $C_{19}H_{20}O_3NaS$ requires 351.1025.

IR (film) ν_{max}/cm^{-1} 2971, 2910, 2873, 1521, 1307, 1247, 1147, 1086, 746.

Ethyl 2-(3-(4-methoxyphenyl)bicyclo[1.1.1]pentan-1-yl)acetate, 2ag

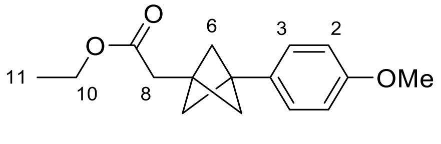

Ethyl 2-(3-iodobicyclo[1.1.1]pentan-1-yl)acetate **1e** (58 mg, 0.21 mmol, 1.0 equiv.), $Fe(acac)_3$ (14 mg, 0.04 mmol, 20 mol%), TMEDA (12 µL, 0.08 mmol, 40 mol%) and 4-methoxyphenylmagnesium bromide (0.4 mL, 0.8 M in THF, 0.32 mmol, 1.6 equiv.) were submitted to **General Procedure 4** at room temperature. The reaction was quenched with aqueous NH_4Cl $_{(sat.)}$ (5 mL). Purification by column chromatography (SiO_2, pentane / Et_2O, 98:2 to 96:4) afforded **2ag** (51 mg, 0.20 mmol, 95%) as a colourless liquid.

R$_f$ 0.19 (pentane / Et_2O, 95:5)

^{1}H NMR (400 MHz, $CDCl_3$) δ 7.17-7.10 (2H, m, H2), 6.88-6.80 (2H, m, H3), 4.16 (2H, q, *J* = 7.1 Hz, H10), 3.79 (3H, s, OMe), 2.58 (2H, s, H8), 2.01 (6H, s, H6), 1.28 (3H, t, *J* = 7.1 Hz, H11).

^{13}C NMR (101 MHz, $CDCl_3$) δ 171.7, 158.4, 133.3, 127.2, 113.7, 60.4, 55.4, 53.2, 41.9, 37.7, 35.0, 14.5.

HRMS [ESI^+, EI^+, CI^+] Not found.

IR (film) ν_{max}/cm^{-1} 3657, 2980, 2906, 2360, 1735, 1247.

Ethyl 2,2-difluoro-2-(3-(4-methoxyphenyl)bicyclo[1.1.1]pentan-1-yl)acetate, 2ah

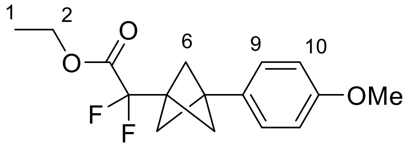

Ethyl 2,2-difluoro-2-(3-iodobicyclo[1.1.1]pentan-1-yl)acetate **1f** (63 mg, 0.20 mmol, 1.0 equiv.), $Fe(acac)_3$ (14 mg, 0.04 mmol, 20 mol%), TMEDA (12 µL, 0.08 mmol, 40 mol%) and 4-methoxyphenylmagnesium bromide (0.4 mL, 0.8 M in THF, 0.32 mmol, 1.6 equiv.) were submitted to **General Procedure 4** at room temperature. The reaction was quenched with aqueous HCl (5 mL, 1 M). Purification by column chromatography (SiO_2, pentane / Et_2O 98:2) afforded **2ah** (20 mg, 0.07 mmol, 34%) as a colourless oil.

R$_f$ 0.17 (pentane / Et_2O 98:2)

^{1}H NMR (400 MHz, $CDCl_3$) δ 7.13 (2H, d, *J* = 8.7 Hz, H9), 6.85 (2H, d, *J* = 8.7 Hz, H10), 4.36 (2H, q, *J* = 7.1 Hz, H2), 3.80 (3H, s, OMe), 2.16 (6H, s, H6), 1.37 (3H, t, *J* = 7.1 Hz, H1).

^{13}C NMR (101 MHz, $CDCl_3$) δ 163.5, 158.9, 131.7, 127.3, 113.9, 112.5 (t, *J* = 249.8 Hz), 62.8, 55.5 (t, *J* = 3.2 Hz), 50.7, 41.2, 37.7 (t, *J* = 31.5 Hz), 14.3.

^{19}F NMR (376 MHz, $CDCl_3$) δ –111.31.

HRMS (ESI^+) Found $[M+H]^+$ = 297.1298; $C_{16}H_{19}O_3F_2$ requires 297.1297.

IR (film) ν_{max}/cm^{-1} 2980, 2884, 1736, 1247, 1157.

2-(3-(4-Methoxyphenyl)bicyclo[1.1.1]pentan-1-yl)pyridine, 2ai

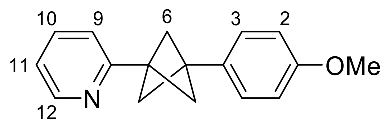

1g (55 mg, 0.20 mmol, 1.0 equiv.), $Fe(acac)_3$ (14 mg, 0.04 mmol, 20 mol%), TMEDA (12 µL, 0.08 mmol, 40 mol%) and 4-methoxyphenylmagnesium bromide (0.4 mL, 0.9 M in THF, 0.32 mmol, 1.6 equiv.) were submitted to **General Procedure 4** at room temperature. The reaction was quenched with aqueous HCl (5 mL, 1 M). Purification by column chromatography (SiO_2, pentane / Et_2O, 80:20) afforded **2ai** (35 mg, 0.14 mmol, 69%) as a white solid.

R_f 0.16 (pentane / Et_2O, 70:30)

m.p. 81-83 °C

^{1}H NMR (400 MHz, $CDCl_3$) δ 8.59 (1H, ddd, *J* = 4.9, 1.9, 1.0 Hz, H12), 7.64 (1H, td, *J* = 7.6, 1.9 Hz, H10), 7.30-7.21 (3H, m, H3, H11), 7.15 (1H, ddd, *J* = 7.6, 4.9, 1.2 Hz, H9), 6.94-6.86 (2H, m, H2), 3.81 (3H, s, OMe), 2.40 (6H, s, H6).

^{13}C NMR (101 MHz, $CDCl_3$) δ 159.9, 158.6, 149.5, 136.3, 133.2, 127.4, 121.6, 120.8, 113.8, 55.4, 53.9, 41.6, 40.8.

HRMS (ESI) Found $[M+H]^+$ = 252.1382; $C_{17}H_{18}ON$ requires 252.1383.

IR (film) ν_{max}/cm^{-1} 2973, 2909, 2971, 1587, 1505, 1246, 802.

2,6-*bis*(3-(4-Methoxyphenyl)bicyclo[1.1.1]pentan-1-yl)pyridine, 2aj

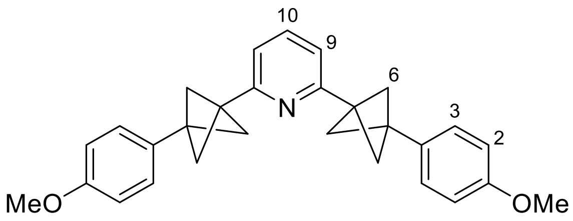

1h (93 mg, 0.20 mmol, 1.0 equiv.), $Fe(acac)_3$ (28 mg, 0.08 mmol, 40 mol%), TMEDA (24 µL, 0.16 mmol, 80 mol%) and 4-methoxyphenylmagnesium bromide (0.91 mL, 0.7 M in THF, 0.636 mmol, 3.2 equiv.) were submitted to **General Procedure 4** at room temperature. The reaction was quenched with aqueous NH_4Cl (5 mL, saturated). Purification by column chromatography (SiO_2, pentane / Et_2O, 95:5) afforded **2aj** (54 mg, 0.13 mmol, 64%) as a white solid.

R_f 0.55 (pentane / Et_2O, 70:30)

m.p. 198-200 °C

^{1}H NMR (400 MHz, $CDCl_3$) δ 7.56 (1H, t, J = 7.7 Hz, H10), 7.26 − 7.23 (4H, m, H3), 7.08 (2H, d, J = 7.7, Hz, H9), 6.89-6.85 (4H, m, H2), 3.82 (6H, s, OMe), 2.38 (12H, s, H6).

^{13}C NMR (101 MHz, $CDCl_3$) δ 159.4, 158.5, 136.0, 133.7, 127.4, 118.3, 113.8, 55.5, 54.0, 41.9, 40.8.

HRMS (ESI) Found $[M+H]^+$ = 424.2269; $C_{29}H_{30}O_2N$ requires 424.2271.

IR (film) ν_{max}/cm^{-1} 2978, 1505, 1246, 1030, 804.

Methyl 6-fluoro-2-(3-(4-methoxyphenyl)bicyclo[1.1.1]pentan-1-yl)-3-methylquinoline-4-carboxylate, 2ak

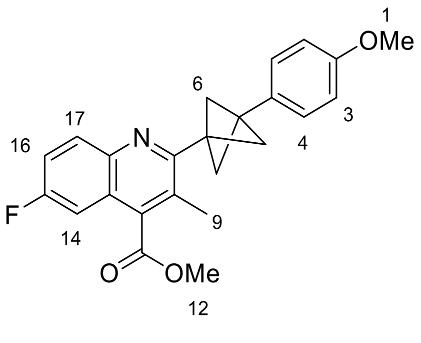

1i (83 mg, 0.20 mmol, 1.0 equiv.), $Fe(acac)_3$ (14 mg, 0.04 mmol, 20 mol%), TMEDA (12 µL, 0.08 mmol, 40 mol%) and 4-methoxyphenylmagnesium bromide (0.4 mL, 0.8 M in THF, 0.32 mmol, 1.6 equiv.) were submitted to **General Procedure 4**. The reaction was quenched with aqueous NH_4Cl (5 mL, saturated). Purification by column chromatography (SiO_2, pentane / Et_2O, 9:1) afforded **2ak** (65 mg, 0.17 mmol, 83%) as a white solid.

R_f 0.21 (pentane / Et_2O, 9:1)

m.p. 148−150 °C

^{1}H NMR (400 MHz, $CDCl_3$) δ 8.07 (1H, dd, J = 9.2, 5.5 Hz, H14), 7.42 (1H, ddd, J = 9.2, 8.4, 2.8 Hz, H16), 7.32-7.21 (3H, m, H4, H17), 6.92-6.86 (2H, m, H3), 4.08 (3H, s, OMe), 3.82 (3H, s, OMe), 2.58 (6H, s, H6), 2.58 (3H, s, H9).

^{13}C NMR (101 MHz, $CDCl_3$) δ 168.4, 161.0 (d, J = 248.1 Hz), 158.7, 157.9 (d, J = 2.8 Hz), 143.5, 138.1 (d, J = 5.4 Hz), 133.1, 132.3 (d, J = 9.3 Hz), 127.8, 127.4, 124.2 (d, J = 10.2 Hz), 119.1 (d, J = 25.6 Hz), 113.9, 107.9 (d, J = 23.4 Hz), 55.5, 54.6, 52.8, 43.4, 42.3, 17.4.

^{19}F NMR (377 MHz, $CDCl_3$) δ −112.25 (ddd, J = 5.5, 8.4, 9.9 Hz).

HRMS (ESI^+) $[M+H]^+$ = 392.1655; $C_{24}H_{23}O_3NF$ requires 392.1657.

IR (film) ν_{max}/cm^{-1} 2970, 1732, 1498, 1386, 1298, 1246.17, 1208, 1172, 833.

2-(3-(4-Methoxyphenyl)bicyclo[1.1.1]pentan-1-yl)ethyl 6-(3-methoxyphenyl)-2-methylnicotinate, 2al

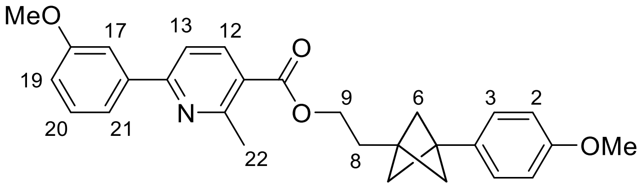

1j (93 mg, 0.20 mmol, 1.0 equiv.), $Fe(acac)_3$ (14 mg, 0.04 mmol, 20 mol%), TMEDA (12 μL, 0.08 mmol, 40 mol%) and 4-methoxyphenylmagnesium bromide (0.4 mL, 0.9 M in THF, 0.32 mmol, 1.6 equiv.) were submitted to **General Procedure 4** at room temperature. The reaction was quenched with aqueous NH_4Cl (5 mL, saturated). Purification by column chromatography (SiO_2, pentane / Et_2O, 95:5) afforded **2al** (35 mg, 0.08 mmol, 40%) as a white solid.

R_f 0.33 (pentane / Et_2O, 90:10)

m.p. 110-112 °C

^{1}H NMR (400 MHz, $CDCl_3$) δ 8.27 (1H, d, J = 8.1 Hz, H12), 7.66 (1H, dd, J = 2.6, 1.6 Hz, H17), 7.65-7.58 (2H, m, ArH), 7.39 (1H, app t, J = 8.1 Hz, H20), 7.18-7.09 (2H, m, H3), 7.00 (1H, ddd, J = 8.1, 2.6, 1.0 Hz, ArH), 6.89-6.79 (2H, m, H2), 4.41 (2H, t, J = 6.6 Hz, H9), 3.90 (3H, s, OMe), 3.78 (3H, s, OMe), 2.94 (3H, s, H22), 2.04 (2H, t, J = 6.6 Hz, H8), 1.99 (6H, s, H6).

^{13}C NMR (101 MHz, *d*6-acetone) δ 166.8, 161.2, 160.2, 159.4, 159.1, 140.5, 140.1, 134.1, 130.6, 127.8, 124.7, 120.3, 118.3, 116.3, 114.3, 113.4, 64.0, 55.7, 55.5, 53.3, 42.4, 37.2, 31.3, 25.4.

HRMS (ESI^+) Found $[M+H]^+$ = 444.2168; $C_{28}H_{30}O_4N$ requires 444.2169.

IR (film) ν_{max}/cm^{-1} 2904, 1719, 1584, 1246, 1083.

2-(3-phenylbicyclo[1.1.1]pentan-1-yl)propanoic acid, 8 (BCP-flurbiprofen)

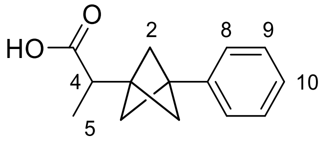

1k (147 mg, 0.50 mmol, 1.0 equiv.), $Fe(acac)_3$ (35 mg, 0.10 mmol, 20 mol%), TMEDA (30 μL, 0.20 mmol, 40 mol%) and phenylmagnesium bromide (0.80 mL, 1.0 M in THF, 0.80 mmol, 1.6 equiv.) were submitted to **General Procedure 4** at room temperature. Water (2 mL) was added and the aqueous was extracted with Et_2O (3 × 2 mL). To the resulting solution was slowly added NaOH (2 mL, 10% in MeOH). The resulting mixture was heated to reflux for 30 min, then cooled to room temperature and concentrated *in vacuo*. The resulting residue was diluted with water (10 mL) and acidified to pH 1 with conc. HCl. The aqueous phase was extracted with Et_2O (2 × 10 mL), dried (Na_2SO_4) and concentrated *in vacuo* to afford **8** (85 mg, 0.39 mmol, 78%) as a colourless oil.

R_f 0.30 (CH_2Cl_2 / MeOH, 98:2)

m.p. 83-84 °C

^{1}H NMR (400 MHz, $CDCl_3$) δ 7.33-7.27 (2H, m, ArH), 7.24-7.20 (3H, m, ArH), 2.73 (1H, q, J = 7.0 Hz, H4), 2.01 (6H, s, H2), 1.20 (3H, d, J = 7.0 Hz, H3).

^{13}C NMR (101 MHz, $CDCl_3$) δ 180.2, 140.8, 128.3, 126.6, 126.2, 51.2, 41.1, 40.8, 39.5, 13.5.

HRMS (ESI$^-$) Found [M−H]$^-$ = 215.1077; $C_{14}H_{15}O_2$ requires 215.1078.

IR (film) ν_{max}/cm^{-1} 2968, 2909, 2871, 1706, 1459, 1417, 1260, 1230, 1168.

Methyl 6-fluoro-2-(3-(4-fluorophenyl)bicyclo[1.1.1]pentan-1-yl)-3-methylquinoline-4-carboxylate, 9 (BCP-Brequinar analogue)

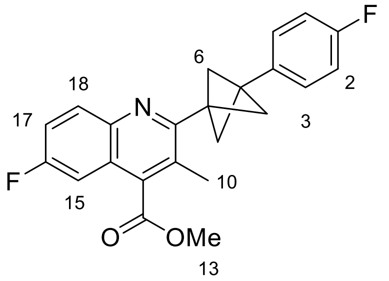

1i (83 mg, 0.20 mmol, 1.0 equiv.), $Fe(acac)_3$ (14 mg, 0.04 mmol, 20 mol%), TMEDA (12 μL, 0.08 mmol, 40 mol%) and 4-fluorophenylmagnesium bromide (0.16 mL, 2.0 M in Et_2O, 0.32 mmol, 1.6 equiv.) were submitted to **General Procedure 4**. The reaction was quenched with aqueous NH_4Cl (5 mL, saturated).

Purification by column chromatography (SiO_2, pentane / EtOAc, 95:5) afforded **9** (50 mg, 0.13 mmol, 66%) as a white solid.

R$_f$ 0.31 (pentane / EtOAc, 95:5)

m.p. 135-139 °C

^{1}H NMR (400 MHz, $CDCl_3$) δ 8.07 (1H, dd, J = 9.2, 5.5 Hz, H18), 7.43 (2H, ddd, J = 9.2, 8.3, 2.7, Hz, H17), 7.32-7.24 (3H, m, ArH), 7.08-6.98 (3H, m, ArH), 4.08 (3H, s, H13), 2.59 (6H, s, H6), 2.57 (3H, s, H10).

^{13}C NMR (101 MHz, $CDCl_3$) δ 168.4, 138.2 (d, J = 5.4 Hz), 162.0 (d, J = 244.8 Hz), 161.0 (d, J = 248.1 Hz), 157.5 (d, J = 2.9 Hz), 143.5, 136.6 (d, J = 3.1 Hz), 132.3 (d, J = 9.4 Hz), 127.9 (d, J = 8.0 Hz), 127.8, 124.2 (d, J = 10.2 Hz), 119.2 (d, J = 25.6 Hz), 115.2 (d, J = 21.3 Hz), 107.9 (d, J = 23.4 Hz), 54.7, 52.8, 43.4, 42.2, 17.4.

^{19}F NMR (377 MHz, $CDCl_3$) δ −116.04 (tt, J = 5.6, 9.0 Hz), −112.11 (ddd, J = 5.5, 8.3, 9.8 Hz).

HRMS (ESI$^+$) $[M+H]^+$ = 380.1457; $C_{23}H_{20}O_2NF$ requires 380.1457.

IR (film) ν_{max}/cm^{-1} 2975, 1732, 1500, 1387, 845, 831.

d) TMS *ipso*-substitution reactions

***tert*-Butyl 4-(3-(4-bromophenyl)bicyclo[1.1.1]pentan-1-yl)piperidine-1-carboxylate, 10**

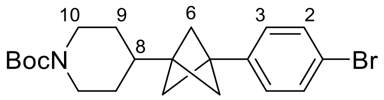

To a vial containing MeOH (0.15 mL) at 60 °C was added a mixture of **2m** (80 mg, 0.20 mmol) and KBr (36 mg, 1.5 equiv., 0.30 mmol) in AcOH (1.4 mL). The reaction was stirred for 20 mins. NCS (32 mg, 1.2 equiv., 0.24 mmol) was then added and the reaction was stirred at 60 °C for a further 2 h. The reaction was then cooled to room temperature and poured into ice water. The aqueous solution was extracted with CH_2Cl_2 (3 × 20 mL) and the combined organic layers washed with NaOH (3 M, 20 mL) and water (20 mL), before drying over $MgSO_4$ and concentrating *in vacuo*. Purification by column chromatography (SiO_2, pentane / Et_2O, 95:5) afforded **10** (56 mg, 0.14 mmol, 69%) as a white solid.

R$_f$ = 0.24 (pentane / Et_2O, 90:10)

m.p. = 83-85 °C

^{1}H NMR (400 MHz, $CDCl_3$) δ 7.39 (2H, d, *J* = 8.3 Hz, ArH), 7.07 (2H, d, *J* = 8.4 Hz, ArH), 4.15 (2H, br app s, H10), 2.66 (2H, app t, *J* = 12.9 Hz, H10), 1.83 (6H, s, H6), 1.64-1.49 (3H, m, H9 + H8), 1.46 (9H, s, *t*-Bu), 1.11 (2H, m , H9).

^{13}C NMR (101 MHz, $CDCl_3$) δ 155.0, 140.4, 131.2, 128.0, 120.3, 79.4, 50.0, 43.9, 41.9, 40.6, 36.5, 28.6, 28.5.

HRMS (ESI$^+$) Found $[M+Na]^+$ = 428.1196; $C_{21}H_{28}O_2N^{79}Br^{23}Na$ requires 428.1196.

IR (film) ν_{max}/cm^{-1} 2964, 2929, 2866, 1692, 1422, 1236, 1161

***tert*-Butyl 4-(3-(4-iodophenyl)bicyclo[1.1.1]pentan-1-yl)piperidine-1-carboxylate, 11**

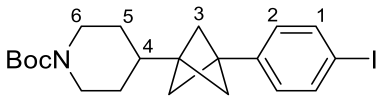

2m (40 mg, 0.10 mmol, 1.0 equiv.) in anhydrous CH_2Cl_2 (1 mL) was cooled to 0 °C and treated with ICl (0.2 mL of a 1 M solution in CH_2Cl_2, 0.2 M). The reaction was maintained at this temperature for 1 h then quenched with $Na_2S_2O_3$ (5 ml of a sat. aq. solution) and $NaHCO_3$ (5 ml of a sat. aq. solution). The phases were separated, and the aqueous phase was extracted with CH_2Cl_2 (3 × 5 mL). The combined organic layers were washed with brine, dried ($MgSO_4$), and concentrated. Purification by column chromatography (SiO_2, pentane / EtOAc, 9:1) afforded **11** (29 mg, 0.64 mmol, 64%) as a pale yellow solid.

R_f 0.54 (pentane / EtOAc, 80:20)

m.p. 78-80 °C

^{1}H NMR (400 MHz, $CDCl_3$) δ 7.64-7.56 (2H, m, ArH), 7.00-6.91 (2H, m, ArH), 4.22-4.07 (2H, m, H6), 2.66 (2H, app t, *J* = 12.2 Hz, H6), 1.82 (6H, s, H3), 1.63-1.49 (3H, m, H4, H5), 1.46 (9H, s, tBu), 1.19-1.04 (2H, m, H5).

^{13}C NMR (101 MHz, $CDCl_3$) δ 155.0, 141.1, 137.2, 128.3, 91.7, 79.4, 50.0, 43.9, 42.0, 40.7, 36.5, 28.6, 28.5.

HRMS (ESI$^+$) $[M+H]^+$ = 476.1057; $C_{21}H_{28}O_2NINa$ requires 476.1057.

IR (film) ν_{max}/cm^{-1} 2964, 2928, 2865, 1691, 1422, 1160.

***tert*-Butyl 4-(3-(4-acetylphenyl)bicyclo[1.1.1]pentan-1-yl)piperidine-1-carboxylate, 12**

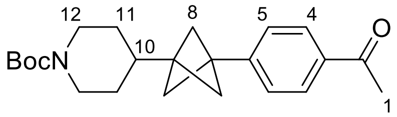

To a flame dried vial under argon was added $AlCl_3$ (40 mg, 0.3 mmol, 3 equiv.) and CH_2Cl_2 (0.5 mL). The mixture was cooled to 0 °C, then AcCl (20 µL, 3 equiv., 0.3 mmol) was added and the mixture stirred for 15 mins. A solution of **2m** (40 mg, 0.1 mmol, 1.0 equiv) in CH_2Cl_2 (0.25 mL) was added before warming to room temperature and stirring for 16 h. The reaction was quenched with cold aqueous NH_4Cl (sat., 5 mL), the layers separated, and the aqueous layer extracted with CH_2Cl_2 (2 × 15 mL). The combined organic phases were dried over $MgSO_4$ and concentrated. The crude product was dissolved in CH_2Cl_2 (0.5 mL) and cooled to 0 °C before adding Boc_2O (33 mg, 1.5 equiv., 0.15 mmol). NEt_3 (40 µL, 3 equiv., 0.3 mmol) was then added dropwise to reaction mixture and the resultant mixture stirred at room temperature for 16 h. Once complete by TLC, water was added to the reaction (10 mL), the layers were separated, and the aqueous layer extracted with CH_2Cl_2 (3 × 20 mL). the combined organic layers were dried over $MgSO_4$, concentrated *in vacuo* and purified by column chromatography (SiO_2, pentane / Et_2O, 90:10 to 80:20), affording **12** (25 mg, 0.07 mmol, 67%) as a white solid.

$\mathbf{R_f}$ = 0.21 (pentane / EtOAc, 80:20)

^{1}H NMR (400 MHz, $CDCl_3$) δ 7.91-7.85 (2H, m, H4), 7.31-7.27 (2H, m, H5), 4.14 (2H, br app s, H12), 2.68 (2H, m, H12), 2.57 (3H, s, H1), 1.88 (6H, s, H8), 1.65-1.52 (3H, m, H11 + H10), 1.46 (9H, s, *t*-Bu), 1.19-1.07 (2H, m, H11).

^{13}C NMR (101 MHz, $CDCl_3$) δ 197.9, 155.0, 146.9, 135.5, 128.5, 126.4, 79.4, 50.2, 43.8, 42.2, 41.0, 36.5, 28.6, 28.5, 26.7.

HRMS (ESI^+) $[M+Na]^+$ = 392.2194; $C_{23}H_{31}O_3NNa$ requires 392.2196.

IR (film) ν_{max}/cm^{-1} 3657, 2980, 2927, 2867, 2361, 1686, 1607, 1422, 1162.

e) Unsuccessful couplings

The following substrates failed in the Kumada coupling.

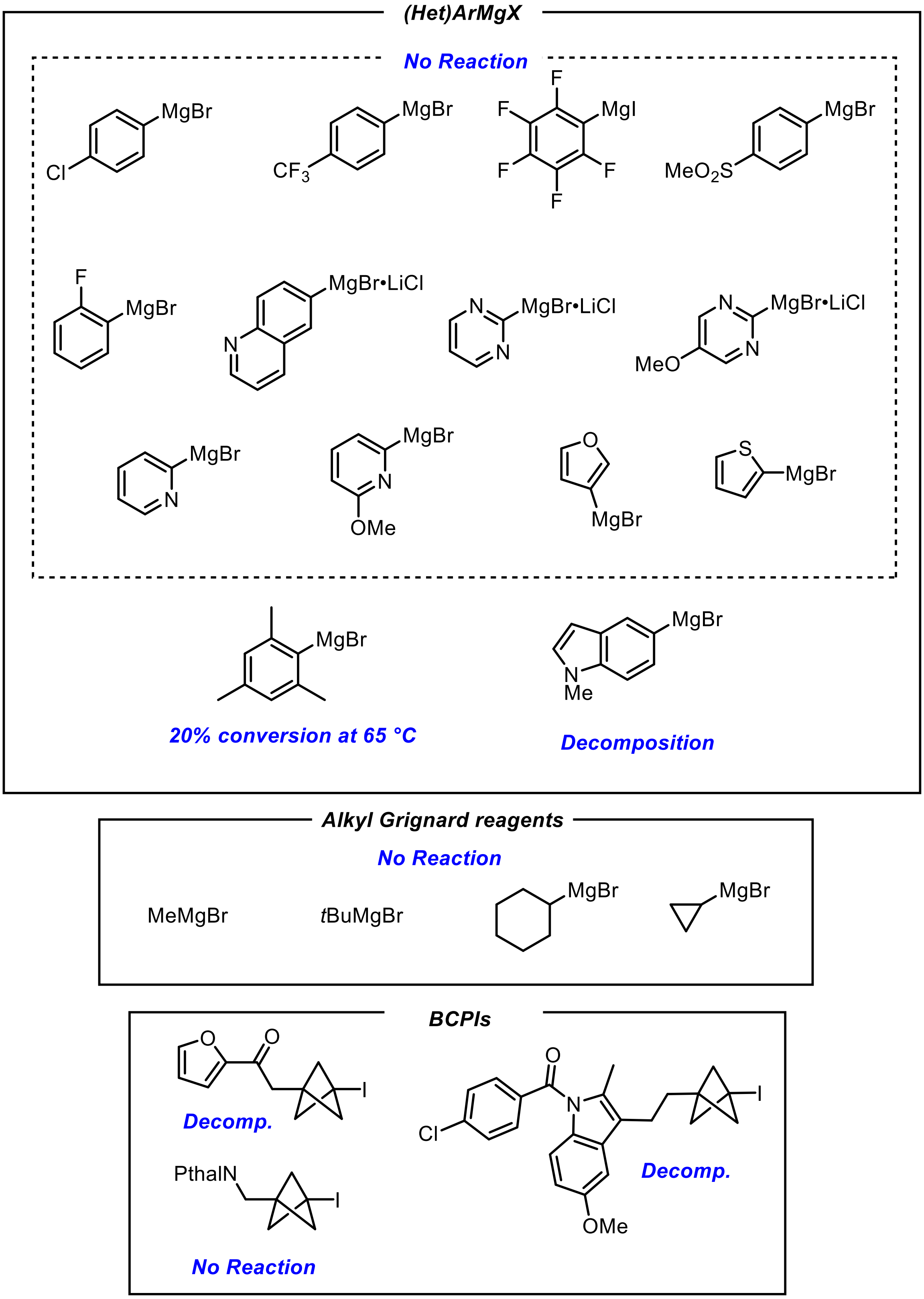

f) References

1. Caputo, D. F. J.; Arroniz, C.; Dürr, A. B.; Mousseau, J. J.; Stepan, A. F.; Mansfield, S. J.; Anderson, E. A., Synthesis and applications of highly functionalized 1-halo-3-substituted bicyclo[1.1.1]pentanes. *Chem. Sci.* **2018,** *9* (23), 5295-5300.

2. Nugent, J.; Arroniz, C.; Shire, B. R.; Sterling, A. J.; Pickford, H. D.; Wong, M. L. J.; Mansfield, S. J.; Caputo, D. F. J.; Owen, B.; Mousseau, J. J.; Duarte, F.; Anderson, E. A., A General Route to Bicyclo[1.1.1]pentanes through Photoredox Catalysis. *ACS Catal.* **2019,** *9* (10), 9568-9574.

3. Batt, D. G.; Petraitis, J. J.; Sherk, S. R. 2-Carbocyclic and 2-heterocyclic quinoline-4-carboxylic acids and salts thereof useful as immunosuppressive agents. PCT/US1996/006321, 1997.

4. Bedford, R. B.; Betham, M.; Bruce, D. W.; Davis, S. A.; Frost, R. M.; Hird, M., Iron nanoparticles in the coupling of alkyl halides with aryl Grignard reagents. *Chem. Commun.* **2006,** (13), 1398-1400.

6. X-ray Crystallography

Low temperature single crystal X-ray diffraction data were collected for **2a** and **9** using Oxford Diffraction (Rigaku) SuperNovae diffractometers at 150 K. These data were reduced using CrysAlisPro, solved using SuperFlip and the structures were refined using CRYSTALS. Further details about the refinements, including disorder modelling, are documented in the CIF. The crystallographic data have been deposited with the CCDC as entries CCDC 1991572-1991573.

Table S2. Crystal data and structure refinement for 2a.

CCDC Identification code	CCDC 1991572	
Empirical formula	C20 H19 F3 O1	
Formula Weight	332.36	
Temperature	150 K	
Wavelength	1.54184 Å	
Crystal system	Monoclinic	
Space Group	P 21/n	
Unit cell dimensions	a = 7.8808(1) Å	α = 90°
	b = 5.7926(1) Å	β = 91.0306(14)°
	c = 35.3177(5) Å	γ = 90°
Volume	1611.73(4) $Å^3$	
Z	4	
Density (calculated)	1.370 Mg/m^3	
Absorption coefficient	0.896 mm^{-1}	
F(000)	696	
Crystal size	0.10 x 0.10 x 0.02 mm^3	
Theta range for data collection	4.98 to 75.96°	
Index ranges	-8<=h<=9, -7<=k<=7, -44<=l<=44	
Reflections collected	36789	
Independent reflections	3355 [R(int) = 0.059]	
Completeness to theta = 74.706°	99.8%	
Absorption correction	Semi-empirical from equivalents	
Max. and min. transmission	0.98 and 0.79	
Refinement method	Full-matrix least-squares on F^2	
Data / restraints / parameters	3351 / 0 / 217	
Goodness-of-fit on F^2	0.9861	
Final R indices [I>2sigma(I)]	R_1 = 0.0379, wR_2 = 0.0970	
R indices (all data)	R_1 = 0.0458, wR_2 = 0.1068	
Largest diff. peak and hole	0.36 and -0.36 $Å^{-3}$	

Table S3. Crystal data and structure refinement for 9.

CCDC Identification code	CCDC 1991573	
Empirical formula	C23 H19 F2 N1 O2	
Formula Weight	379.41	
Temperature	150 K	
Wavelength	1.54184 Å	
Crystal system	Triclinic	
Space Group	P -1	
Unit cell dimensions	a = 9.0702(6) Å	α = 96.857(5)°
	b = 9.2449(7) Å	β = 107.522(5)°
	c = 12.0135(7) Å	γ = 100.787(6)°
Volume	926.90(12) $Å^3$	
Z	2	
Density (calculated)	1.36 Mg/m^3	
Absorption coefficient	0.832 mm^{-1}	
F(000)	396	
Crystal size	0.05 x 0.05 x 0.30 mm^3	
Theta range for data collection	4.958° to 77.199°	
Index ranges	-11<=h<=10, -11<=k<=10, -10<=l<=15	
Reflections collected	6936	
Independent reflections	3851	
Completeness to theta	99.6%	
Absorption correction	Multi-scan	
Max. and min. transmission	0.96 and 0.86	
Refinement method	Full-matrix least-squares on F^2	
Data / restraints / parameters	3851 / 139 / 281	
Goodness-of-fit on F^2	0.9964	
Final R indices [I>2sigma(I)]	R_1 = 0.0482, wR_2 = 0.1146	
R indices (all data)	R_1 = 0.0398, wR_2 = 0.1046	
Largest diff. peak and hole	0.28 and -0.27 $Å^{-3}$	

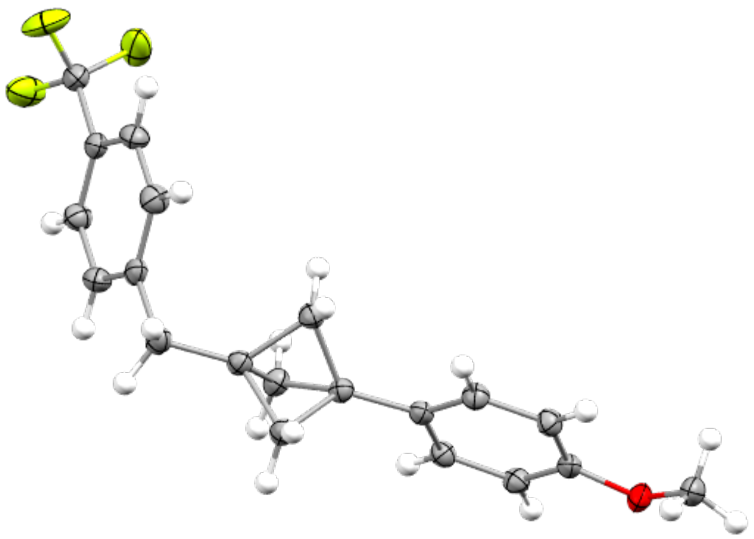

Figure S7: Solid state structure of **2a**. Displacement ellipsoid plots are drawn at 50% probability. Hydrogen atoms have been set to a fixed radius of 0.20 Å for clarity.

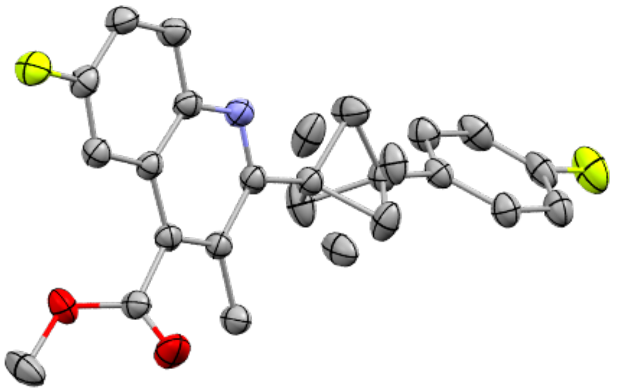

a.

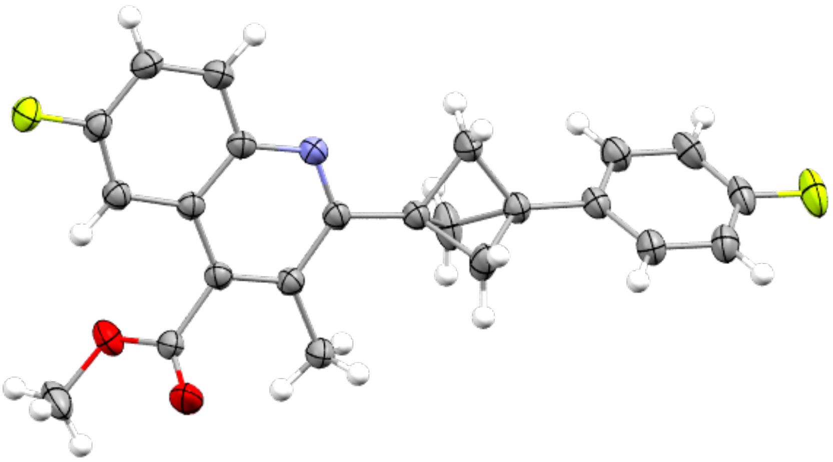

b.

Figure S8: a. Solid state structure of **9**. Disorder in the bicyclo[1.1.1]pentyl CH_2 units are displayed (occupancy = 0.873(3) : 0.127(3)). Displacement ellipsoid plots are drawn at 50% probability. Hydrogen atoms have removed for clarity. **b.** Solid state structure of **9**. Displacement ellipsoid plots are drawn at 50% probability. For clarity, minor disorder in the bicyclo[1.1.1]pentyl CH_2 units has been removed and Hydrogen atoms have been set to a fixed radius of 0.20 Å.

7. Copies of NMR Spectra

tert-Butyl 4-(3-iodobicyclo[1.1.1]pentan-1-yl)piperidine-1-carboxylate, 1b

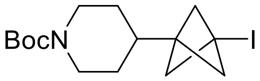

^{1}H NMR (400 MHz, CDCl$_3$)

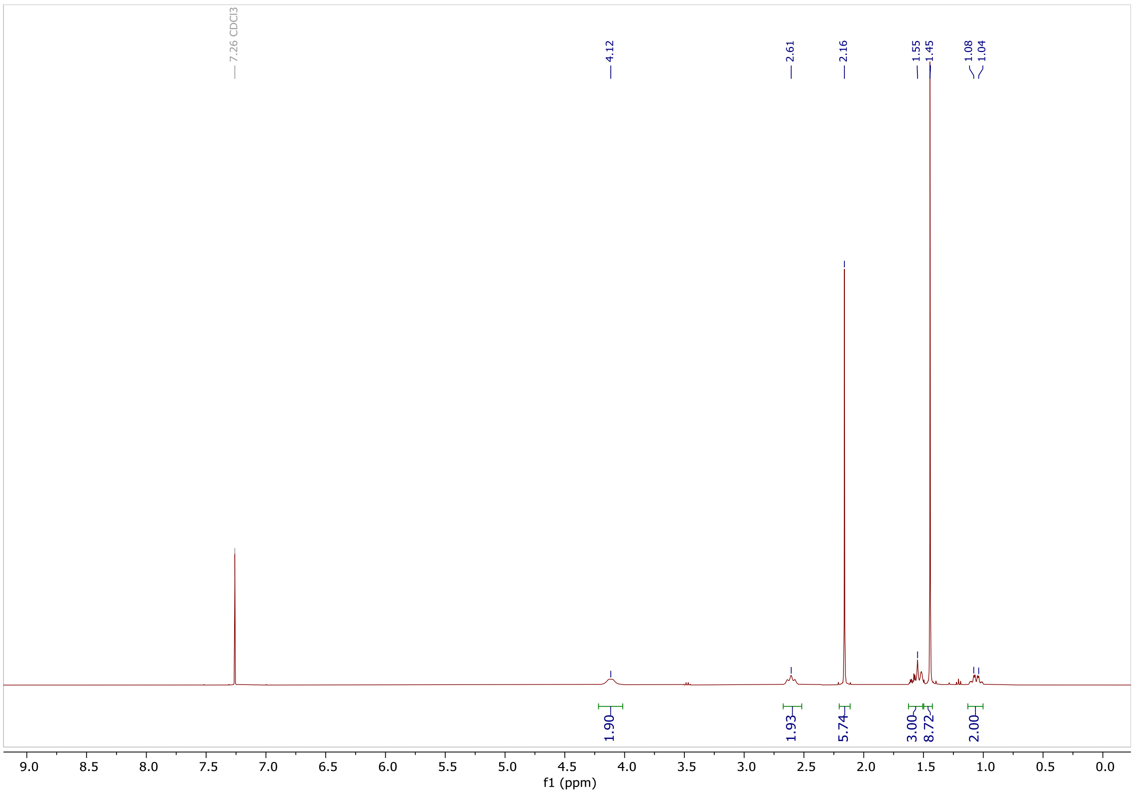

^{13}C NMR (101 MHz, CDCl$_3$)

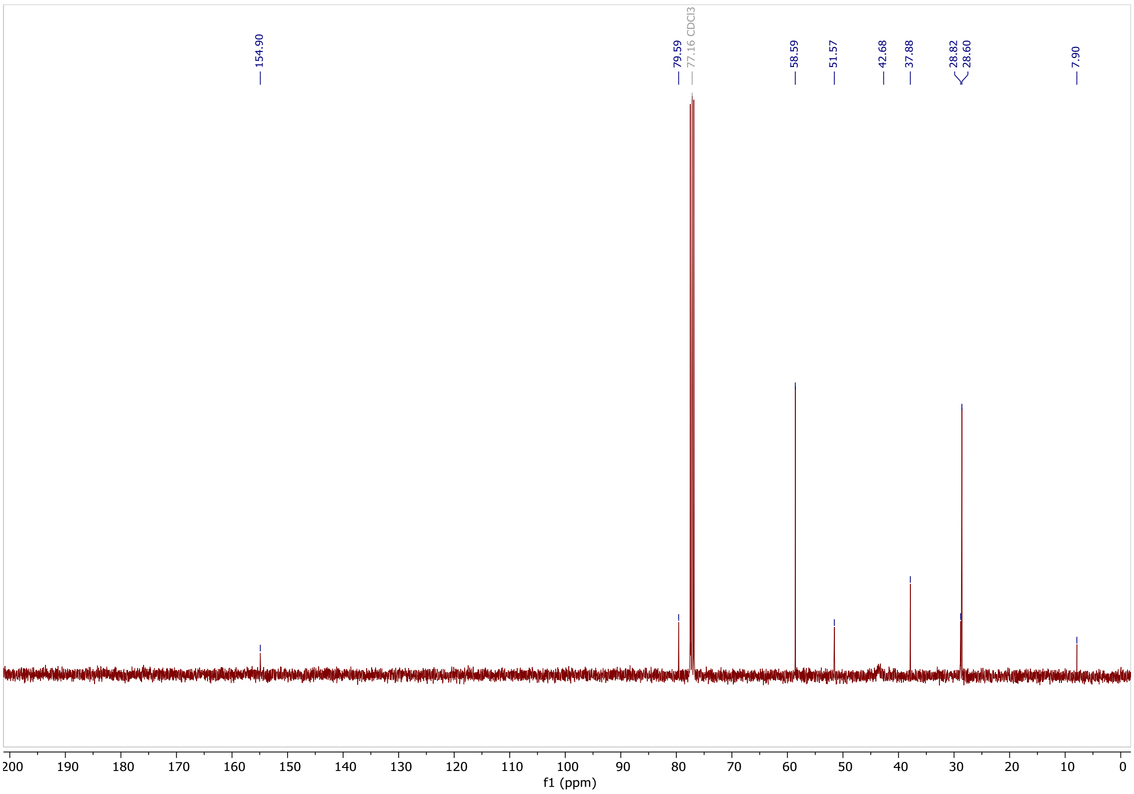

***tert*-Butyl 3-(3-iodobicyclo[1.1.1]pentan-1-yl)azetidine-1-carboxylate, 1c**

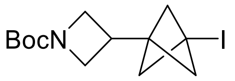

^{1}**H NMR** (400 MHz, $CDCl_3$)

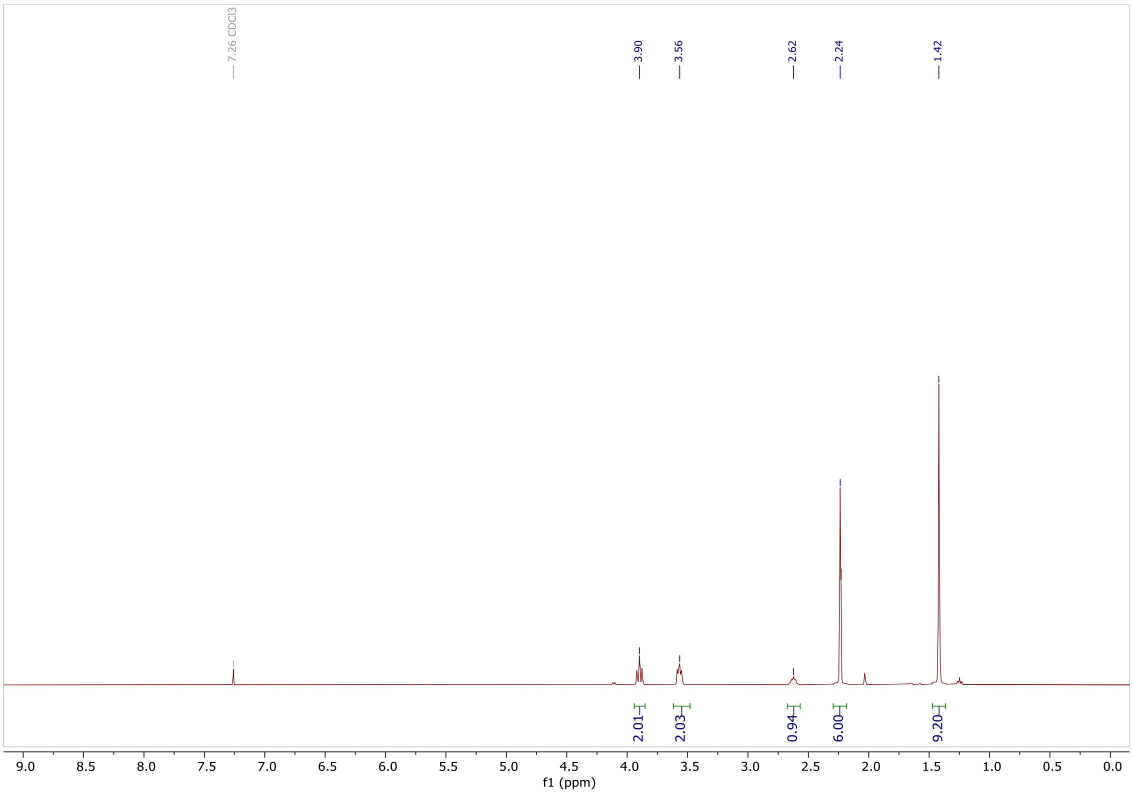

^{13}**C NMR** (101 MHz, $CDCl_3$)

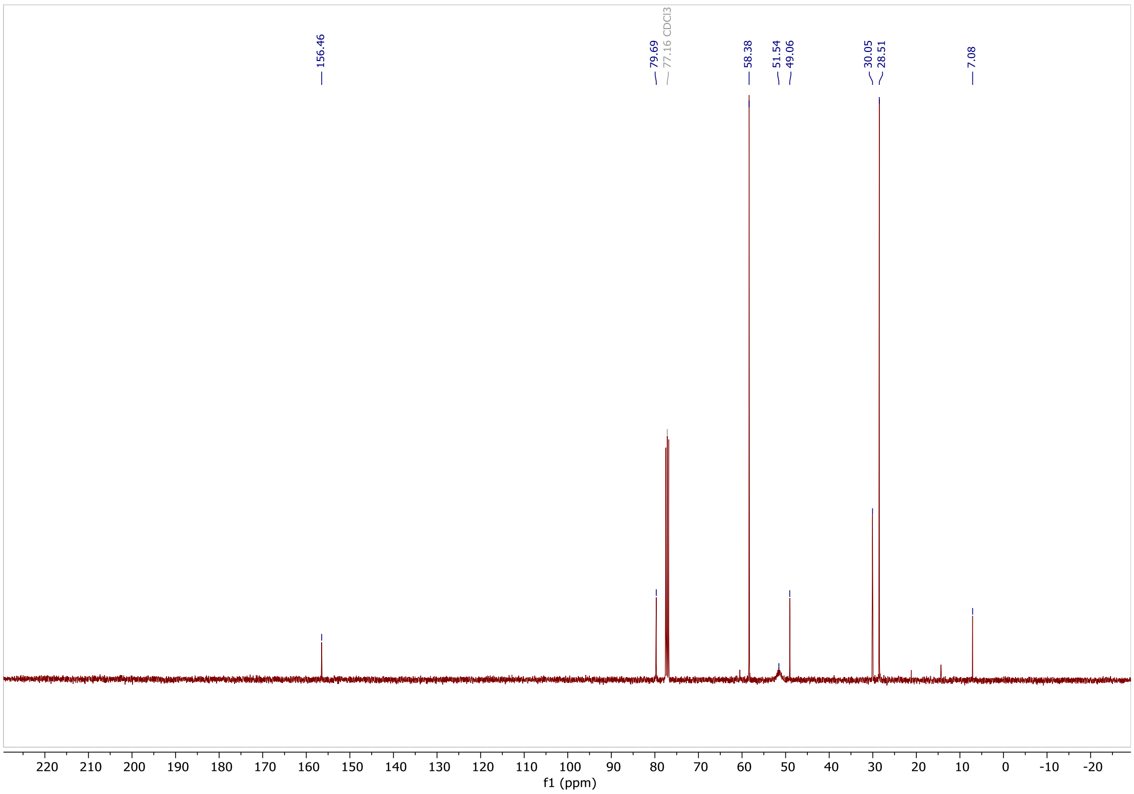

5-Fluoro-1-propionylindoline-2,3-dione, S1

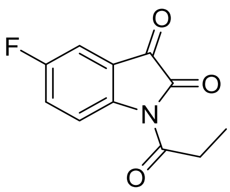

^{1}H NMR (400 MHz, DMSO-d_6)

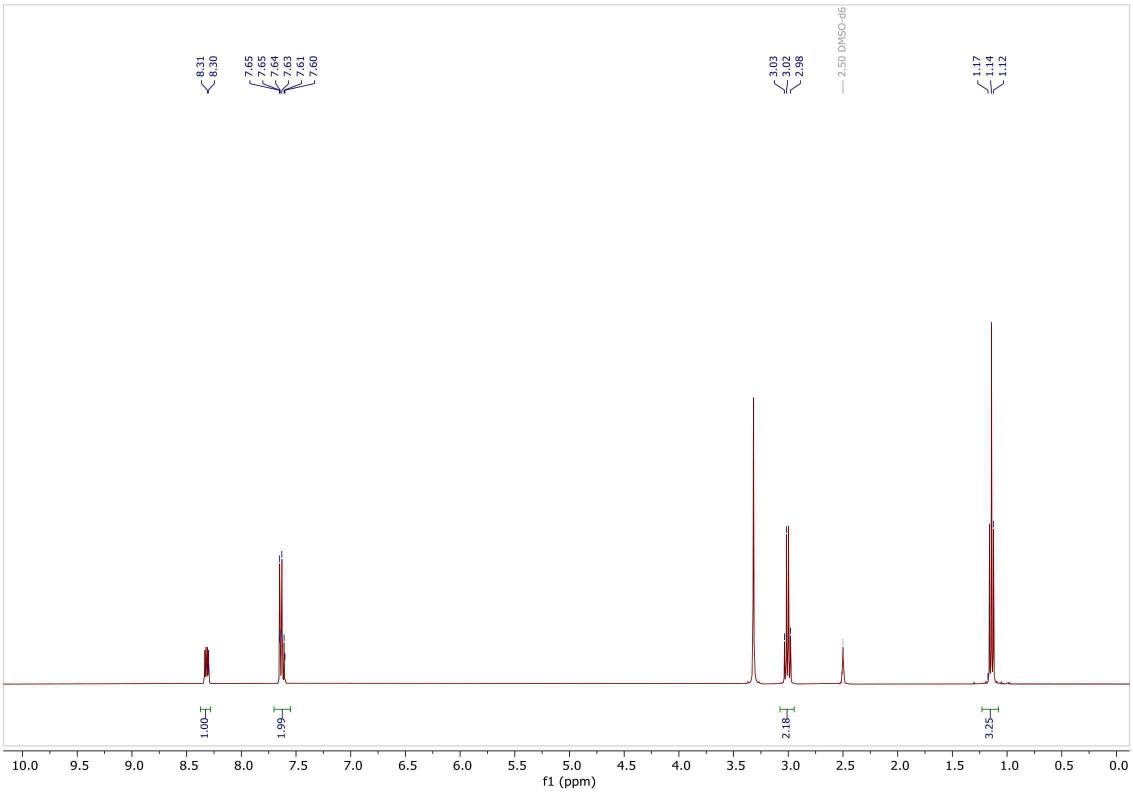

^{13}C NMR (101 MHz, DMSO-d_6)

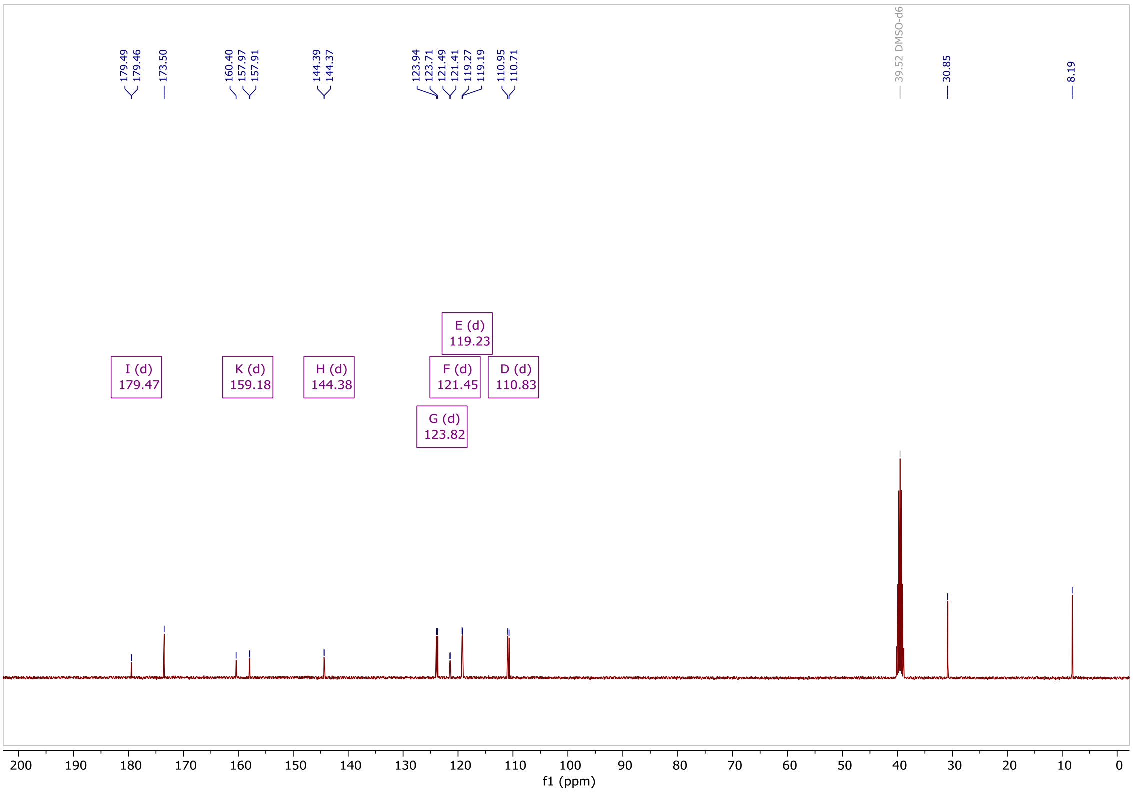

^{19}F NMR (376 MHz, DMSO-d_6)

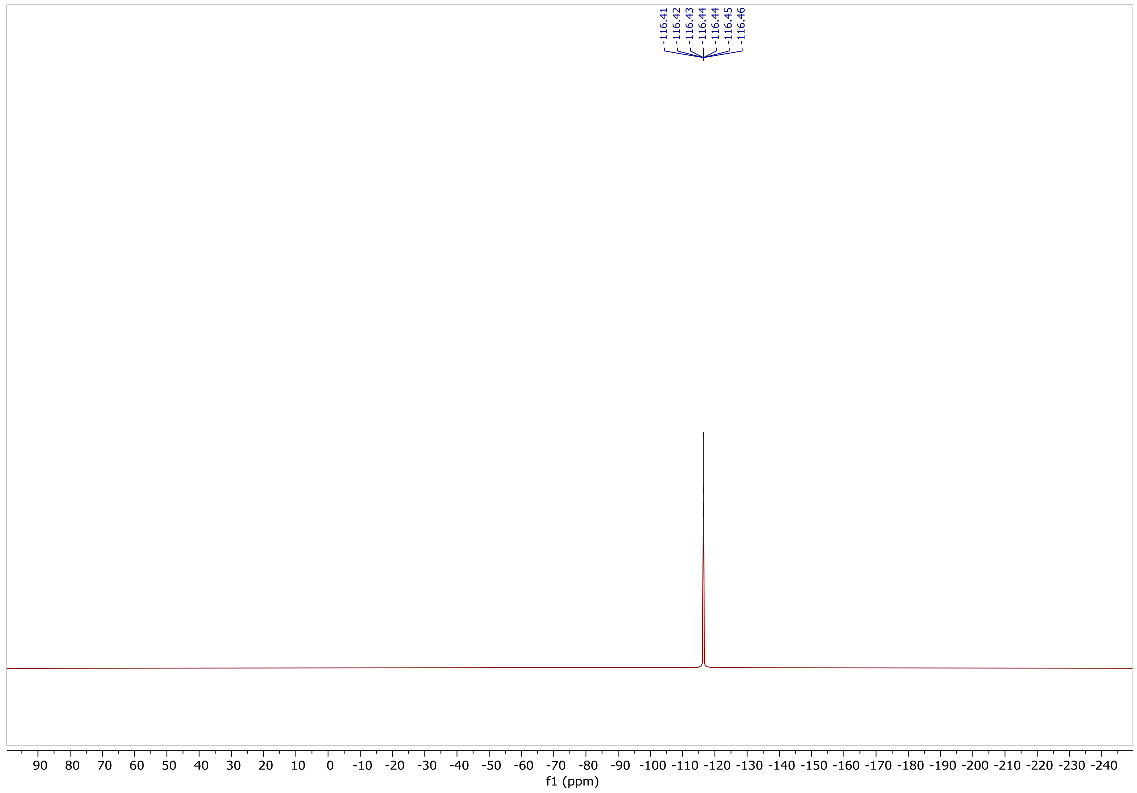

6-Fluoro-2-hydroxy-3-methylquinoline-4-carboxylic acid, S2

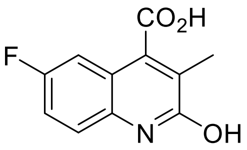

^{1}H NMR (400 MHz, DMSO-*d*$_6$)

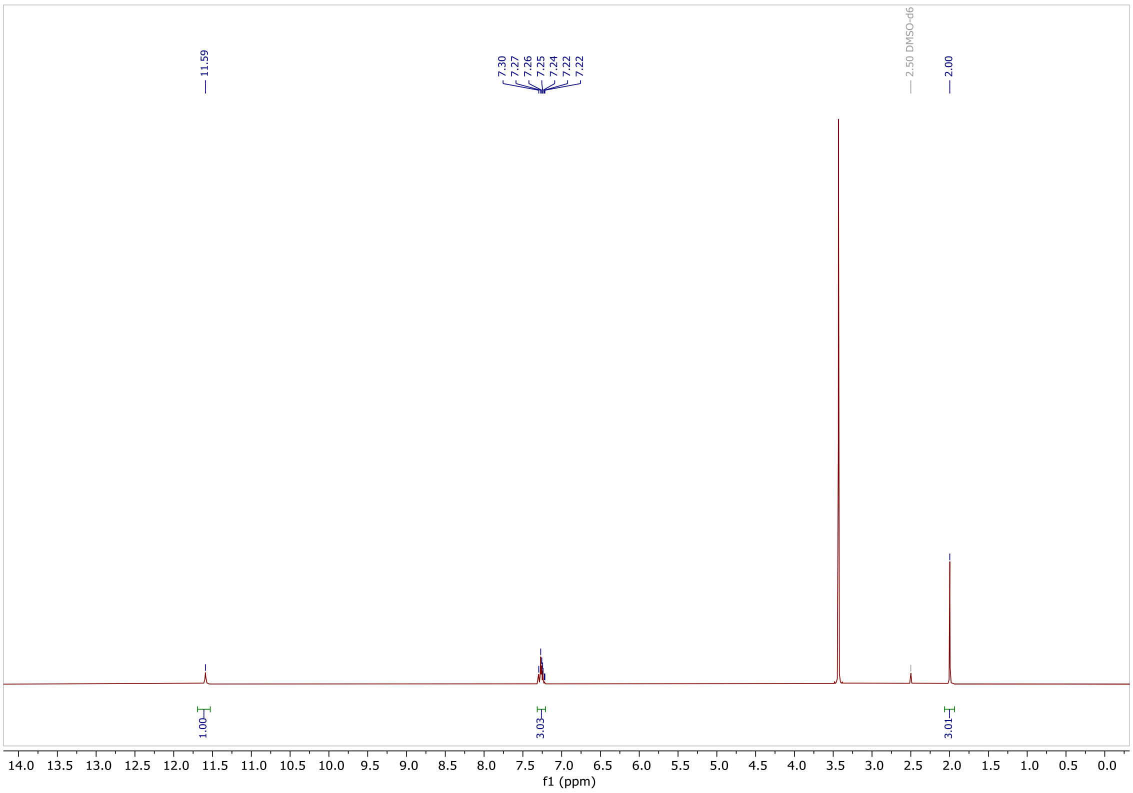

^{13}C NMR (101 MHz, DMSO-*d*$_6$)

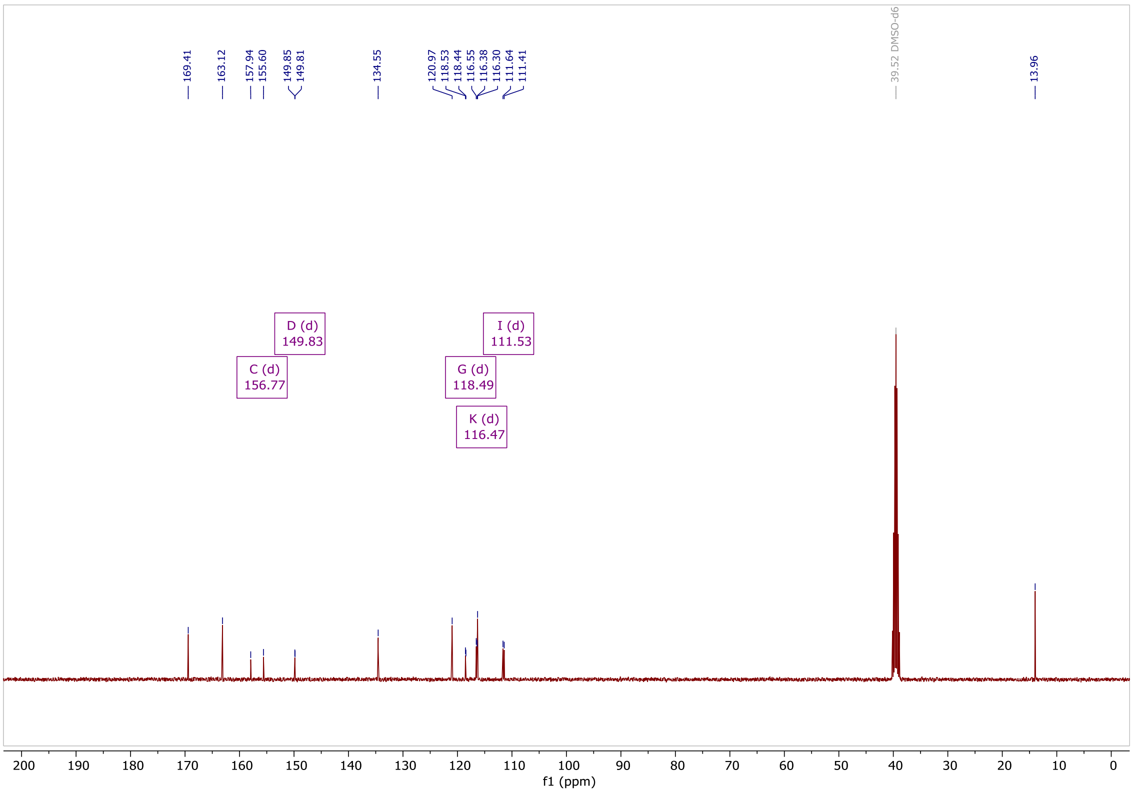

^{19}F NMR (376 MHz, DMSO-d_6)

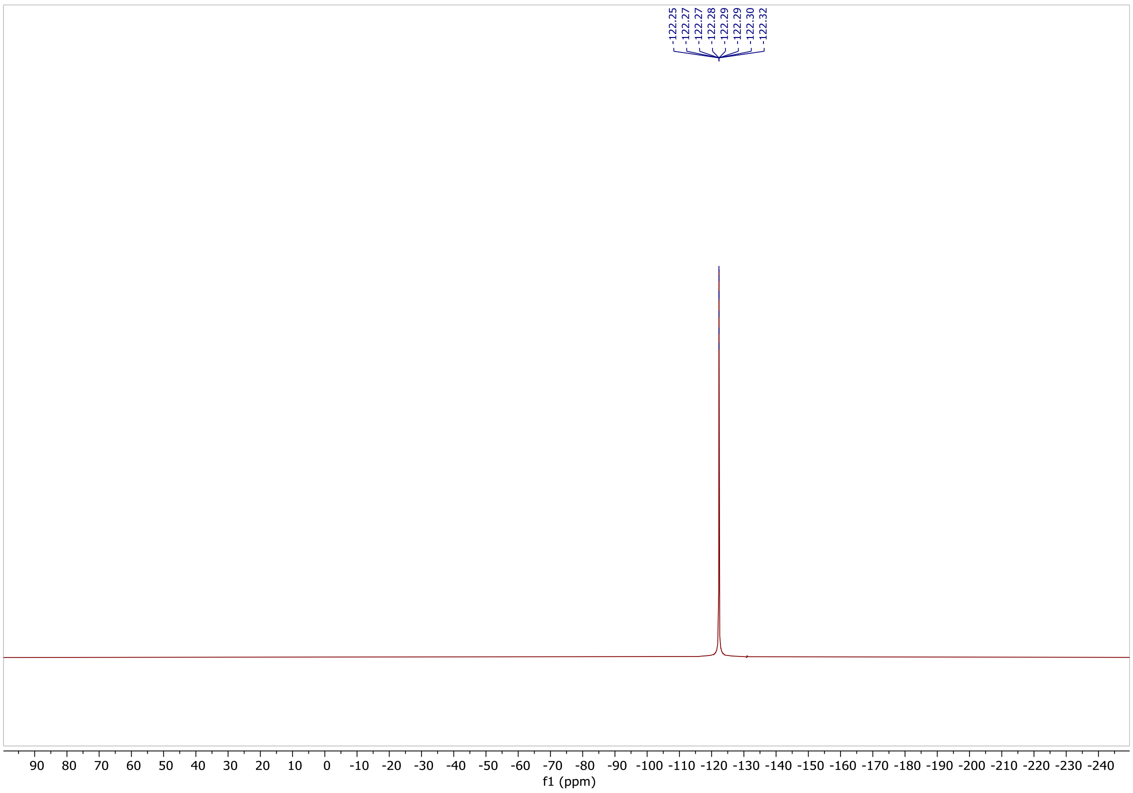

Methyl 2-bromo-6-fluoro-3-methylquinoline-4-carboxylate, S3

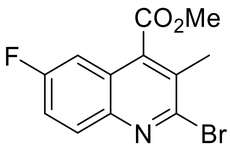

[1]**H NMR** (400 MHz, DMSO-d_6)

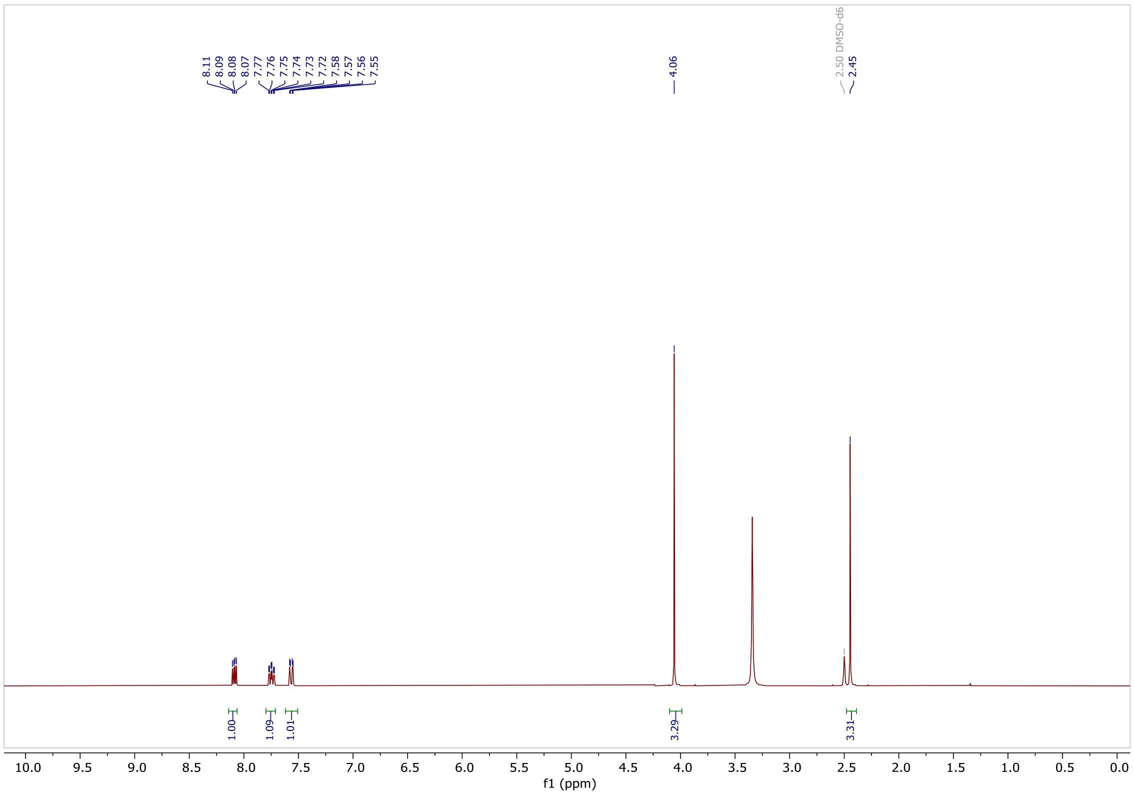

[13]**C NMR** (101 MHz, DMSO-d_6)

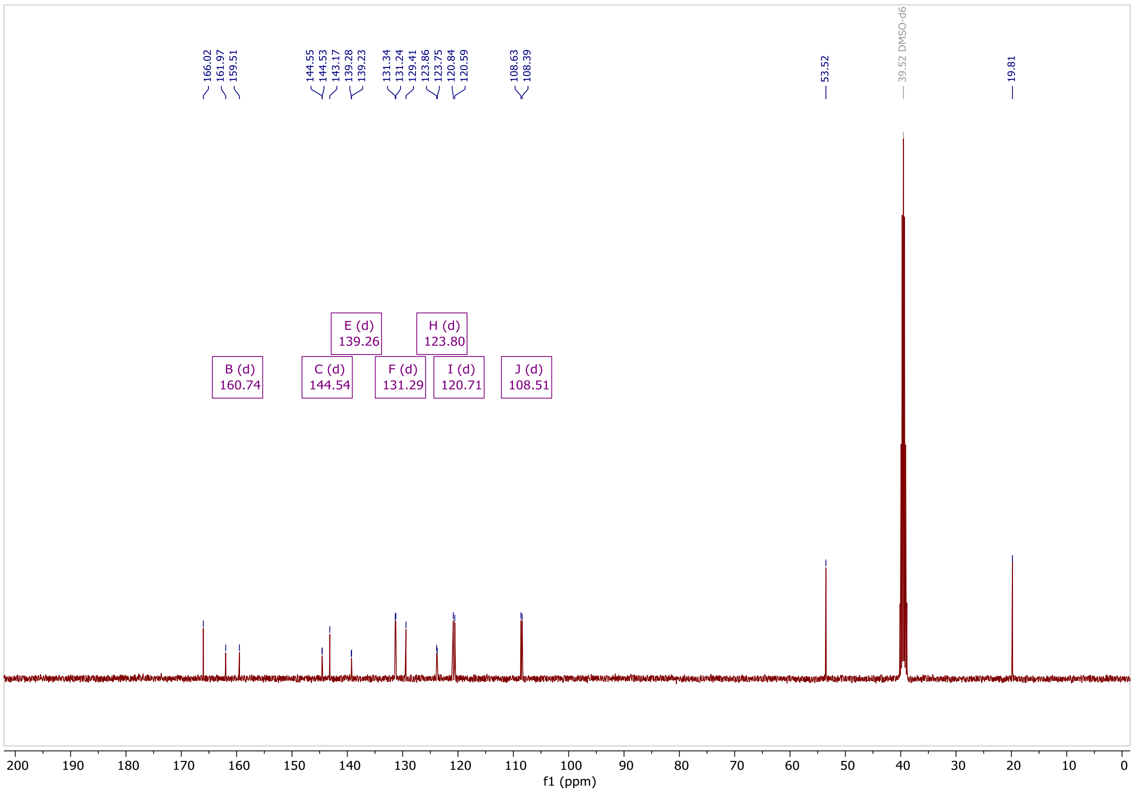

^{19}F NMR (376 MHz, DMSO-d_6)

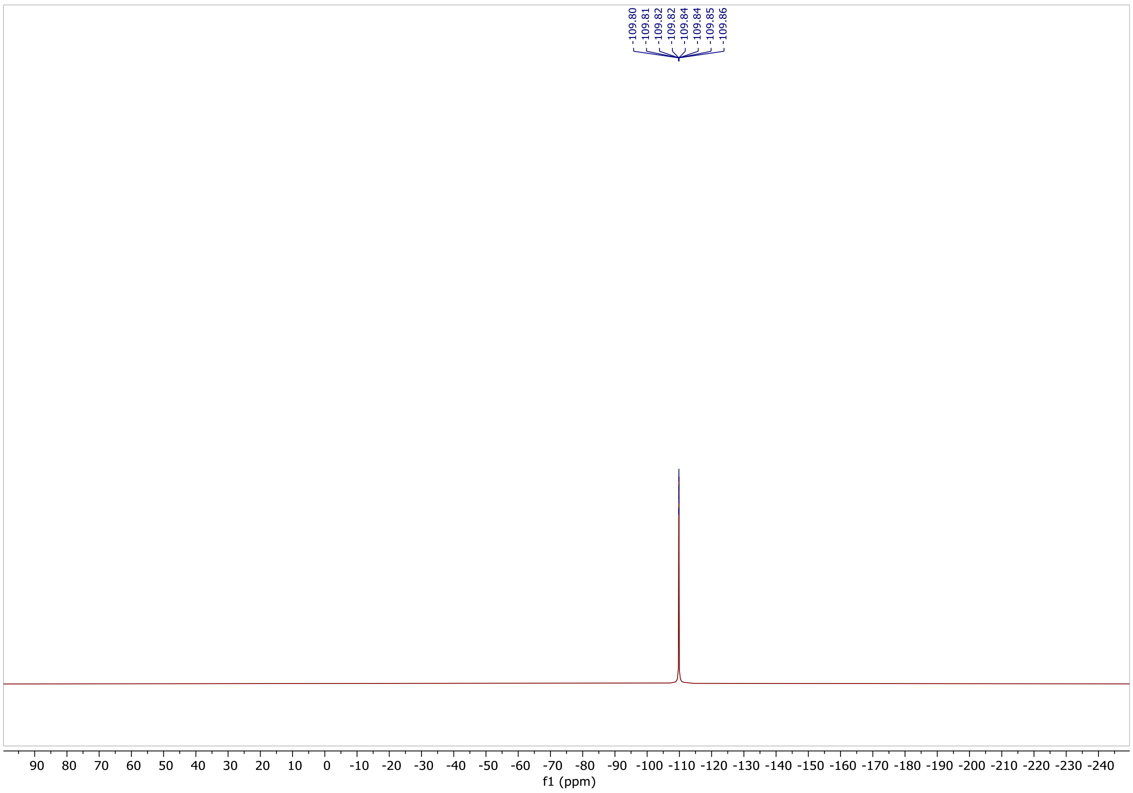

Methyl 6-fluoro-2-iodo-3-methylquinoline-4-carboxylate, 7

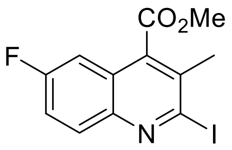

^{1}H NMR (400 MHz, $CDCl_3$)

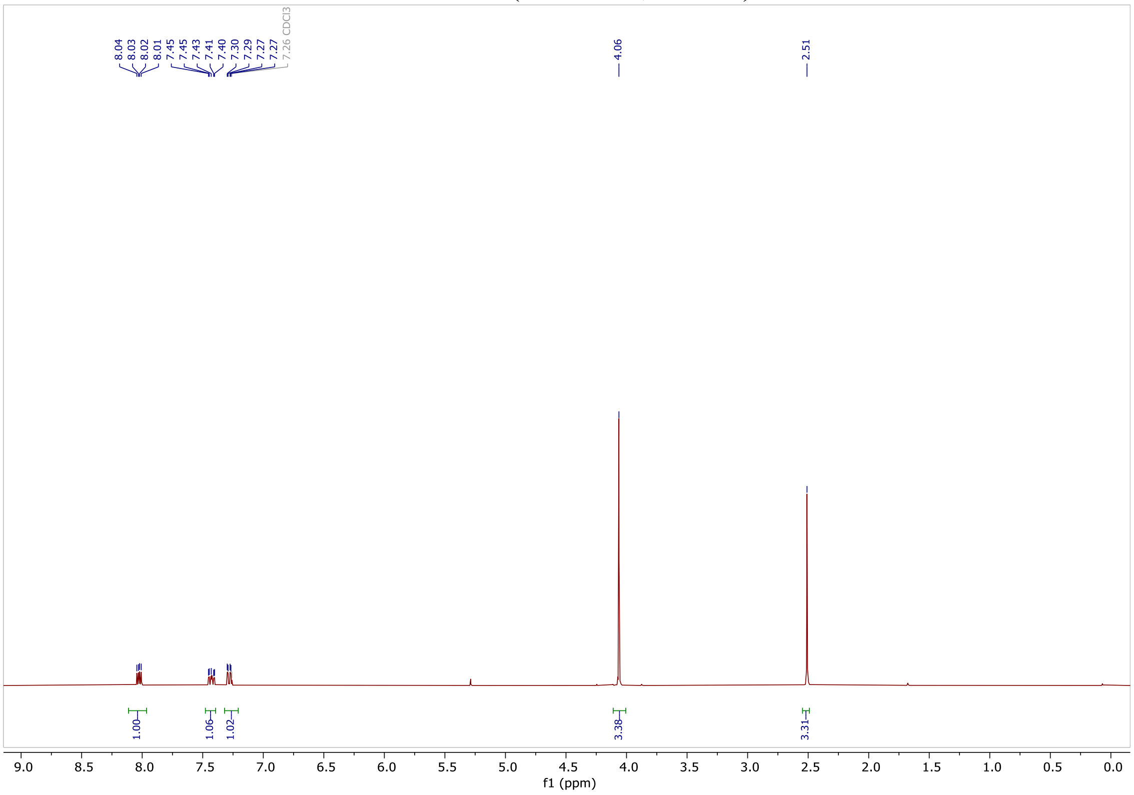

^{13}C NMR (101 MHz, $CDCl_3$)

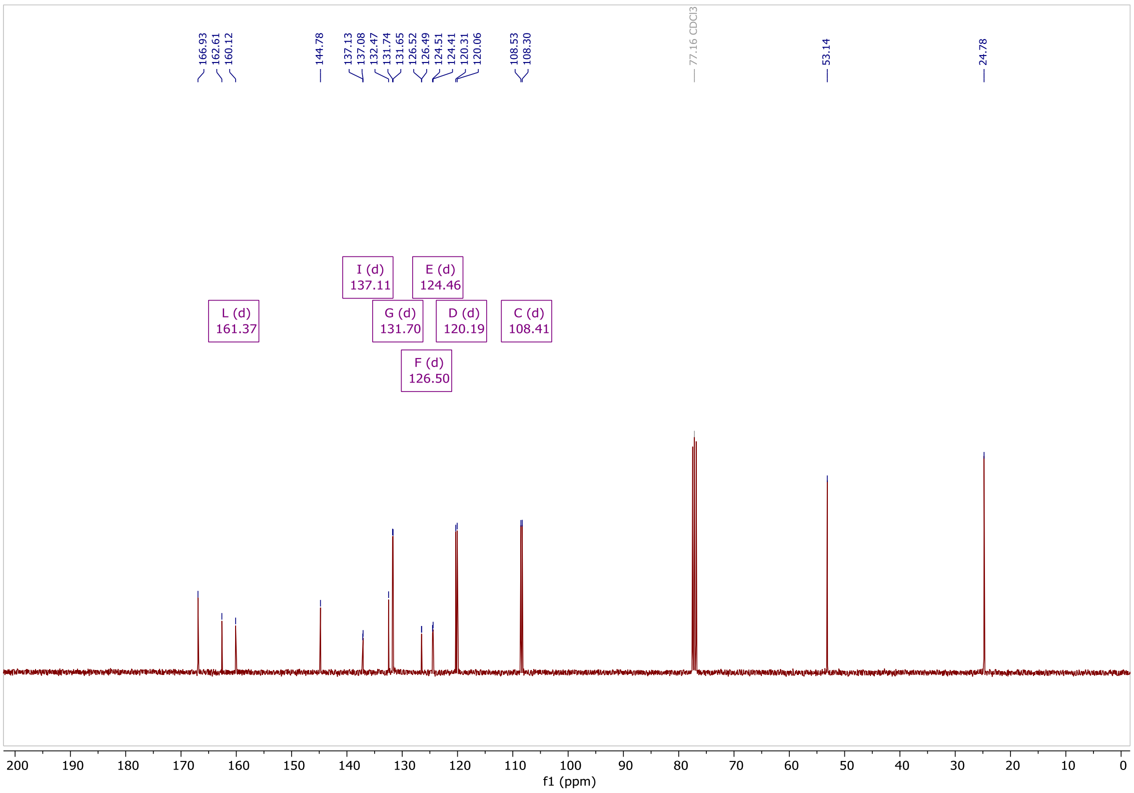

^{19}F NMR (376 MHz, $CDCl_3$)

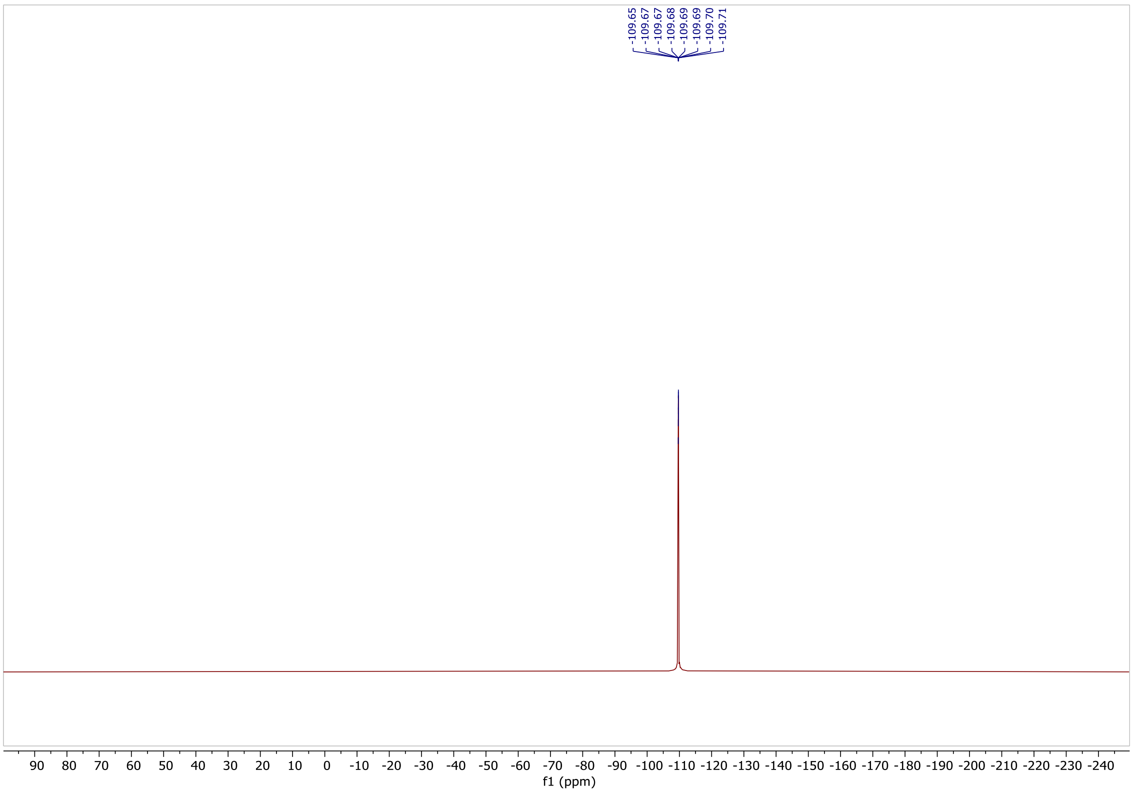

Methyl 6-fluoro-2-(3-iodobicyclo[1.1.1]pentan-1-yl)-3-methylquinoline-4-carboxylate, 1i

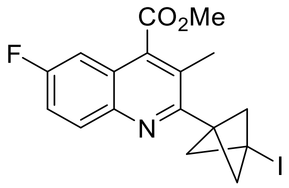

^{1}H NMR (400 MHz, $CDCl_3$)

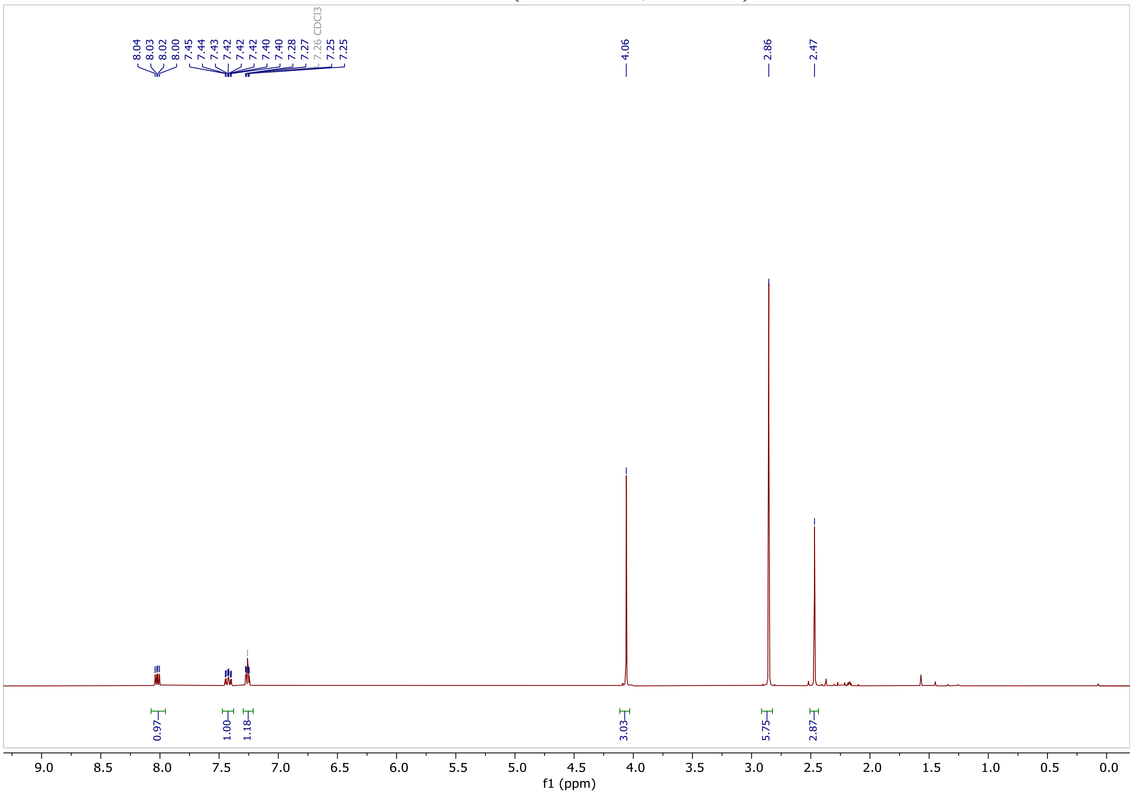

^{13}C NMR (101 MHz, $CDCl_3$)

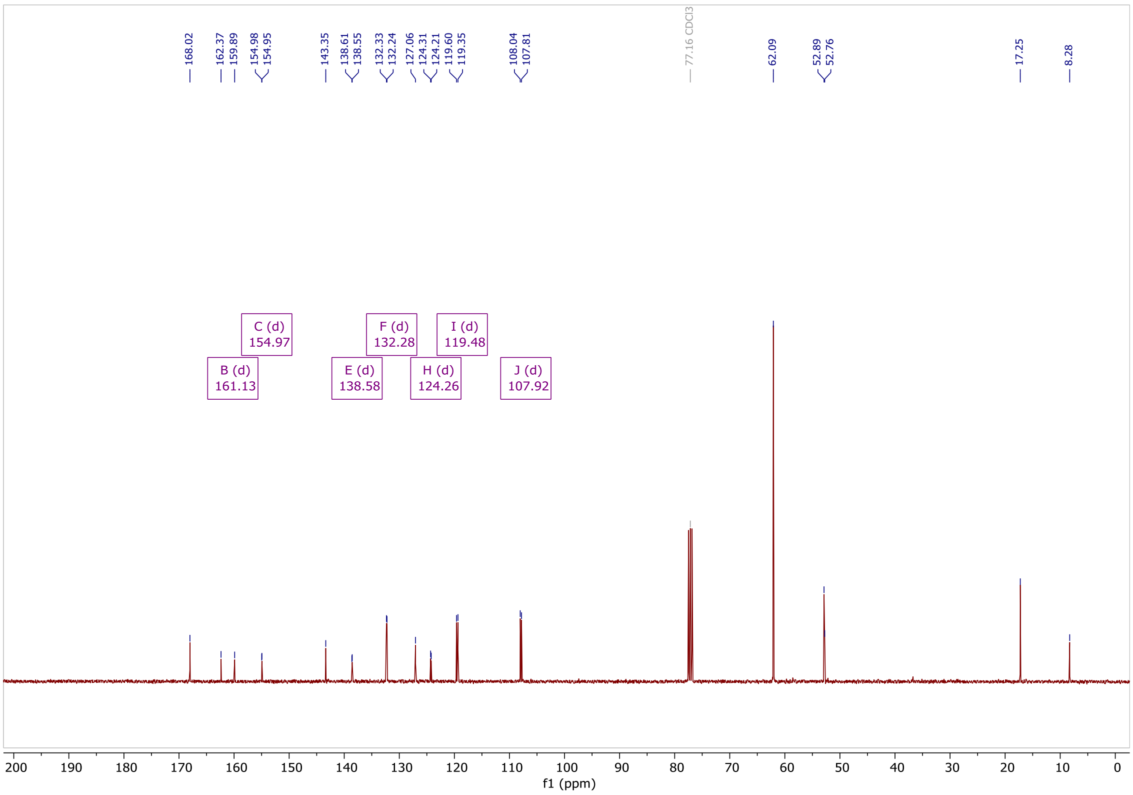

^{19}**F NMR** (376 MHz, $CDCl_3$)

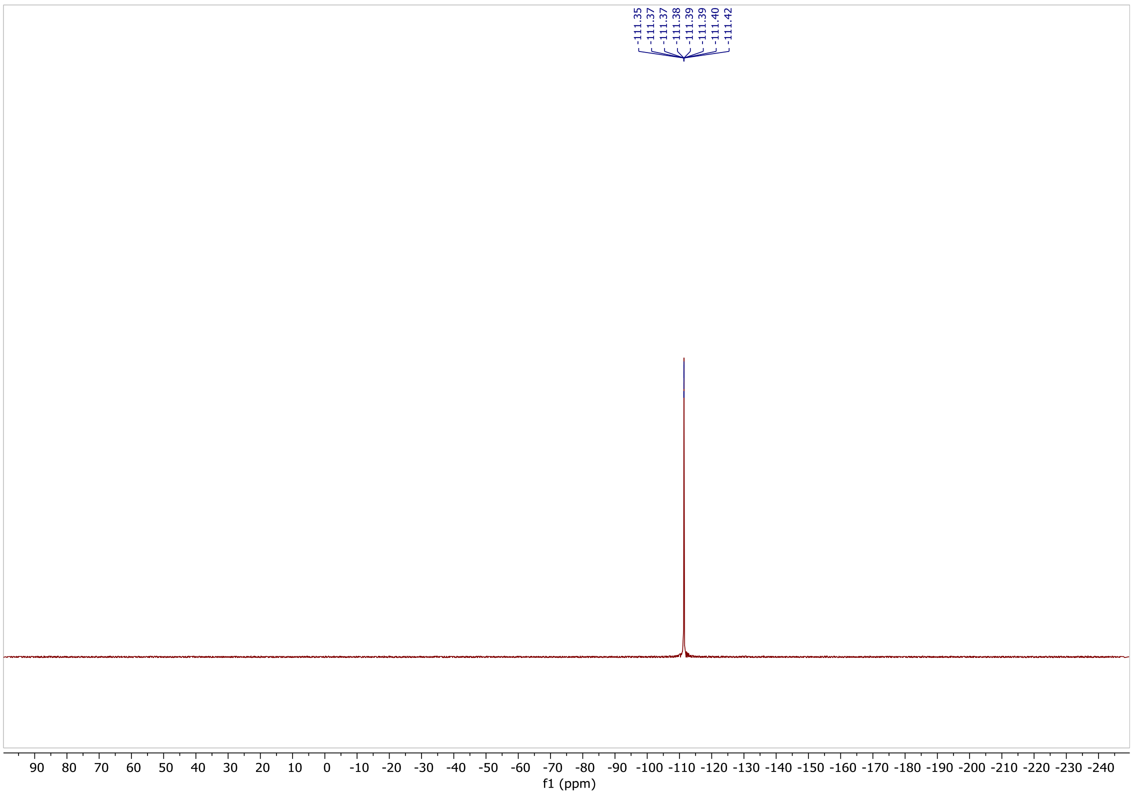

Ethyl 2-(3-iodobicyclo[1.1.1]pentan-1-yl)propanoate, 1k

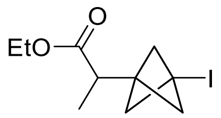

^{1}H NMR (400 MHz, $CDCl_3$)

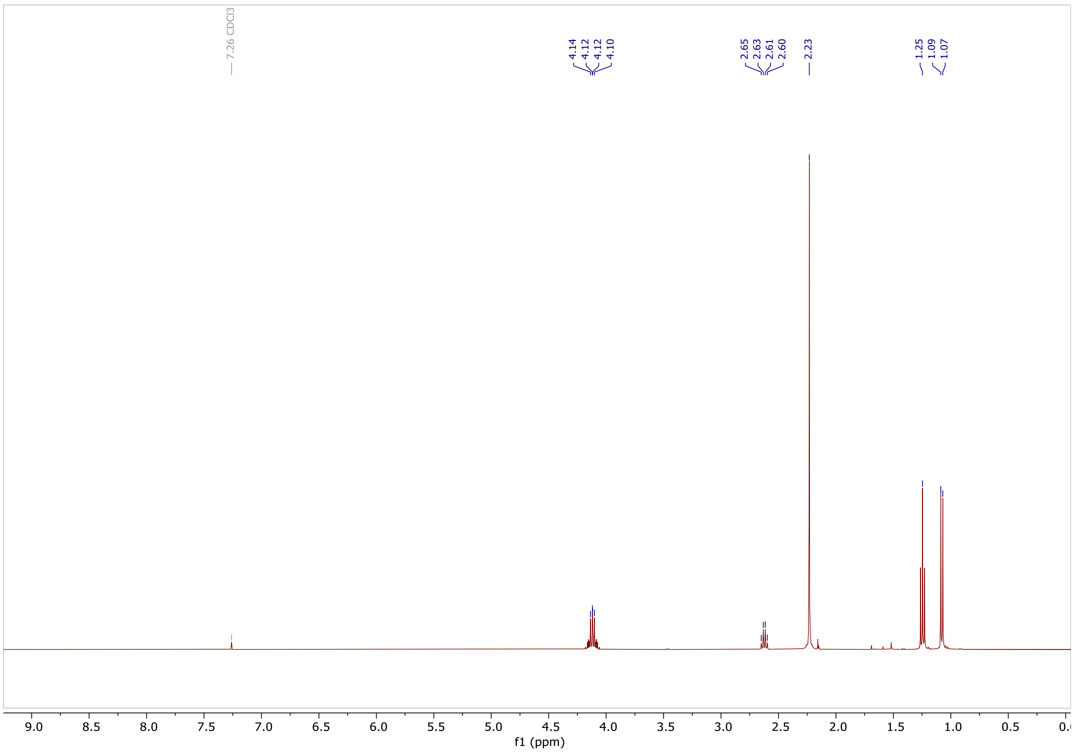

^{13}C NMR (101 MHz, $CDCl_3$)

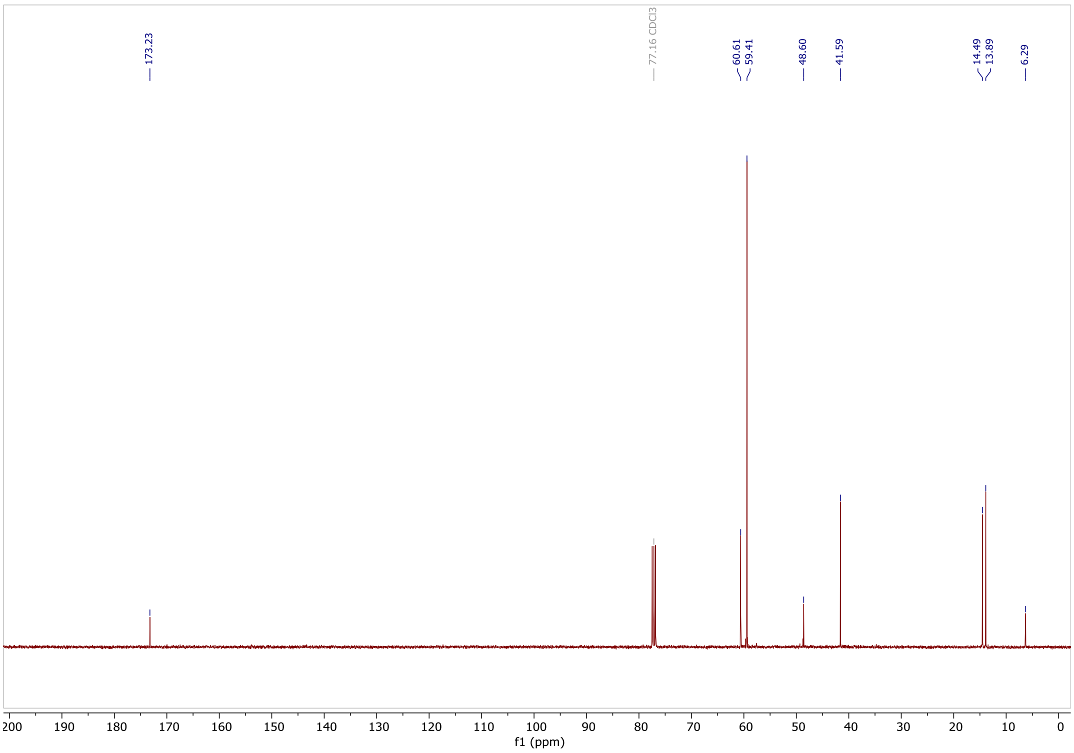

***tert*-Butyl 4-(3-methylenecyclobutylidene)piperidine-1-carboxylate, 3**

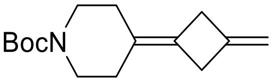

^{1}H NMR (400 MHz, $CDCl_3$)

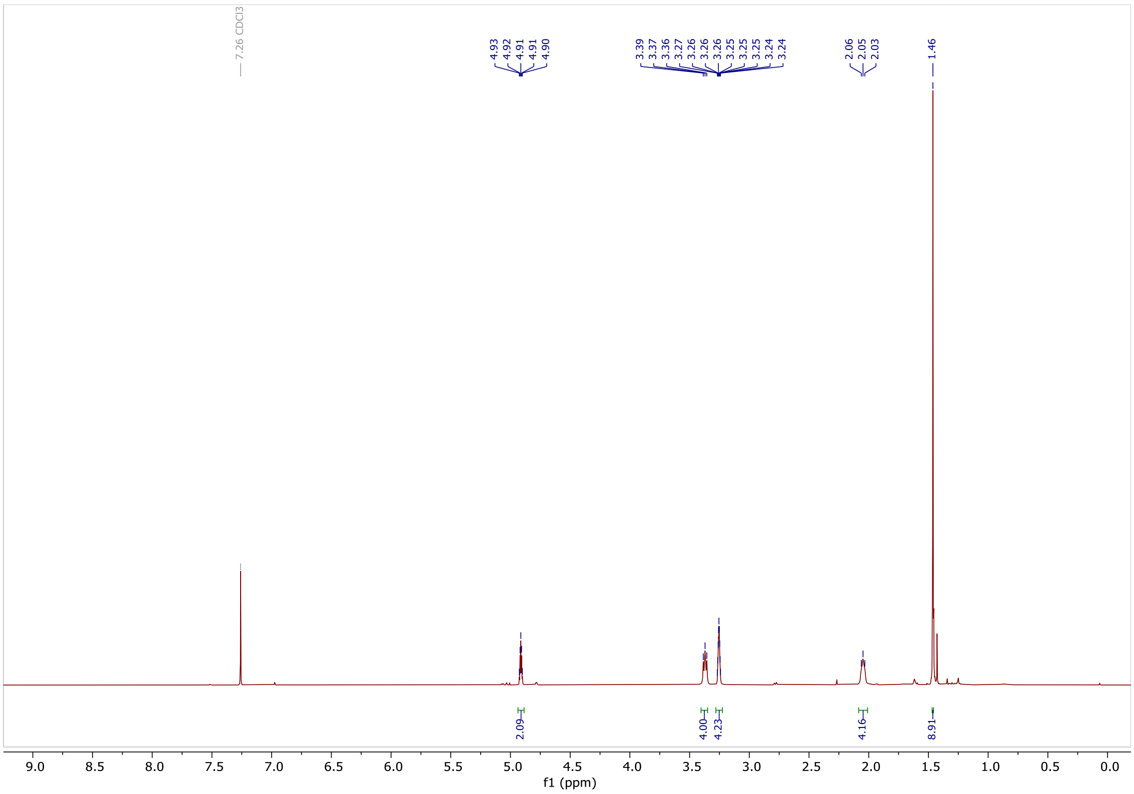

^{13}C NMR (101 MHz, $CDCl_3$)

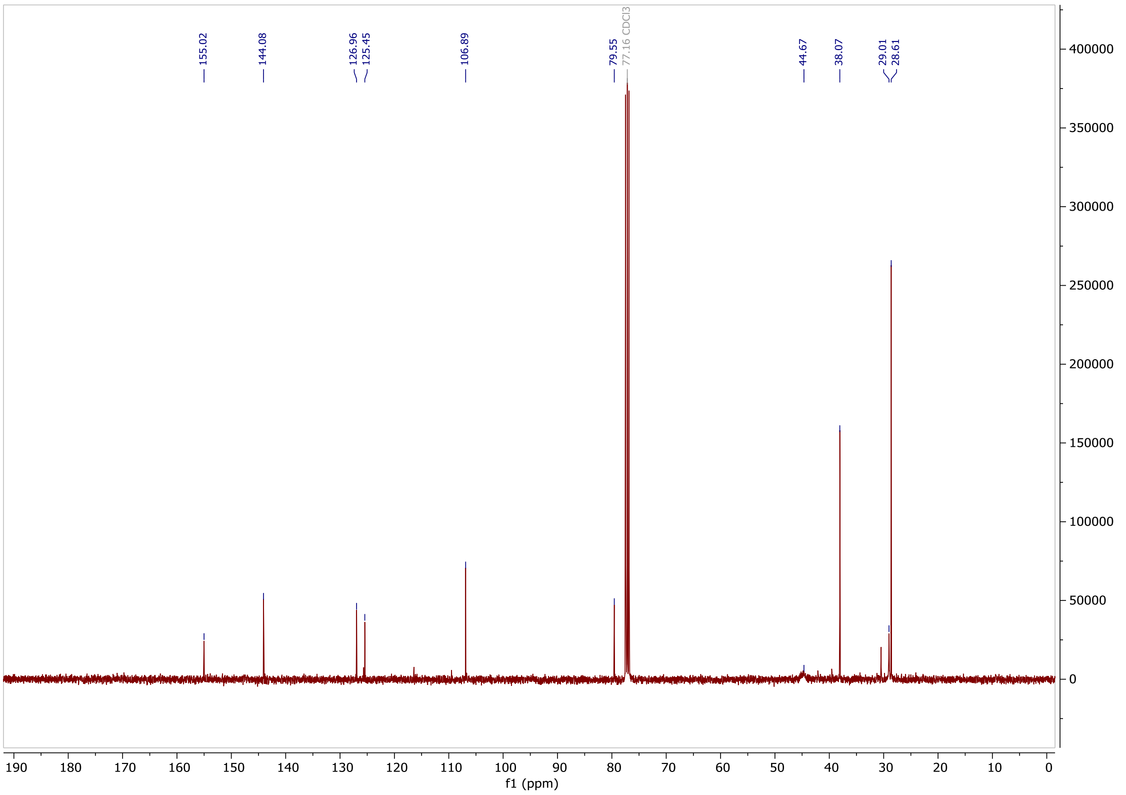

1-(4-Methoxyphenyl)-3-(4-(trifluoromethyl)benzyl)bicyclo[1.1.1]pentane, 2a

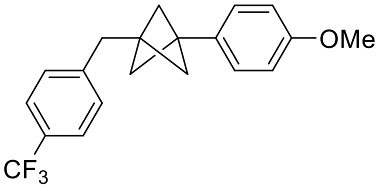

^{1}H NMR (400 MHz, $CDCl_3$)

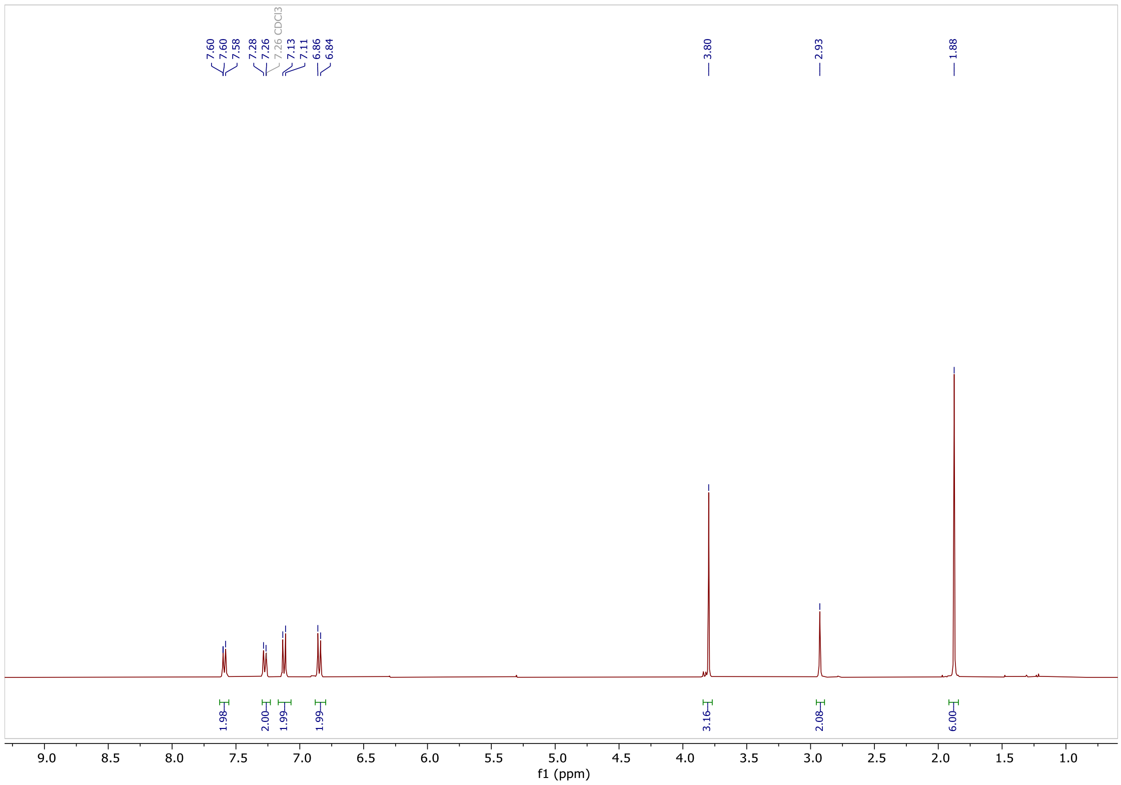

^{13}C NMR (101 MHz, $CDCl_3$)

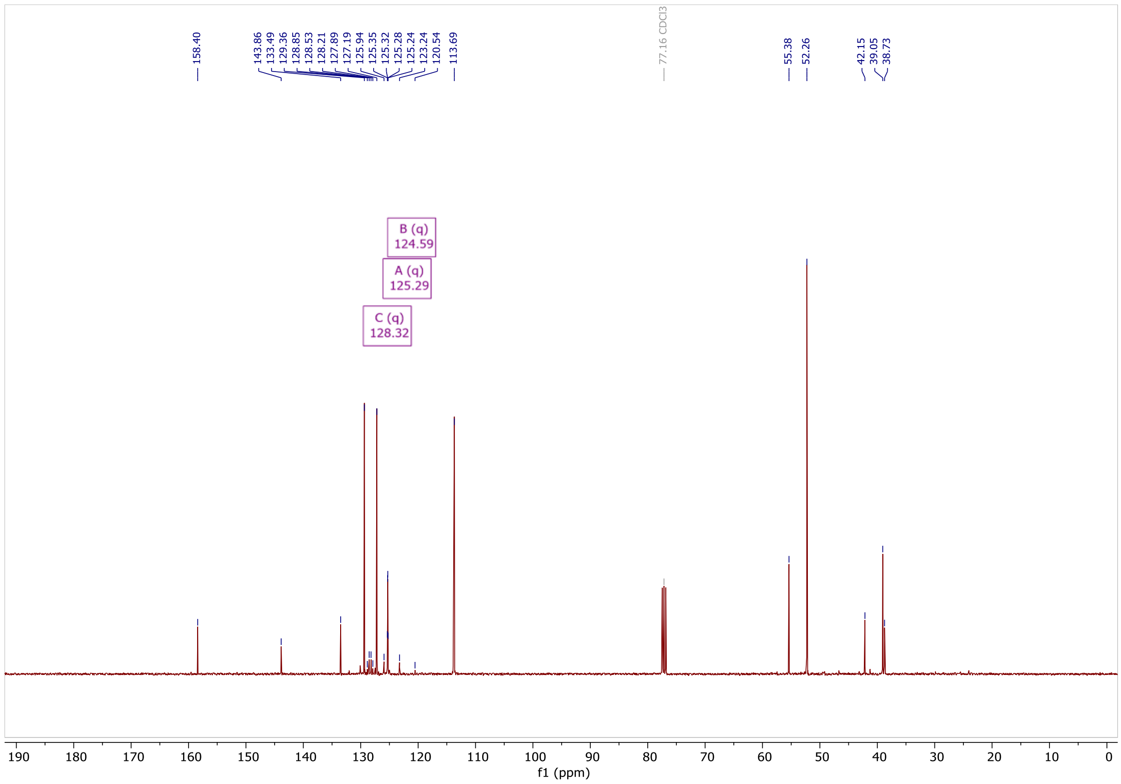

1-(4-(Trifluoromethoxy)phenyl)-3-(4-(trifluoromethyl)benzyl)bicyclo[1.1.1]pentane, 2b

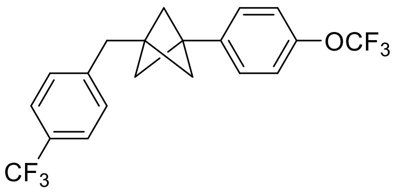

^{1}H NMR (400 MHz, CD_3OD)

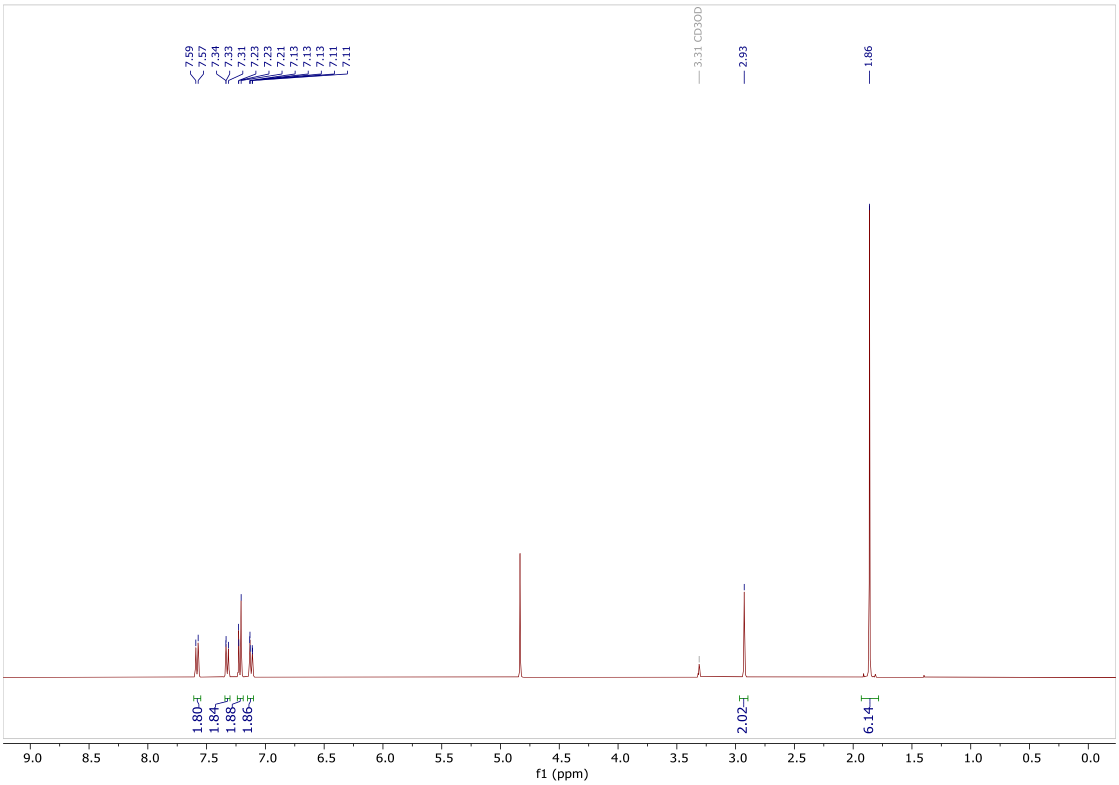

^{13}C NMR (101 MHz, CD_3OD)

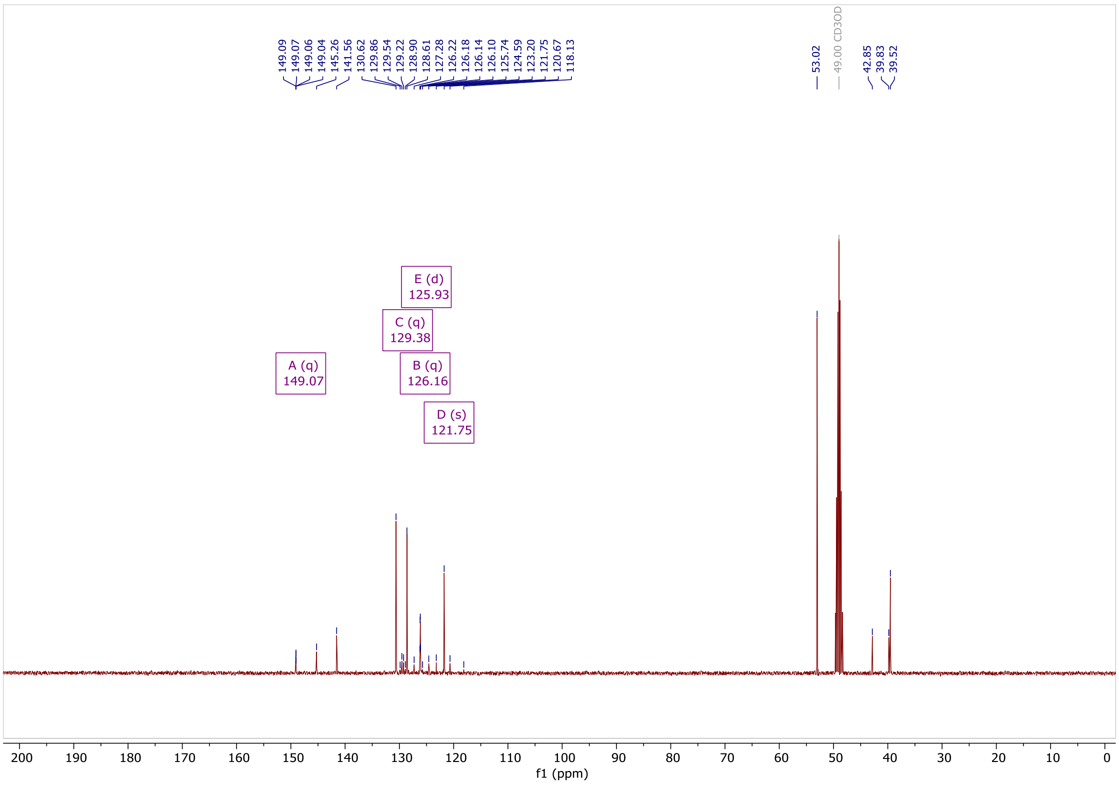

^{19}F NMR (376 MHz, CD_3OD)

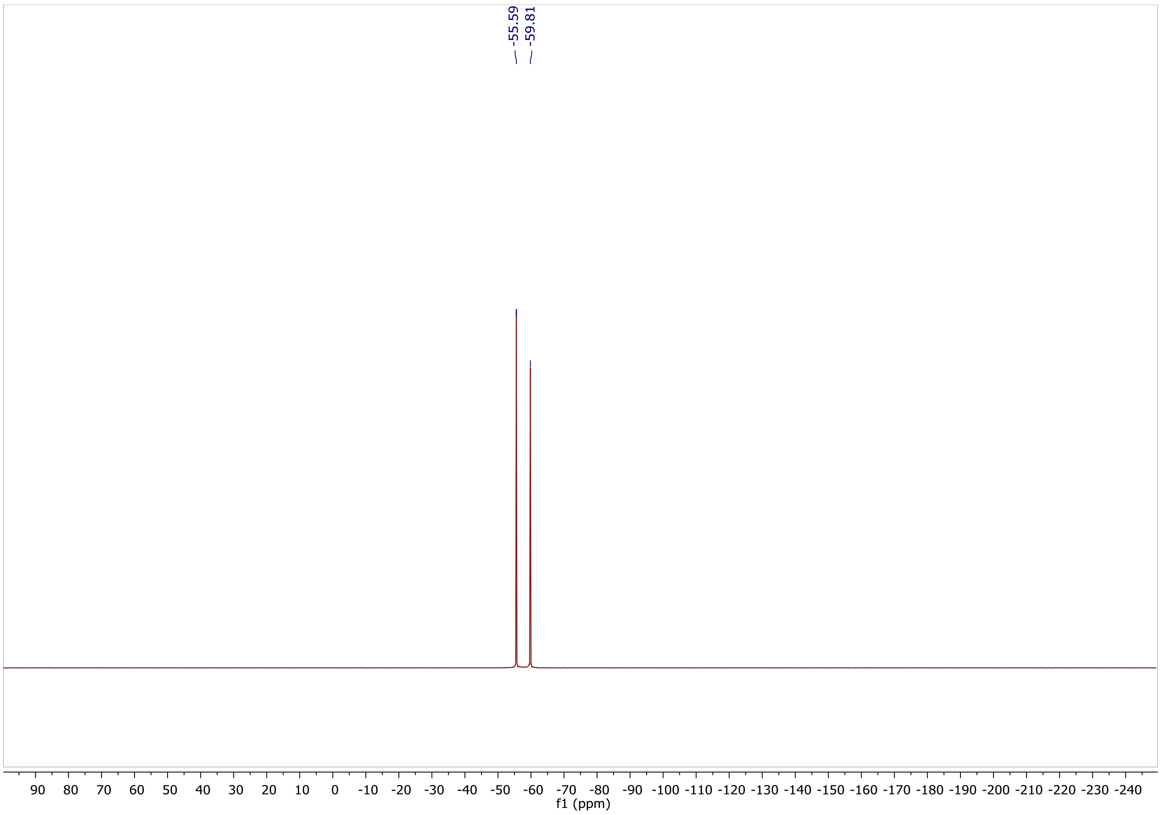

N,N-Dimethyl-4-(3-(4-(trifluoromethyl)benzyl)bicyclo[1.1.1]pentan-1-yl)aniline, 2c

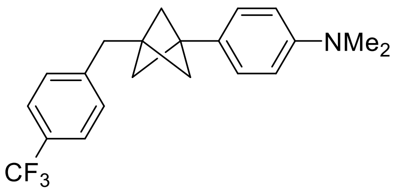

^{1}H NMR (400 MHz, $CDCl_3$)

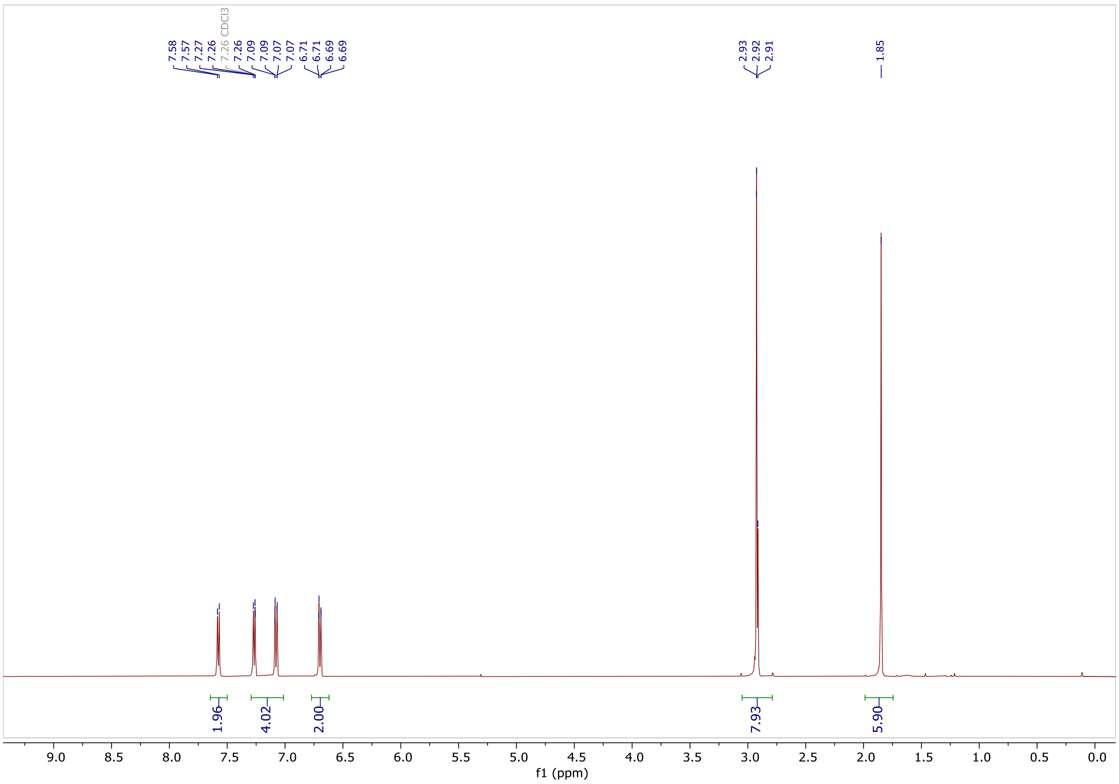

^{13}C NMR (101 MHz, $CDCl_3$)

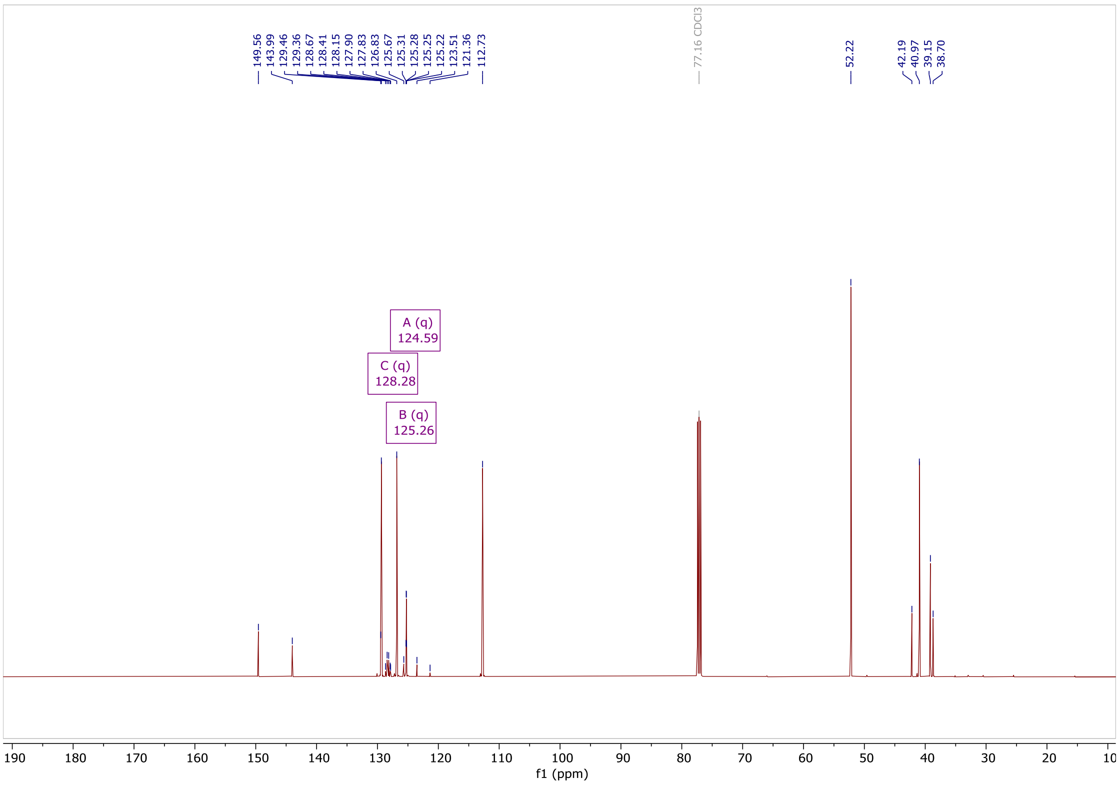

^{19}F NMR (376 MHz, $CDCl_3$)

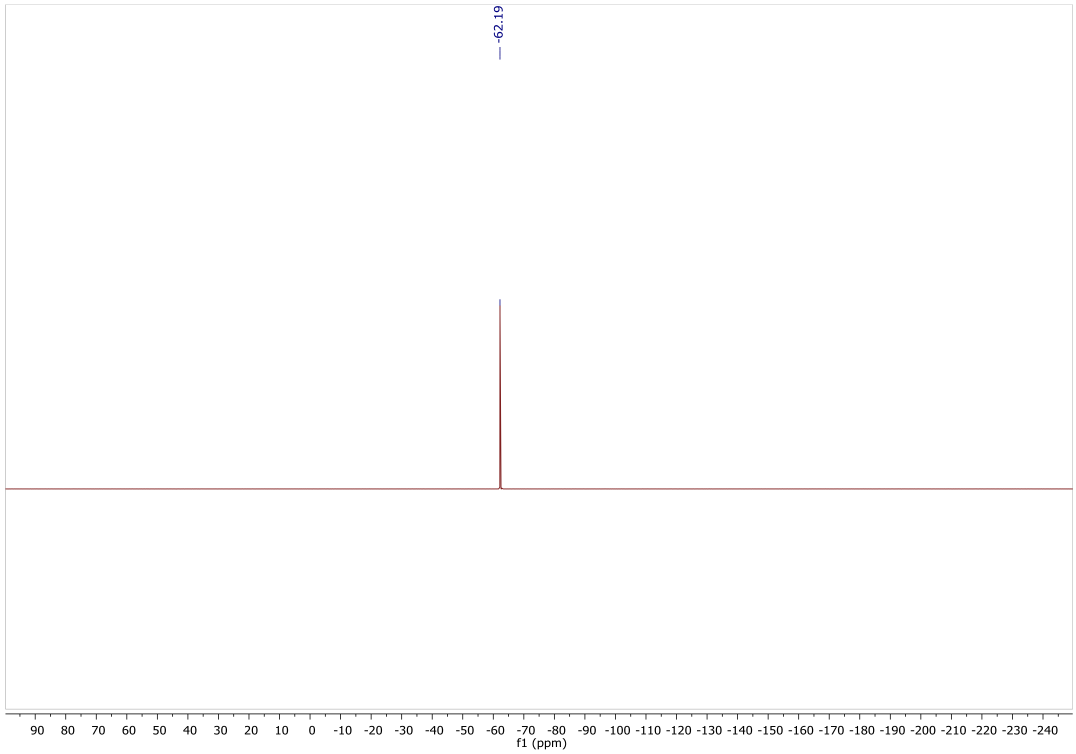

1-(4-fluorophenyl)-3-(4-(trifluoromethyl)benzyl)bicyclo[1.1.1]pentane, 2d

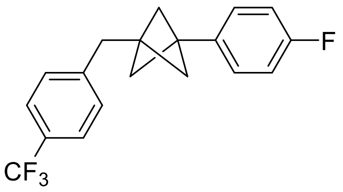

^{1}H NMR (400 MHz, $CDCl_3$)

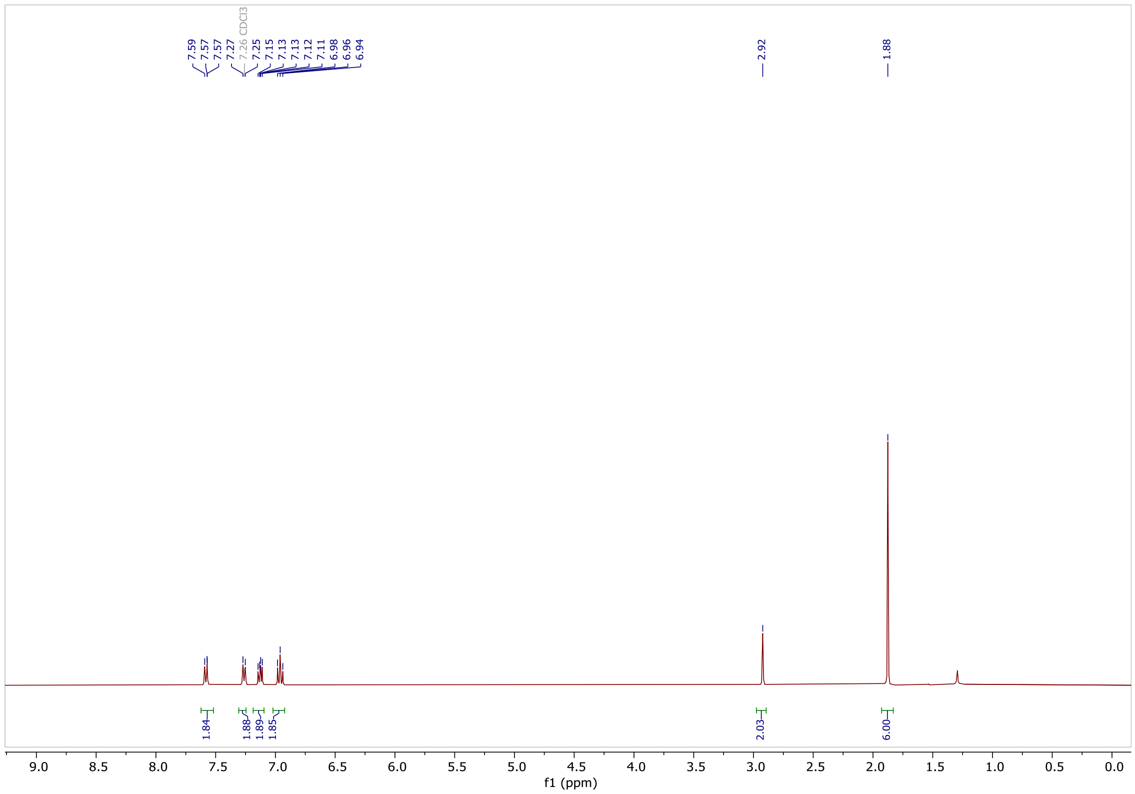

^{13}C NMR (101 MHz, $CDCl_3$)

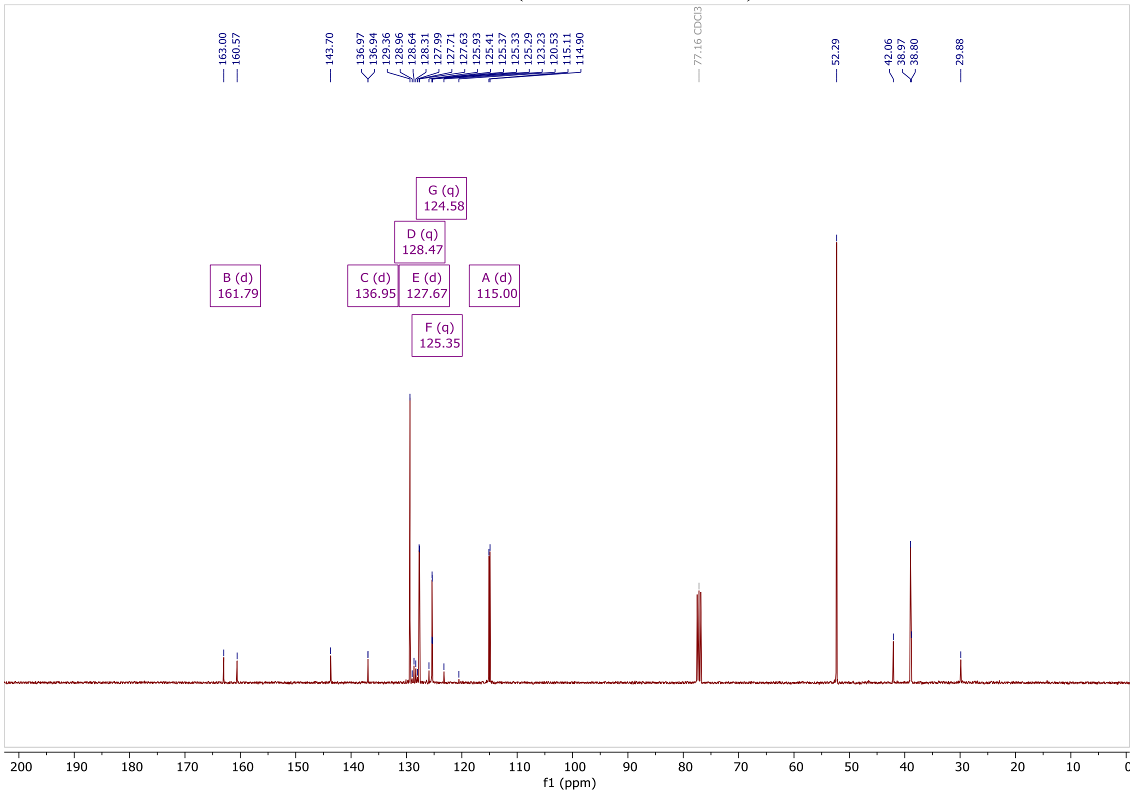

^{19}F NMR (376 MHz, $CDCl_3$)

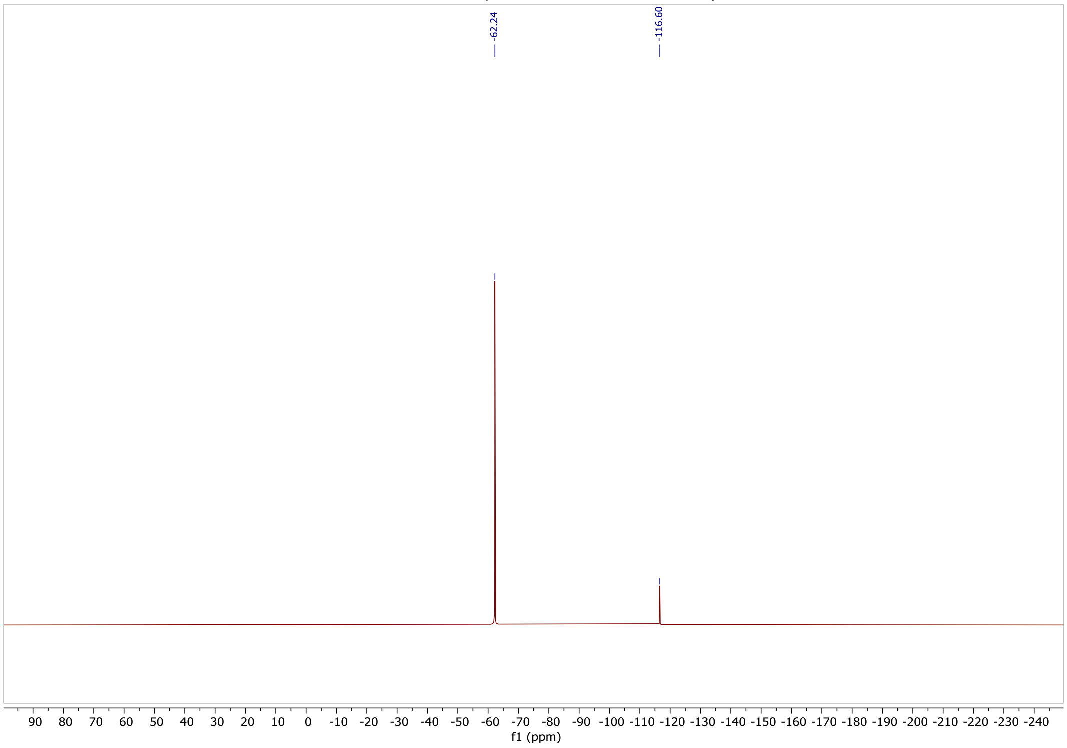

tert-Butyl 4-(3-(4-methoxyphenyl)bicyclo[1.1.1]pentan-1-yl)piperidine-1-carboxylate, 2e

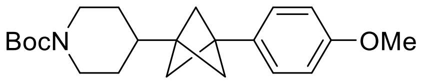

^{1}H NMR (400 MHz, CD_3OD)

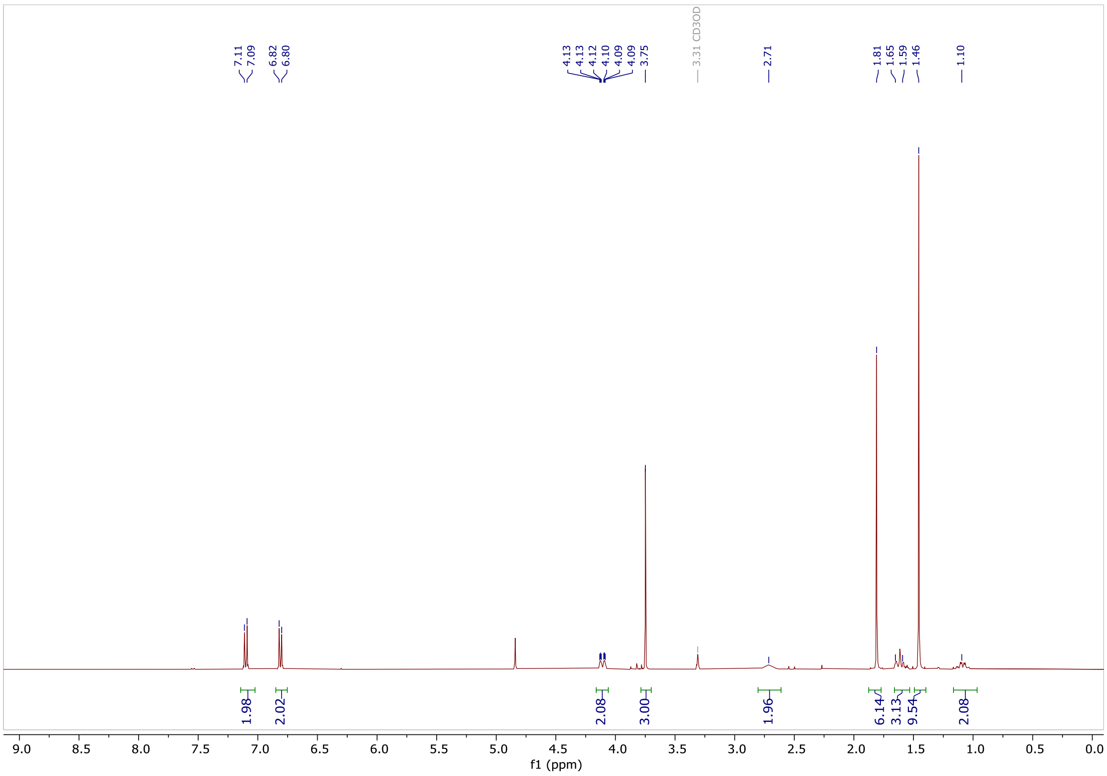

^{13}C NMR (101 MHz, CD_3OD)

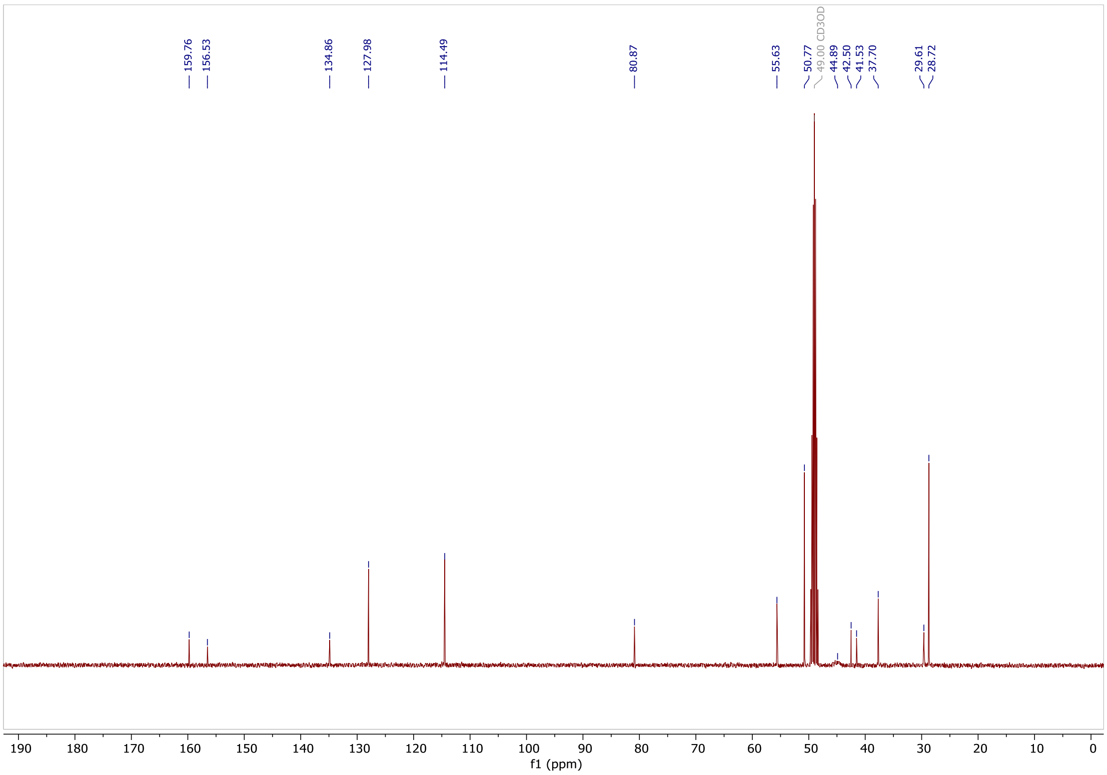

***tert*-Butyl 4-(3-(4-ethoxyphenyl)bicyclo[1.1.1]pentan-1-yl)piperidine-1-carboxylate, 2f**

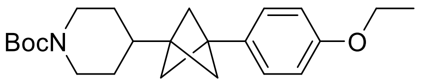

^{1}H NMR (400 MHz, $CDCl_3$)

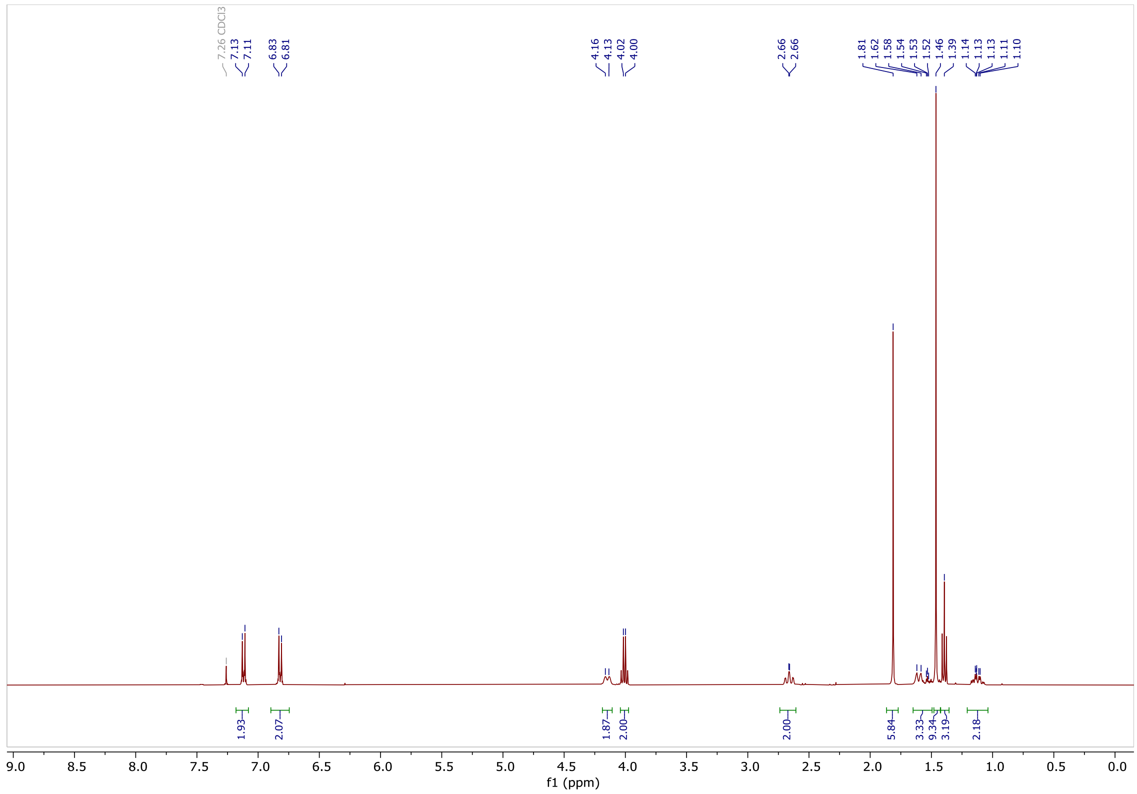

^{13}C NMR (101 MHz, $CDCl_3$)

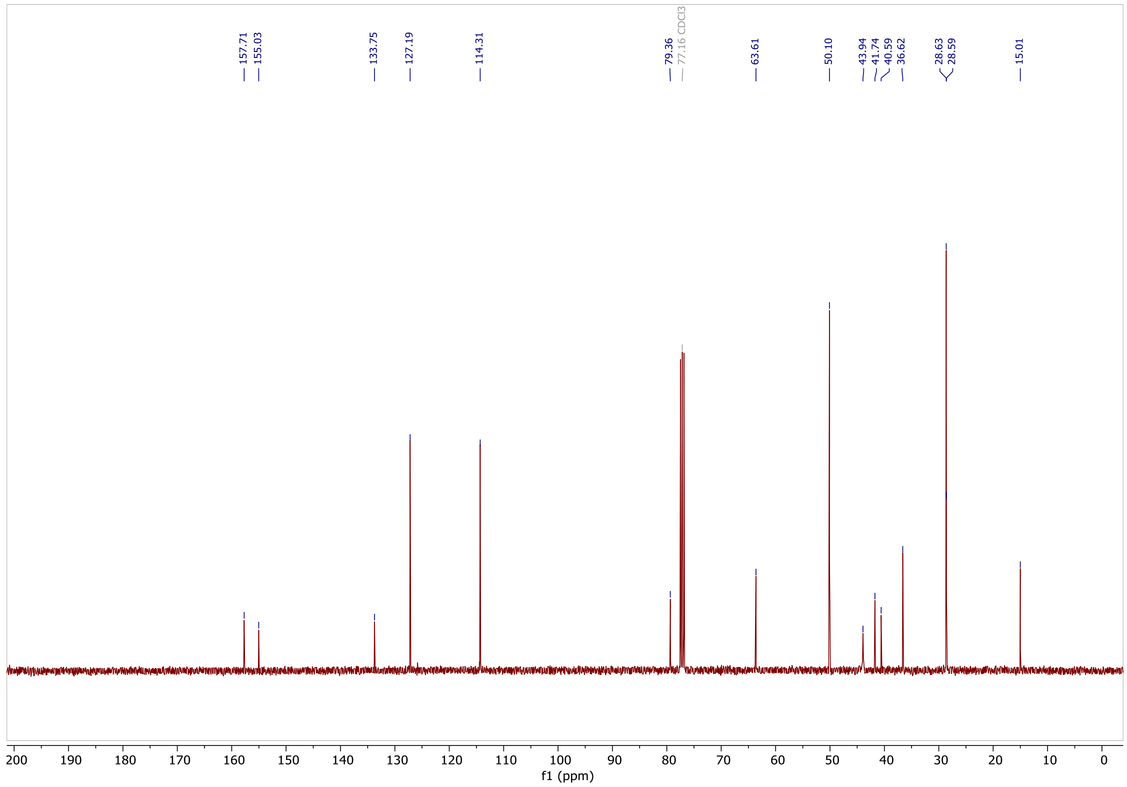

tert-Butyl 4-(3-(4-phenoxyphenyl)bicyclo[1.1.1]pentan-1-yl)piperidine-1-carboxylate, 2g

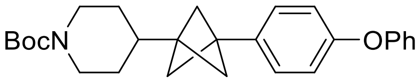

^{1}H NMR (400 MHz, $CDCl_3$)

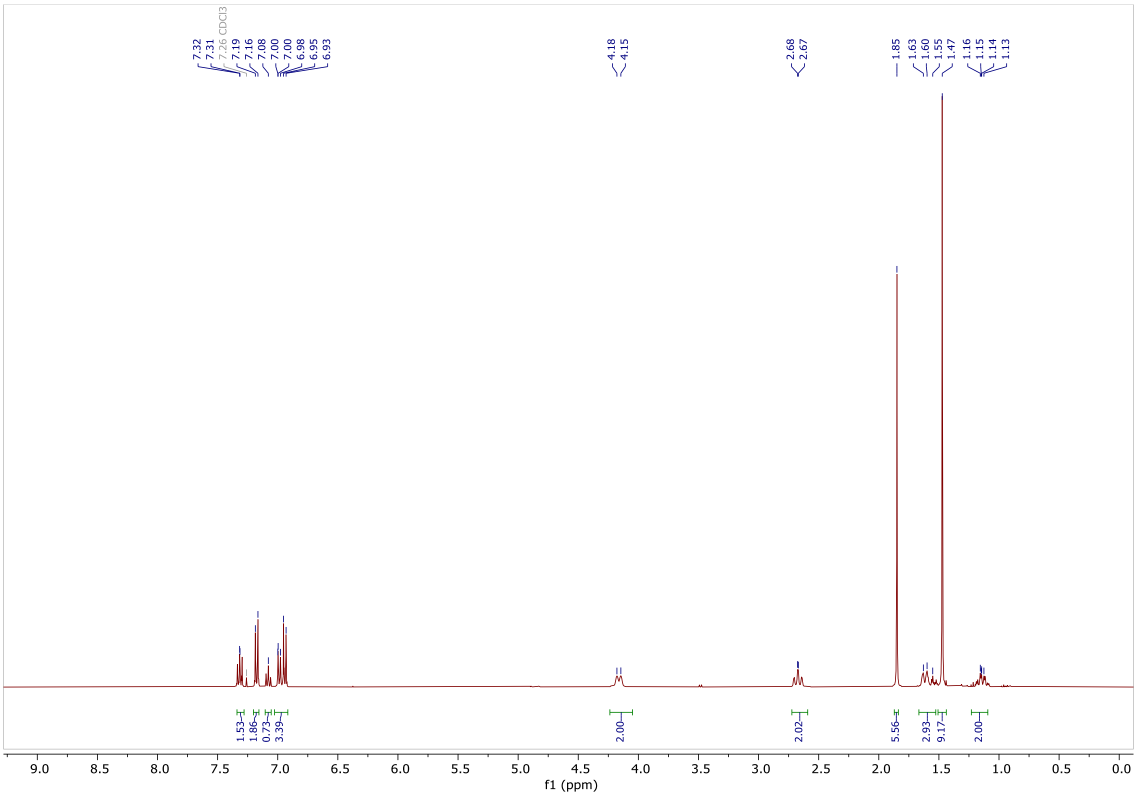

^{13}C NMR (101 MHz, $CDCl_3$)

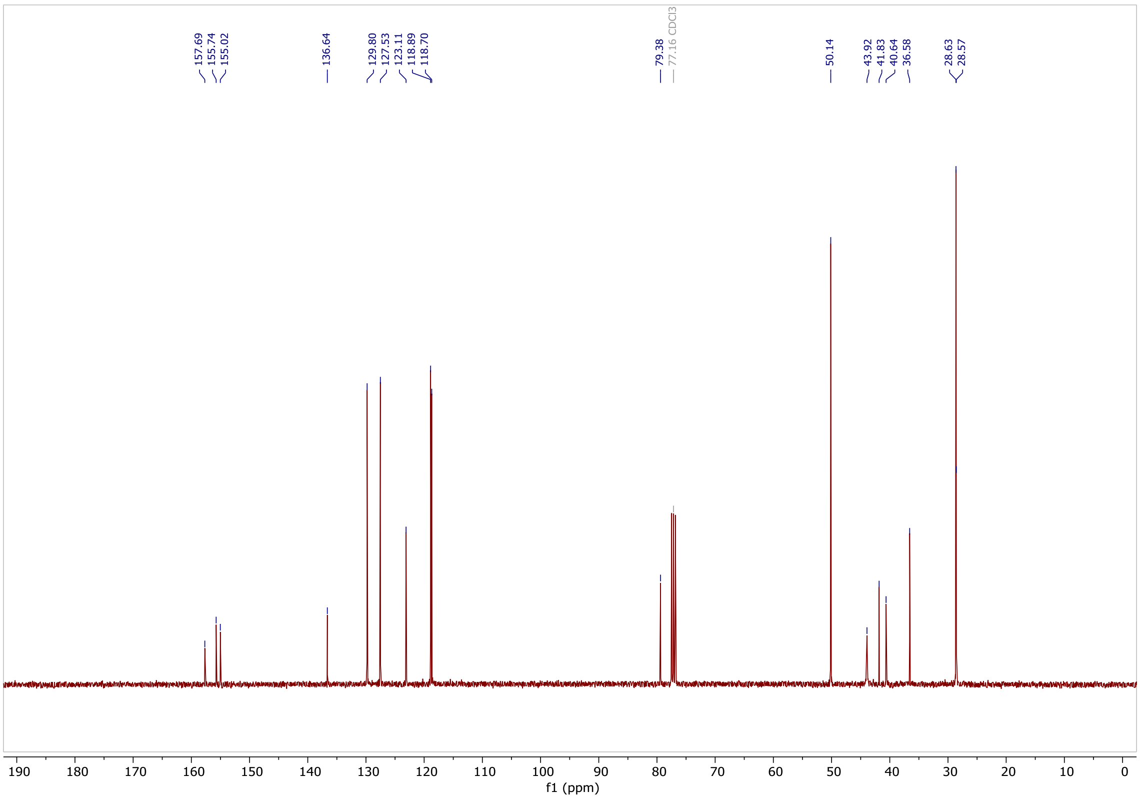

***tert*-Butyl 4-(3-(*p*-tolyl)bicyclo[1.1.1]pentan-1-yl)piperidine-1-carboxylate, 2h**

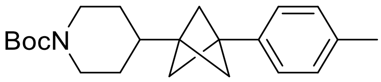

^{1}H NMR (400 MHz, $CDCl_3$)

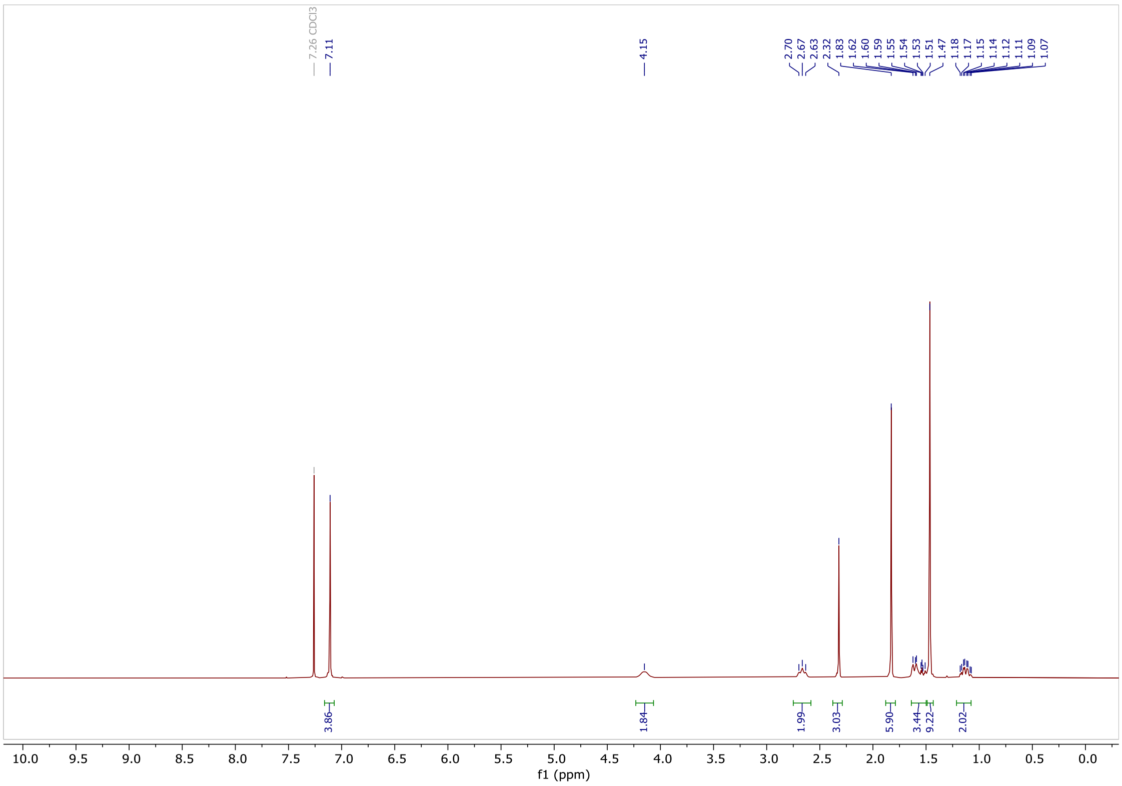

^{13}C NMR (101 MHz, $CDCl_3$)

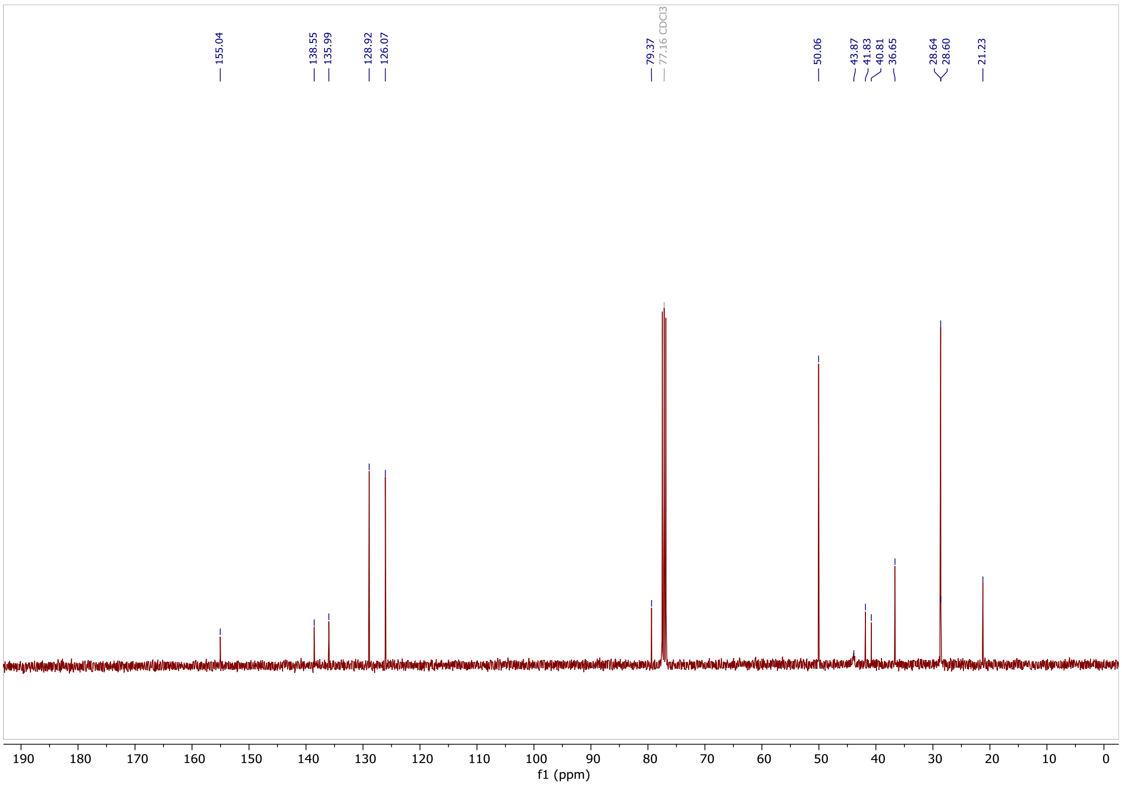

tert-Butyl 4-(3-phenylbicyclo[1.1.1]pentan-1-yl)piperidine-1-carboxylate, 2i

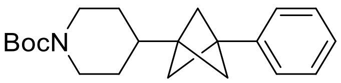

^{1}H NMR (400 MHz, $CDCl_3$)

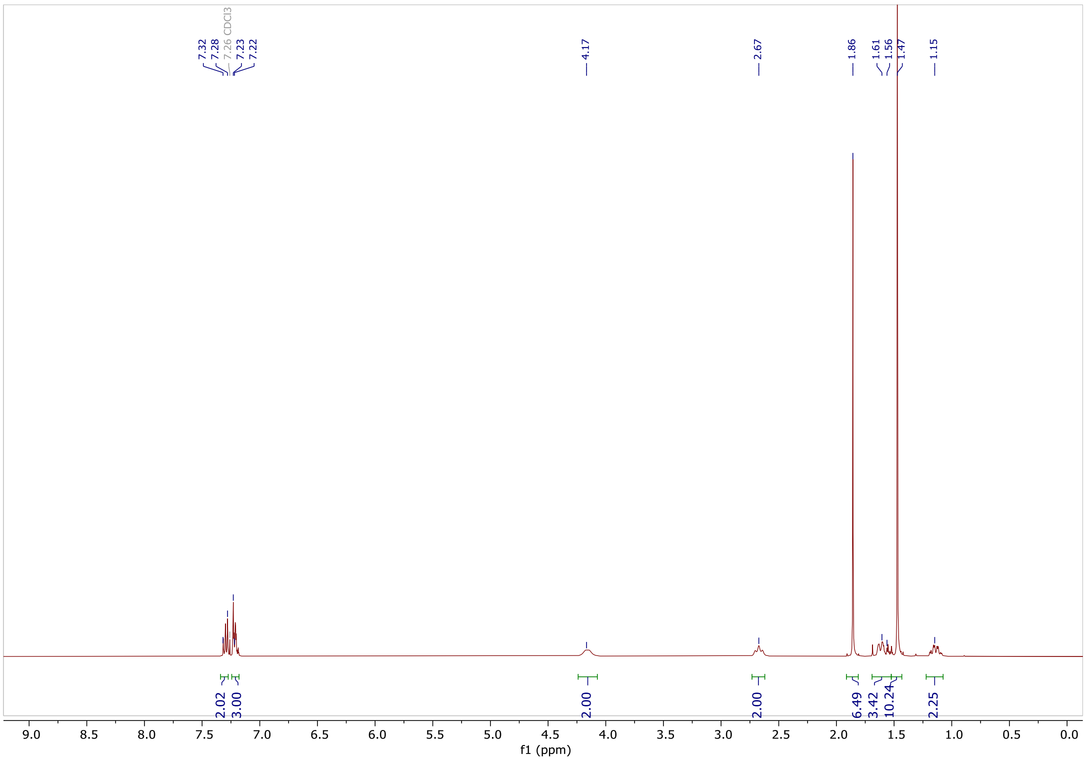

^{13}C NMR (101 MHz, $CDCl_3$)

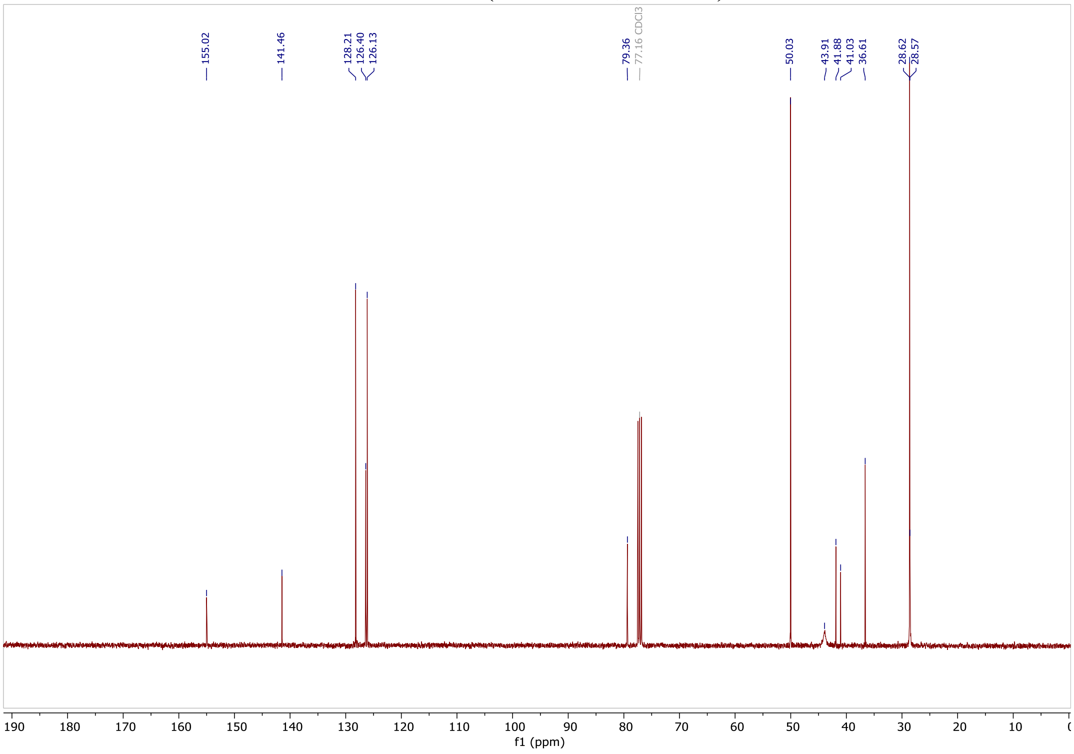

tert-Butyl 4-(3-(4-(_tert_-butyl)phenyl)bicyclo[1.1.1]pentan-1-yl)piperidine-1-carboxylate, 2j

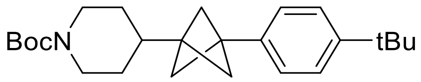

^{1}H NMR (400 MHz, $CDCl_3$)

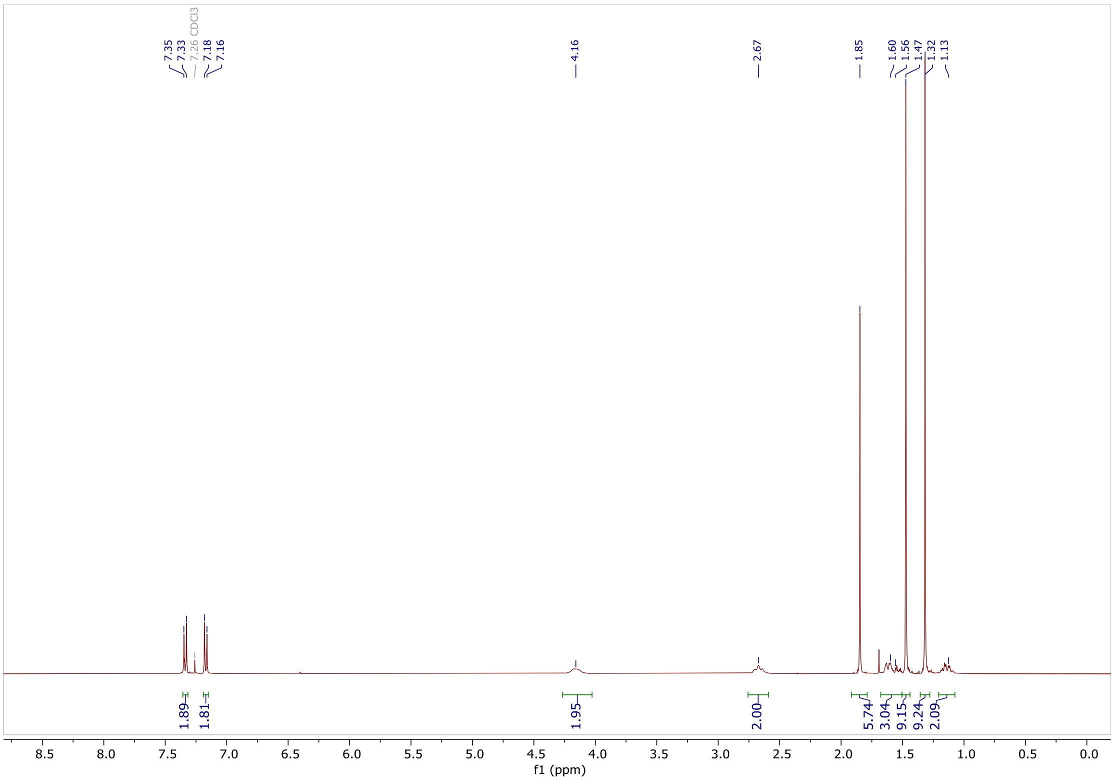

^{13}C NMR (101 MHz, $CDCl_3$)

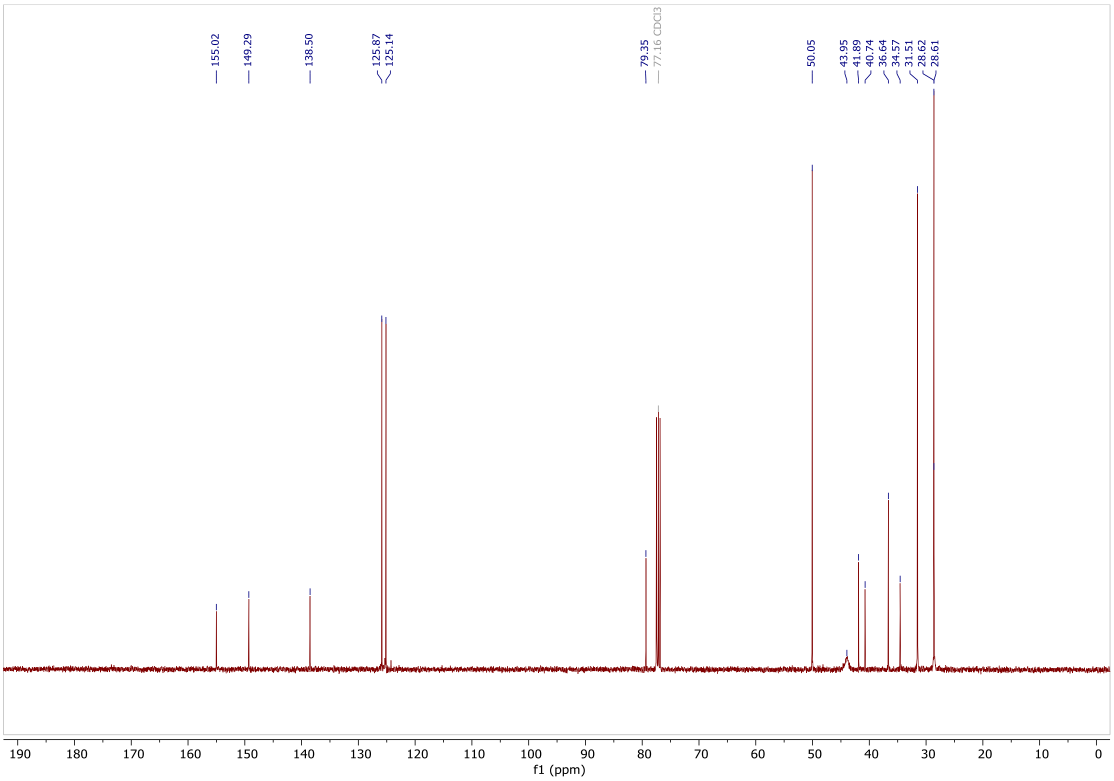

***tert*-Butyl 4-(3-(4-fluorophenyl)bicyclo[1.1.1]pentan-1-yl)piperidine-1-carboxylate, 2k**

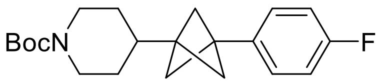

^{1}H NMR (400 MHz, $CDCl_3$)

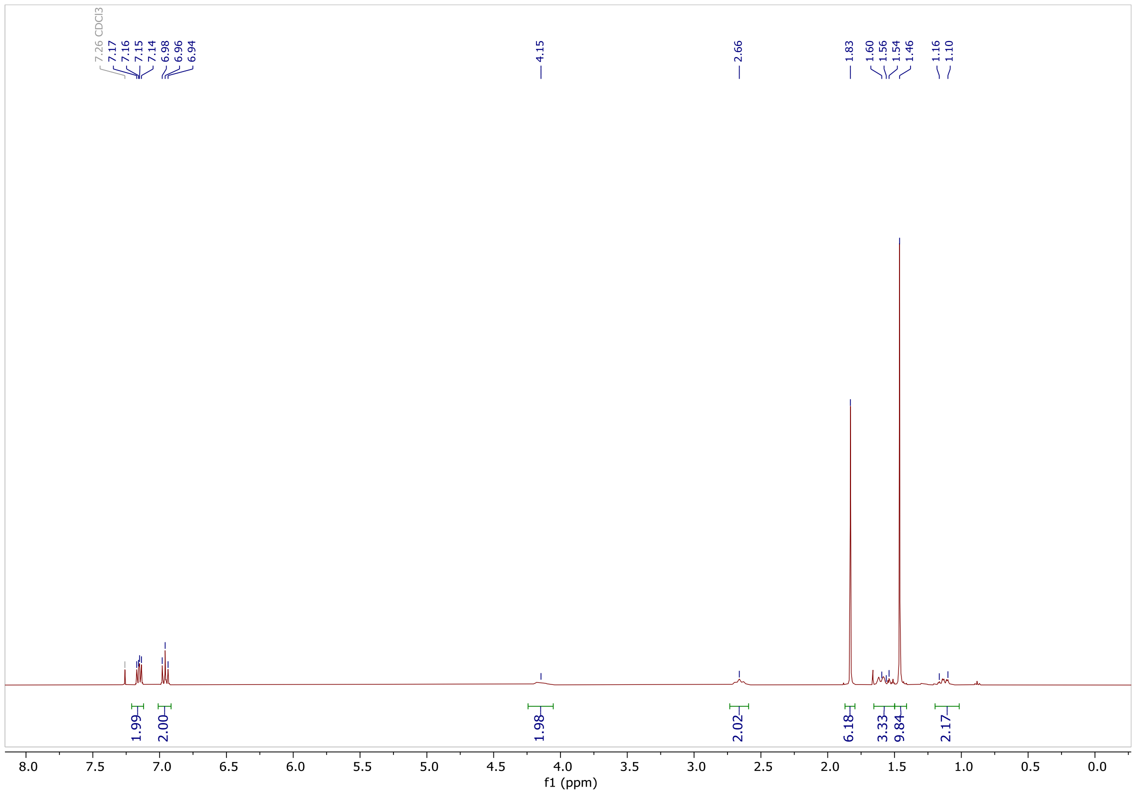

^{13}C NMR (101 MHz, $CDCl_3$)

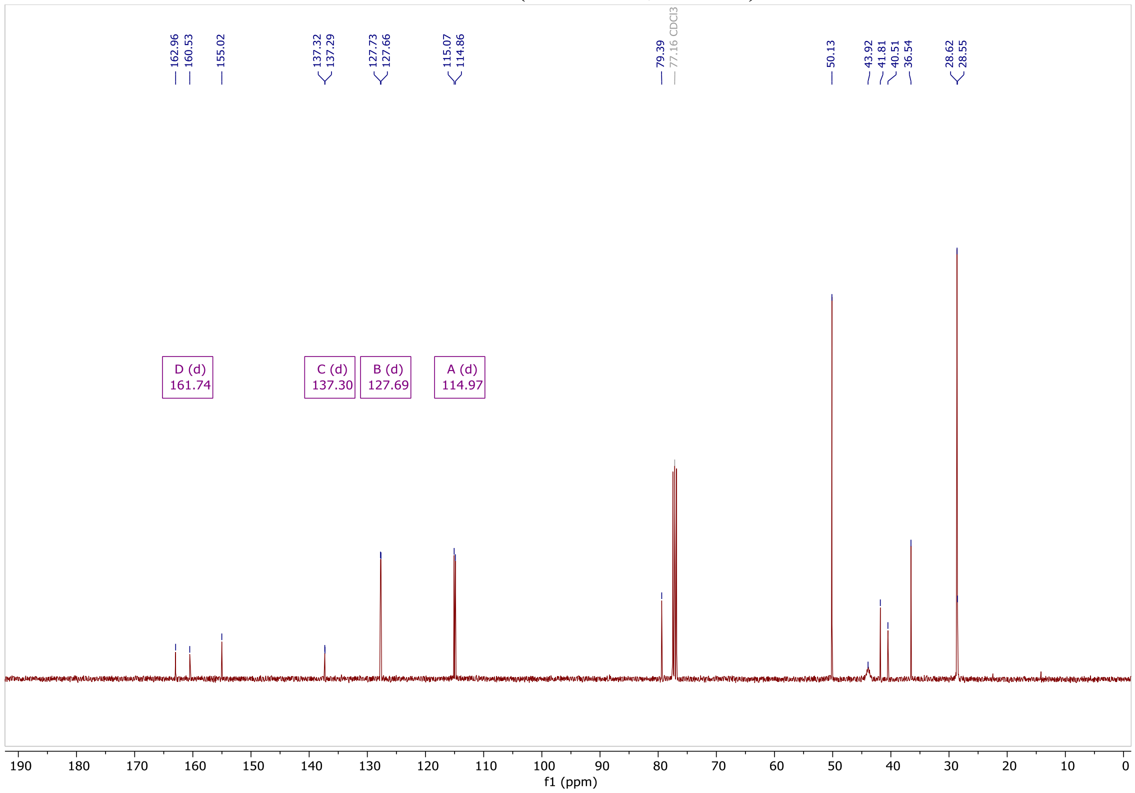

^{19}F NMR (376 MHz, $CDCl_3$)

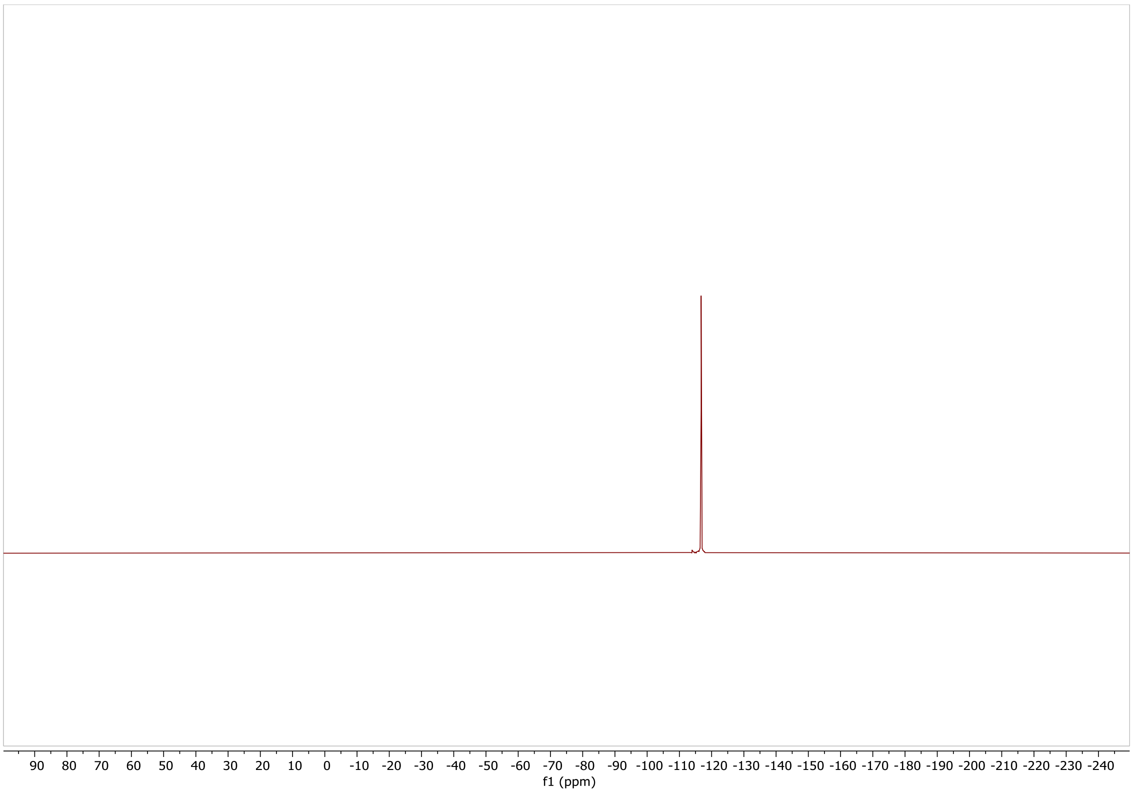

tert-Butyl 4-(3-(4-(pyridin-2-yl)phenyl)bicyclo[1.1.1]pentan-1-yl)piperidine-1-carboxylate, 2l

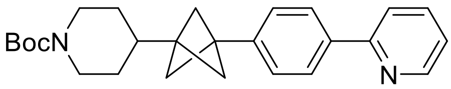

^{1}H NMR (400 MHz, $CDCl_3$)

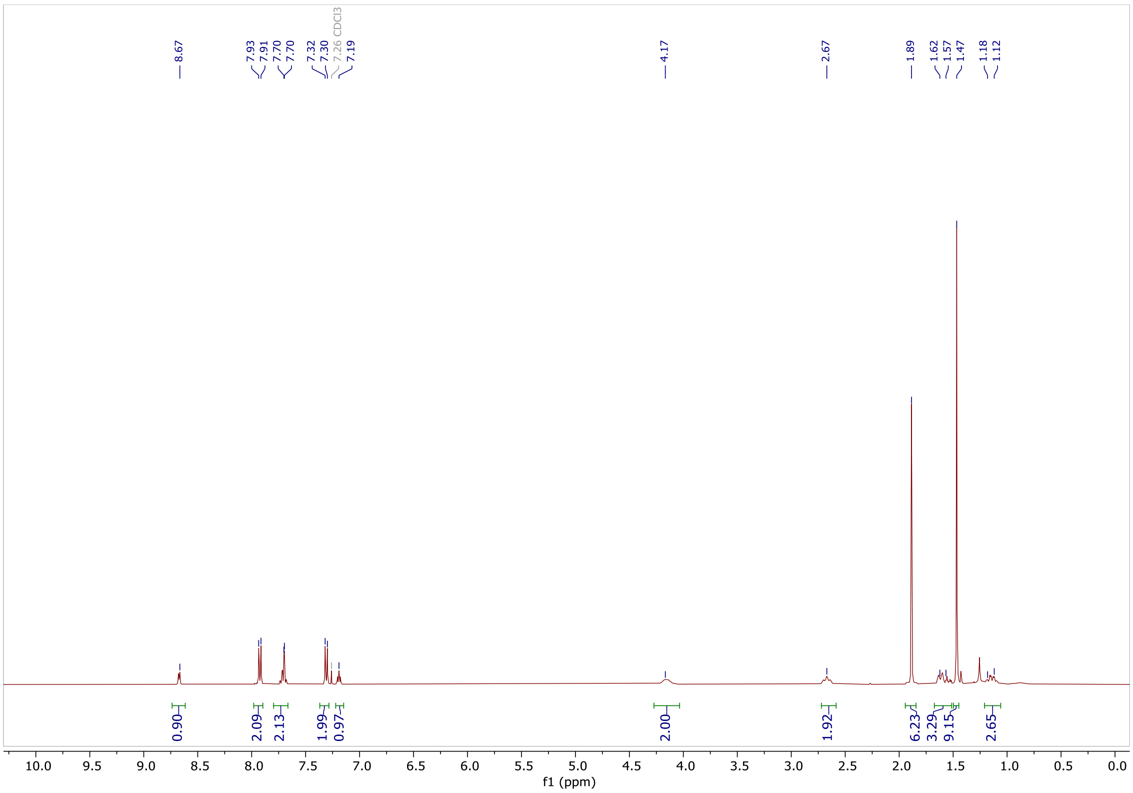

^{13}C NMR (101 MHz, $CDCl_3$)

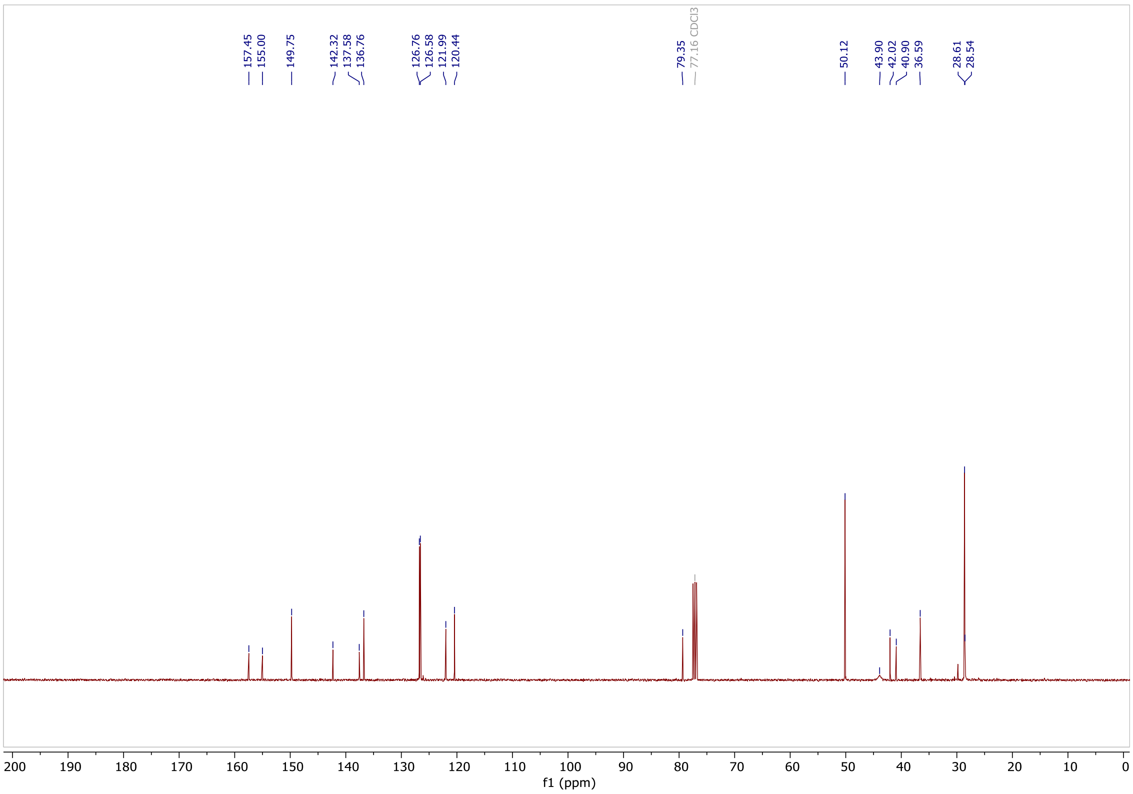

tert-butyl 4-(3-(4-(trimethylsilyl)phenyl)bicyclo[1.1.1]pentan-1-yl)piperidine-1-carboxylate, 2m

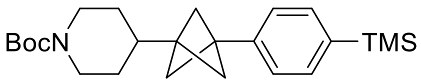

^{1}H NMR (400 MHz, $CDCl_3$)

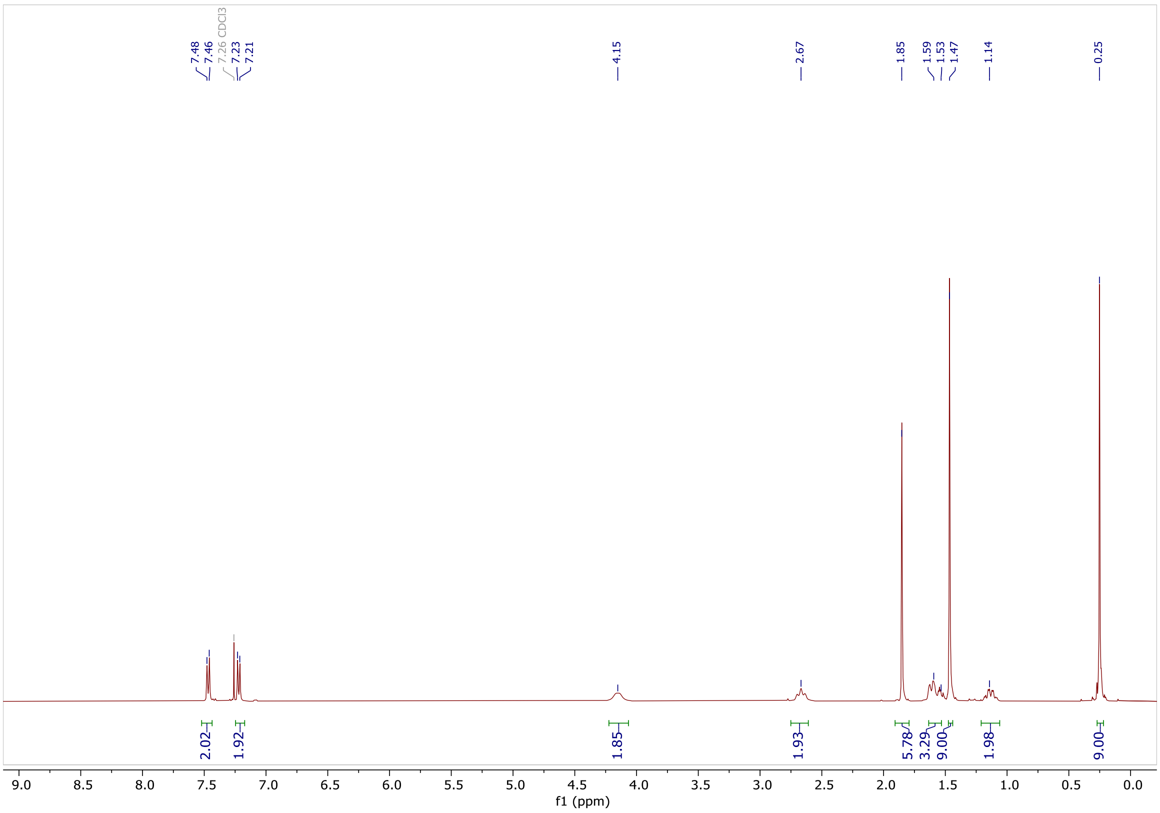

^{13}C NMR (101 MHz, $CDCl_3$)

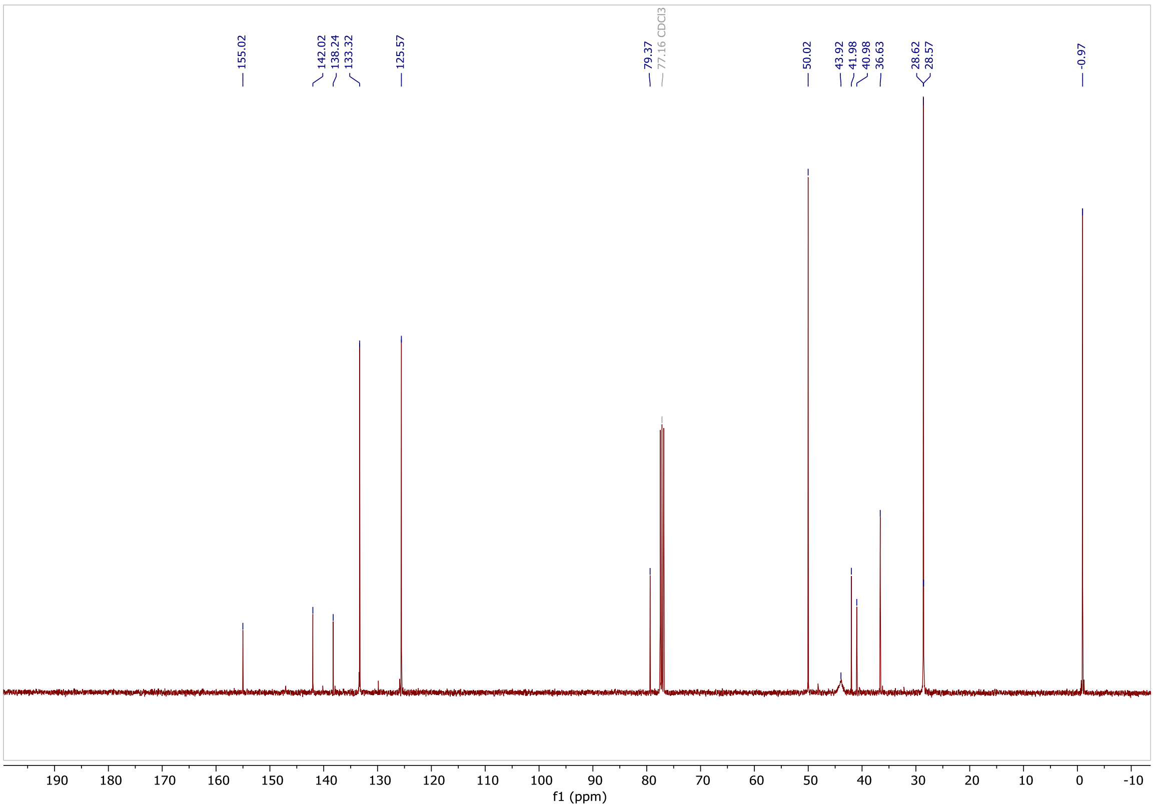

1-(2-Methoxyphenyl)-3-(4-(trifluoromethyl)benzyl)bicyclo[1.1.1]pentane, 2n

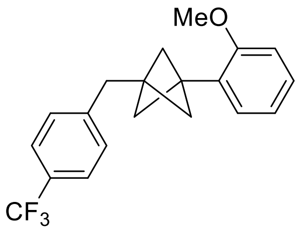

^{1}H NMR (400 MHz, $CDCl_3$)

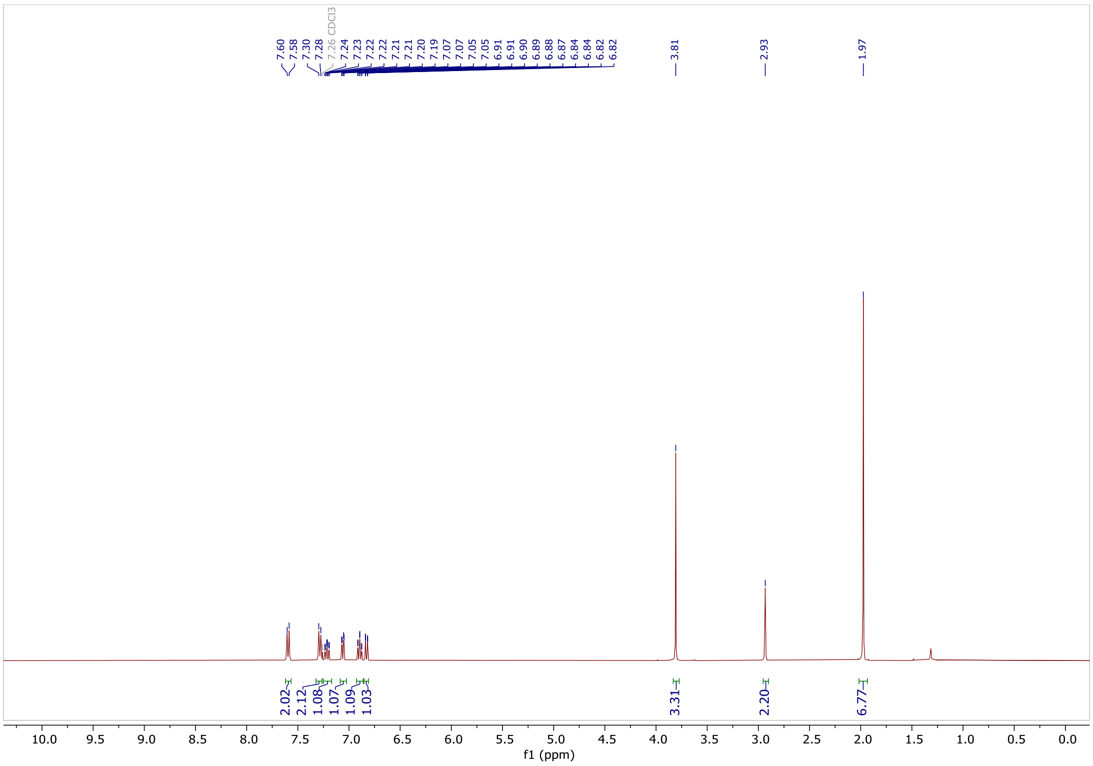

^{13}C NMR (101 MHz, $CDCl_3$)

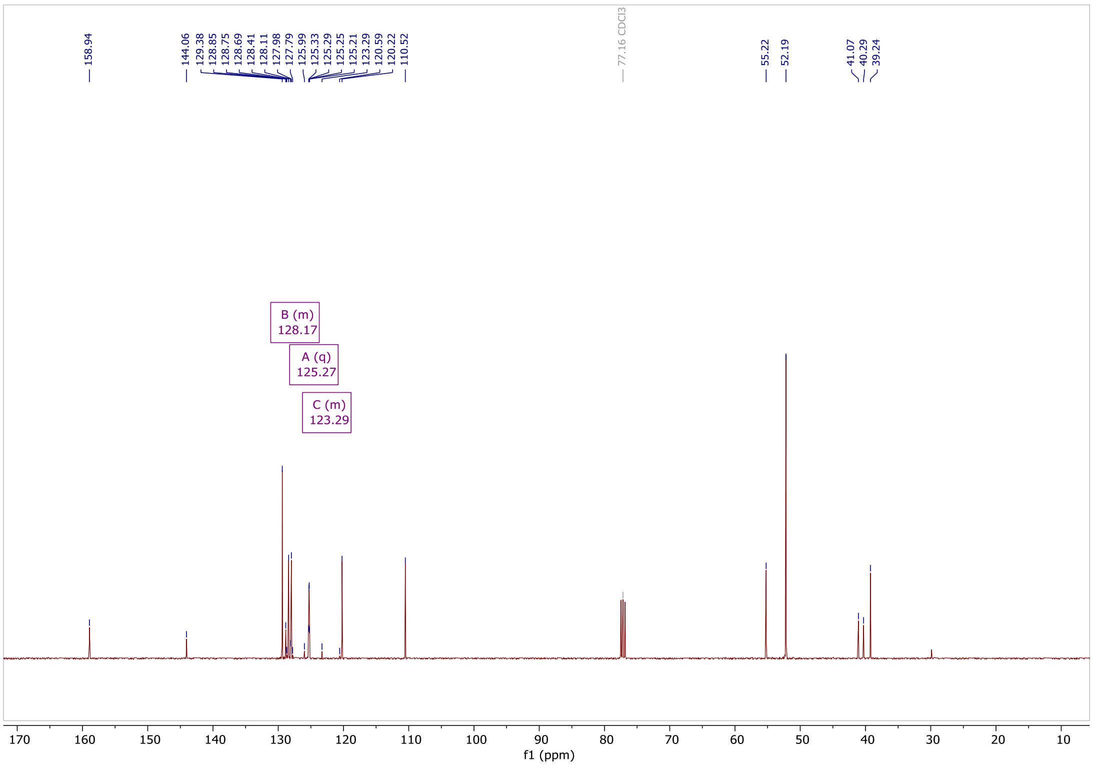

^{19}F NMR (376 MHz, $CDCl_3$)

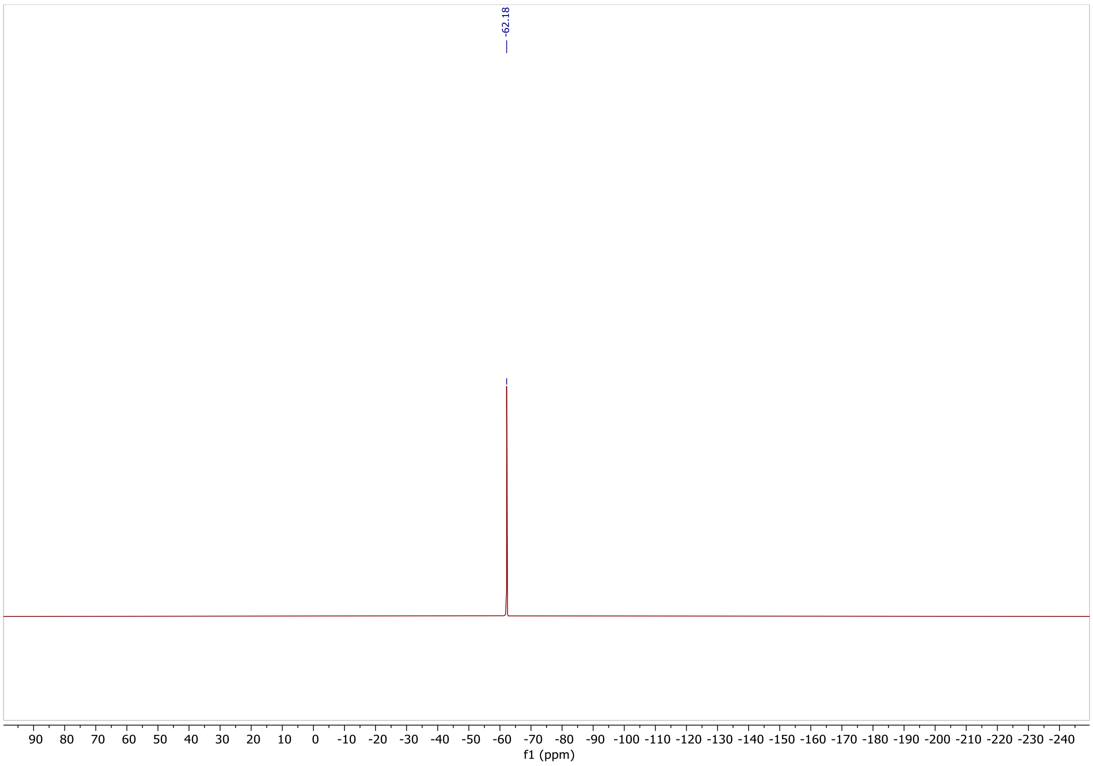

***tert*-butyl 4-(3-(o-tolyl)bicyclo[1.1.1]pentan-1-yl)piperidine-1-carboxylate, 2o**

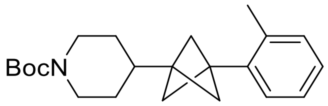

^{1}H NMR (400 MHz, $CDCl_3$)

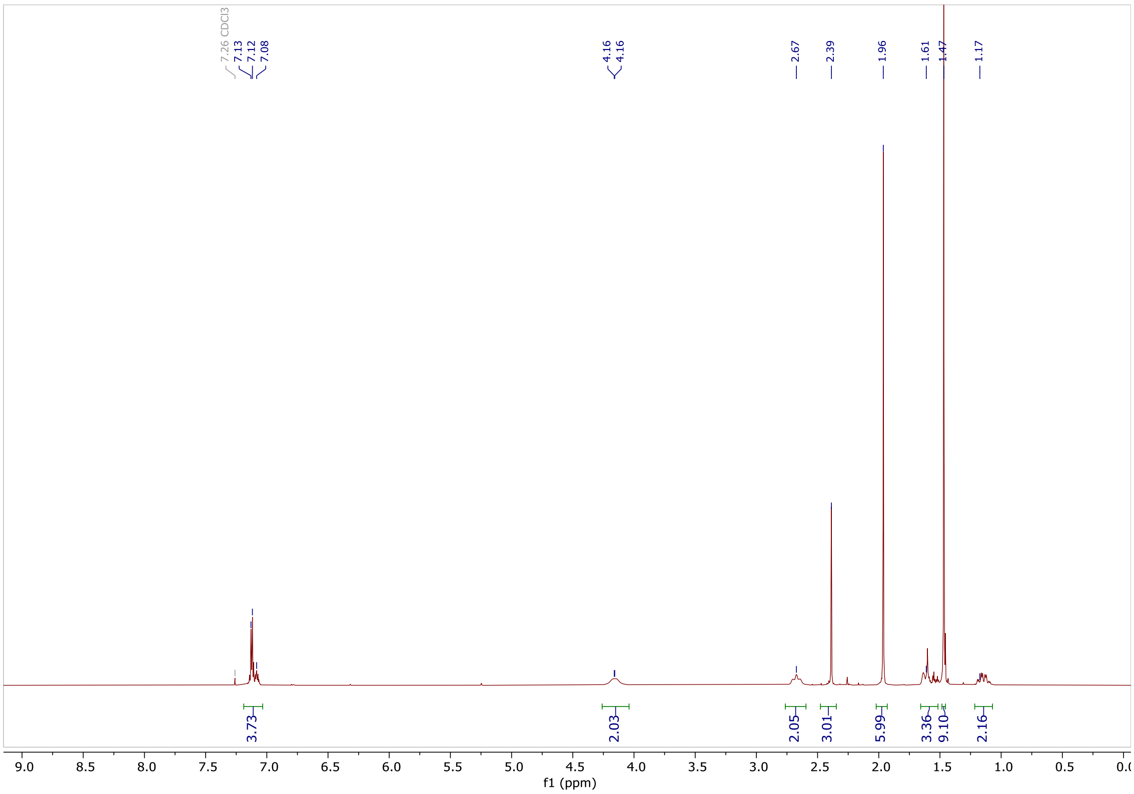

^{13}C NMR (101 MHz, $CDCl_3$)

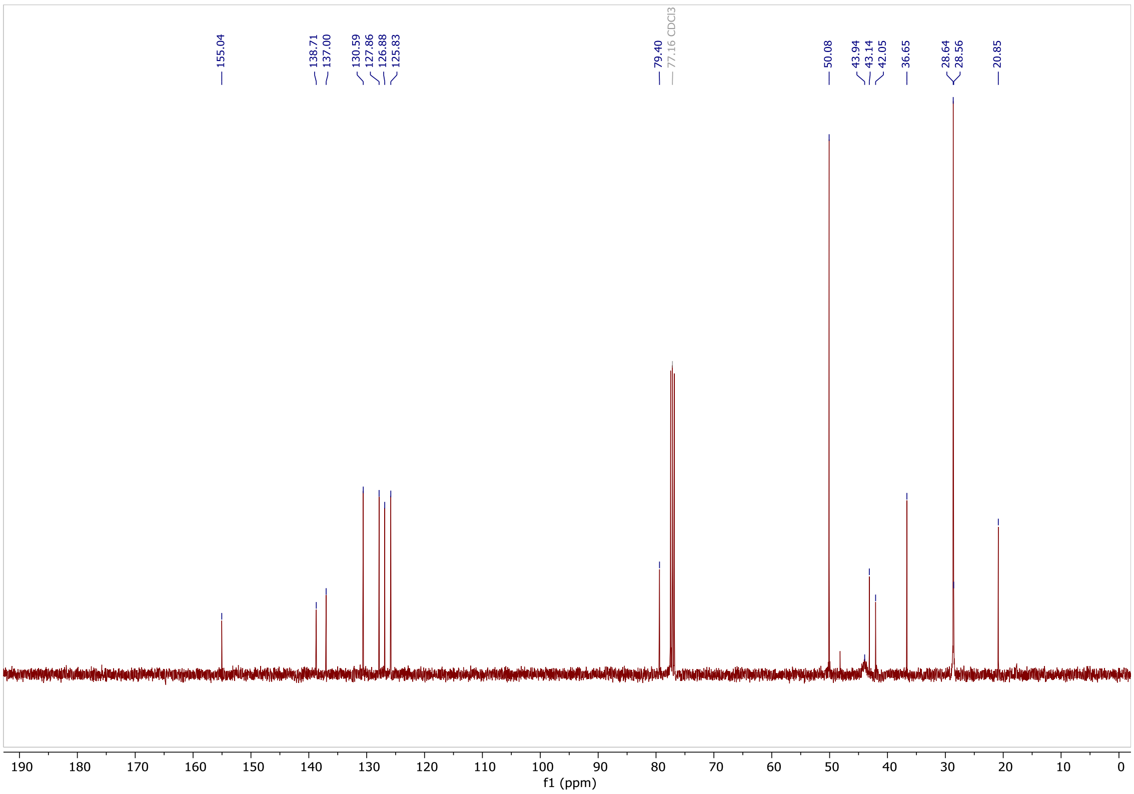

1-(3-Methoxyphenyl)-3-(4-(trifluoromethyl)benzyl)bicyclo[1.1.1]pentane, 2p

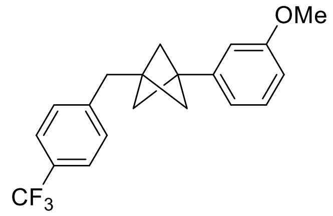

^{1}H NMR (400 MHz, $CDCl_3$)

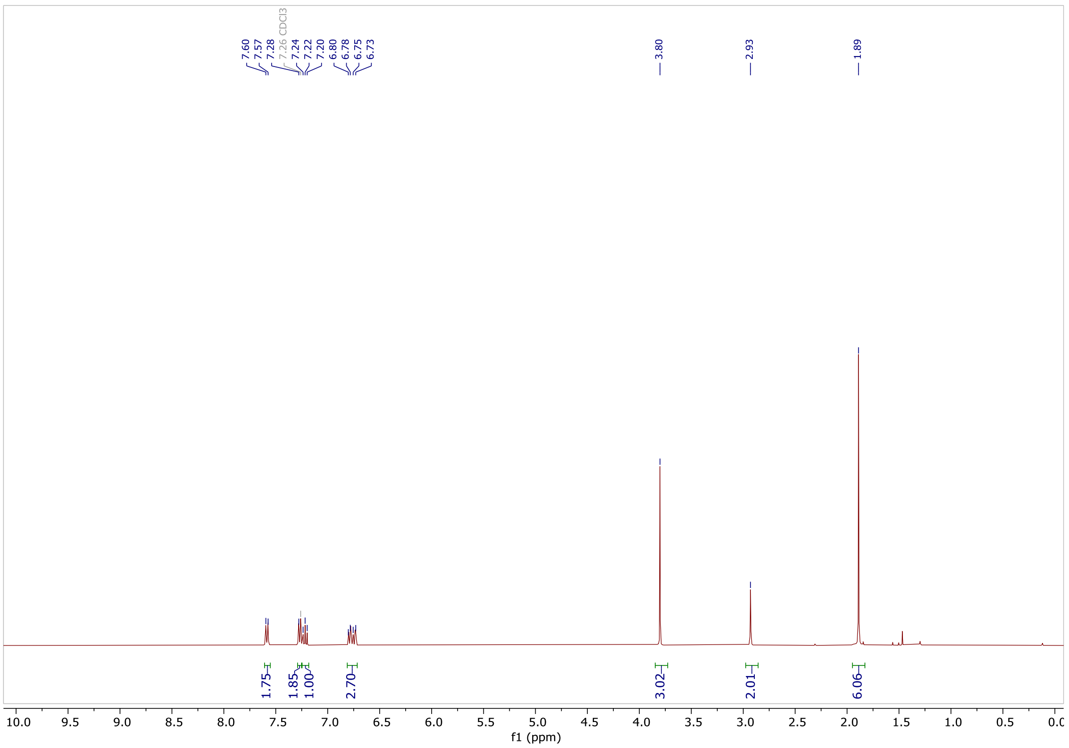

^{13}C NMR (101 MHz, $CDCl_3$)

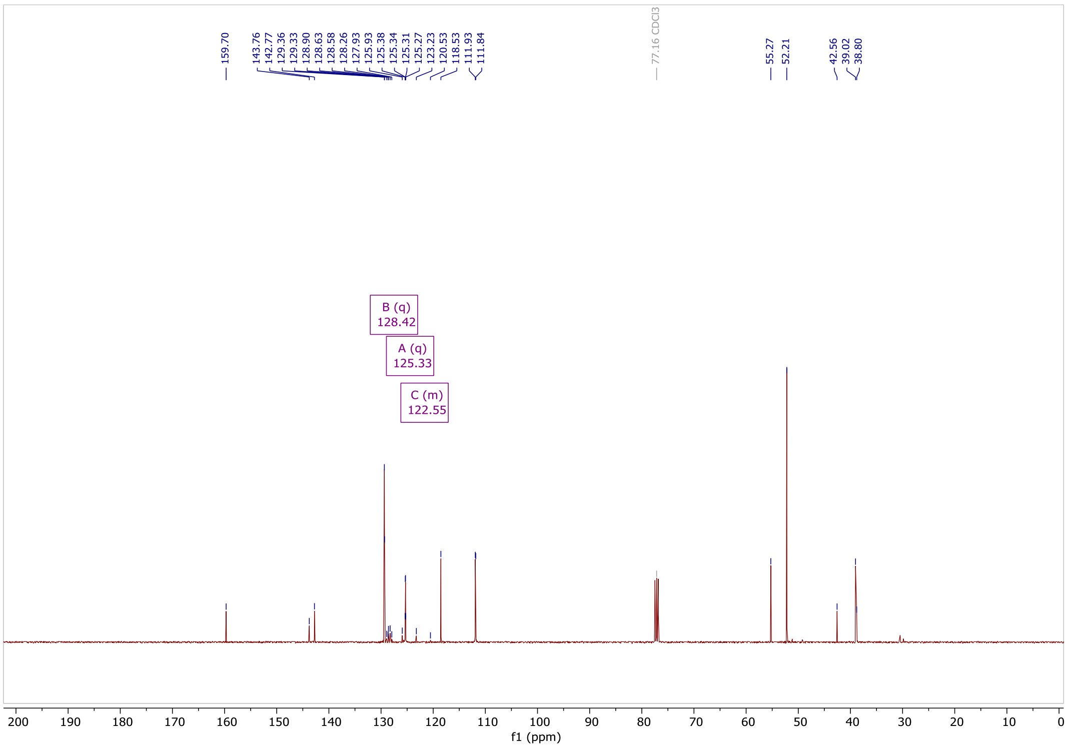

^{19}F NMR (376 MHz, $CDCl_3$)

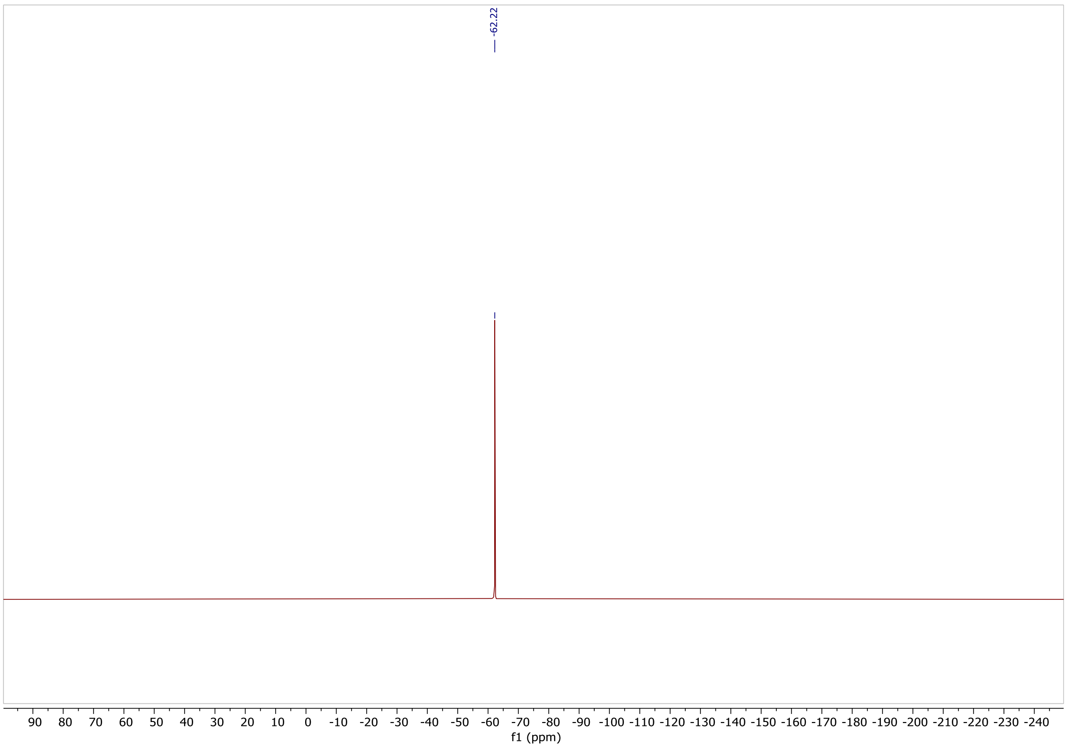

***tert*-Butyl 4-(3-(3-phenoxyphenyl)bicyclo[1.1.1]pentan-1-yl)piperidine-1-carboxylate, 2q**

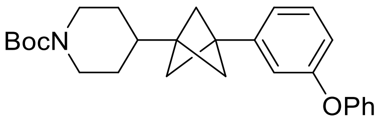

^{1}H NMR (400 MHz, $CDCl_3$)

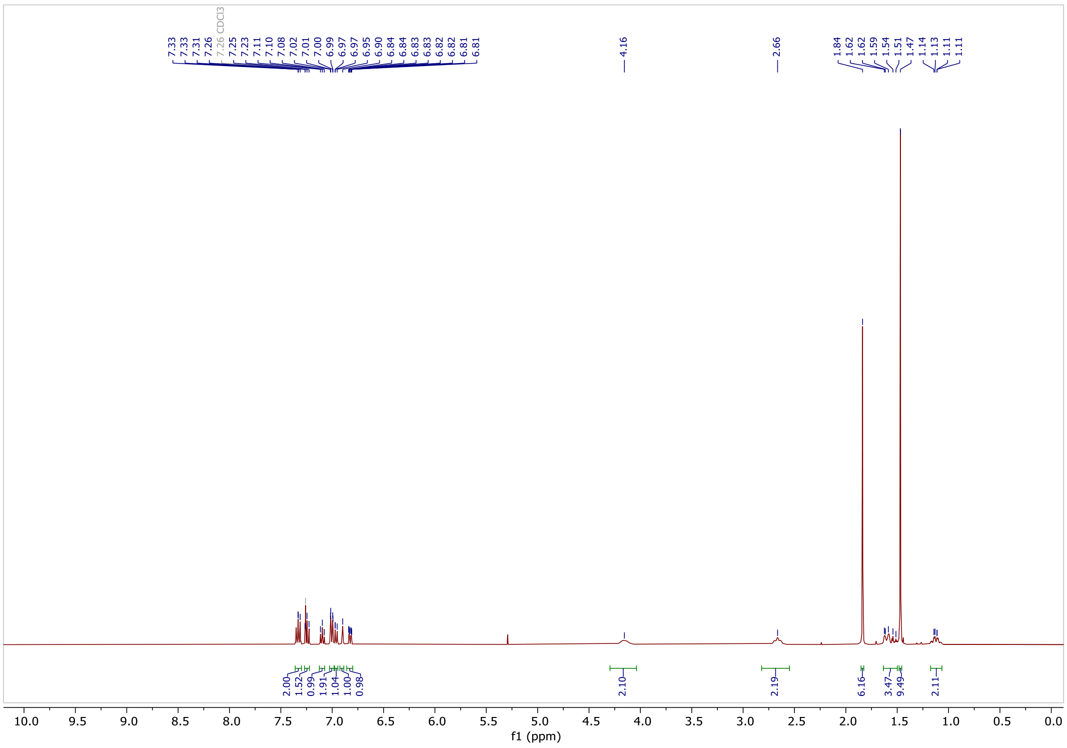

^{13}C NMR (101 MHz, $CDCl_3$)

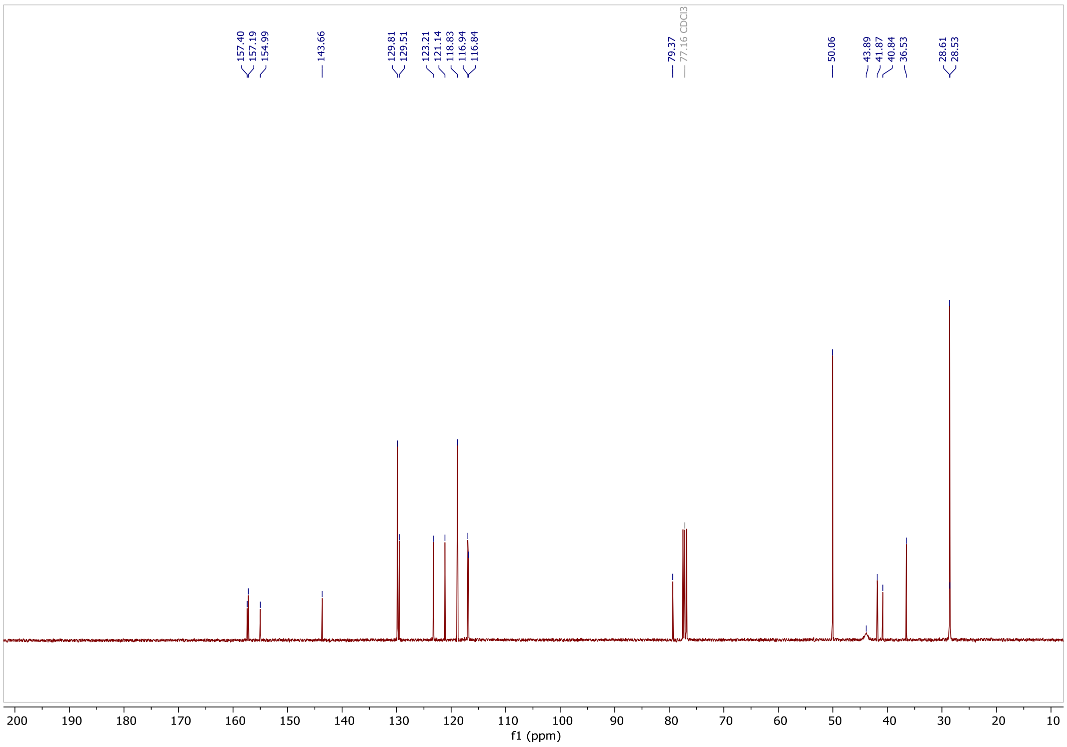

***tert*-Butyl 4-(3-(3-(1,3-dioxolan-2-yl)phenyl)bicyclo[1.1.1]pentan-1-yl)piperidine-1-carboxylate, 2r**

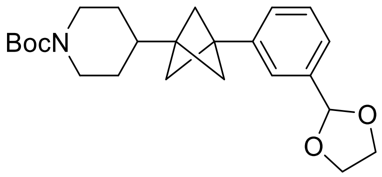

^{1}H NMR (400 MHz, $CDCl_3$)

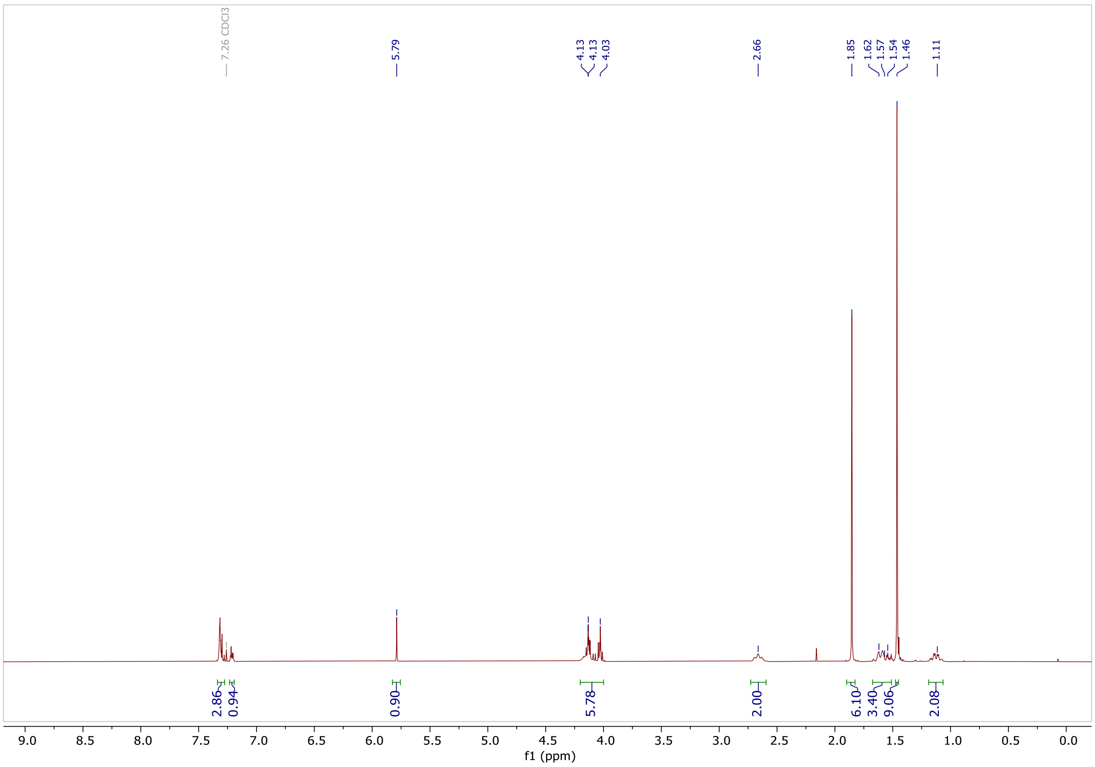

^{13}C NMR (101 MHz, $CDCl_3$)

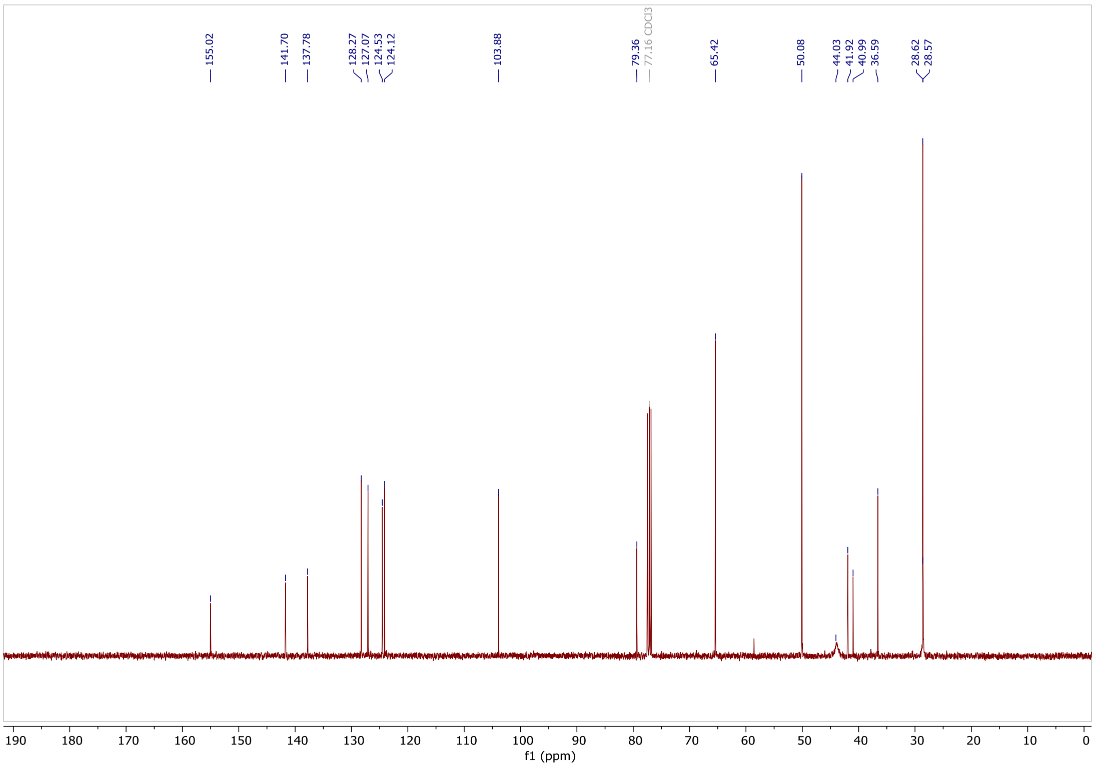

1-(5-Fluoro-2-methoxyphenyl)-3-(4-(trifluoromethyl)benzyl)bicyclo[1.1.1]pentane, 2s

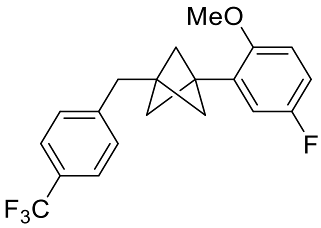

^{1}H NMR (400 MHz, $CDCl_3$)

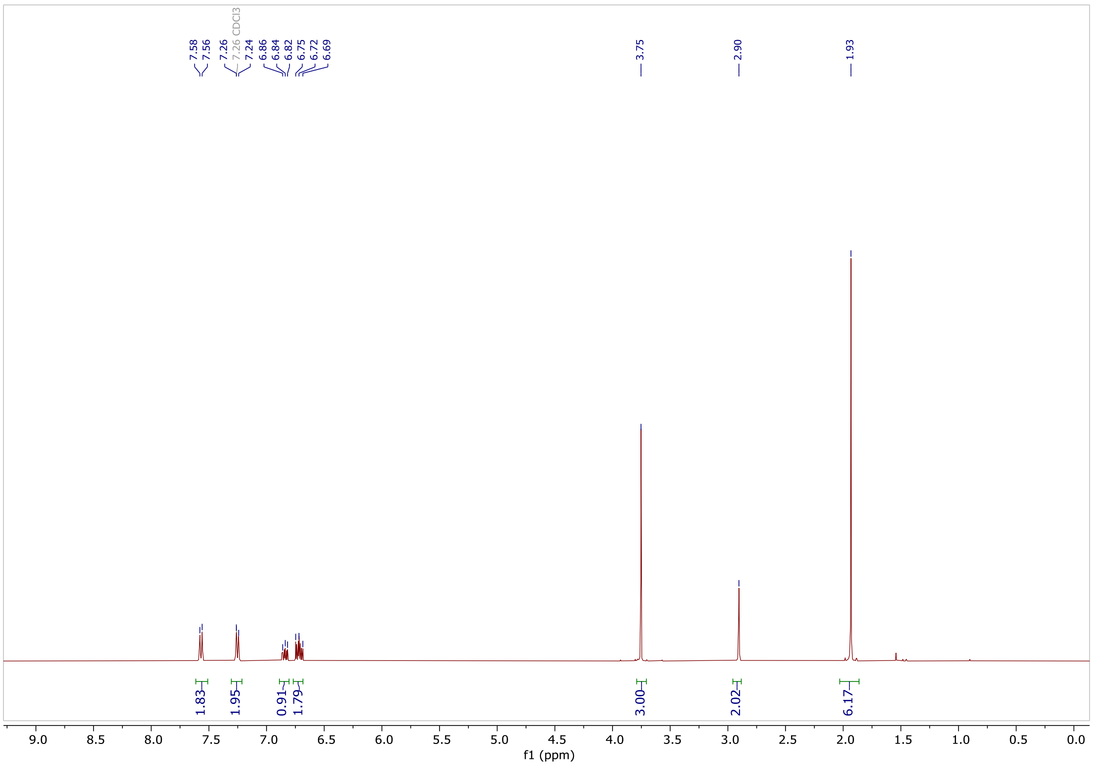

^{13}C NMR (101 MHz, $CDCl_3$)

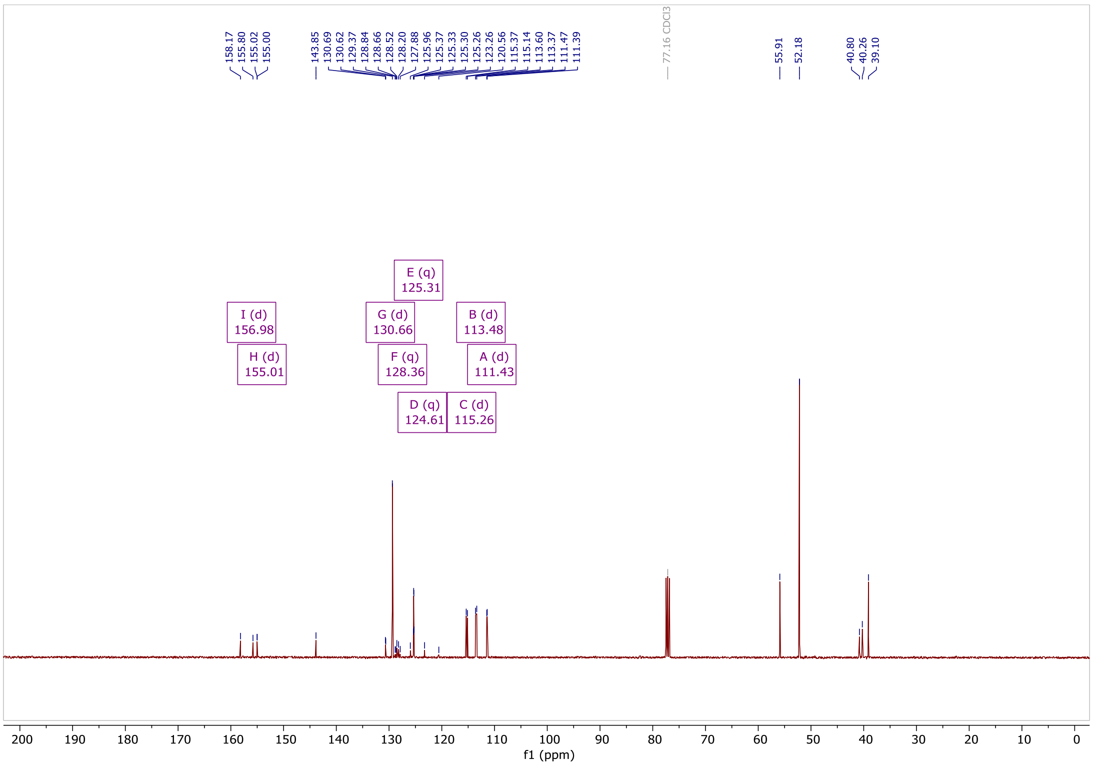

^{19}F NMR (376 MHz, $CDCl_3$)

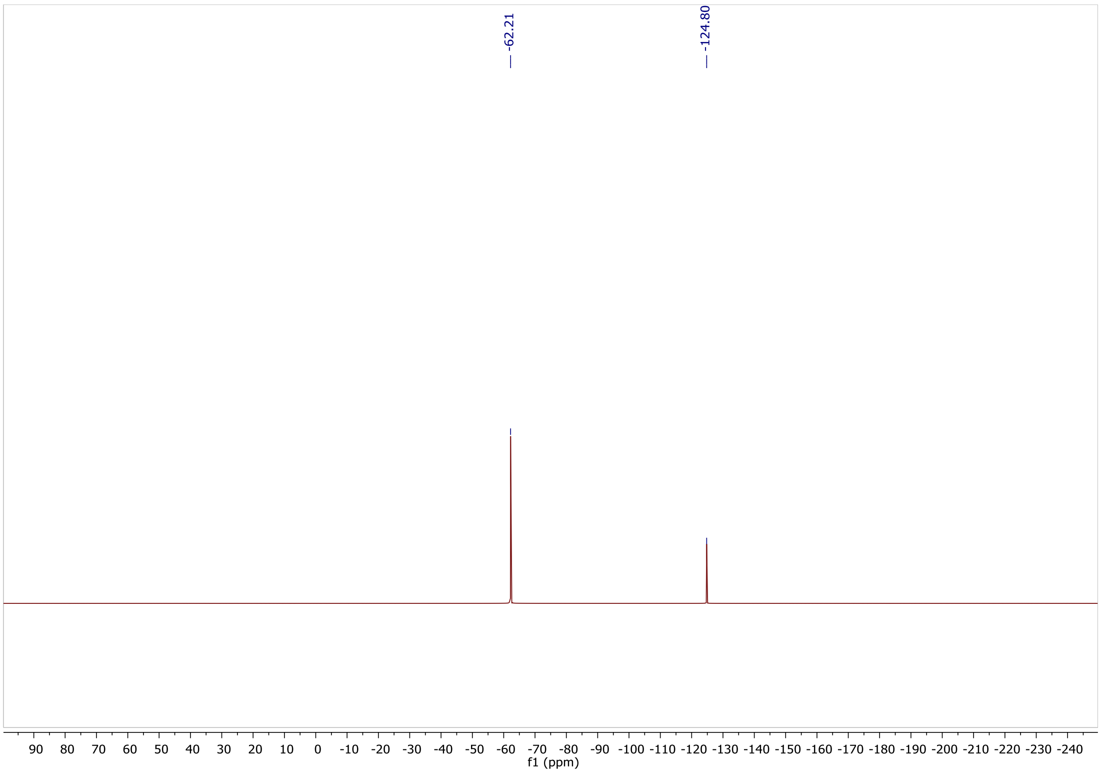

5-(3-(4-(Trifluoromethyl)benzyl)bicyclo[1.1.1]pentan-1-yl)-2,3-dihydrobenzofuran, 2t

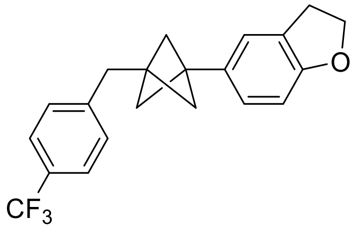

^{1}H NMR (400 MHz, $CDCl_3$)

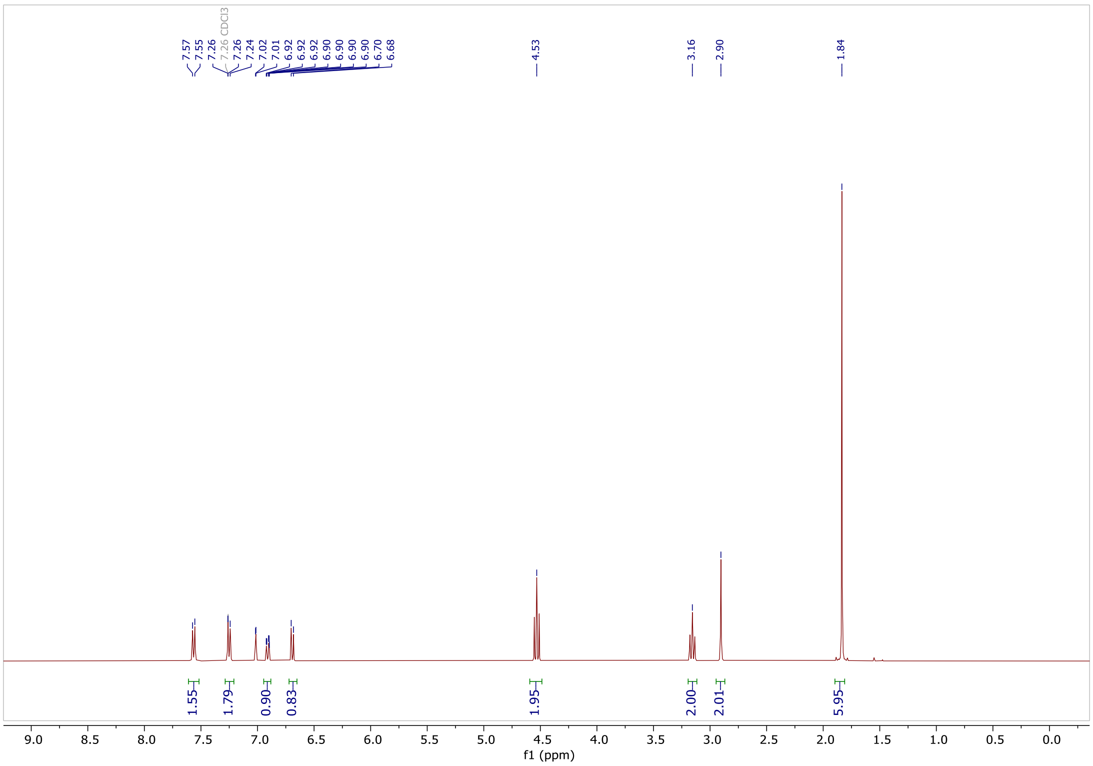

^{13}C NMR (101 MHz, $CDCl_3$)

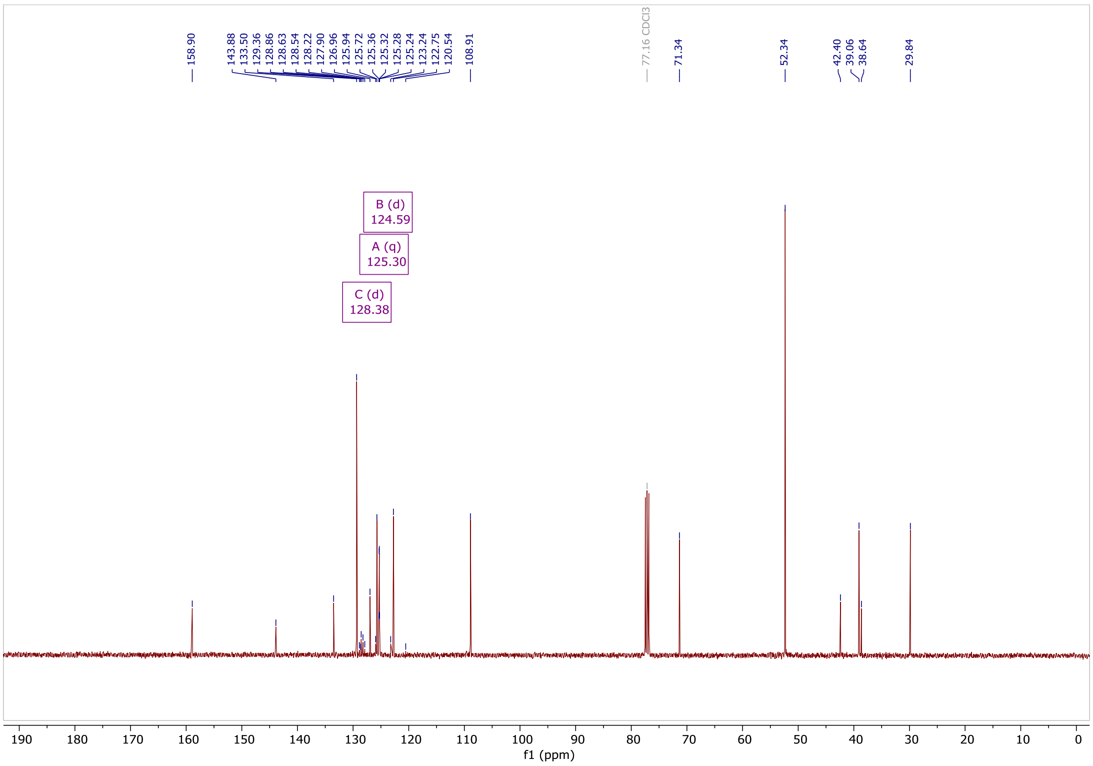

^{19}F NMR (376 MHz, $CDCl_3$)

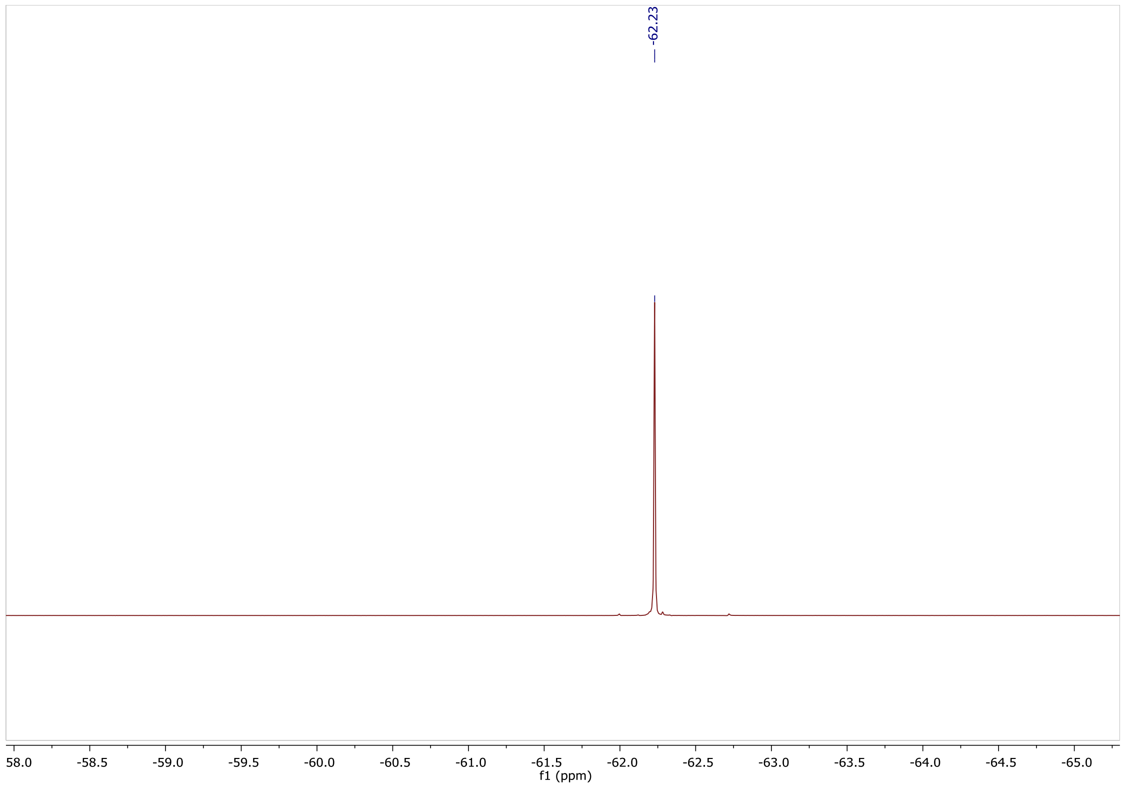

5-(3-(4-(Trifluoromethyl)benzyl)bicyclo[1.1.1]pentan-1-yl)benzo[*d*][1,3]dioxole, 2u

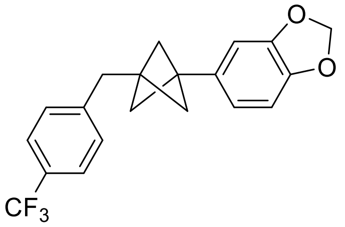

^{1}H NMR (400 MHz, $CDCl_3$)

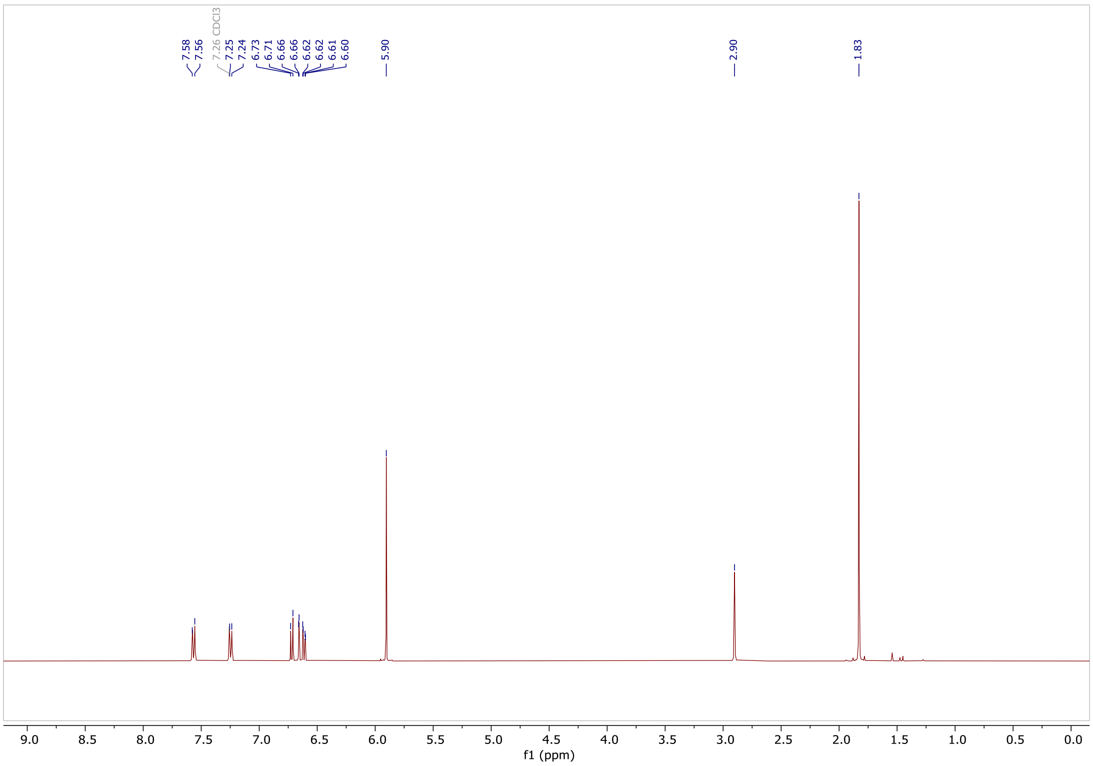

^{13}C NMR (101 MHz, $CDCl_3$)

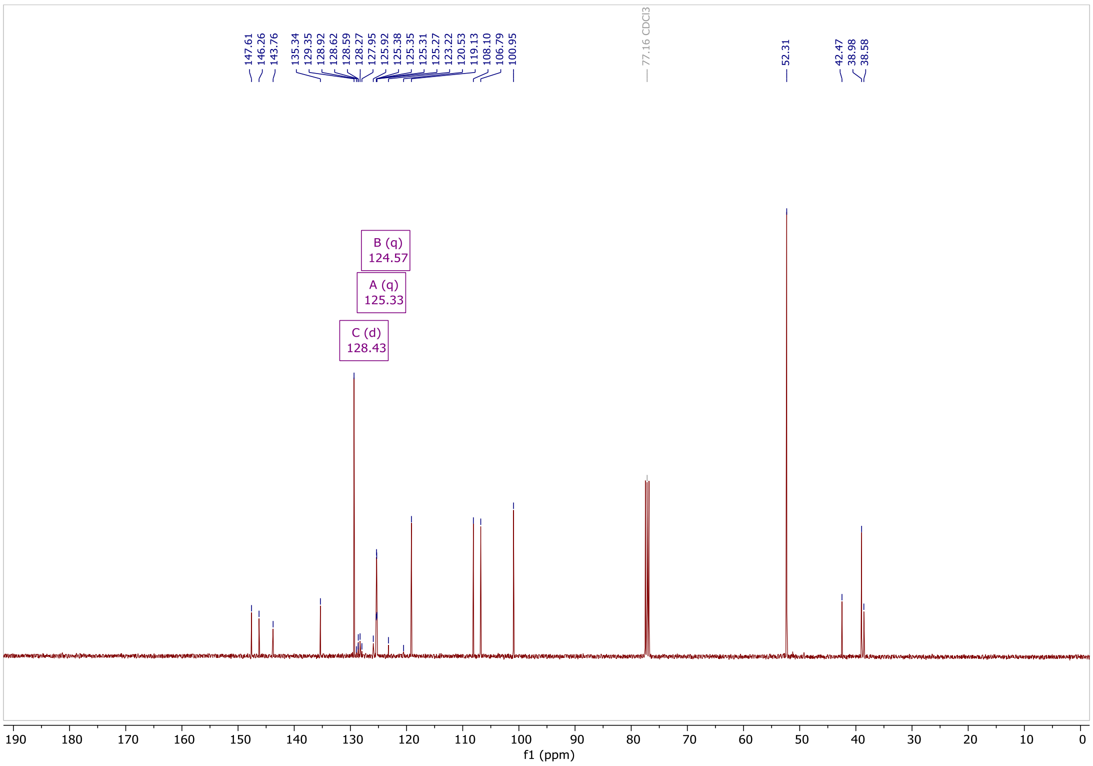

^{19}F NMR (376 MHz, $CDCl_3$)

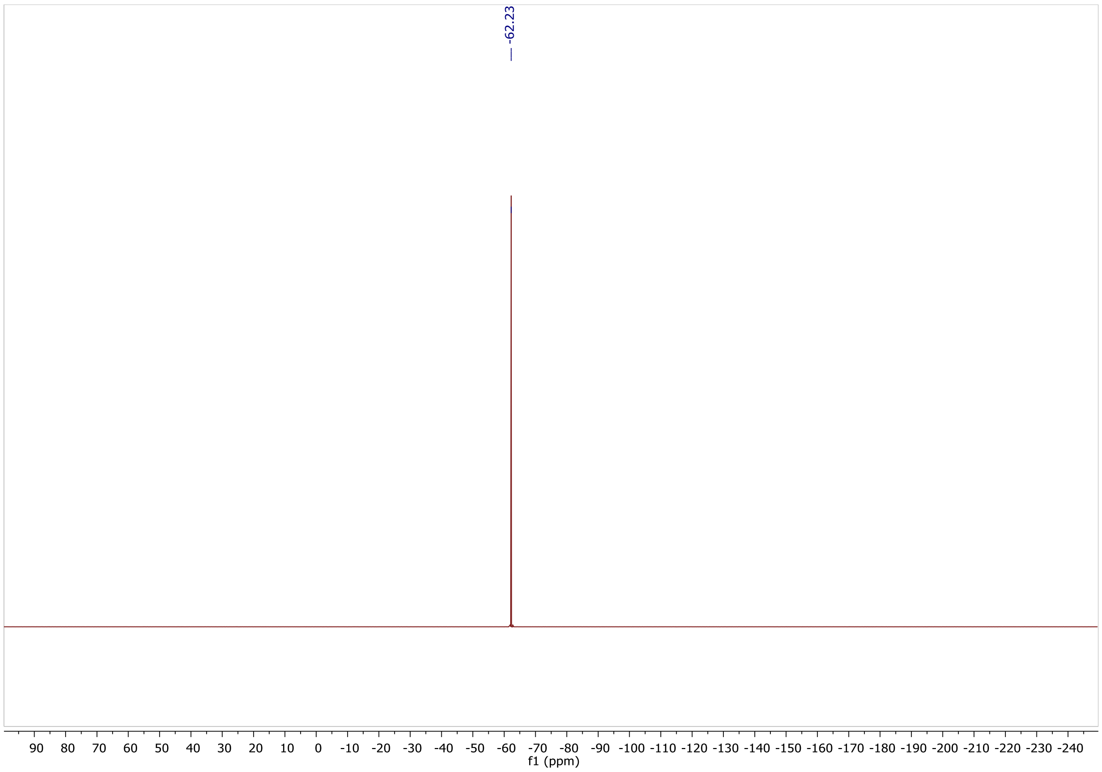

tert-Butyl 4-(3-(pyridin-3-yl)bicyclo[1.1.1]pentan-1-yl)piperidine-1-carboxylate, 2v

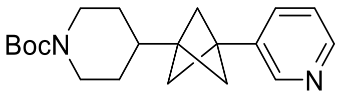

^{1}H NMR (400 MHz, $CDCl_3$)

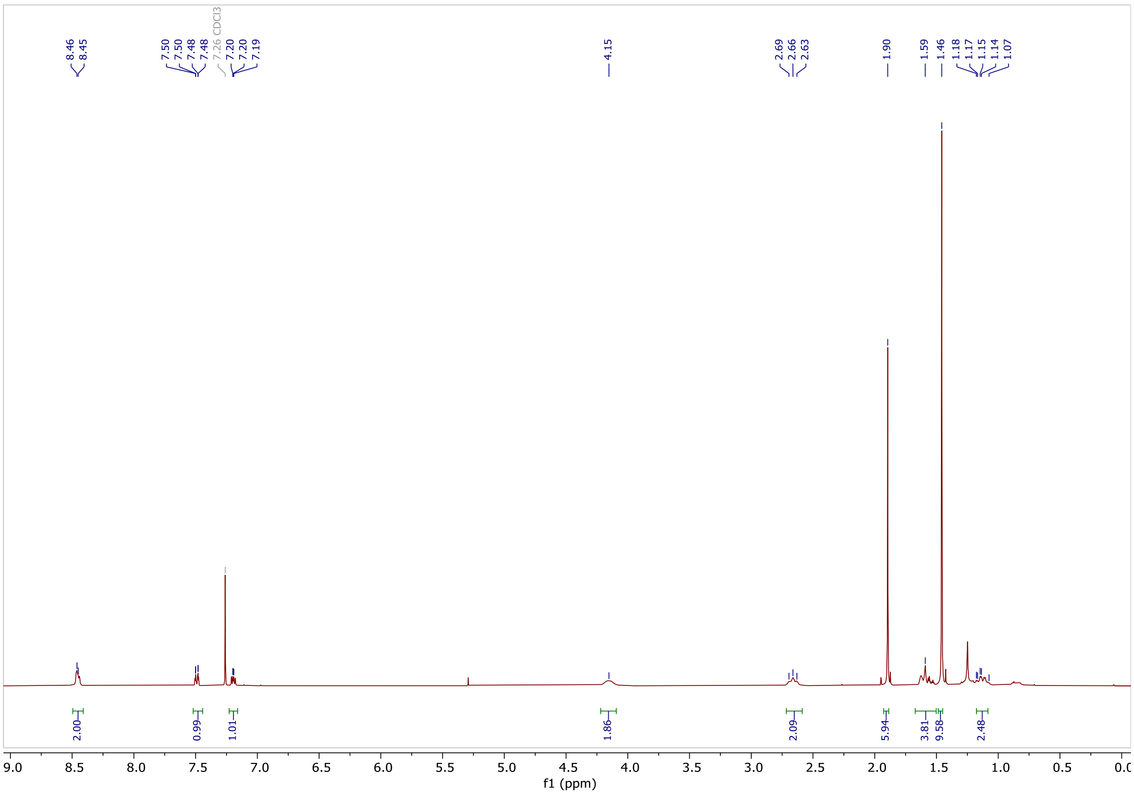

^{13}C NMR (101 MHz, $CDCl_3$)

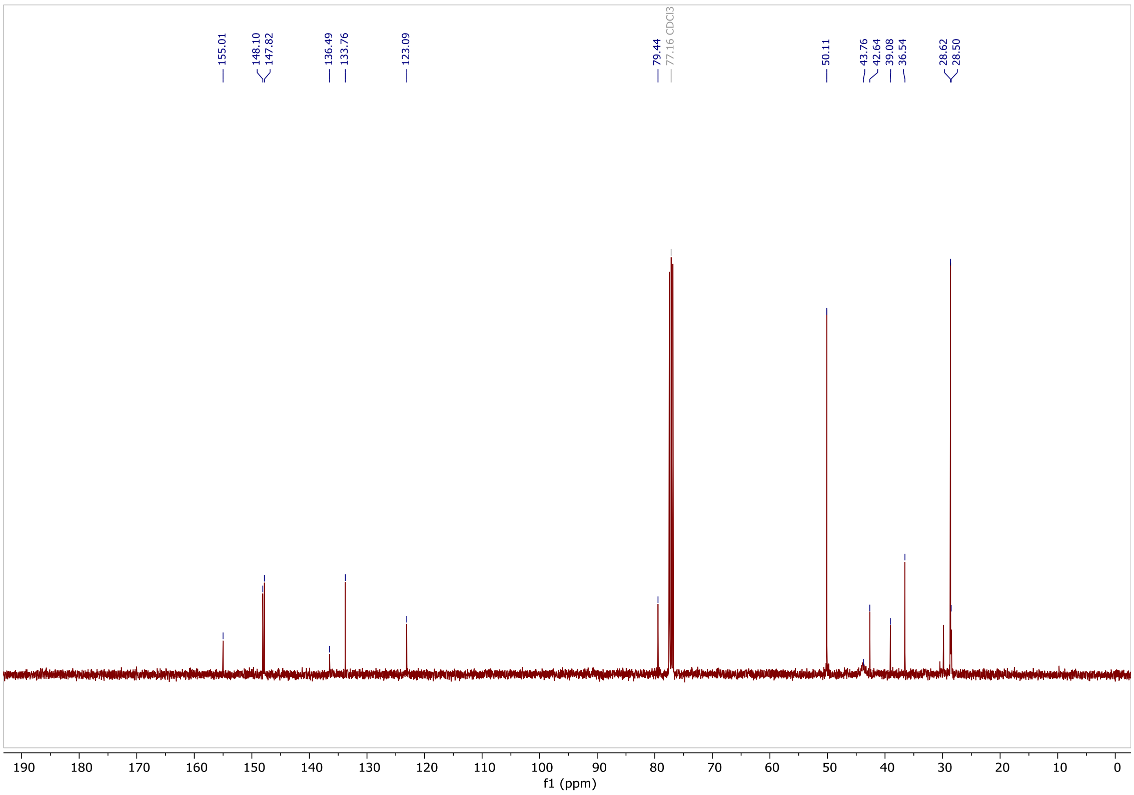

***tert*-butyl 4-(3-(6-ethoxypyridin-3-yl)bicyclo[1.1.1]pentan-1-yl)piperidine-1-carboxylate, 2w**

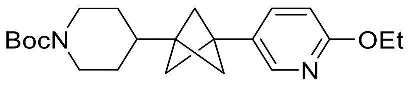

^{1}H NMR (400 MHz, $CDCl_3$)

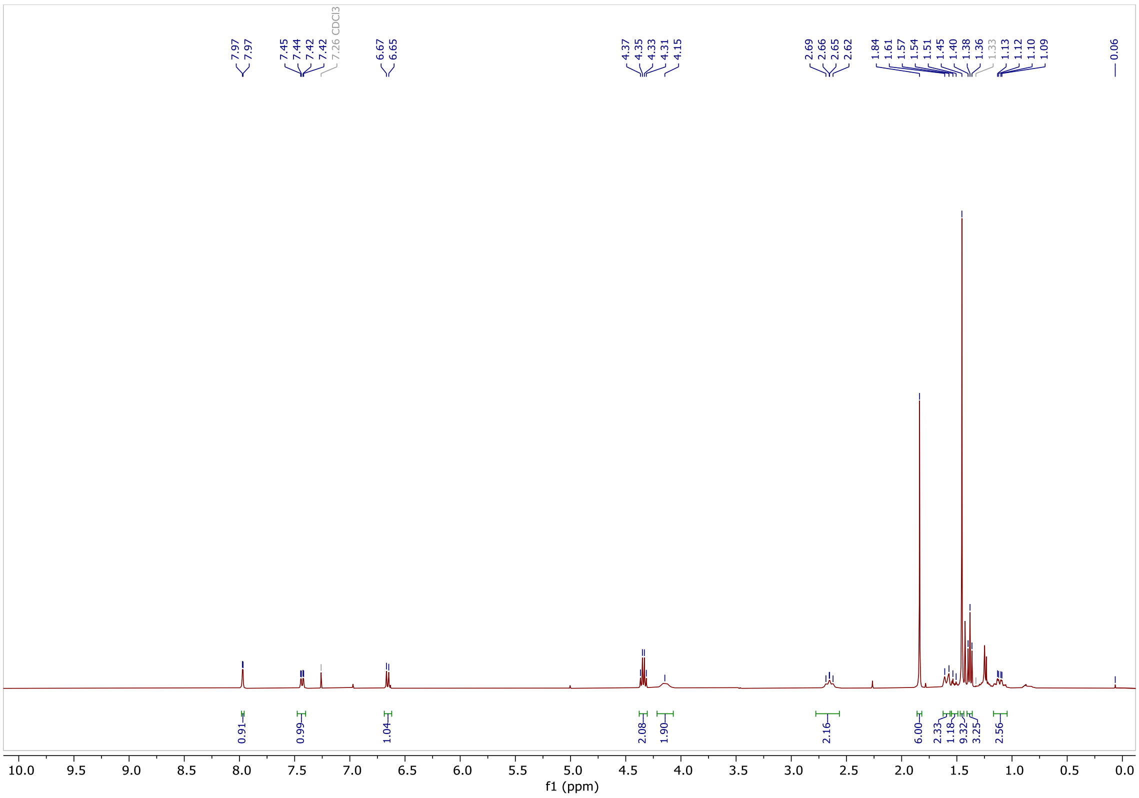

^{13}C NMR (126 MHz, $CDCl_3$)

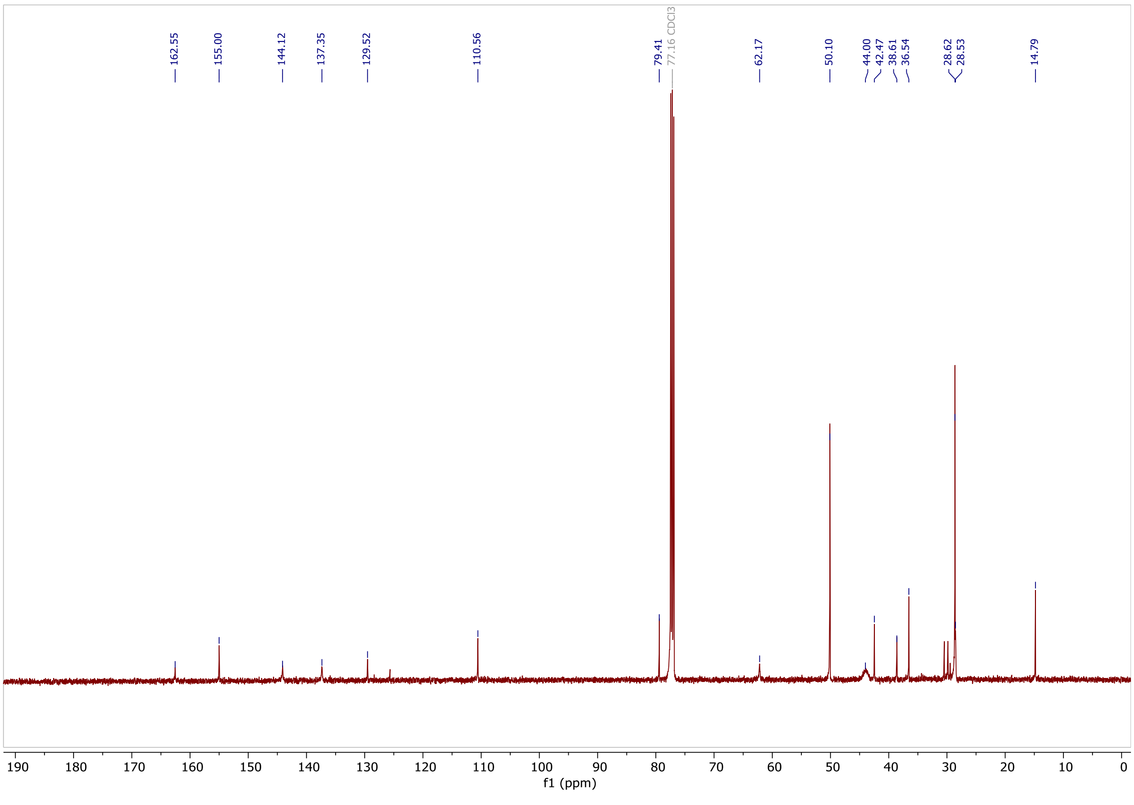

tert-Butyl 4-(3-(5-methoxypyridin-3-yl)bicyclo[1.1.1]pentan-1-yl)piperidine-1-carboxylate, 2x

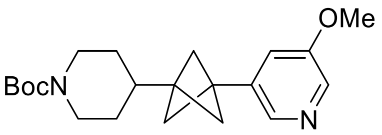

^{1}H NMR (400 MHz, $CDCl_3$)

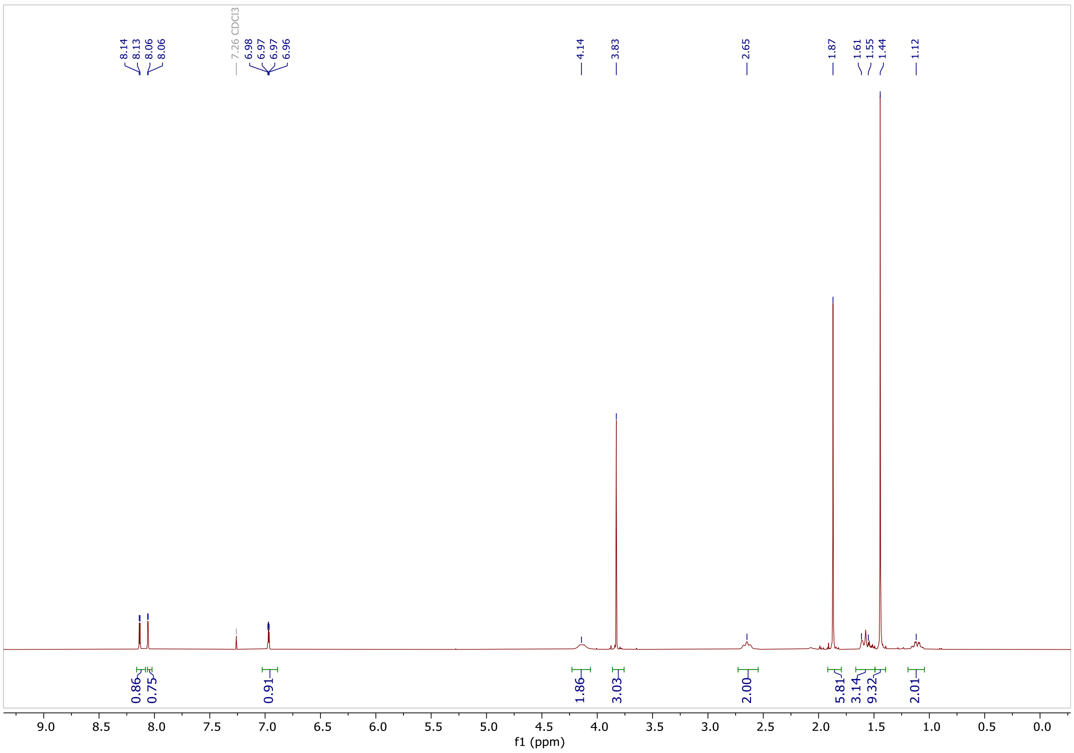

^{13}C NMR (101 MHz, $CDCl_3$)

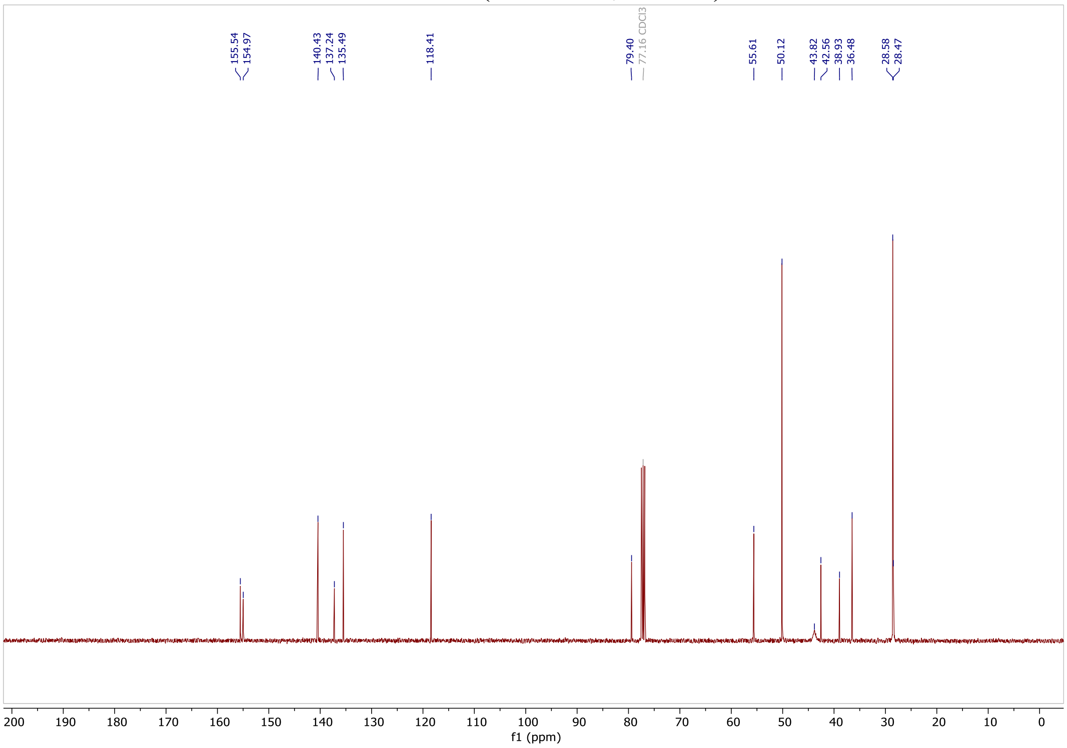

tert-butyl 4-(3-(2-methoxypyridin-4-yl)bicyclo[1.1.1]pentan-1-yl)piperidine-1-carboxylate, 2y

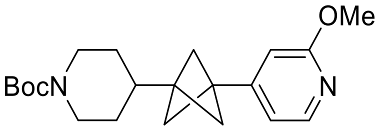

^{1}H NMR (400 MHz, $CDCl_3$)

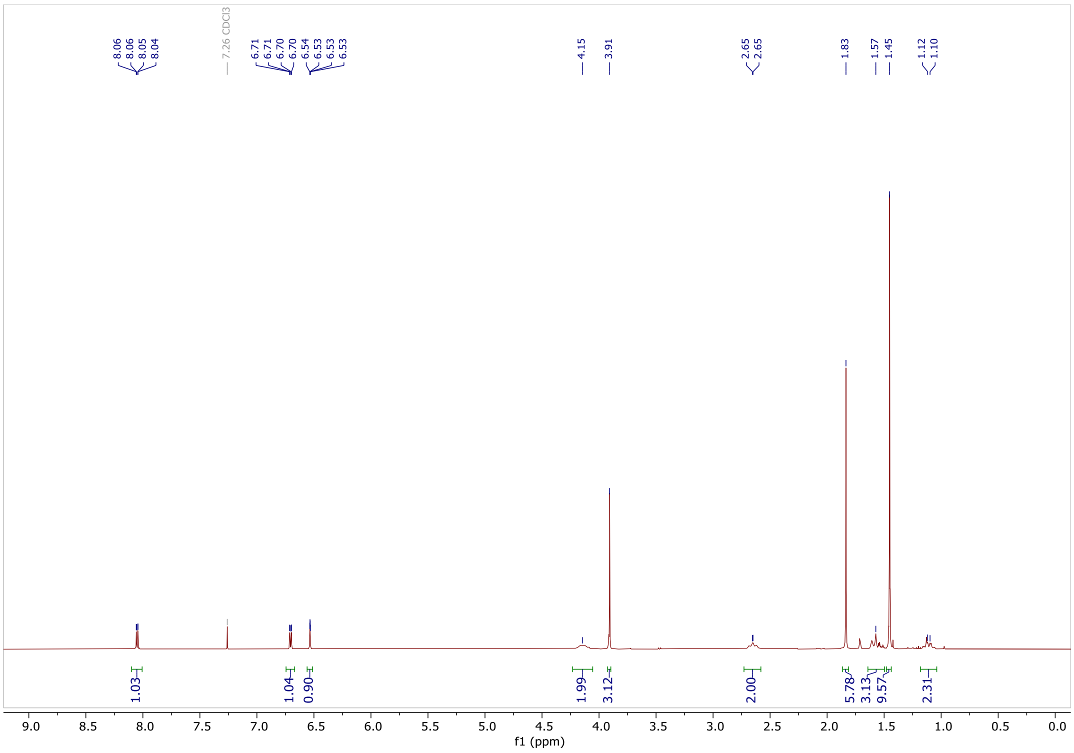

^{13}C NMR (101 MHz, $CDCl_3$)

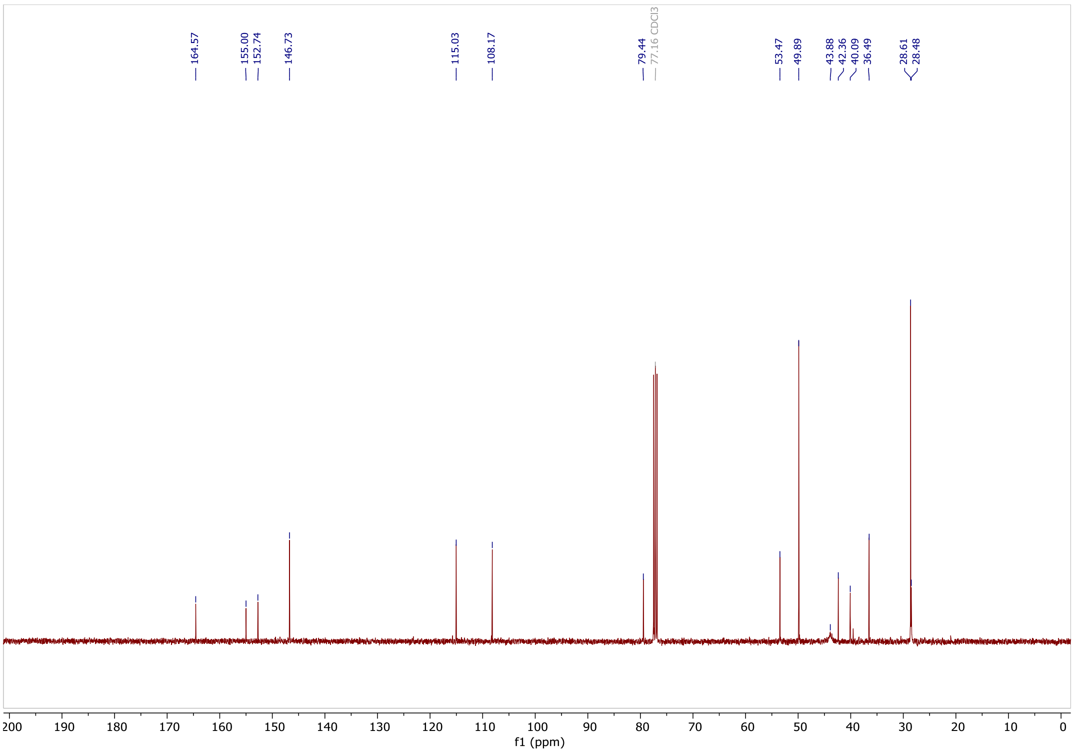

***tert*-Butyl 4-(3-(benzofuran-5-yl)bicyclo[1.1.1]pentan-1-yl)piperidine-1-carboxylate, 2z**

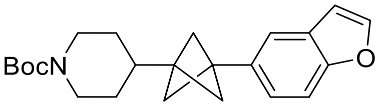

^{1}H NMR (400 MHz, $CDCl_3$)

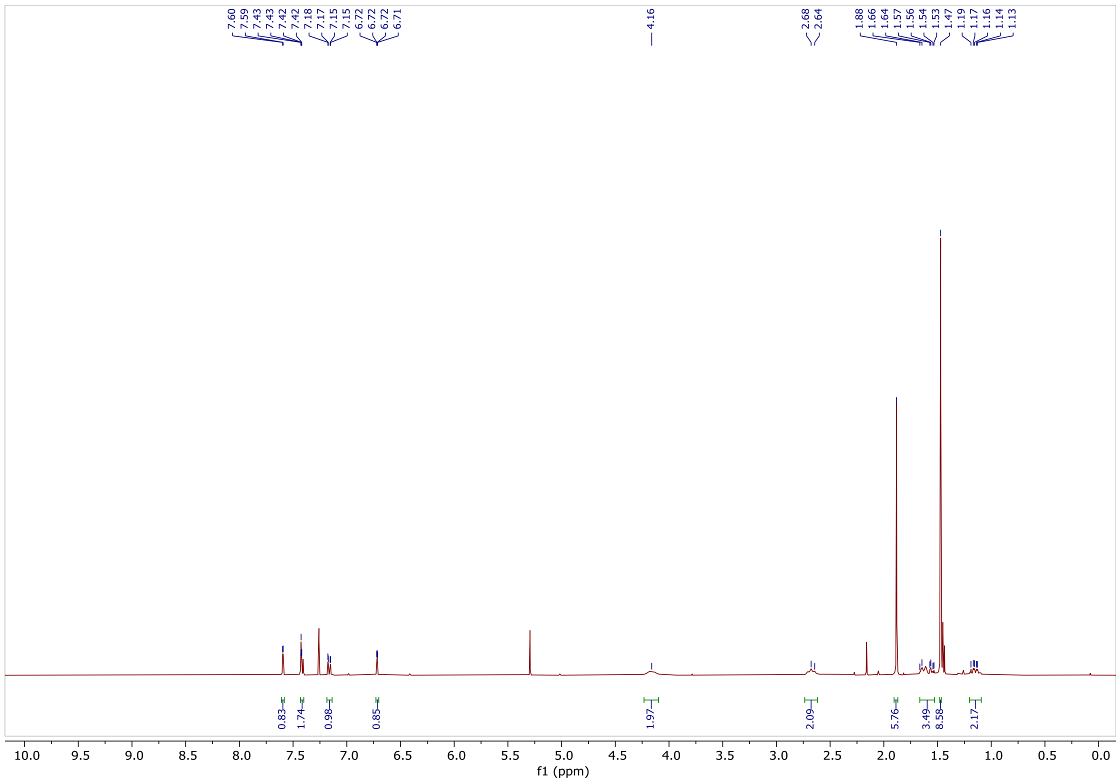

^{13}C NMR (101 MHz, $CDCl_3$)

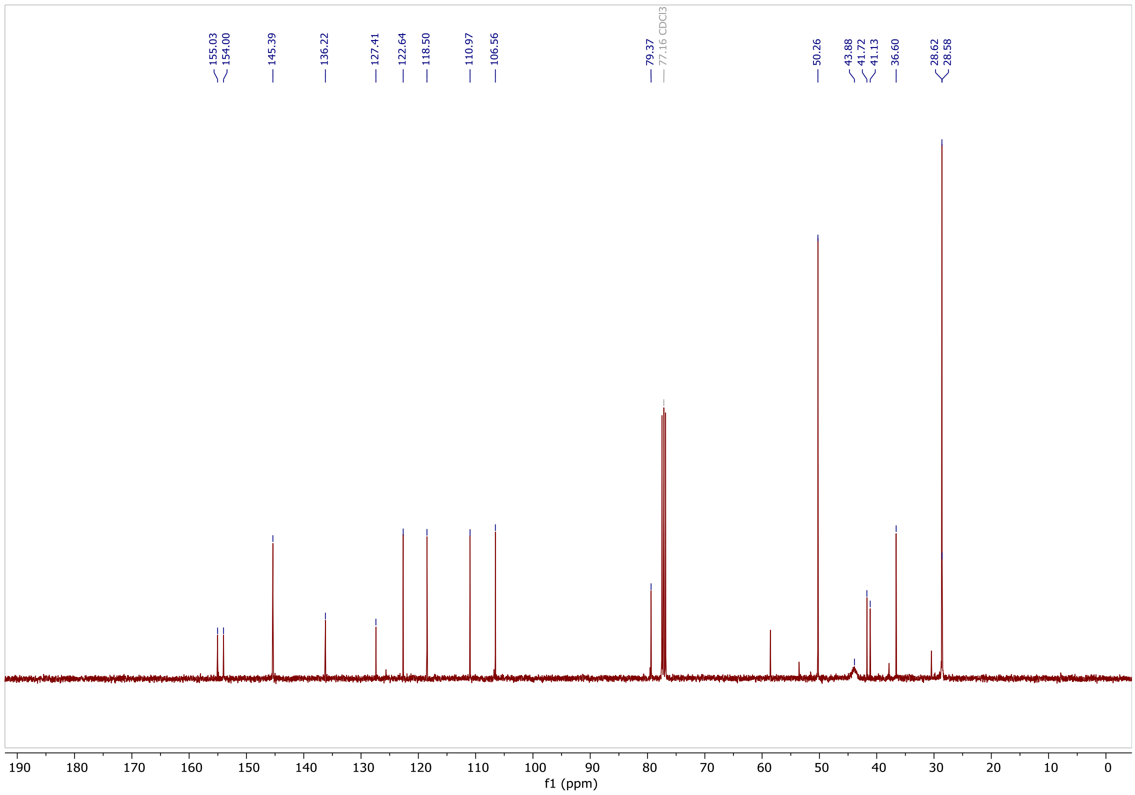

tert-butyl 4-(3-(4-(trifluoromethyl)benzyl)bicyclo[1.1.1]pentan-1-yl)indoline-1-carboxylate, 2aa

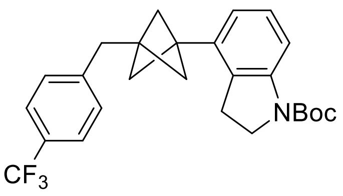

^{1}H NMR (400 MHz, $CDCl_3$)

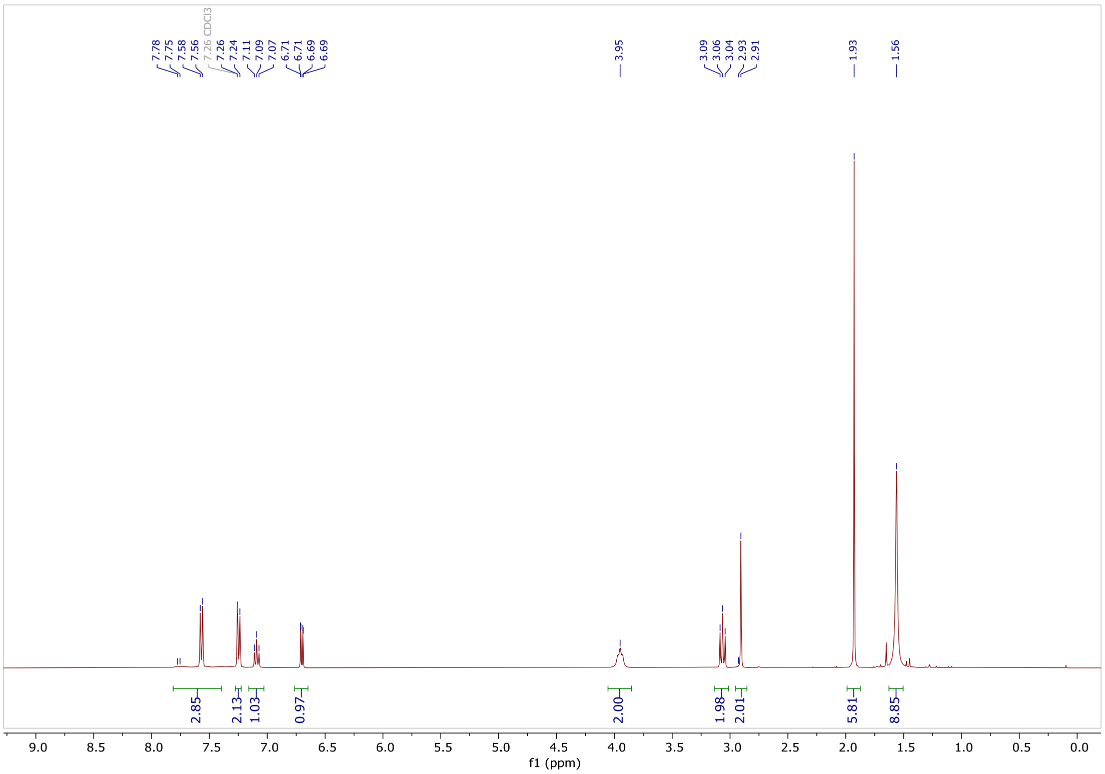

^{13}C {^{19}F} NMR (125 MHz, $CDCl_3$)

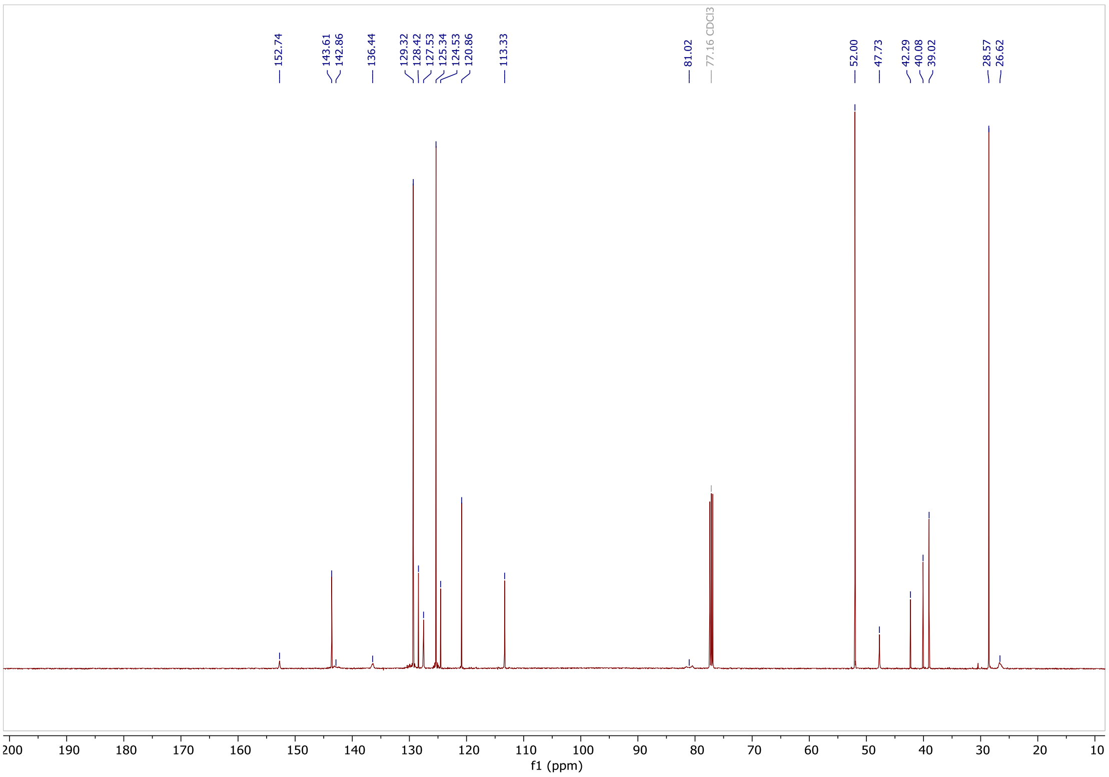

***tert*-butyl 5-(3-(1-(tert-butoxycarbonyl)piperidin-4-yl)bicyclo[1.1.1]pentan-1-yl)-1*H*-indole-1-carboxylate, 2ab**

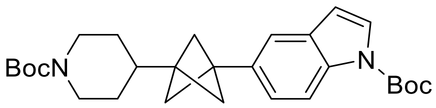

^{1}H NMR (400 MHz, $CDCl_3$)

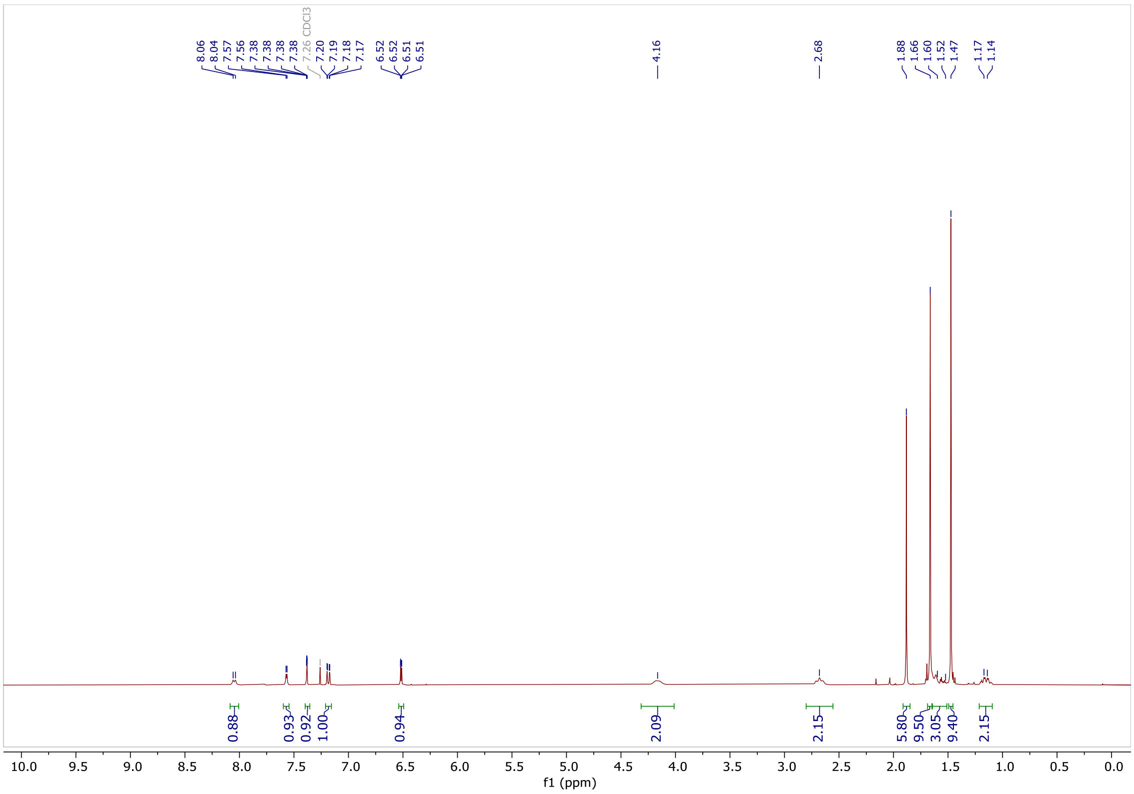

^{13}C NMR (101 MHz, $CDCl_3$)

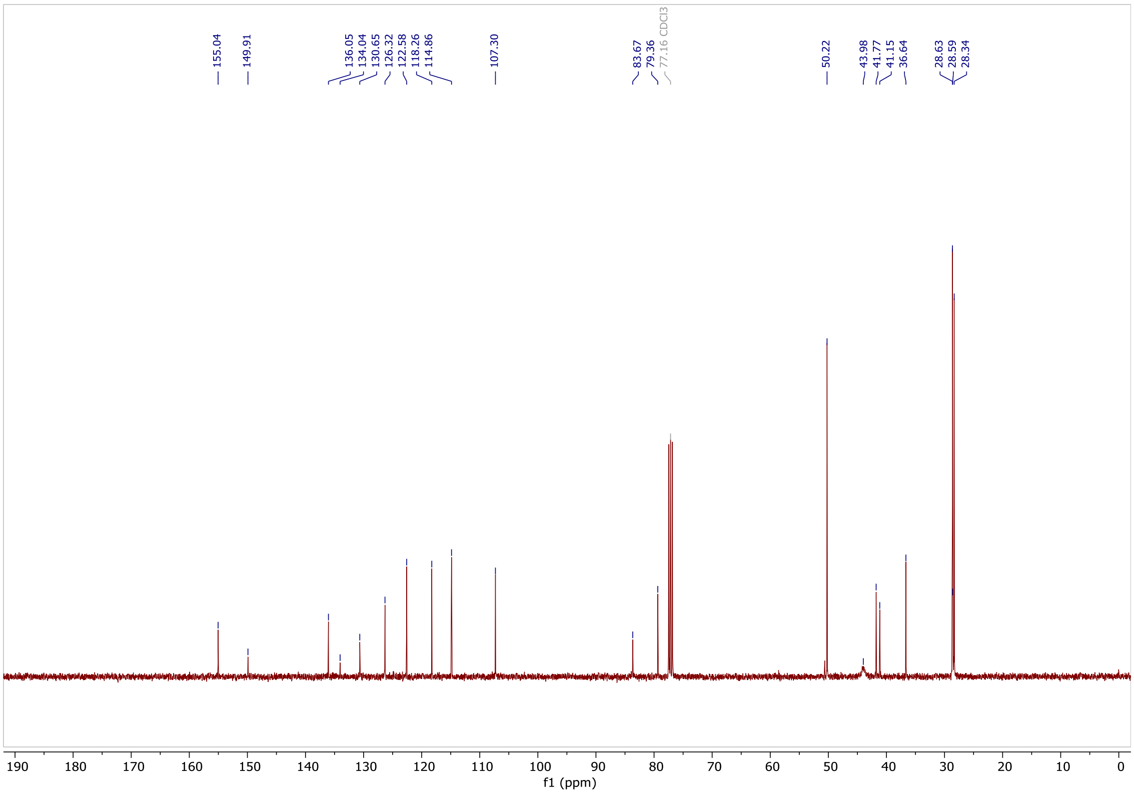

***tert*-Butyl 4-(3-(1-methyl-1*H*-indazol-5-yl)bicyclo[1.1.1]pentan-1-yl)piperidine-1-carboxylate, 2ac**

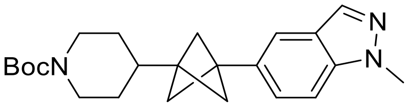

^{1}H NMR (400 MHz, $CDCl_3$)

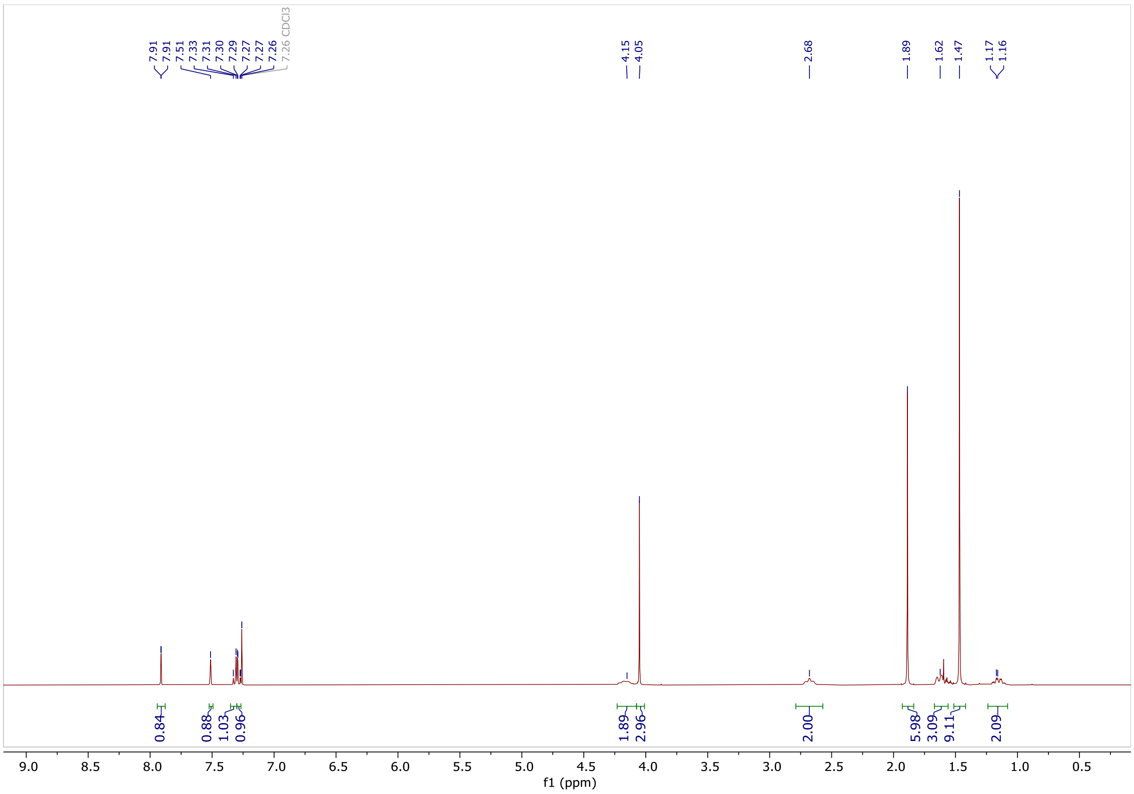

^{13}C NMR (101 MHz, $CDCl_3$)

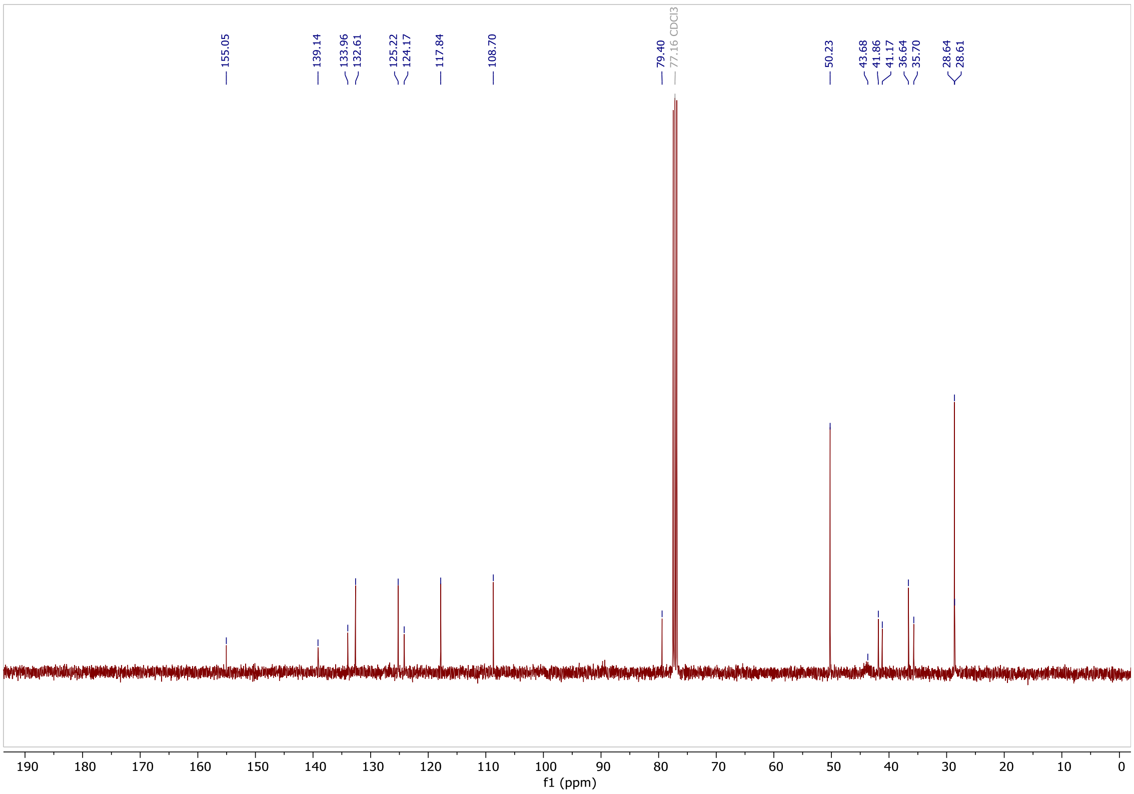

tert-butyl 4-(3-(9-phenyl-_9H_-carbazol-3-yl)bicyclo[1.1.1]pentan-1-yl)piperidine-1-carboxylate, 2ad

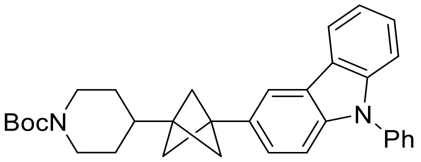

^{1}H NMR (400 MHz, $CDCl_3$)

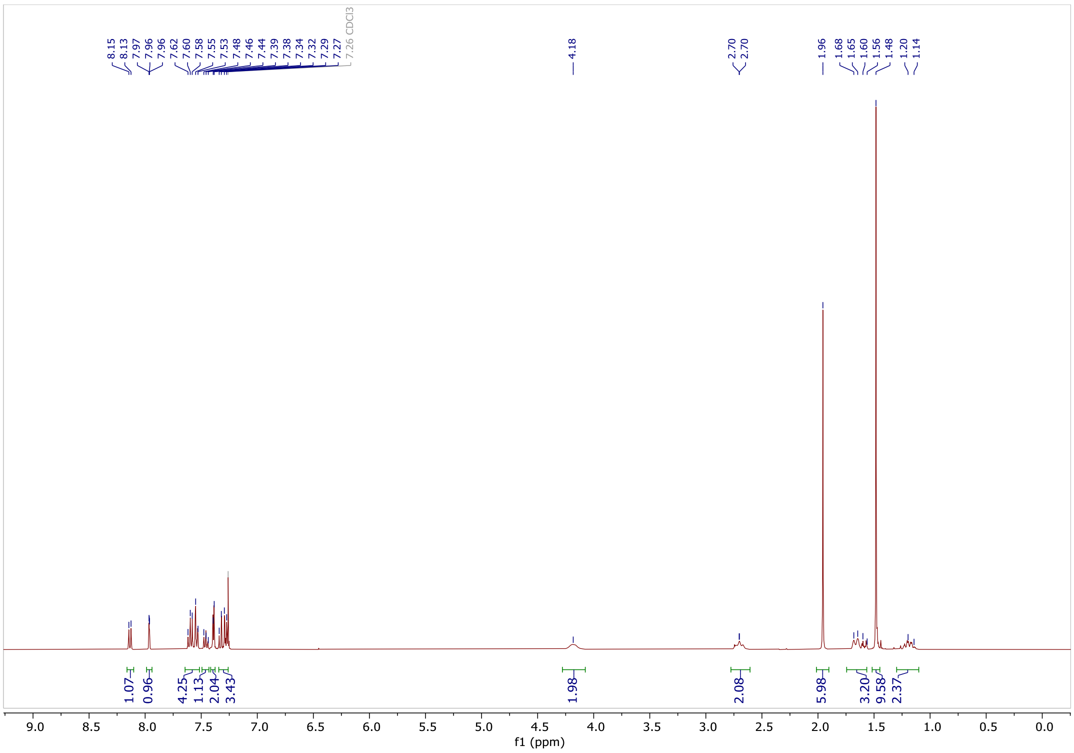

^{13}C NMR (101 MHz, $CDCl_3$)

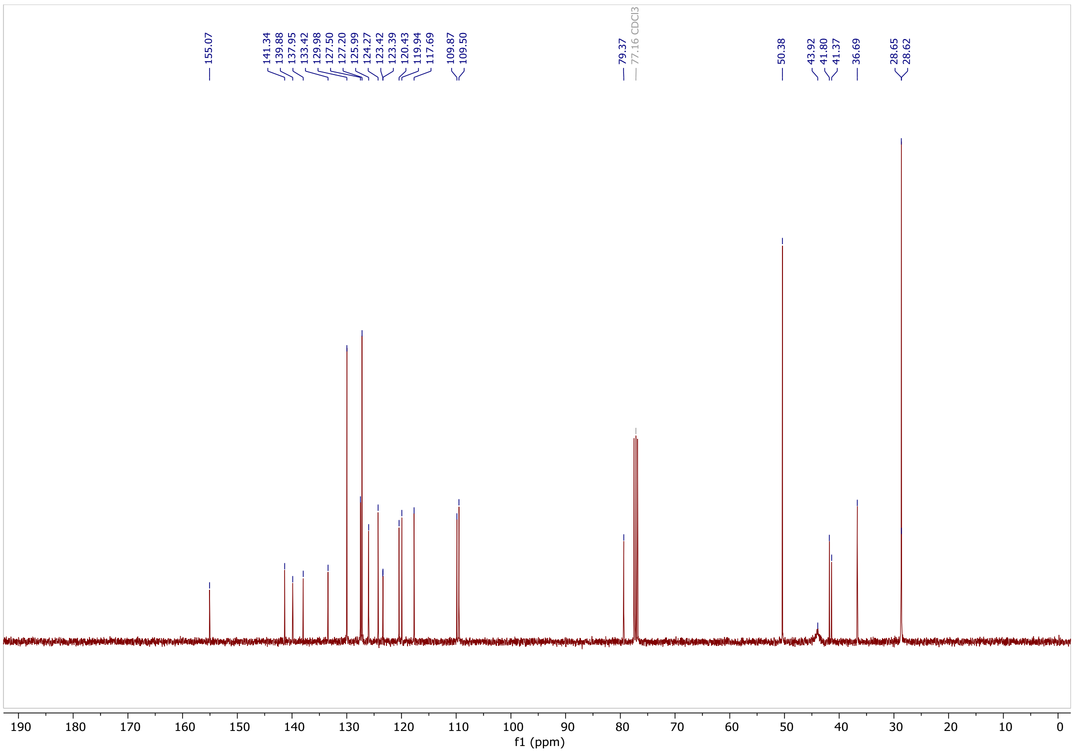

tert-Butyl 3-(3-(4-methoxyphenyl)bicyclo[1.1.1]pentan-1-yl)azetidine-1-carboxylate, 2ae

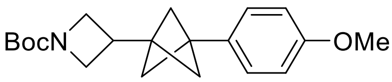

^{1}H NMR (400 MHz, $CDCl_3$)

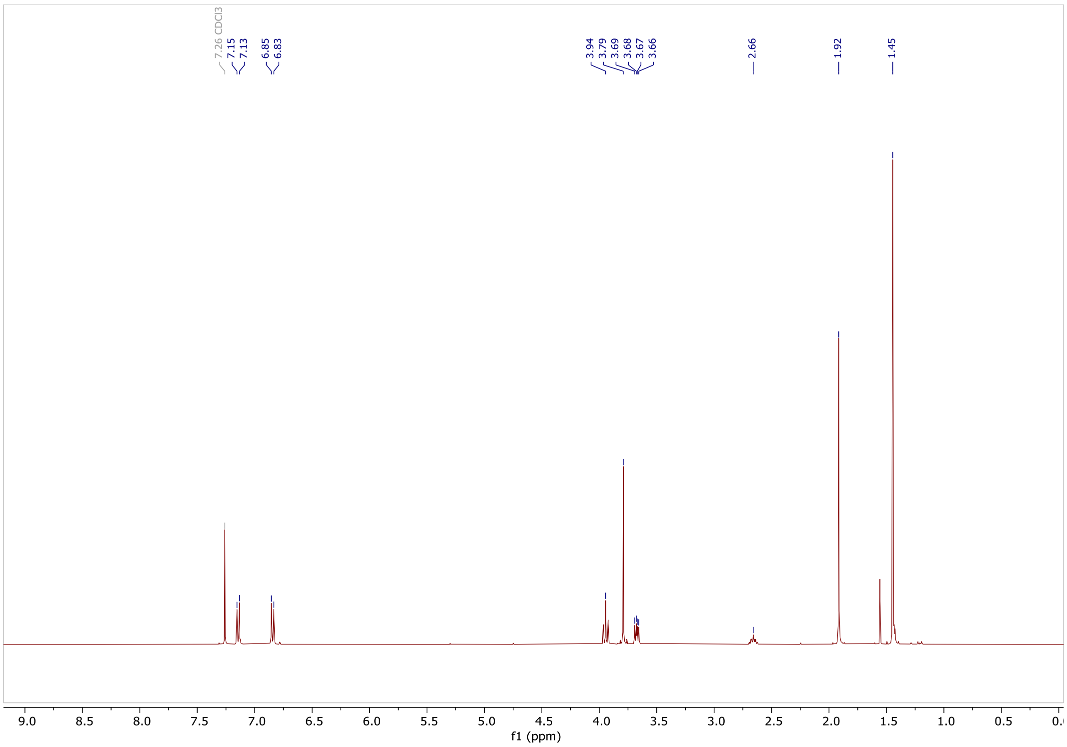

^{13}C NMR (101 MHz, $CDCl_3$)

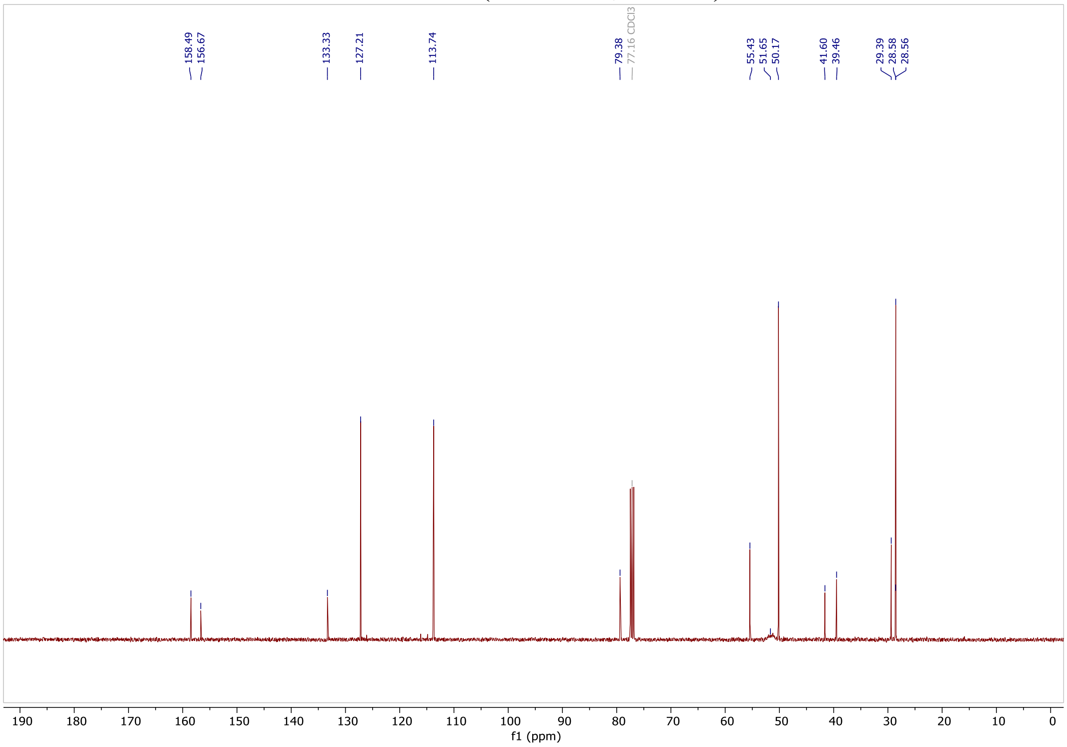

1-(4-Methoxyphenyl)-3-((phenylsulfonyl)methyl)bicyclo[1.1.1]pentane, 2af

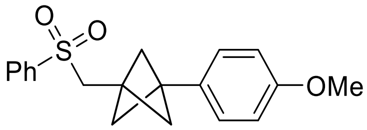

^{1}H NMR (400 MHz, $CDCl_3$)

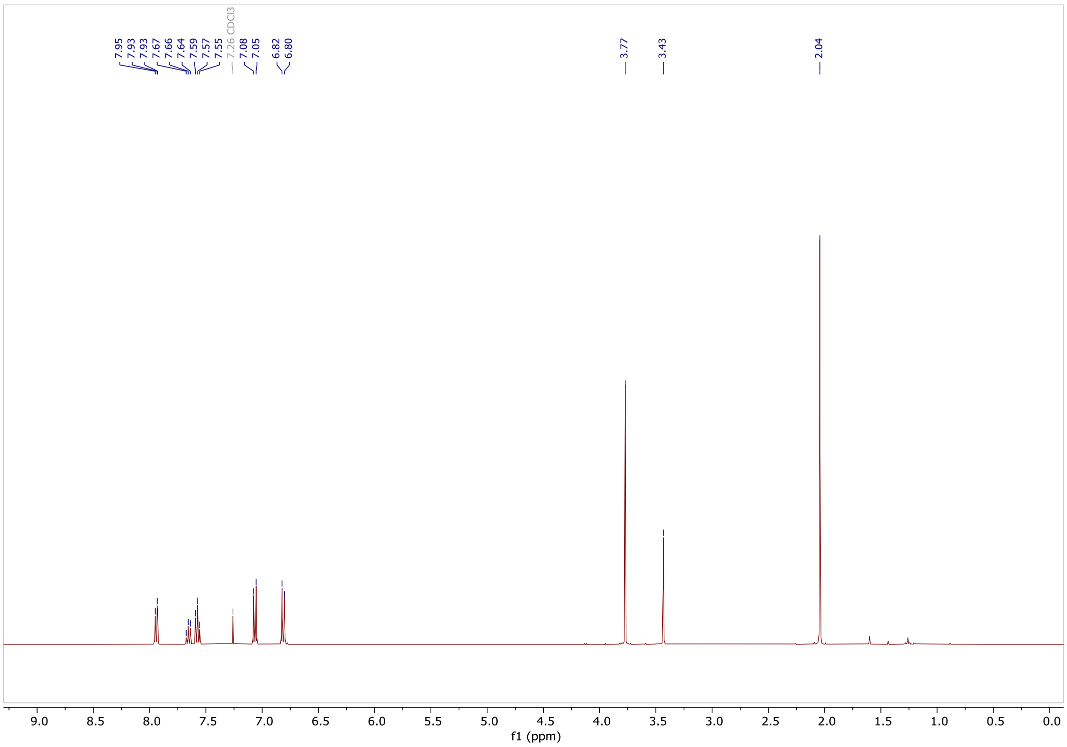

^{13}C NMR (101 MHz, $CDCl_3$)

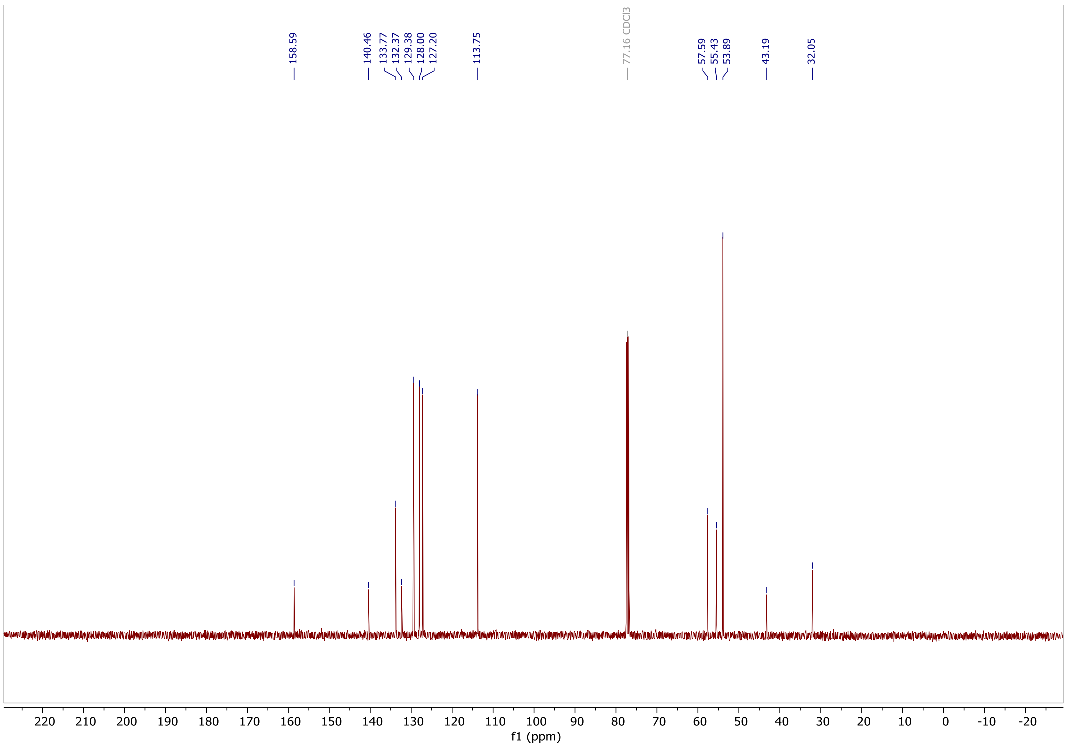

Ethyl 2-(3-(4-methoxyphenyl)bicyclo[1.1.1]pentan-1-yl)acetate, 2ag

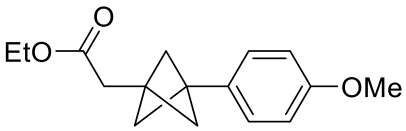

^{1}H NMR (400 MHz, $CDCl_3$)

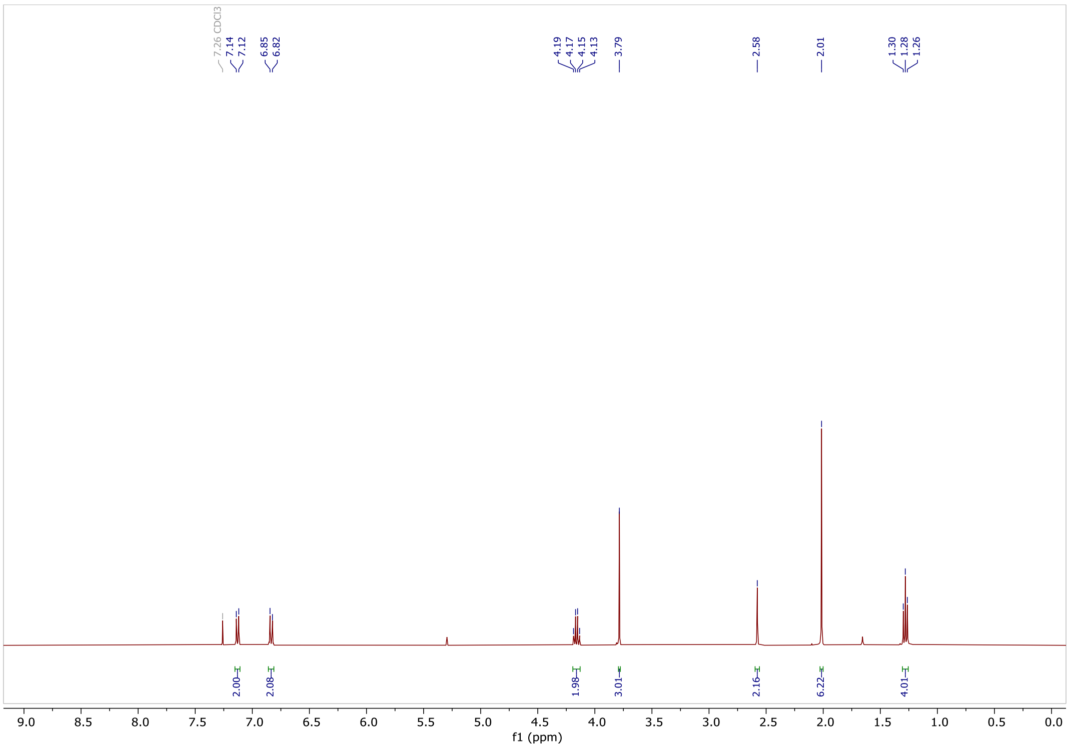

^{13}C NMR (101 MHz, $CDCl_3$)

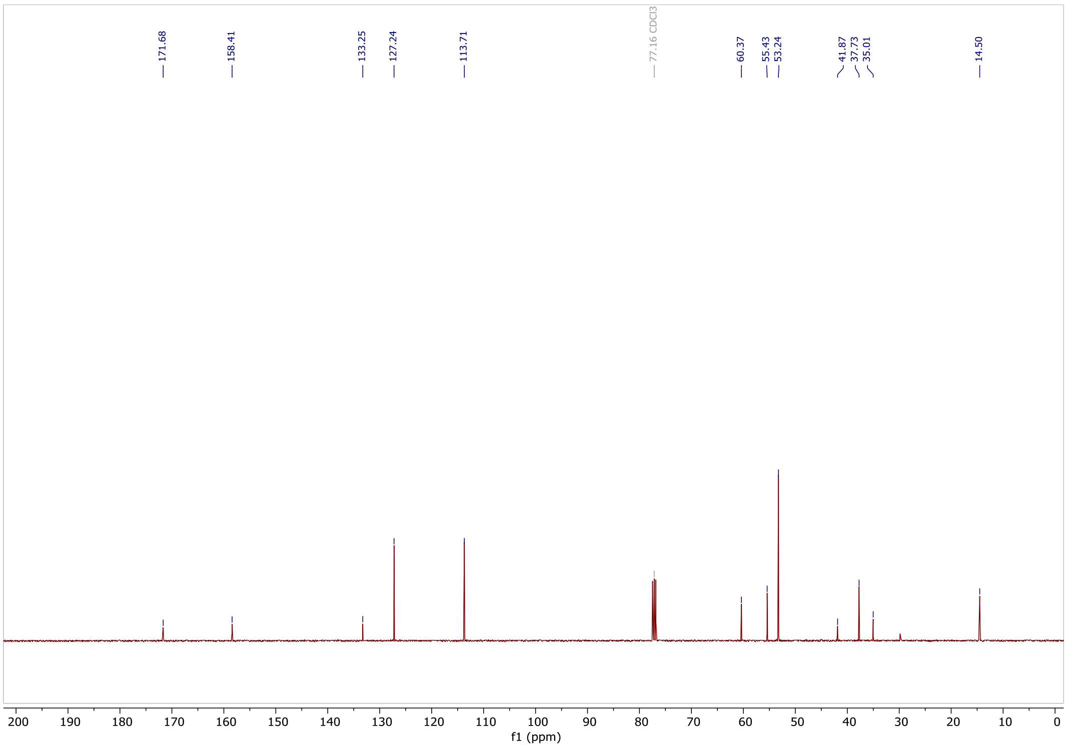

Ethyl 2,2-difluoro-2-(3-(4-methoxyphenyl)bicyclo[1.1.1]pentan-1-yl)acetate, 2ah

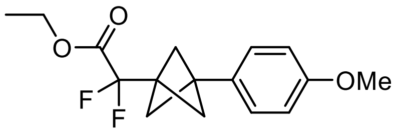

^{1}H NMR (400 MHz, $CDCl_3$)

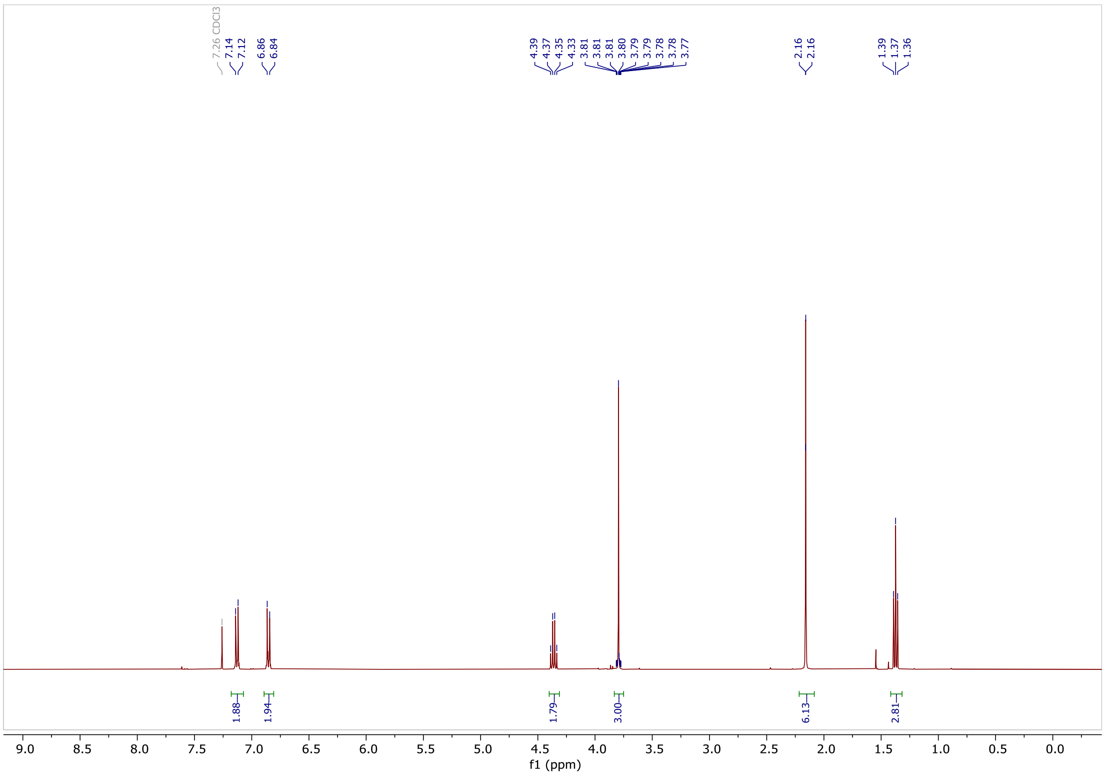

^{13}C NMR (101 MHz, $CDCl_3$)

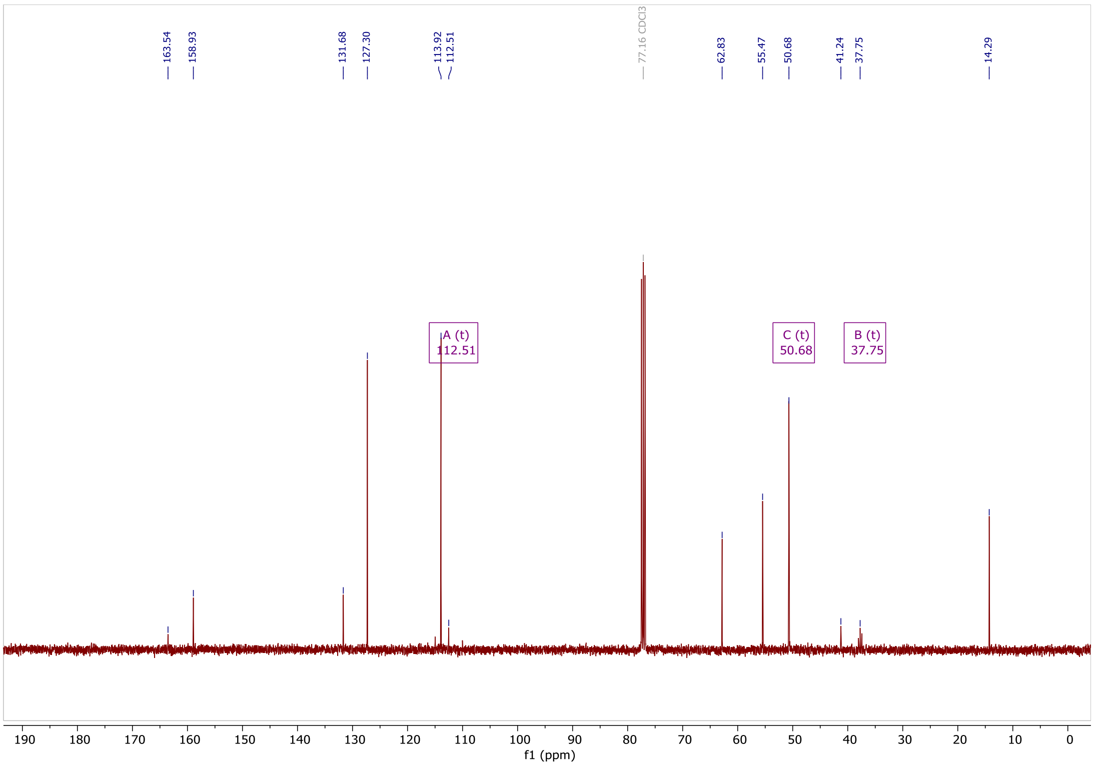

^{19}F NMR (376 MHz, $CDCl_3$)

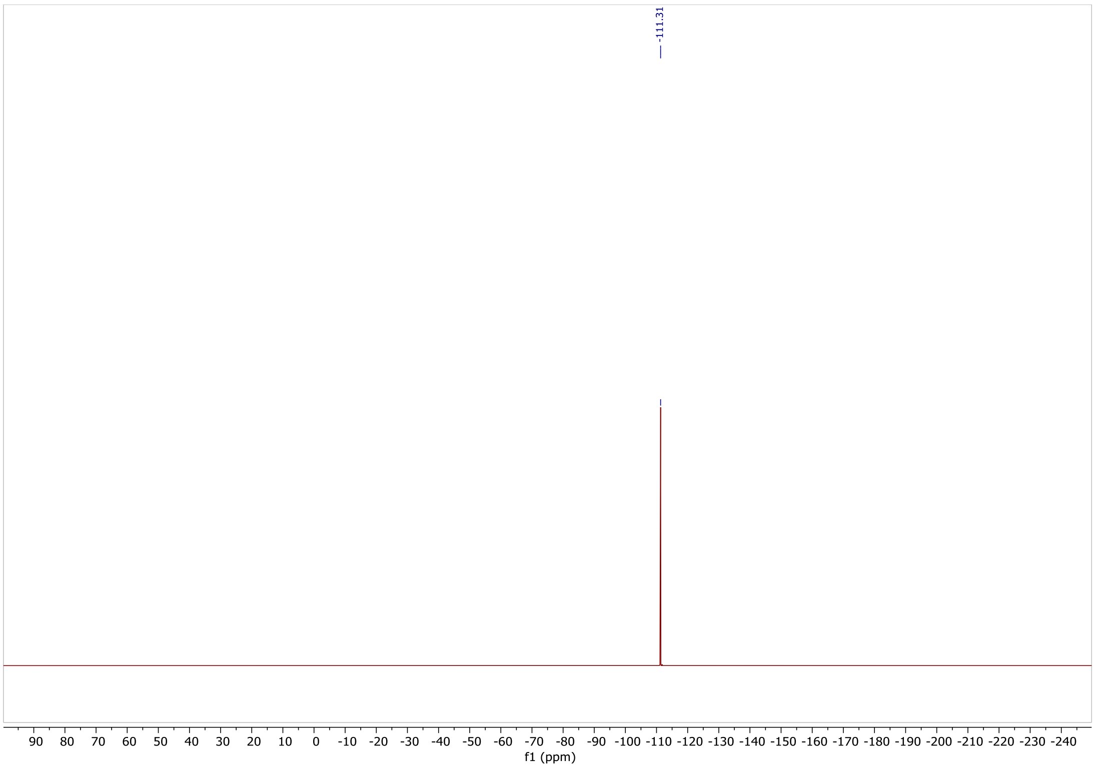

2-(3-(4-Methoxyphenyl)bicyclo[1.1.1]pentan-1-yl)pyridine, 2ai

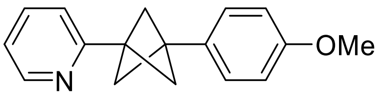

^{1}H NMR (400 MHz, $CDCl_3$)

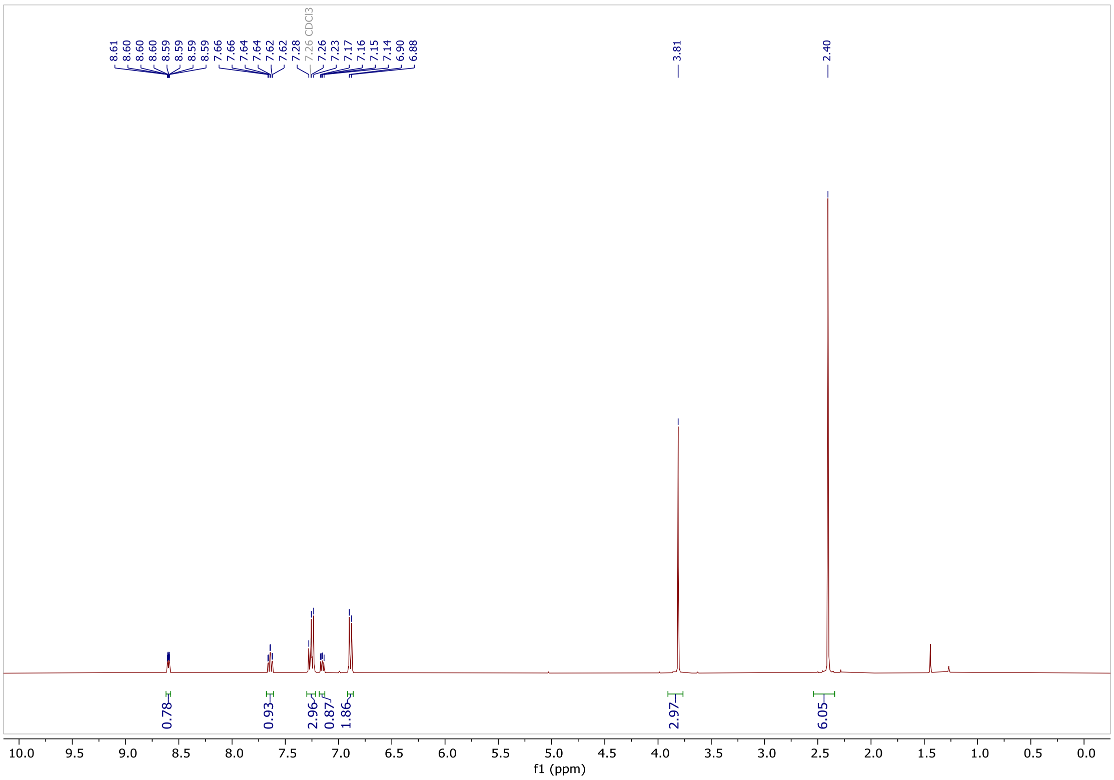

^{13}C NMR (101 MHz, $CDCl_3$)

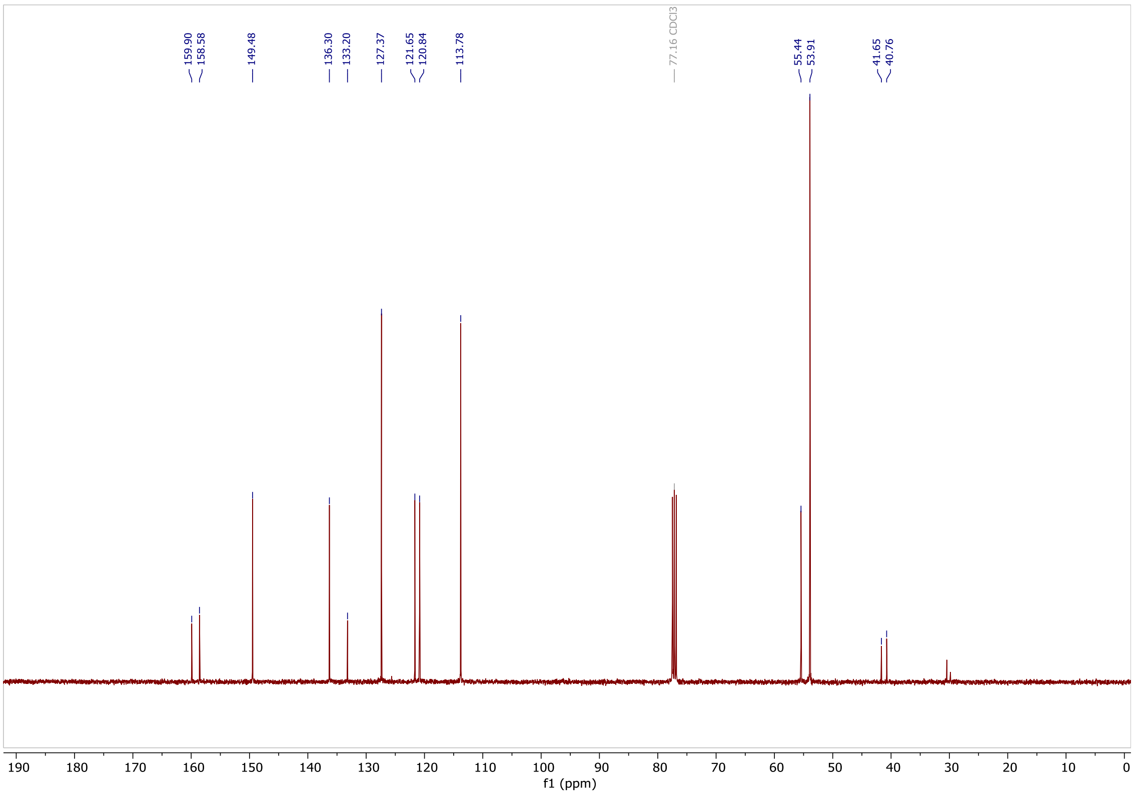

2,6-Bis(3-(4-methoxyphenyl)bicyclo[1.1.1]pentan-1-yl)pyridine, 2aj

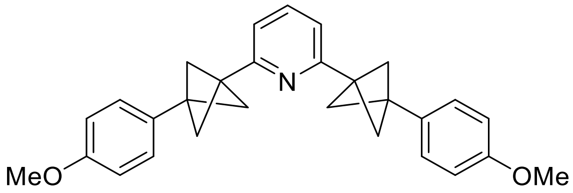

^{1}H NMR (400 MHz, $CDCl_3$)

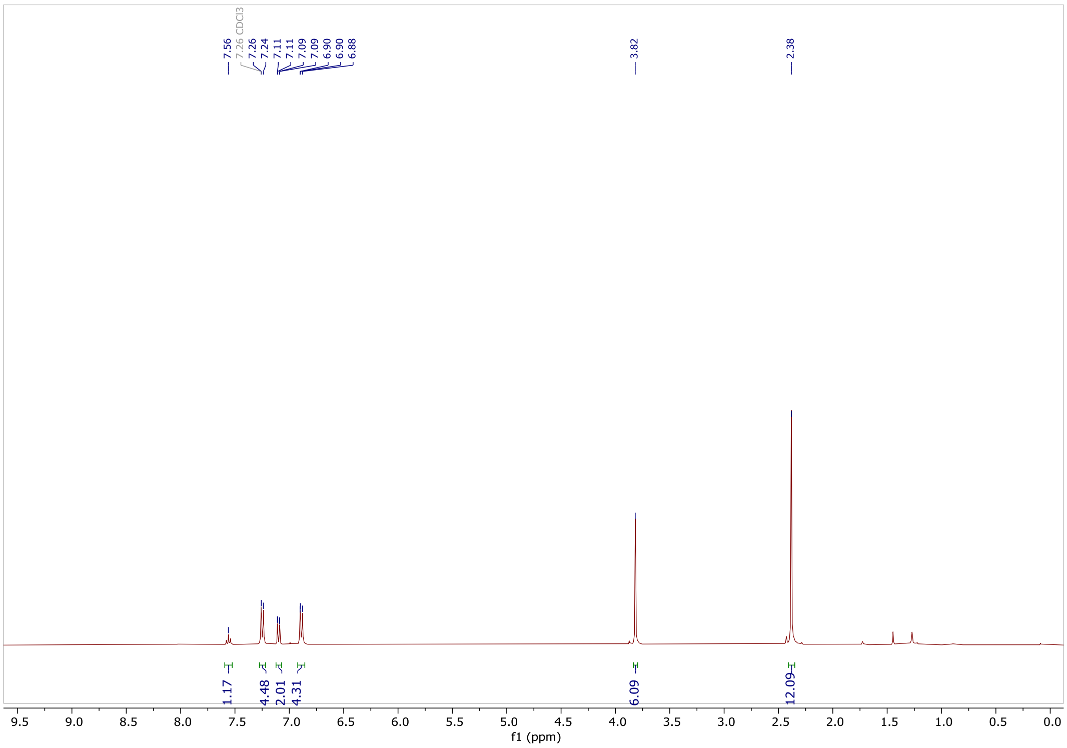

^{13}C NMR (101 MHz, $CDCl_3$)

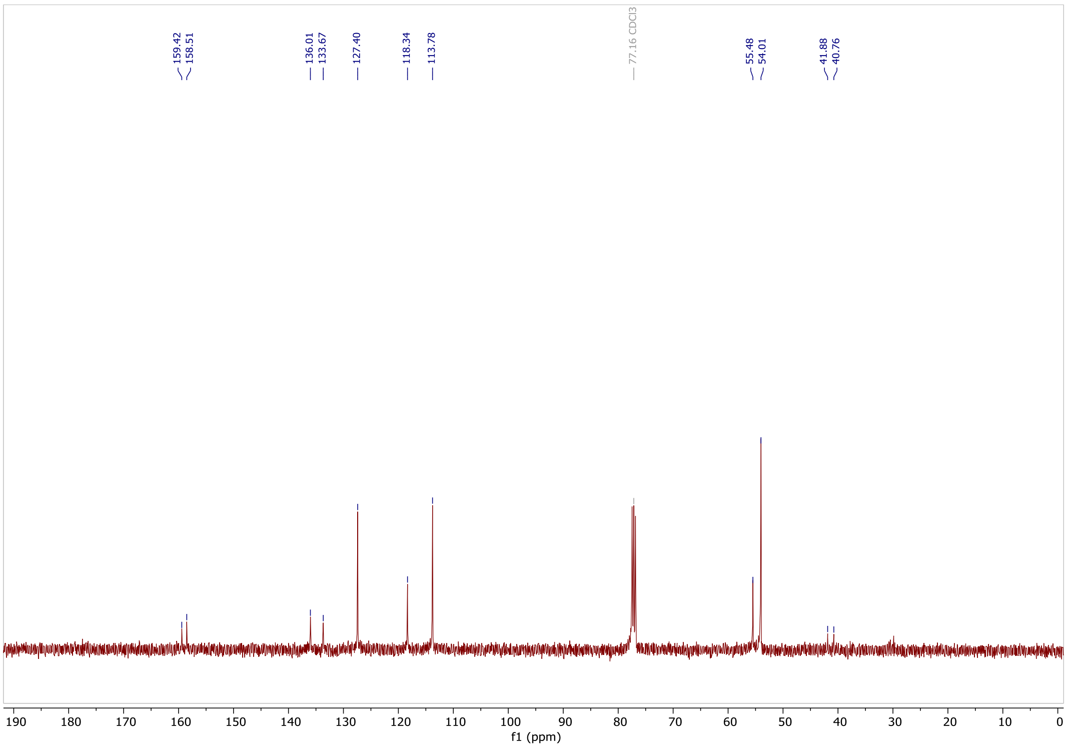

Methyl 6-fluoro-2-(3-(4-methoxyphenyl)bicyclo[1.1.1]pentan-1-yl)-3-methylquinoline-4-carboxylate, 2ak

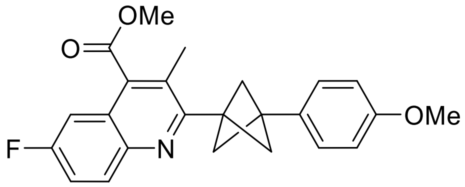

^{1}H NMR (400 MHz, $CDCl_3$)

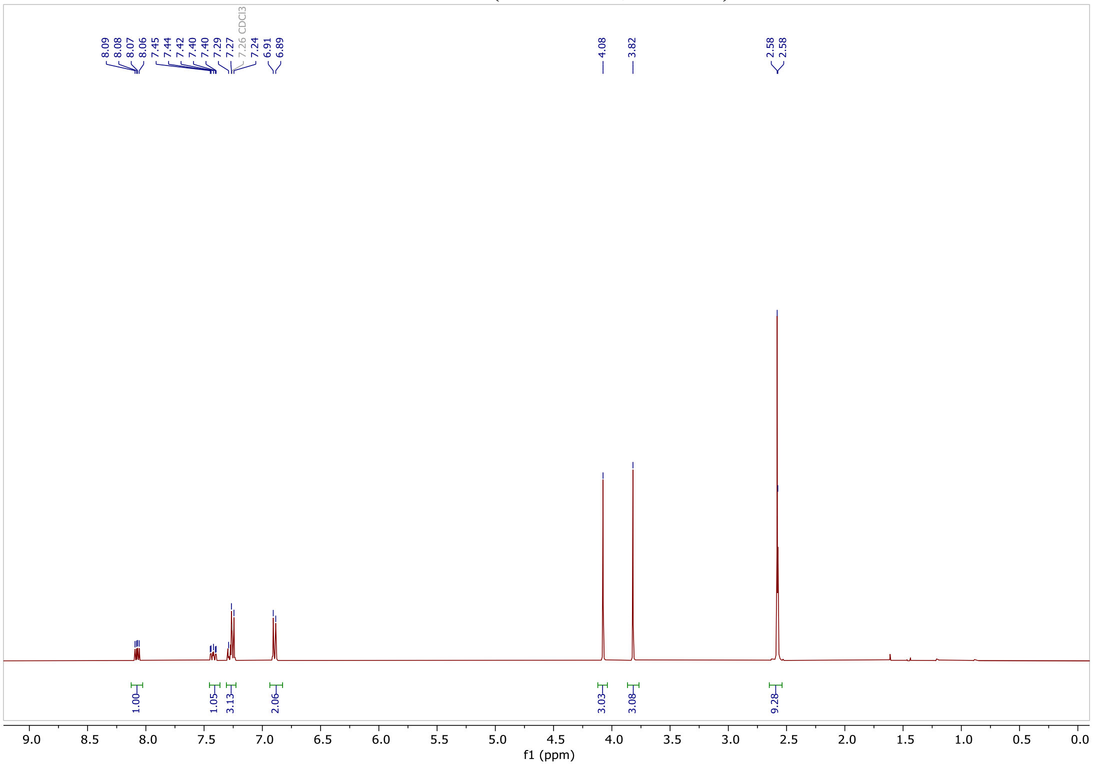

^{13}C NMR (101 MHz, $CDCl_3$)

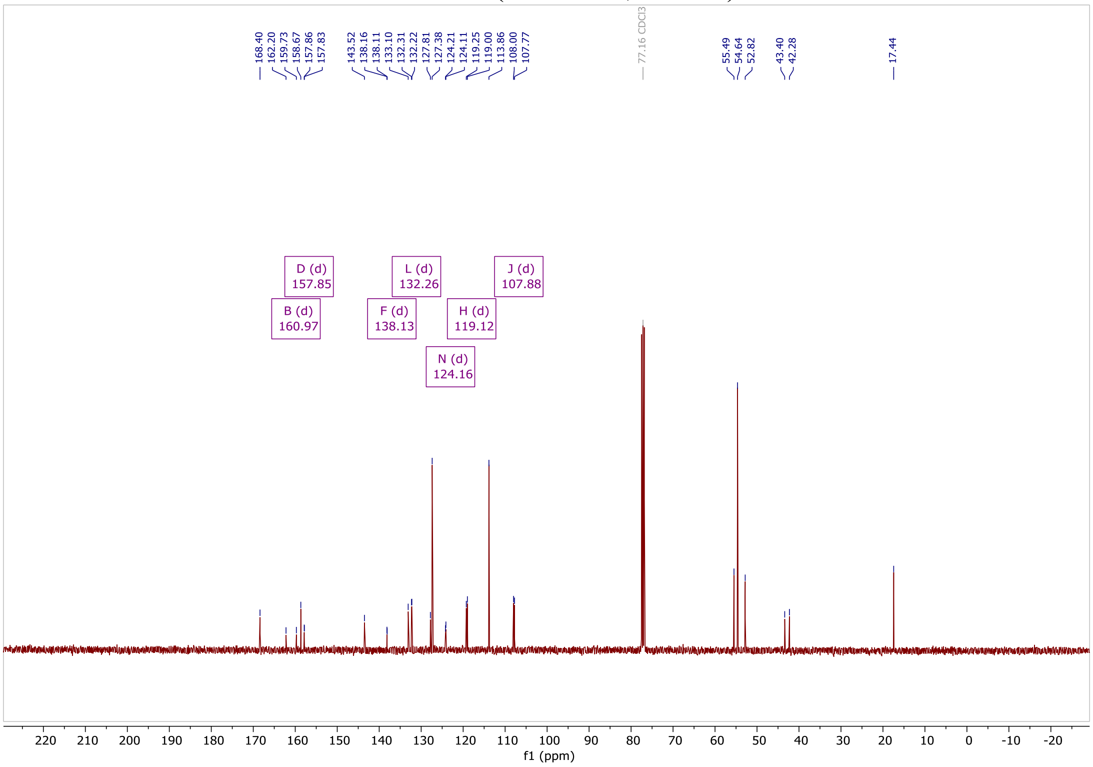

^{19}F NMR (376 MHz, $CDCl_3$)

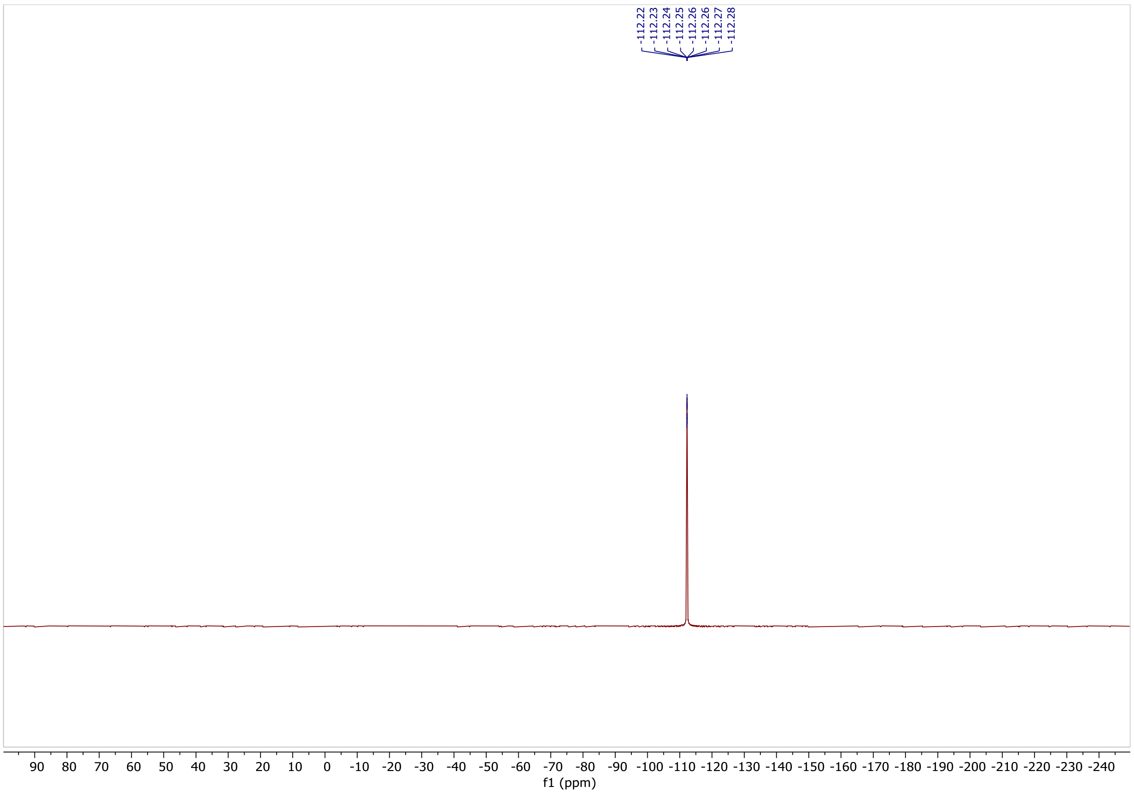

2-(3-(4-methoxyphenyl)bicyclo[1.1.1]pentan-1-yl)ethyl 6-(3-methoxyphenyl)-2-methylnicotinate, 2al

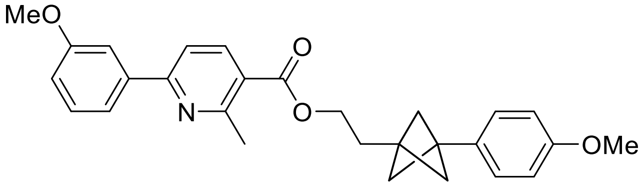

^{1}H NMR (400 MHz, $CDCl_3$)

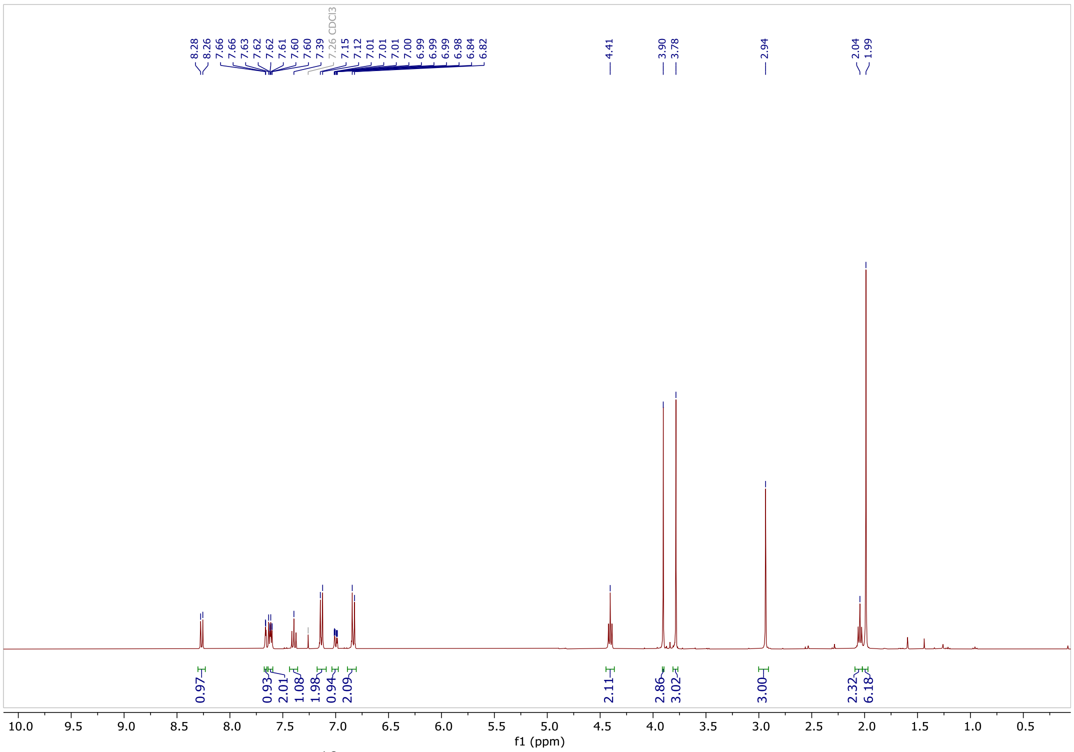

^{13}C NMR (101 MHz, $(CD_3)_2CO$))

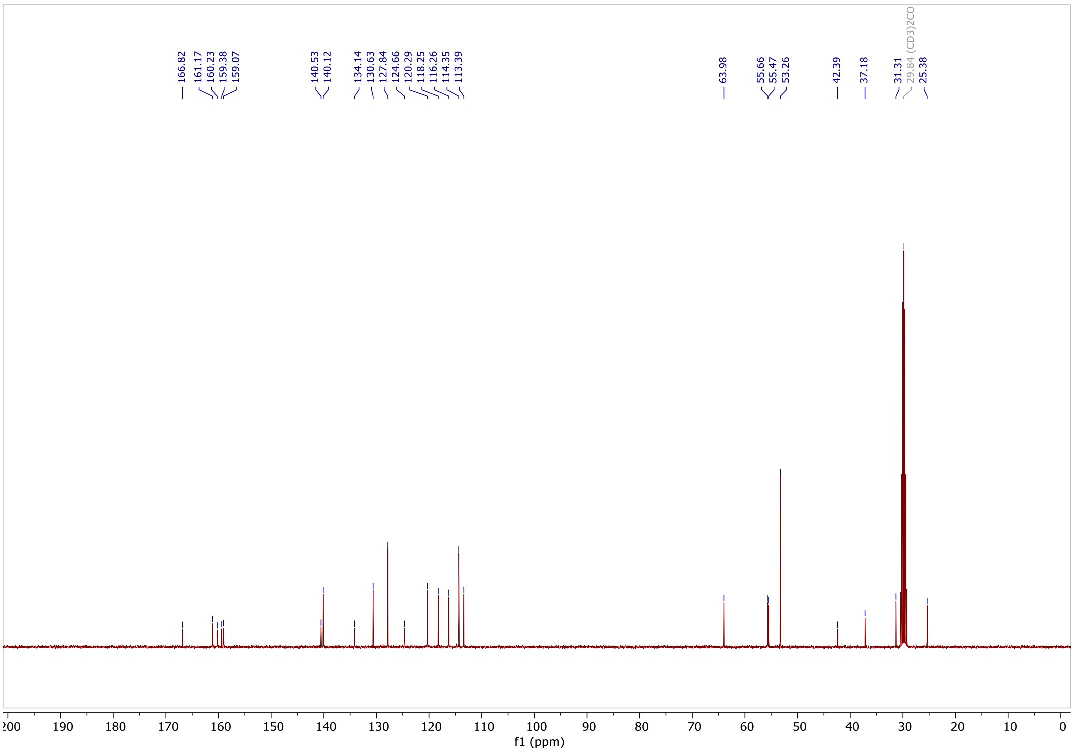

2-(3-phenylbicyclo[1.1.1]pentan-1-yl)propanoic acid, 8

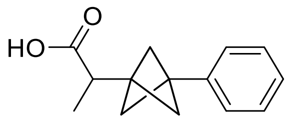

^{1}H NMR (400 MHz, $CDCl_3$)

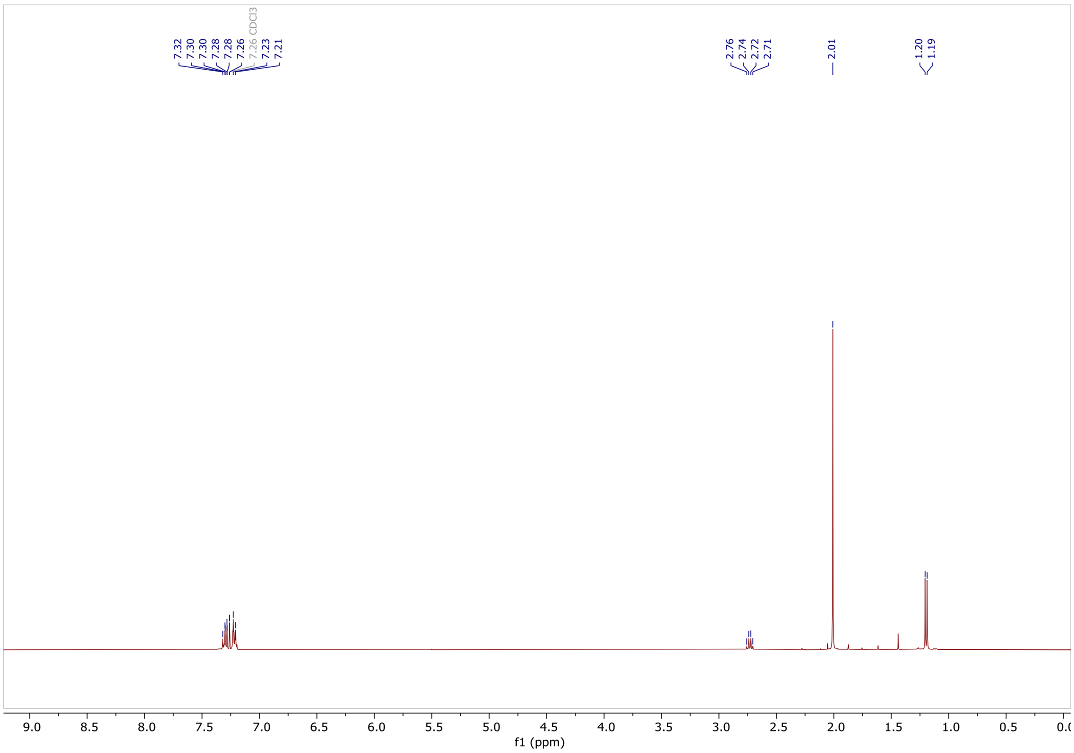

^{13}C NMR (101 MHz, $CDCl_3$)

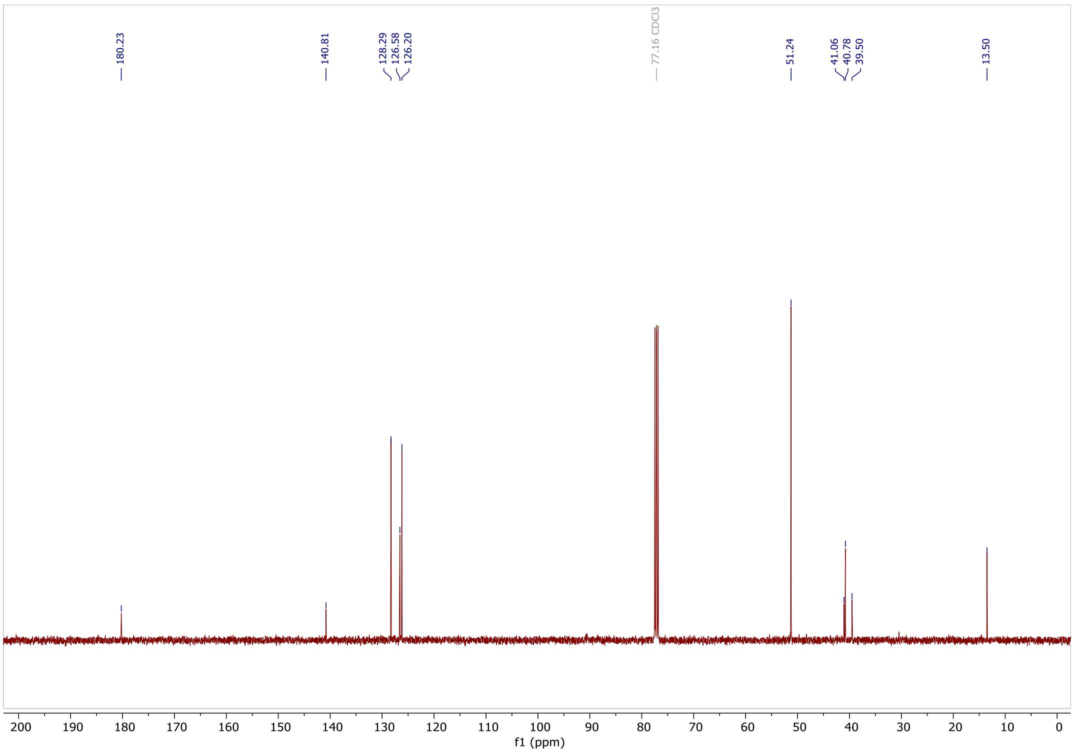

Methyl 6-fluoro-2-(3-(4-fluorophenyl)bicyclo[1.1.1]pentan-1-yl)-3-methylquinoline-4-carboxylate, 9

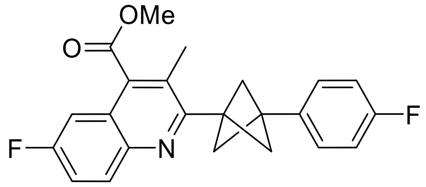

^{1}H NMR (400 MHz, $CDCl_3$)

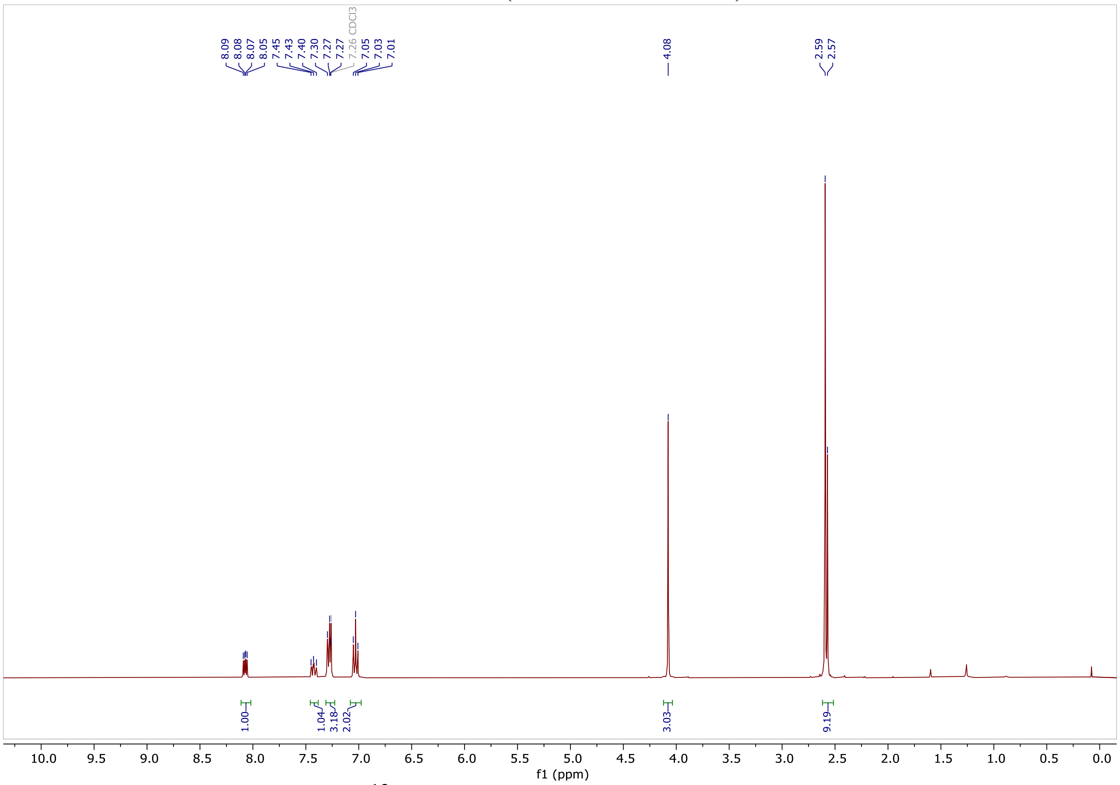

^{13}C NMR (101 MHz, $CDCl_3$)

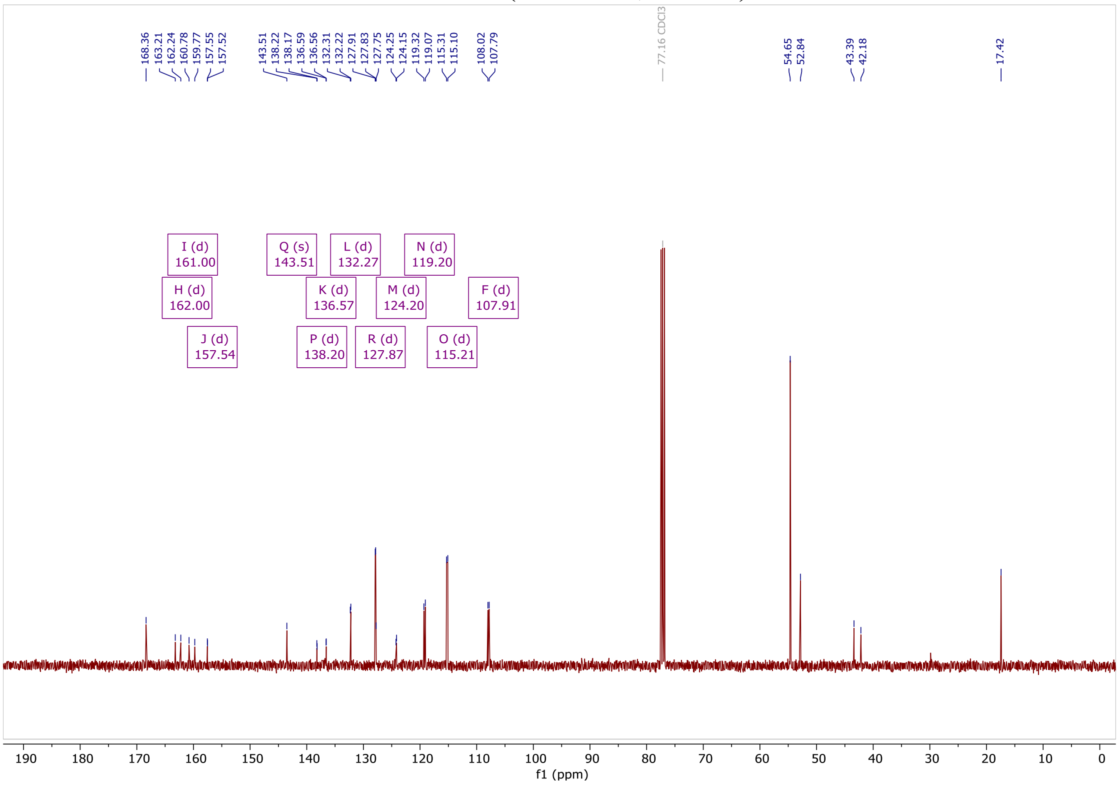

^{19}F NMR (376 MHz, $CDCl_3$)

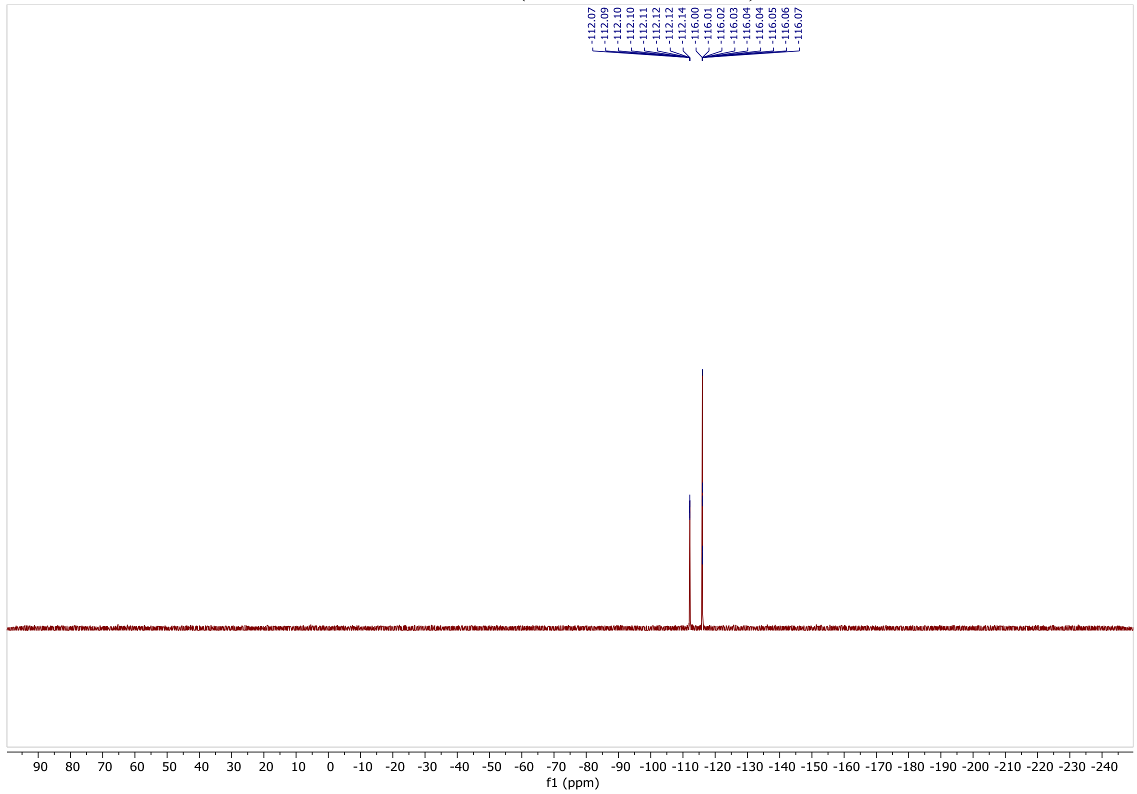

tert-butyl 4-(3-(4-bromophenyl)bicyclo[1.1.1]pentan-1-yl)piperidine-1-carboxylate, 10

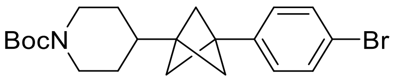

^{1}H NMR (400 MHz, $CDCl_3$)

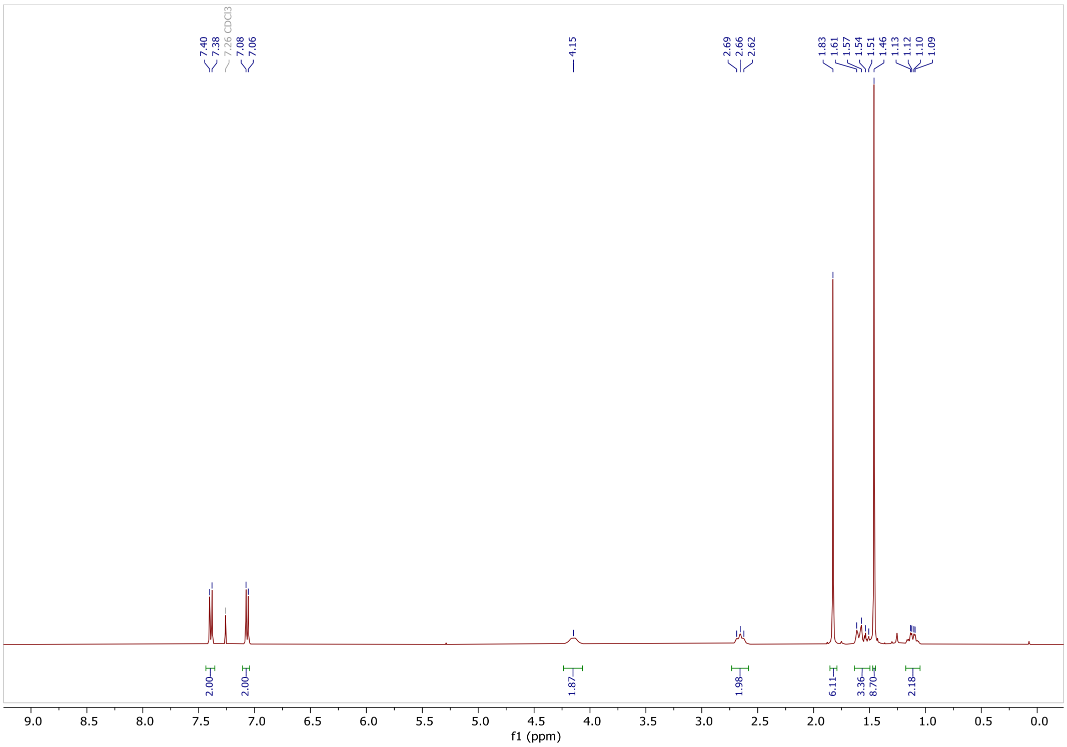

^{13}C NMR (101 MHz, $CDCl_3$)

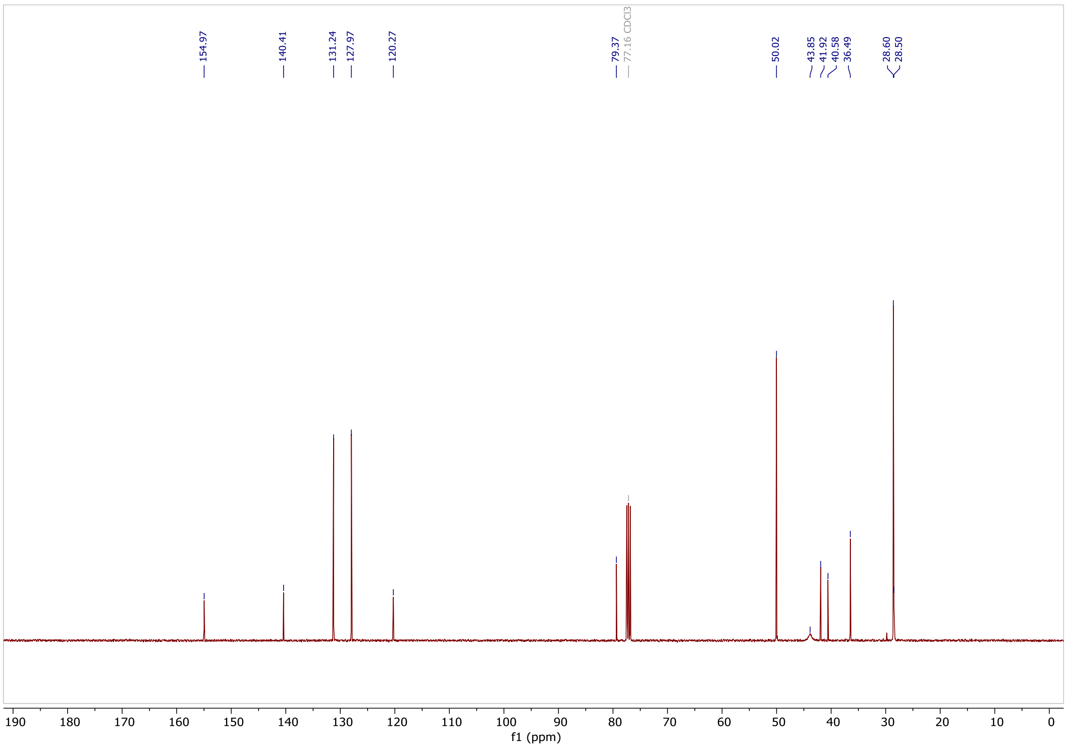

***tert*-butyl 4-(3-(4-iodophenyl)bicyclo[1.1.1]pentan-1-yl)piperidine-1-carboxylate, 11**

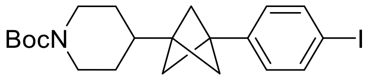

^{1}H NMR (400 MHz, $CDCl_3$)

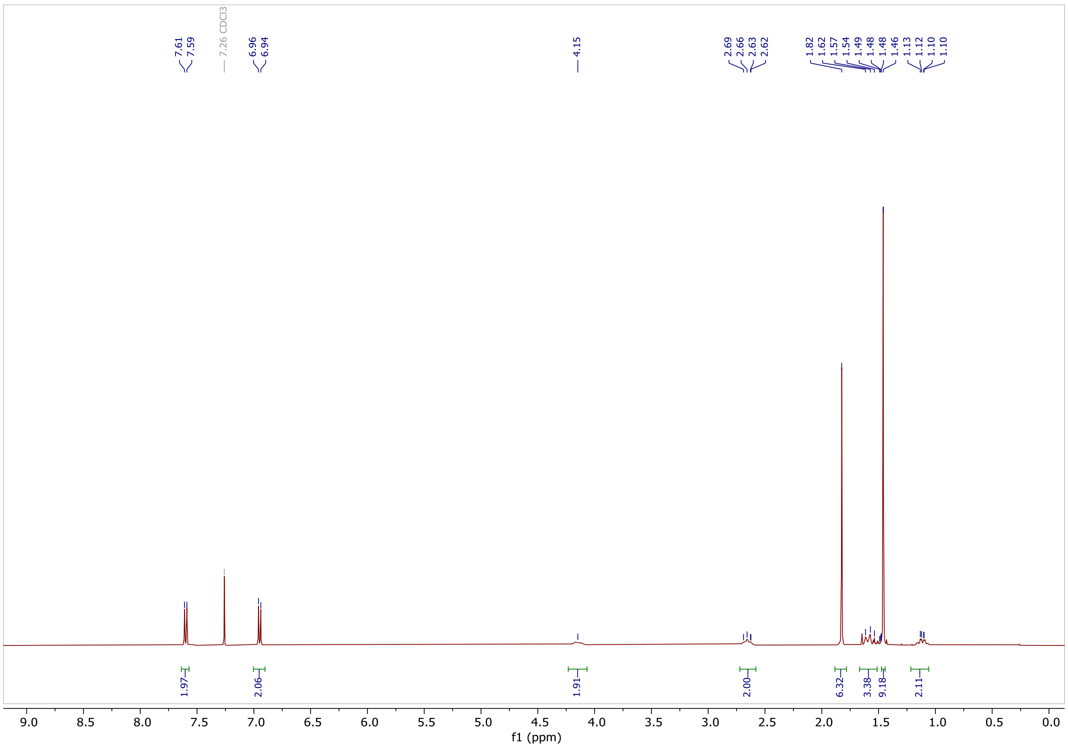

^{13}C NMR (101 MHz, $CDCl_3$)

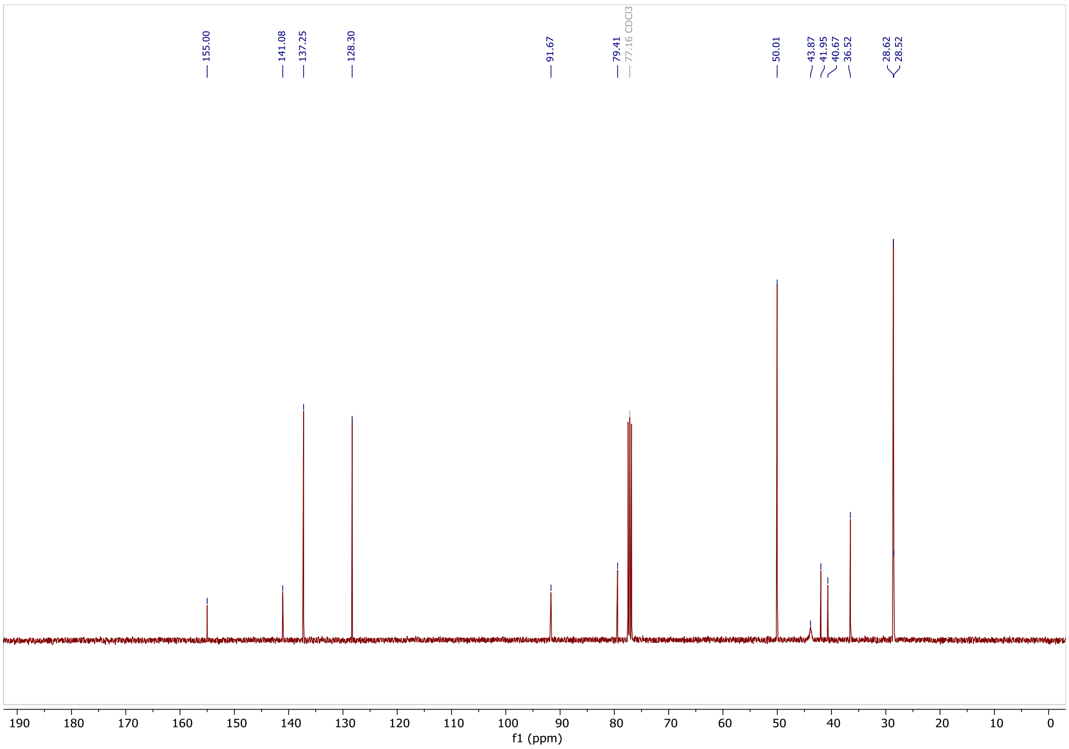

***tert*-butyl 4-(3-(4-acetylphenyl)bicyclo[1.1.1]pentan-1-yl)piperidine-1-carboxylate, 12**

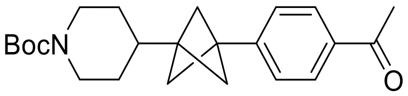

^{1}H NMR (400 MHz, $CDCl_3$)

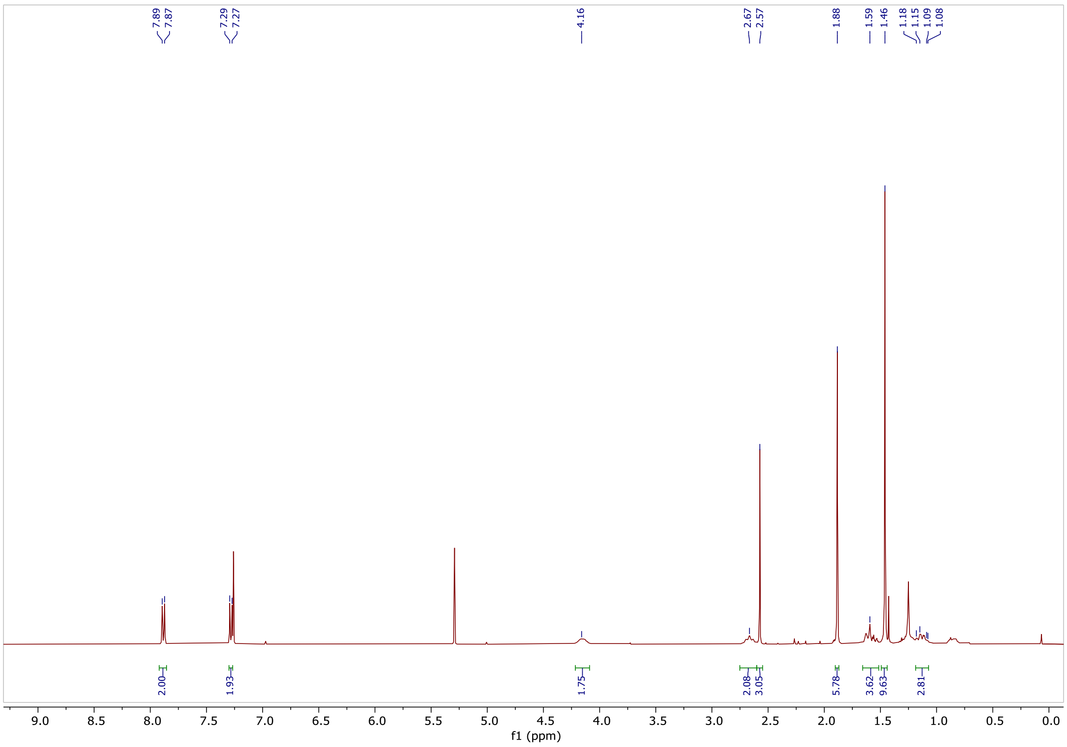

^{13}C NMR (101 MHz, $CDCl_3$)

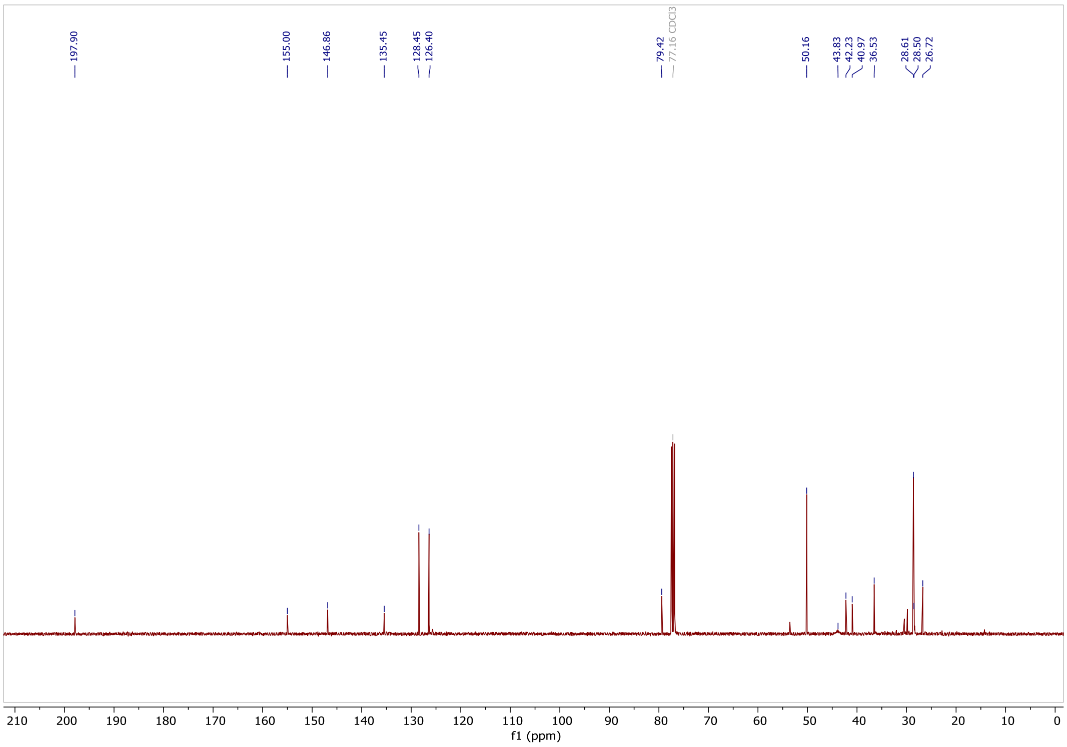

Supplement: Supplementary file 1 — Supplementary [file ANIE-59-11866-s001.pdf]
